# Synthesis, Fungicidal Activity, and Structure Activity Relationship of *β*-Acylaminocycloalkylsulfonamides against *Botrytis cinerea*

**DOI:** 10.1038/srep42096

**Published:** 2017-02-08

**Authors:** Chun-Hui Liu, Xiao-Yuan Chen, Pei-Wen Qin, Zhi-Qiu Qi, Ming-Shan Ji, Xing-Yu Liu, P. Vijaya Babu, Xing-Hai Li, Zi-Ning Cui

**Affiliations:** 1 Department of Pesticide Science, Plant Protection College, Shenyang Agricultural University, Shenyang 110866, Liaoning, China; 2 State Key Laboratory for Conservation and Utilization of Subtropical Agro-bioresources, Integrative Microbiology Research Centre, Guangdong Province Key Laboratory of Microbial Signals and Disease Control, South China Agricultural University, Guangzhou 510642, China; 3 Key Laboratory of Green Pesticide and Agricultural Bioengineering, Ministry of Education, Guizhou University, Guiyang 550025, China

## Abstract

In order to discover new antifungal agrochemicals that could have highly active and novel motifs, thirty-six new 2-acylaminocycloalkylsulfonamides (**IV**) were synthesized. Their structures were characterized and confirmed by ^1^H NMR, ^13^C NMR, IR, MS, elemental analysis and X-ray single crystal diffraction. *In vitro* and *in vivo* activities against various *Botrytis cinerea* strains were evaluated. Bioassay results revealed that most of the title compounds exhibited excellent *in vitro* fungicidal activity, in which compound **IV-26** showed the highest activity against sensitive, low-resistant, moderate-resistant and high-resistant strains of *B. cinerea* compared with the positive fungicide procymidone. Meanwhile *in vivo* fungicidal activity of compound **IV-31** was better than the commercial fungicides procymidone and chesulfamide in greenhouse trial. The structure activity relationship (SAR) was also discussed and the results were of importance to the structural optimization and development of more potent sulfonamides antifungal agents.

*Botrytis cinerea* (teleomorph: *Botryotinia fuckeliana*) is an airborne plant pathogen with a necrotrophic lifestyle attacking over 200 crop hosts worldwide. Many kinds of fungicides have been failed to control this plant disease due to its genetic plasticity^1^. Moreover, the continuous use of fungicides, such as carbendazim, diethofencarb, procymidone, and pyrimethanil *etc*, has led to the growing resistance of this plant pathogen to fungicides^2^. Thus, phytofungal disease control is urgently necessitated the discovery and development of new antifungal agents with highly active, low resistance and novel motifs for plant protection.

As very important sulfur-containing analogs of amino carboxylic acids, 2-aminoethanesulfonic acid was first isolated from ox bile in 19^th^ century by Tiedemann and Gmelin, which name ‘taurine’ was attributed by Gmelin^3^,^4^. In addition, to be an essential amino acid of human body, taurine has also shown a variety of biological functions^5^,^6^,^7^,^8^,^9^,^10^,^11^,^12^,^13^. Its derivatives had been received much more attention around the world. For example, ASPA (3-amino-2-sulfopropanoic acid, Fig. 1) and CA (2-amino-3-sulfopropanoic acid, Fig. 1), the simple substituted taurines, showed some anti-inflammatory activities^14^. As representatives of cyclic taurine derivatives, TAPS ((1*S*,2*S*)-2-aminocyclopentane-1-sulfonic acid, Fig. 1) and PSA (piperidi-3-sulfonic acid, Fig. 1) gave different effects on ATP-dependent calcium ion uptake^15^, while CAHS ((1*R*,2*S*)-2-aminocyclohexane-1-sulfonic acid, Fig. 1) and TAHS ((1*S*,2*S*)-2-aminocyclohexane-1-sulfonic acid, Fig. 1) had the thermoregulation ability *via* interaction with the central serotonergic system^16^.

Besides, 2-aminoethanesulfonic acid had been found as key structural moieties in some natural products, such as dimethyl arsenic aminosulfonate (**A**, Fig. 1), which was isolated from *Sargassum lacerifolium*^17^. Flavocristamides (**B**, Fig. 1), isolated from a marine bacterium *Flavobacterium sp*., was able to inhibit the enzyme DNA polymerase α^18^. 5-Taurinomethyluridine (**C**, Fig. 1) was discovered in mammalian mitochondrial tRNAs^19^, which was considered to be responsible for precise codon recognition and the absence of these derivatives led to mitochondrial encephalomyopathic diseases. In addition, taurine deoxyadenosine monophosphates (Tau-dAMP, **D**, Fig. 1) were recently developed as potential substrates for the HIV-1 reverse transcriptase^20^.

Studies on the synthesis and biological activity of taurine analogues 2-acylaminoethylsulfonamides have been reported frequently. For instance, *β*-aminoethanesulfonyl azides **E** (Fig. 1)^21^ and taurine-containing peptidomimetics **F** (Fig. 1)^22^ were synthesized, and 2-indole-acylsulfonamides **G** (Fig. 1)^23^ was used as myeloid cell leukemia-1 inhibitors.

While 2-aminocycloalkylsulfonic acid and its derivatives were rarely reported so far^24^,^25^, and there are no reports on the synthesis of 2-acylaminocycloalkyl-sulfonamides. Although its application in field of medicine was primarily reported, in agricultural research was still poorly applied. Recently, our group reported a series of 2-oxycycloalkylsulfonamides (**H-N**, Figs 2 and 3), which possessed highly fungicidal activity^26^,^27^,^28^,^29^, of which compound **L** (chesulfamide, Fig. 3) could be great promise and a lead compound in fungicide research and development. Based on the lead structure of compound **L**, compounds **M** and **N** (Fig. 3) were designed and synthesized with much higher fungicidal activity^30^,^31^,^32^.

These findings encouraged us to further extend the structural modification of compound **L** with the aim to find more potent antifungal agents. In this paper, 2-acylaminocycloalkylsulfonamides (**IV**, Fig. 3) were constructed by reaction of reductive amination and acylation (Fig. 4). The single-crystal structure of the title compounds **IV-3** and **IV-31** were analyzed. The fungicidal activity of the title compounds against various *B. cinerea* strains was evaluated. According to their fungicidal activities, structure-activity relationship (SAR) was also discussed.

## Results and Discussion

### Synthesis and Structure Elucidation

The synthetic route of title compounds **IV-1** to **IV-36** was outlined in Fig. 4 using 2-oxycycloalkylsulfonamides as a starting material. Reductive amination method in ref. 30 was applied to the treatment of ketones with ammonia in ethanol and titanium (IV) isopropoxide, followed by *in situ* sodium borohydride reduction. In our experiments, however, the method is improved. For the synthesis of compounds **II** from compounds **I**, the ethanol solution of ammonia was replaced directly by continuous passing of ammonia gas. The reaction was completed in a short time by monitoring ammonia gas pressure upto 20 mmHg. It was easy to operate and, and the yield of compounds **II** were from 42% to 96%. In addition, the title compounds **IV** were easily obtained by the reaction of compounds **II** with acyl chloride. Yields of title compounds **IV** were generally high (over 90%).

Crystal structures of compounds **IV-3** and **IV-31** were analyzed by X-ray single crystal diffraction. Their structures were shown in Fig. 5, and their crystal data were shown in Table S1 to Table S3. Compound **IV-3** was typical chair conformation, in which the chiralities of the 9^th^ and the 14^th^ carbon atoms on cyclohexane were *R* and *S* respectively. In addition, the bulky sulfonamide group was on equatorial bond and the smaller amide group was on axial bond. The spatial configuration was presented as *cis*-1, 2-disubstituent. Two benzene rings were far apart, which avoided the steric hindrance effect. For compound **IV-31**, the chiralities of the 3^th^ and the 9^th^ carbon atoms on cycloheptane were *S* and *R* respectively. Similarly, that of the two groups was also on the same side of the ring plane and its space conformation was *cis*-1, 2-disubstituent. Specific optical rotation of compounds **IV-3** and **IV-31** were tested as −39.2° and −0.67° respectively. Compound **IV-3** possessed specific optical rotation value of −39.2° due to the better stability of cyclohexane, while compound **IV-31** was unstable in methanol solution, of which two conformations were mutually transformed to a raceme, resulting in the optical activity disappeared.

The structures of the synthesized compounds were confirmed by ^1^H NMR, ^13^C NMR, IR, LC-MS and elemental analysis. Due to the structural similarity, all the compounds showed similar spectroscopic characteristics. In ^1^H NMR spectra of compounds **II** and **IV**, the protons on the benzene ring appeared in low field in the range of *δ*_H_ 7.0 to 8.0 ppm, while cycloalkyl group gave signals in the range of *δ*_H_ 0 to 5.0 ppm appointed to the protons of CH_3_-, CH_2_- and CH-. In addition, active hydrogen atoms of -NH_2_ and SO_2_NH- in compounds **II** appeared around 8.3 ppm, and these two types of hydrogen signals were combined together to represent a broad singlet. The reason may be that active hydrogen of SO_2_NH- is transferred to -NH_2_, forming a structure of -NH^3+^. While that of O = C-NH and SO_2_NH- in compound **IV** appeared around 8.3 ppm and 9.3 ppm respectively.

Coupling splitting of protons on CH-SO_2_, is very characteristic. Generally, the proton of CH-SO_2_ showed doublet of doublet of doublets (ddd), such as compounds **IV-5**, **IV-7**, **IV-14**, **IV-21** and **IV-23**, and corresponding splitting of proton on CH-N was triplet of doublets (td) (Fig. 6). While in the spectra of some compounds, such as compound **IV-3**, the proton of CH-SO_2_ showed doublet of doublets (dd), instead of ddd, and corresponding splitting of proton on CH-N was doublet of triplets (dt) (Fig. 7). However, there were some compounds which coupling splitting of protons on CH-SO_2_ were special because the conformation was dynamic, such as compounds **IV-13**, **IV-15**, **IV-16**, **IV-30** and **IV-32** and so on, the protons of CH-SO_2_ and CH-N showed different signal peak type. This phenomenon was very interesting, but the reason was still unknown. We choose the dominant conformation to explain the normal splitting characteristic of CH-SO_2_.

Taking the ^1^H NMR spectra of compound **IV-3** as an example (Fig. 7), according to the crystal structure, the conformation diagram (Fig. 8) of compound **IV-3** was drawn to explain the reasons for this splitting characteristic. As shown in Fig. 8, the proton of C_14_ linked -SO_2_ was located in axial bond (C_14_-H_*a*_) due to coupling splitting effects of equatorial bond (C9-H_*e*_) on C_9_, equatorial bond (C_13_-H_*e*_) and axial bond (C_13_-H_*a*_) on C_13_. Normally the proton of C_14_ linked -SO_2_ showed ddd signal due to the magnetic non-equivalence of these three protons (C_9_-H_*e*_, C_13_-H_*e*_ and C_13_-H_*a*_), appeared as dd signal. Its spatial conformation was shown in Fig. 8(a), which can be determined from the single crystal structure in Fig. 5. It showed a strong coupling splitting effect due to C_14_-H_*a*_, C_9_-H_*e*_ and C_13_-H_*e*_ lying on one side of cyclohexane plane and at a close distance, while it showed a weak coupling splitting effect due to C_13_-H_*a*_ and C_14_-Ha lying on both sides of cyclohexane plane and at a distant position, which led to its split signal invisible in the spectrum. Therefore, in ^1^H NMR spectra C_14_-H_*a*_ showed double doublets.

Correspondingly, the proton of C_9_-H_*e*_ linked -NH showed double triplets. The reason can be explained by its spatial conformation. As shown in Fig. 8(b), the difference of magnetic non-equivalence of two protons between C_10_-H_*e*_ and C_10_-H_*a*_ is small due to the protons adjacent C_10_-H_*e*_ and C_10_-H_*a*_ close to proton of C_9_-H_*e*_. So proton of C_9_-H_*e*_ affected by protons of C_10_-H_*e*_ and C_10_-H_*a*_, which signal showed coupling splitting of triplets, and protons of C_9_-H_*e*_ affected by protons of C_14_-H_*e*_, which signal showed coupling splitting of doublets. Therefore, in ^1^H NMR spectra double triplets were assigned to the proton of C9-H_*e*_.

In ^13^C NMR spectra (see supplementary information), compounds **I**, **II** and **IV** revealed signals of carbon in the range of *δ*_C_ 0 to 70 ppm assigned to methyl, methylene and methane on naphthene, and carbon signals of benzene ring and trifluoromethyl in the range of *δ*_C_ 115 to 140 ppm in low field. Compounds **I** and **IV** gave carbon signals around 202 ppm and 166 ppm respectively assigned to C = O.

In IR spectra of compounds **I** and **IV**, the absorption peak of carbonyl stretching vibration appeared around 1700 cm^−1^ and 1650 cm^−1^, respectively. While the absorption peak of imino group stretching vibration appeared around 3300 cm^−1^. In addition, the stretching vibration absorption (-NH_2_ and -SO_2_NH) of compounds **II** appeared around 3500 cm^−1^ and 3150 cm^−1^.

In LC-MS (ES^+^ mode) spectrum of **IV-1** (Fig. 9), the quasi-molecular ion peak was 491 [M + H]^+^, which accorded with the nitrogen rule. Firstly, sulfonamide bond was broken into a characteristic ion peak at *m*/*z* 296, and then fragment ion peak of *m*/*z* 135 was obtained by amide bond fracture. Finally, fragment ion peaks of *m*/*z* 92 and *m*/*z* 77 were obtained *via* McLafferty rearrangement on the benzene ring and after losing a methylene, respectively. According to the above analysis, fragment missing was reasonable.

### Bioassay of Fungicidal Activities

*B. cinerea* strains showed multiple physiological characteristics because of the different living environment and fungicide application level. As a result, sensitivities of strains from different areas are also disparate to new compounds.

### Fungicidal activity and structure-activity relationship of compounds IV-1~IV-29

In order to screen out active compounds correctly and quickly, firstly the title compounds (**IV-1**~**IV-29**) were tested against two *B cinerea* strains (Dd-15 and Sy-10), which inhibition rates were shown in Fig. 10. Two-factor analysis of variance between strains and compounds was conducted by SPSS20.0. Analytical results showed that there were sensitivity differences in the twenty-nine new compounds against the two strains. For example, the activity of the title compounds against Dd-15 was generally high, and average inhibition rate was about 76.0%. While the activity was relatively low against Sy-10, which average inhibition rate was about 58.4%. According to the analysis of bioactivity against two strains, there were twenty-one compounds, which fungicidal activities were higher than that of the positive control procymidone.

The preliminary structure-activity relationship can be summarized in four points. First, for substituent benzoyl chloride (**IV-1**~**IV-17**), fungicidal activity was mediocre on the benzene ring containing two substituents, and substituted phenyl groups at *ortho*- and *para*-position with methoxyl group and fluorine atom showed excellent activity. However, fungicidal activity was higher at *meta*-substituted methyl group and *meta*-substituted chlorine atom. When trifluoromethyl group was on the benzene ring such as compounds **IV-15** and **IV-16**, fungicidal activity was the highest. Second, for alkylacyl chloride (**IV-18**~**IV-23**), fungicidal activity of compounds showed a rising trend with the increase of carbon number in the alkyl group, for example, that of which containing *n*-hexanoyl chloride (**IV-22**) or *n*-heptanoyl chloride (**IV-23**) was the highest. Third, for halogenated acetyl chloride (**IV-24**~**IV-27**), the bioactivity increased with the increase of chlorine atom number, and activity of chloro-substituted compounds was higher than that of the bromo-substituted ones. Finally, for 2-alkoxyl acetyl chloride (**IV-28** and **IV-29**), the activity of 2-ethoxyl acetyl chloride was higher than that of the 2-methoxyl acetyl chloride. As a result, eleven highly active compounds were chosen as candidates in the second round screening.

As shown in Fig. 11, eleven compounds were screened out to determine fungicidal activity against other six different *B. cinerea* strains. Two-factor analysis of variance results showed that there remained significant differences in sensitivities of the six *B. cinerea* strains to the title compounds. For example, the average inhibition rates of eleven compounds against As-12, Cy-07, Dd-04, Dl-17, Fs-06 and Hld-16 were 51.97%, 29.17%, 62.82%, 55.84%, 35.70% and 47.74% respectively. The activities of eleven compounds against the six *B. cinerea* strains could be divided into eight subsets (a–h), in which those of compounds **IV-23**, **IV-24**, **IV-26** and **IV-29** were higher than the positive control procymidone. These four compounds were selected to do the further study and their EC_50_ values were evaluated and shown in Table 1.

Fungicidal activities of all the four title compounds were higher than that of the positive fungicide procymidone. Overall, the fungicidal activities of compound **IV-26** against six strains (As-12, Cy-07, Dd-04, Dl-17, Fs-06, and Hld-16), **IV-29** against four strains (As-12, Cy-07, Dd-04, and Fs-06), **IV-23** and **IV-24** against three strains (As-12, Cy-07, and Fs-06) were higher than those of procymidone. Activities of the four title compounds against the six *B. cinerea* strains were different, for example, EC_50_ values of compound **IV-26** against the six strains were 0.37~7.56 mg/L, while those of procymidone were 2.49~75.84 mg/L. Referring to resistant grading standards to procymidone^33^,^34^, Dd-04 and Dl-17 were low-resistant strains; As-12, Cy-07 and Hld-16 were moderate-resistant strains; Fs-06 was high-resistant strain. The results displayed that the *in vitro* activities of compound **IV-26** against all the resistant strains were excellent.

After the above test, structure-activity relationship between acyl chloride and fungicidal activity was confirmed. It was to be sure that trichloroacetyl chloride had the greatest contribution to the fungicidal activity for the twenty-nine acyl chlorides. Therefore, trichloroacetyl chloride was marked as the active group in the later structural modification of cycloalkyl group (**IV-30**~**IV-36**). Moreover, compared to the fungicidal activity screened for one strain, it was more reliable for different strains from different areas to choose as the test targets.

### Fungicidal activity and structure-activity relationship of compounds IV-26 and IV-30~IV-36

As shown in Table 2, after the structural modification of cycloalkyl group, compounds **IV-30**~**IV-36** also had very high fungicidal activity against five other *B. cinerea* strains. Referring to resistant grading standards to procymidone^33^,^34^, As-11 was sensitive strain; Dl-11 was low-resistant strain; Cy-09 was moderate-resistant strain; Fs-11 and Hld-15 were high-resistant strains. The statistical results of SPSS showed that compounds **IV-26**, **IV-30**, **IV-31**, **IV-32**, **IV-33** and **IV-34** exhibited excellent activity. It was found that the size of cycloalkyl group was important factor to determine the fungicidal activity compared with that of **IV-26**. For example, the EC_50_ values of compounds **IV-26**, **IV-30** and **IV-31** were 0.15~3.64 mg/L, 0.66~11.68 mg/L and 0.82~9.49 mg/L, respectively. The activities of compound **IV-26** containing 6-membered ring were better than those of compounds **IV-30** and **IV-31** respectively containing 5- and 7-membered ring. In addition, it was found that the types of substituent alkyl group on the cyclohexane had a significant effect on the fungicidal activity by comparing activities of compounds **IV-32**~**IV-36**. The activity decreased with the increase of alkyl carbon number. Moreover, the position of alkyl group also had effect on the activity. For example, compared with compounds **IV-32**, **IV-33** and **IV-34**, their fungicidal activity was *para*-methyl > *ortho*-methyl > *meta*-methyl in the order.

### *In vivo* fungicidal activity against *B. cinerea* on leaves of cucumber (mycelium inoculation method)

**Figure 3:**
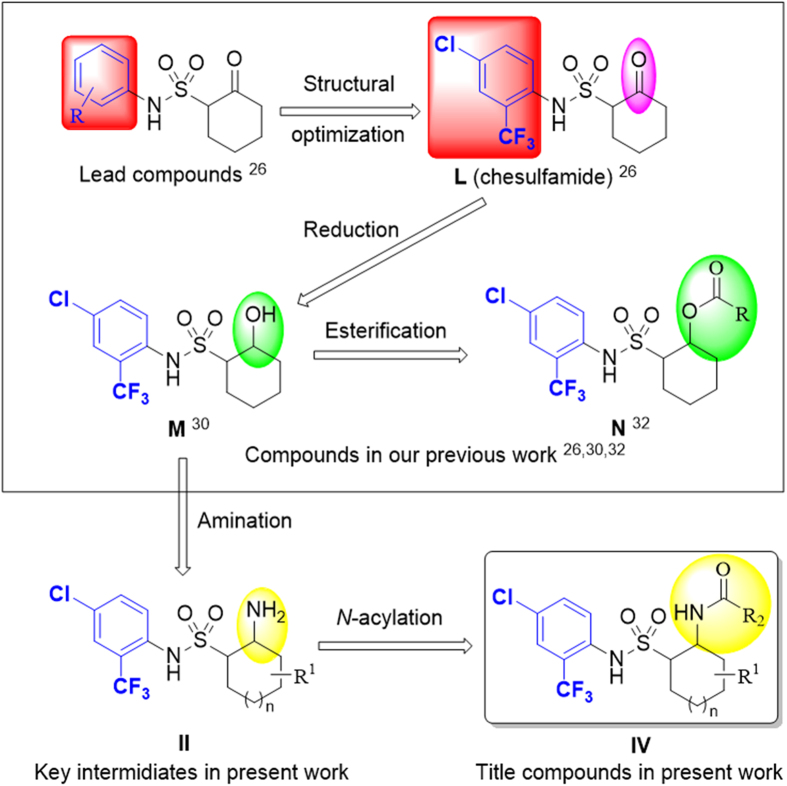
The designed strategy for the key intermediates **II** and title compounds **IV**.

Six compounds (**IV-25**, **IV-26**, **IV-30**, **IV-31**, **IV-32**, and **IV-33**) were tested for their *in vivo* fungicidal activity on leaves of cucumber, and the leading compound chesulfamide (**L**, Fig. 3) was used as the positive control. The bioassay results in Table 3 showed that the control efficiency of compound **IV-31** was significantly higher than that of the positive control chesulfamide. Fungicidal activity of compounds **IV-26**, **IV-30**, **IV-32** and **IV-33** was equivalent to the chesulfamide.

Compared with the previous work, the structure of the title compounds was modified and new. Meanwhile, their fungicidal activity had greater improvement than that of the lead compound. From the point of view of chemical synthesis, novel key intermediates 2-aminocycloalkylsulfonamides (II-1~II-8) were obtained, which had the vital significance for obtaining the title molecules with structural diversity. In addition, the effective improvements of synthesis method for compounds **II** were made, which greatly increased the yield and the reaction progress. On the other hand, structural characterization of title compounds **IV** was described in detail. In particular, the NMR spectra are very characteristic. The single crystal structure was obtained, which provided the basis for accurately structural analysis. According to the single crystal structure, the computer-aided design could be simulated, which is helpful to further molecular design and structural optimization. Preliminary mechnism study indicated that cyclohexyl alkyl sulfonamides might inhibit the growth of gray mould by affecting the synthesis of the internal substance^35^. The elucidation of the mode of action of these new compound is worth research, which will be studied in detail in the future.

## Conclusion

In conclusion, we reported the synthesis of a new series of 2**-**acylaminocycloalkylsulfonamides and their *in vitro* and *in vivo* fungicidal activities against various *B. cinerea* strains were evaluated. Some title compounds showed notable activity, especially compound **IV-31** was of great potential to be developed as new antifungal agents for plant protection. Moreover, single crystal structure of compound **IV-31** was determined to assist the further molecular design and structural modification. In addition, the SAR results indicated that structure of acylchloride and naphthenic scaffold had significant effects on the activity. Thus, the present results were of great promise for the design and development of novel sulfonamides antifungal agents. Further research was necessary on the more extensive structural modification and the broad determination of the fungicidal spectra.

## Materials and Methods

### General

Nuclear magnetic resonance (NMR) spectra were recorded in CDCl_3_ and DMSO-*d*_*6*_ unless indicated otherwise with a Bruker Avance III 600 MHz spectrometer (Bruker, Fallanden, Switzerland), using tetramethylsilane (TMS) as an internal standard. Infrared (IR) spectra were recorded on a Shimadzu IR Affinity-1 spectrophotometer (Shimadzu, Kyoto, Japan) with KBr disks. UPLC-MS/MS (Agilent, Palo Alto, CA. USA): ACQUITY UPLC BEH C_18_ chromatographic column (2.1 mm × 100 mm, 1.7 μm); column temperature: 40 °C; mobile phase: solvent A for acetonitrile, solvent B for 0.1% formic acid-water solution; gradient elution program: 10% A at the initial time of 0 min, and then 90% A~10% B in the range of 0 to 2.0 min, 50% A in the range of 2.0 to 4.0 min, 10% A~90% B in the range of 4.0 to 4.2 min, 10% A in the range of 4.2 to 5.2 min; velocity of flow: 0.2 mL/min; sampling volume: 3 μL. Ion source: ESI; acquisition methods: using multiple reaction monitoring and electrospray ionization in positive mode. Melting points were determined on an X-5 melting-point apparatus (Beijing Tech Instrument Co., Ltd., Beijing, China), and the thermometer was uncorrected. Optical rotation was measured on an automatic polarimeter (ATOGO AP-300; condition: λ = 589 nm, L = 100 mm, Temp. = 22.0 °C). The solvents and reagents were used as received or were dried prior to use, as needed. High resolution mass spectra for new compounds were recorded on a G2-XS QTof Mass Spectrometry Facility (Waters, Milford, MA, USA). Elemental analysis was carried out with a Flash EA 1112 elemantal analyzer (Thermo Finnigan, Bremen, Germany).

### Botrytis cinerea strains

Thirteen different *B. cinerea* strains, Sy-10, Dd-04, Dd-15, Hld-15, Hld-16, Fs-06, Fs-11 Dl-11, Dl-17, Cy-07, Cy-09, As-11 and As-12, were isolated from damaged parts of tomato in a greenhouse in Shenyang, Dandong, Huludao, Fushun, Dalian, Chaoyang and Anshan respectively, Liaoning Province, China, in April 2014, and cultured on potato dextrose agar (PDA) at 28 °C and maintained at 4 °C with periodic subculturing.

### Synthesis

**Figure 4:**
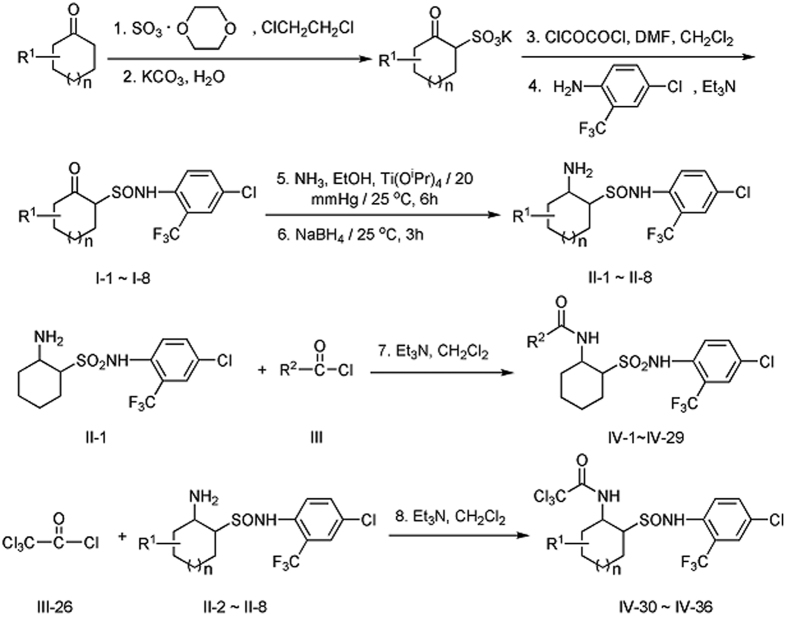
Synthetic route for the key intermediates **II** and the title compounds **IV-1** to **IV-36**.

**Figure 5:**
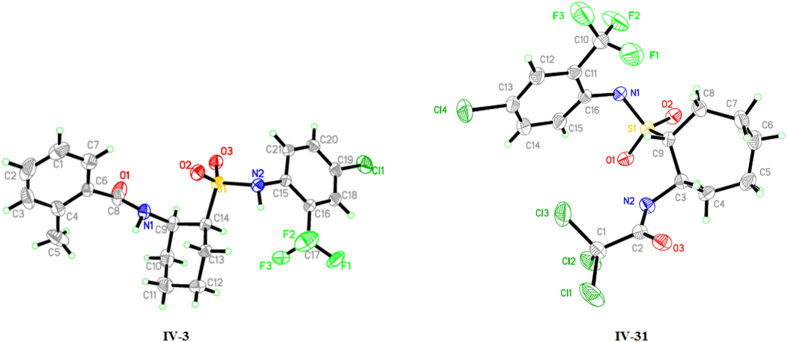
Crystal structures of **IV-3** and **IV-31**.

**Figure 6:**
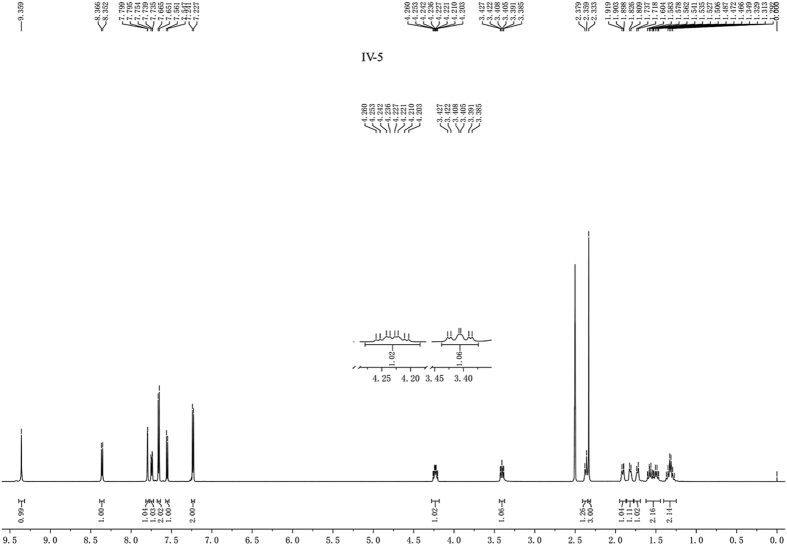
^1^H NMR spectrum of compound **IV-5**.

**Figure 7:**
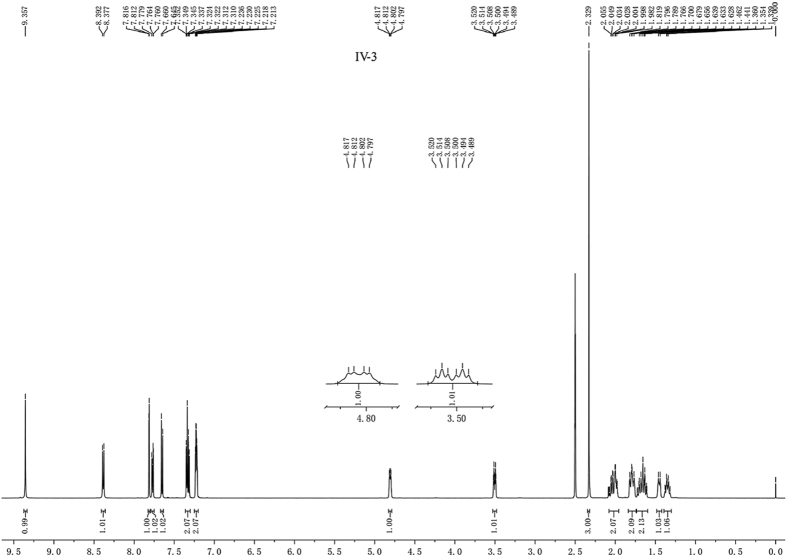
^1^H NMR spectrum of compound **IV-3**.

**Figure 8:**
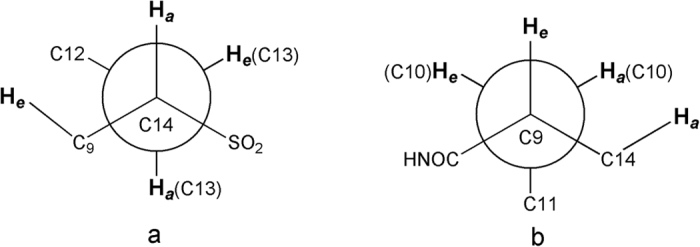
Conformation of compound **IV-3** according to the crystal structure.

**Figure 9:**
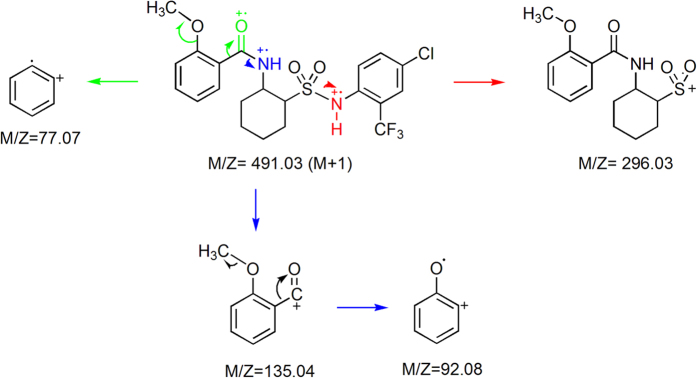
MS (ES^+^ mode) analysis of **IV-1** with the fragmentation patterns.

**Figure 10:**
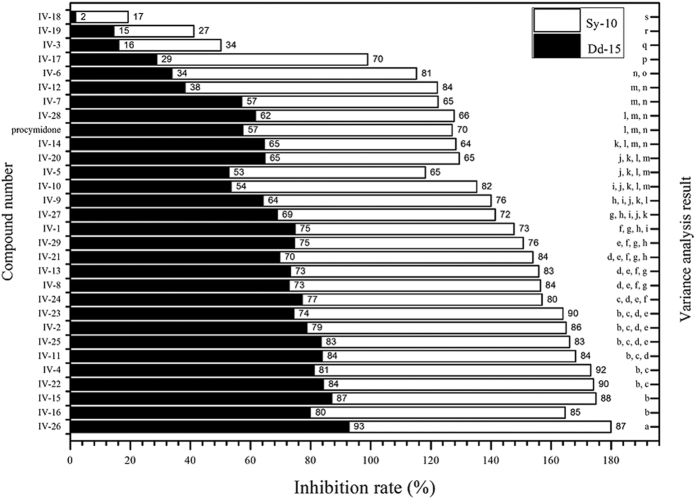
Fungicidal activity of compounds **IV-1**~**IV-29** against two *B. cinerea* strains (Sy-10 and Dd-15, 50 mg/L).

**Figure 11:**
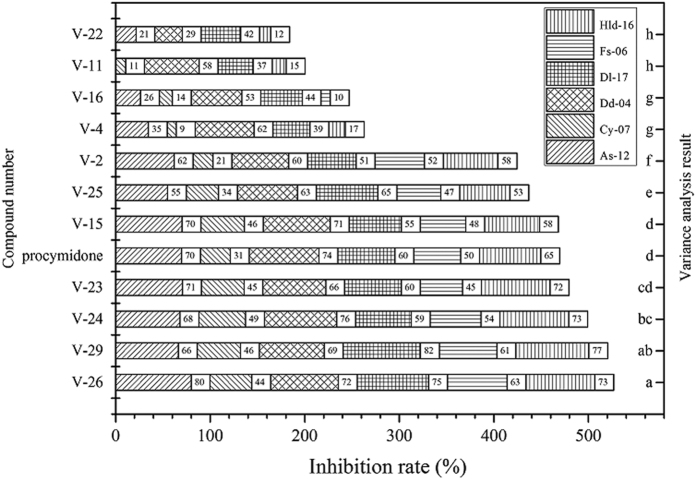
Fungicidal activity of title compounds **IV** against six *B. cinerea* strains (50 mg/L).

The synthetic routes of the key intermediates **II** and title compounds **IV** were outlined in Fig. 4.

### Synthesis of *N*-(2-trifluoromethyl-4-chlorophenyl)-2-oxocyclohexylsulfonamides I-1~I-8

Compounds **I** were synthesized according to the method given in the ref. 26. The synthetic route of compounds **I-1** to **I-8** was outlined in Fig. 4. **I-1** (n = 1, R^1^ = H), **I-2** (n = 0, R^1^ = H), **I-3** (n = 2, R^1^ = H) were already known^30^ and **I-4**~**I-8** are new compounds. Their physical data and spectra data were shown as follows:

### *N*-(2-trifluoromethyl-4-chlorophenyl)-3-methyl-2-oxocyclohexylsulfonamide (I-4)

(n = 1, R^1^ = 3-Me) Colorless crystal; yield, 71%; mp 108–109 °C; ^1^H NMR (CDCl_3_) *δ*: 1.11 (d, *J* = 6.4 Hz, 3H, CH_3_), 1.47–2.64 (m, 7H, C_4_H_7_), 3.97 (dd, *J* = 13.4, 5.3 Hz, 1H, CH-SO_2_), 7.37 (s, 1H, SO_2_-NH), 7.51–7.71 (m, 3H, Ph-H); ^13^C NMR (DMSO-*d*_*6*_) *δ:* 14.41, 23.59, 30.20, 35.94, 45.28, 70.74, 118.99, 121.97, 123.79, 126.94, 131.63, 132.20, 133.43, 204.54; IR (*ν*, cm^−1^): 3344, 1708; MS (z/e): 369(M^+^), 195, 175, 111, 83, 55; Anal. Calcd for C_14_H_15_ClF_3_NO_3_S: C, 45.47; H, 4.09; N, 3.79; found: C, 45.31; H, 3.94; N, 3.92.

### *N*-(2-trifluoromethyl-4-chlorophenyl)-4-methyl-2-oxocyclohexylsulfonamide (I-5)

(n = 1, R^1^ = 4-Me) Colorless crystal; yield, 91%; mp 97–99 °C; ^1^H NMR (CDCl_3_) *δ*: 1.34–2.62 (m, 10H, C_5_H_10_), 3.90 (dd, *J* = 13.0, 5.7 Hz, 1H, CH-SO_2_), 7.35 (s, 1H, SO_2_-NH), 7.51–7.71 (m, 3H, Ph-H); ^13^C NMR (DMSO-*d*_*6*_) *δ:* 21.84, 26.24, 28.86, 34.55, 48.07, 69.24, 118.64, 118.99, 125.61, 126.98, 132.23, 133.28, 145.66, 202.41; IR (*ν*, cm^−1^): 3365, 1708; MS (z/e): 369(M^+^), 148, 131, 126, 120, 91; Anal. Calcd for C_14_H_15_ClF_3_NO_3_S: C, 45.47; H, 4.09; N, 3.79; found: C, 45.63; H, 3.98; N, 3.57.

### *N*-(2-trifluoromethyl-4-chlorophenyl)-5-methyl-2-oxocyclohexylsulfonamide(I-6)

(n = 1, R^1^ = 5-Me) Colorless crystal; yield, 94%; mp 104–105 °C; ^1^H NMR (CDCl_3_) *δ*: 1.07–2.63 (m, 10H, C_5_H_10_), 3.99 (dd, *J* = 13.3, 5.4 Hz, 1H, CH-SO_2_), 7.37 (s, 1H, SO_2_-NH), 7.51–7.69 (m, 3H, Ph-H); ^13^C NMR (DMSO-*d*_*6*_) *δ:* 21.16, 26.44, 30.32, 34.52, 36.63, 69.63, 119.00, 121.97, 123.78, 126.91, 131.69, 132.19, 133.44, 203.09; IR (*ν*, cm^−1^): 3367, 1710; MS (z/e): 369(M^+^), 352, 306, 195, 175, 55; Anal. Calcd for C_14_H_15_ClF_3_NO_3_S: C, 45.47; H, 4.09; N, 3.79; found: C, 45.66; H, 4.31; N, 3.59.

### *N*-(2-trifluoromethyl-4-chlorophenyl)-5-ethyl-2-oxocyclohexylsulfonamide (I-7)

(n = 1, R^1^ = 5-Et) Colorless crystal; yield, 99%; mp 90~93 °C; ^1^H NMR (CDCl_3_) *δ*: 0.96–2.66 (m, 12H, C_6_H_12_), 3.98 (dd, *J* = 12.5, 5.4 Hz, 1H, CH-SO_2_), 7.38 (s, 1H, SO_2_-NH), 7.52–7.70 (m, 3H, Ph-H); ^13^C NMR (DMSO-*d*_*6*_) *δ:* 11.80, 28.21, 32.23, 34.41, 36.63, 41.27, 69.70, 121.97, 123.79, 126.91, 131.70, 132.21, 133.46, 133.82, 203.20; IR (*ν*, cm^−1^): 3375, 1714; MS (z/e): 383(M^+^), 366, 320, 195, 175, 55; MS (z/e): 383(M^+^), 366, 320, 195, 175, 55; Anal. Calcd for C_15_H_17_ClF_3_NO_3_S: C, 46.94; H, 4.46; N, 3.65; found: C, 47.11; H, 4.37; N, 3.78.

### *N*-(2-trifluoromethyl-4-chlorophenyl)-5-tertiarybutyl-2-oxocyclohexylsulfonamide (I-8)

(n = 1, R^1^ = 5-*t*-Bu) Colorless crystal; yield, 93%; mp 86–89 °C; ^1^H NMR (CDCl_3_) *δ*: 0.93–2.70 (m, 16H, C_8_H_16_), 3.95 (dd, *J* = 13.3, 5.3 Hz, 1H, CH-SO_2_), 7.38 (s, 1H, SO_2_-NH), 7.52–7.70 (m, 3H, Ph-H); ^13^C NMR (DMSO-*d*_*6*_) *δ:* 8.97, 27.63, 30.23, 32.64, 41.31, 44.82, 69.96, 119.01, 123.78, 126.91, 131.76, 132.28, 133.49, 133.99, 203.08; IR (*ν*, cm^−1^): 3329, 1714; MS (z/e): 280, 194, 175, 154, 69, 57; Anal. Calcd for C_17_H_21_ClF_3_NO_3_S; C, 49.57; H, 5.14; N, 3.40; found; C, 49.68; H, 4.95; N, 3.61.

### Synthesis of the key intermediates *N*-(2-trifluoromethyl-4-chlorophenyl)-2-aminocycloalkylsulfonamides II-1~II-8

The synthetic route of compounds **II-1** *to* **II-8** was outlined in Fig. 4, according to the method given in the ref. 36, under a nitrogen atmosphere, compounds **I** (30 mmol) and titanium (IV) isopropoxide (17 mL, 60 mmol) in dry ethyl alcohol (150 mL) were stirred, while the ammonia gas passed through the reaction mixture and maintained the pressure of ammonia upto 20 mmHg at room temperature for 6 h, which was monitored by TLC analysis. Then sodium borohydride (1.7 g, 45 mmol) was added slowly to the resulting mixture at room temperature and stirred for 3 h. The reaction was quenched by addition of ammonium hydroxide solution (2 M, 120 mL). The resulting inorganic precipitate was filtered off, and washed with ethyl acetate (150 mL). The filtrate was concentrated under reduced pressure to remove ethyl acetate, and then extracted with ethyl acetate (200 mL). The combined organic extracts were washed with brine (300 mL), dried over anhydrous Na_2_SO_4_, evaporated under reduced pressure, and recrystallized from methanol to afford pure key intermediates **II**. Their physical and spectra data were shown as follows.

### *N*-(2-trifluoromethyl-4-chlorophenyl)-2-aminocyclohexylsulfonamide (II-1)

(n = 1, R^1^ = H) Colorless crystal, yield, 73%; mp 252–254 °C; ^1^H NMR (DMSO*-d*_*6*_) *δ*: 1.32–2.00 (m, 8H, 4CH_2_), 2.89 (dt, *J* = 12.4, 3.1 Hz, 1H, CH-N), 3.79 (d, *J* = 2.1 Hz, 1H, CH-SO_2_), 7.27–7.42 (m, 3H, Ph-H), 8.21 (s, 3H, NH_2_ + NH); ^13^C NMR (DMSO-*d*_*6*_) *δ*: 24.07, 24.17, 25.09, 30.36, 50.21, 60.99, 118.65, 122.25, 123.87, 125.68, 125.84, 132.23, 147.54; IR (*ν*, cm^−1^): 3516, 3078; MS (z/e): 357[M + H]^+^, 175, 162, 98, 81; Anal. Calcd for C_17_H_21_ClF_3_NO_3_S: C, 43.76; H, 4.52; N, 7.85. found: C, 43.88; H, 4.69; N, 7.61.

### *N*-(2-trifluoromethyl-4-chlorophenyl)-2-aminocyclopentylsulfonamide (II-2)

(n = 0, R^1^ = H) White powder; yield, 95%; mp 183–186 °C; ^1^H NMR (DMSO*-d*_*6*_) *δ*: 1.52–2.05 (m, 6H, 3CH_2_), 3.40–3.44(m, 1H, CH-N), 3.64 (dd, *J* = 11.6, 6.6 Hz, 1H, CH-SO_2_), 7.27–7.47 (m, 3H, Ph-H), 8.14 (s, 3H, NH_2_ + NH); ^13^C NMR (DMSO-*d*_*6*_) *δ*: 21.72, 26.00, 30.71, 51.90, 61.45, 118.71, 121.93, 123.88, 125.68, 125.86, 132.24, 147.70; IR (*ν*, cm^−1^): 3614, 3198; MS (z/e): 342(M^+^), 196, 176, 148, 84, 67; Anal. Calcd for C_12_H_14_ClF_3_N_2_O_2_S: C, 42.05; H, 4.12; N, 8.17; found: C, 41.92; H, 3.98; N, 8.35.

### *N*-(2-trifluoromethyl-4-chlorophenyl)-2-aminocycloheptyl sulfonamide (II-3)

(n = 2, R^1^ = H) White powder; yield, 91%; mp 230–232 °C; ^1^H NMR (DMSO*-d*_*6*_) *δ*: 1.41–2.28 (m, 10H, 5CH_2_), 2.92 (dd, J = 10.0, 2.2 Hz, 1H, CH-N), 3.99 (td, J = 5.5, 2.4 Hz, 1H, CH-SO_2_), 7.27–7.39 (m, 3H, Ph-H), 8.27 (s, 3H, NH_2_ + NH); ^13^C NMR (DMSO-*d*_*6*_) *δ*: 21.81, 22.09, 25.95, 27.41, 32.06, 49.81, 61.71, 118.64, 122.28, 123.92, 125.73, 125.80, 132.23, 147.29; IR (*ν*, cm^−1^): 3523, 3095; MS (z/e): 370, 194, 174; Anal. Calcd for C_14_H_18_ClF_3_N_2_O_2_S; C, 45.35; H, 4.89; N, 7.55; found; C, 45.21; H, 5.02; N, 7.63.

### *N*-(2-trifluoromethyl-4-chlorophenyl)-3-methyl-2-aminocyclohexylsulfonamide (II-4)

(n = 1, R^1^ = 3-Me) White powder; yield, 79%; mp 213–216 °C; ^1^H NMR (DMSO*-d*_*6*_) *δ*: 0.85–2.11 (m, 10H, C_5_H_10_), 2.83 (td, *J* = 11.5, 3.5 Hz, 1H, CH-N), 3.18 (td, *J* = 11.4, 4.4 Hz, 1H, CH-SO_2_), 7.27–7.42 (m, 3H, Ph-H), 8.34 (s, 3H, NH_2_ + NH); ^13^C NMR (DMSO-*d*_*6*_) *δ*: 17.05, 19.17, 21.96, 24.89, 31.34, 52.13, 56.10, 118.64, 122.26, 123.87, 125.68, 125.79, 132.20, 147.29; IR (*ν*, cm^−1^): 3599, 3140; MS (z/e): 370(M^+^), 176, 112, 95, 67; Anal. Calcd for C_14_H_18_ClF_3_N_2_O_2_S: C, 45.35; H, 4.89; N, 7.55; found: C, 45.18; H, 4.62; N, 7.69.

### *N*-(2-trifluoromethyl-4-chlorophenyl)-4-methyl-2-aminocyclohexylsulfonamide (II-5)

(n = 1, R^1^ = 4-Me) White powder; yield, 86%; mp 230–233 °C; ^1^H NMR (DMSO*-d*_*6*_) *δ*: 0.74–2.08 (m, 10H, C_5_H_10_), 3.11 (s, 1H, CH-N), 3.29 (s, 1H, CH-SO_2_), 7.22–7.51 (m, 3H, Ph-H), 8.25 (s, 3H, NH_2_ + NH); ^13^C NMR (DMSO-*d*_*6*_) *δ*: 22.27, 25.40, 28.00, 31.26, 33.51, 50.43, 55.57, 118.60, 122.07, 123.86, 125.66, 125.83, 132.15, 147.74; IR (*ν*, cm^−1^): 3523, 3072; MS (z/e): 370(M^+^), 176, 112, 95, 67, 55; Anal. Calcd for C_14_H_18_ClF_3_N_2_O_2_S: C, 45.35; H, 4.89; N, 7.55; found: C, 45.56; H, 4.69; N, 7.41.

### *N*-(2-trifluoromethyl-4-chlorophenyl)-5-methyl-2-aminocyclohexylsulfonamide (II-6)

(n = 1, R^1^ = 5-Me) White powder; yield, 85%; mp 250–252 °C; ^1^H NMR (DMSO*-d*_*6*_) *δ*: 0.90–2.12 (m, 10H, C_5_H_10_), 2.83 (td, *J* = 11.5, 3.5 Hz, 1H, CH-N), 3.18 (td, *J* = 11.4, 4.4 Hz, 1H, CH-SO_2_), 7.28–7.42 (m, 3H, Ph-H),8.35 (s, 3H, NH_2_ + NH); ^13^C NMR (DMSO-*d*_*6*_) *δ*: 22.14, 25.68, 30.73, 32.35, 50.00, 55.70, 60.67, 118.52, 118.67, 122.14, 122.28, 125.85, 132.23, 147.44; IR (*ν*, cm^−1^): 3523, 3170; MS (z/e): 370(M^+^), 278, 250, 197; Anal. Calcd for C_14_H_18_ClF_3_N_2_O_2_S: C, 45.35; H, 4.89; N, 7.55; found: C, 45.57; H, 4.98; N, 7.38.

### *N*-(2-trifluoromethyl-4-chlorophenyl)-5-ethyl-2-aminocyclohexylsulfonamide (II-7)

(n = 1, R^1^ = 5-Et) White powder; yield, 54%; mp 227–230 °C; ^1^H NMR (DMSO*-d*_*6*_) *δ*: 0.81–2.04 (m, 12H, C_6_H_12_), 3.05–3.06 (m, 1H, CH-N), 3.55–3.56 (m, 1H, CH-SO_2_), 7.26–7.46 (m, 3H, Ph-H), 8.19 (s, 3H, NH_2_ + NH); ^13^C NMR (DMSO-*d*_*6*_) *δ*: 12.02, 24.62, 27.03, 29.29, 32.53, 37.50, 46.60, 55.72, 118.55, 122.07, 122.27, 125.84, 132.15, 132.23, 147.67; IR (*ν*, cm^−1^): 3523, 3277; MS (z/e): 384(M^+^), 194, 174, 95, 67, 56; Anal. Calcd for C_15_H_20_ClF_3_N_2_O_2_S: C, 46.81; H, 5.24; N, 7.28; found: C, 47.02; H, 5.06; N, 7.51.

### *N*-(2-trifluoromethyl-4-chlorophenyl)-5-tertiarybutyl-2-aminocyclohexylsulfonamide (II-8)

(n = 1, R^1^ = 5-*t*-Bu) White powder; yield, 42%; mp 230–233 °C; ^1^H NMR (DMSO*-d*_*6*_) *δ*: 0.77–2.45 (m, 16H, C_8_H_16_), 2.87 (d, *J* = 12.2 Hz, 1H, CH-N), 3.76 (s, 1H, CH-SO_2_), 7.27–7.41 (m, 3H, Ph-H), 8.20 (s, 3H, NH_2_ + NH); ^13^C NMR (DMSO-*d*_*6*_) *δ*: 19.56, 22.51, 27.66, 28.86, 32.74, 46.15, 46.23, 60.46, 118.64, 122.32, 123.90, 125.70, 125.82, 132.18, 147.31; IR (*ν*, cm^−1^): 3502, 3109; MS (z/e): 412(M^+^), 397, 355, 194, 154; Anal. Calcd for C_17_H_24_ClF_3_N_2_O_2_S: C, 49.45; H, 5.86; N, 6.78; found: C, 49.25; H, 6.04; N, 6.66.

### Synthesis of acyl chlorides III

Substituent benzoyl chlorides (**III-**1~**III-**17), acetyl chlorides (**III-**18~**III-**23), halogenated acetyl chlorides (**III-**24~**III-**27), alkoxylacetyl chlorides (**III-**28~**III**-29) were synthesized according to the given method in the ref. 32.

### Synthesis of title compounds 2-acylaminocycloalkylsulfonamides IV-1~IV-29 and IV-30~IV-36

Under nitrogen, acyl chlorides **III** (3 mmol) were dropwise added to the solution of **II** (3 mmol) and triethylamine (Et_3_N, 3.9 mmol) in dry dichloromethane (40 mL). (Fig. 4) The solution was stirred at room temperature for 2 h. The mixture was filtered and washed with 3 M HCl (30 mL), saturated NaHCO_3_ (30 mL), and brine (40 mL). After dried by anhydrous Na_2_SO_4_ and concentrated *in vacuo*, the crude product was recrystallized with the acetone/petroleum ether to afford pure **IV**. Their physical data and spectra data were shown as follows.

### *N*-(2-trifluoromethyl-4-chlorophenyl)-2-(2-methoxybenzoylamino) cyclohexylsulfonamide (IV-1)

(R^2^ = 2-CH_3_OC_6_H_4_) White solid; yield, 95%; mp 129–130 °C; ^1^H NMR (DMSO*-d*_*6*_) *δ*: 1.48–2.14 (m, 8H, 4CH_2_), 3.55 (dt, *J* = 11.9, 3.3 Hz, 1H, CH-N), 3.94 (s, 3H, OCH_3_), 4.64 (dd, *J* = 6.9, 3.4 Hz, 1H, CH-SO_2_), 7.05–7.89 (m, 7H, Ph-H), 8.60 (d, *J* = 7.3 Hz, 1H, CO-NH), 9.57 (s, 1H, SO_2_-NH); ^13^C NMR (DMSO-*d*_*6*_) *δ*: 20.07, 22.53, 24.01, 29.88, 45.54, 56.60, 62.79, 112.70, 121.11, 121.96, 122.02, 126.85, 127.07, 131.24, 131.29, 131.49, 133.21, 133.64, 133.84, 157.79, 164.40; IR (*ν*, cm^−1^): 3370, 3121, 1649; MS (z/e): 491[M + H]^+^, 296, 135, 92, 77; Anal. Calcd for C_21_H_22_ClF_3_N_2_O_4_S: C,51.38; H, 4.52; N, 5.71; found: C, 51.62; H, 4.69; N, 5.52.

### *N*-(2-trifluoromethyl-4-chlorophenyl)-2-(4-methoxybenzoylamino) cyclohexylsulfonamide (IV-2)

(R^2^ = 4-CH_3_OC_6_H_4_) White solid; yield, 86%; mp 139–141 °C; ^1^H NMR (DMSO*-d*_*6*_) *δ*: 1.37–2.17 (m, 8H, 4CH_2_), 3.51 (dt, *J* = 11.8, 3.4 Hz, 1H, CH-N), 3.81 (s, 3H, OCH_3_), 4.72 (dd, *J* = 7.9, 3.6 Hz, 1H, CH-SO_2_), 6.97–7.79 (m, 7H, Ph-H), 7.91 (d, *J* = 8.5 Hz, 1H, CO-NH), 9.43 (s, 1H, SO_2_-NH); ^13^C NMR (DMSO-*d*_*6*_) *δ*: 19.71, 21.72, 24.15, 30.65, 45.26, 55.74, 63.08, 113.62, 113.62, 122.05, 123.87, 126.55, 127.07, 127.41, 129.98, 131.01, 131.26, 133.62, 134.04, 161.95, 166.86; IR (*ν*, cm^−1^): 3390, 3080, 1631; HRMS-ESI, m/z calcd for C_21_H_23_ClF_3_N_2_O_4_S, [M + H]^+^491.1019; found, 491.1022.

### *N*-(2-trifluoromethyl-4-chlorophenyl)-2-(2-methylbenzoylamino) cyclohexylsulfonamide (IV-3)

(R^2^ = 2-CH_3_C_6_H_4_) White crystal; yield, 96%; mp 192–194 °C; ^1^H NMR (DMSO*-d*_*6*_) *δ*: 1.36–1.96 (m, 8H, 4CH_2_), 2.33 (s, 3H, CH_3_), 3.50 (dt, *J* = 11.9, 3.6 Hz, 1H, CH-N), 4.81 (dd, *J* = 8.9, 3.1 Hz, 1H, CH-SO_2_), 7.20–7.81 (m, 7H, Ph-H), 8.38 (d, *J* = 9.2 Hz, 1H, CO-NH), 9.36 (s, 1H, SO_2_-NH); ^13^C NMR (DMSO-*d*_*6*_) *δ*: 19.40, 19.55, 21.36, 24.39, 31.18, 44.43, 63.36, 122.08, 123.89, 125.57, 126.97, 128.00, 129.56, 130.46, 130.68, 131.03, 133.62, 134.15, 135.86, 137.37, 169.89; IR (*ν*, cm^−1^): 3388, 3074, 1645; MS (z/e): 475[M + H]^+^, 280, 216, 119, 91; Anal. Calcd for C_21_H_22_ClF_3_N_2_O_3_S: C,53.11; H, 4.67; N, 5.90; found: C, 53.35; H, 4.50; N, 6.09.

### *N*-(2-trifluoromethyl-4-chlorophenyl)-2-(3-methylbenzoylamino) cyclohexylsulfonamide (IV-4)

(R^2^ = 3-CH_3_C_6_H_4_) White crystal; yield, 43%; mp 176–177 °C; ^1^H NMR (DMSO*-d*_*6*_) *δ*: 1.32–2.12 (m, 8H, 4CH_2_), 2.36 (s, 3H, CH_3_), 3.54–3.49 (m, 1H, CH-N), 4.74 (d, *J* = 4.3 Hz, 1H, CH-SO_2_), 7.34–7.80 (m, 7H, Ph-H), 8.04 (d, *J* = 8.6 Hz, 1H, CO-NH), 9.42 (s, 1H, SO_2_-NH); ^13^C NMR (DMSO-*d*_*6*_) *δ*: 19.71, 21.32, 21.71, 24.15, 30.61, 45.27, 63.04, 122.05, 123.87, 125.31, 127.02, 128.30, 128.60, 130.95, 131.23, 132.01, 133.62, 134.03, 135.29, 137.60, 167.60; IR (*ν*, cm^−1^): 3385, 3046, 1629; MS (z/e): 475[M + H]^+^, 280, 216, 119, 91; Anal. Calcd for C_21_H_22_ClF_3_N_2_O_3_S: C, 53.11; H, 4.67; N, 5.90; found: C, 52.94; H, 4.57; N, 5.71.

### *N*-(2-trifluoromethyl-4-chlorophenyl)-2-(4-methylbenzoylamino) cyclohexylsulfonamide (IV-5)

(R^2^ = 4-CH_3_C_6_H_4_) White solid; yield, 98%; mp 217–219 °C; ^1^H NMR (DMSO*-d*_*6*_) *δ*: 1.32–2.37 (m, 8H, 4CH_2_), 2.33 (s, 3H, CH_3_), 3.41 (td, *J* = 11.1, 2.9 Hz, 1H, CH-N), 4.23 (ddd, *J* = 19.2, 10.6, 4.0 Hz, 1H, CH-SO_2_), 7.23–7.80 (m, 7H, 7H, Ph-H), 8.36 (d, *J* = 8.5 Hz, 1H, CO-NH), 9.36 (s, 1H, SO_2_-NH); ^13^C NMR (DMSO-*d*_*6*_) *δ*: 21.32, 24.36, 24.45, 27.08, 32.95, 48.53, 65.11, 122.03, 123.84, 126.78, 126.97, 127.69, 128.91, 128.98, 131.28, 131.33, 132.37, 133.58, 134.25, 141.24, 165.99; IR (*ν*, cm^−1^): 3346, 3045, 1630; MS (z/e): 475[M + H]^+^, 280, 216, 119, 91; Anal. Calcd for C_21_H_22_ClF_3_N_2_O_3_S: C, 53.11; H, 4.67; N, 5.90; found: C, 53.31; H, 4.88; N, 6.12.

### *N*-(2-trifluoromethyl-4-chlorophenyl)-2-(2,4-dimethylbenzoylamino) cyclohexylsulfonamide (IV-6)

(R^2^ = 2,4-(CH_3_)_2_C_6_H_3_) White solid; yield, 95%; mp 179–181 °C; ^1^H NMR (DMSO*-d*_*6*_) *δ*: 1.35–2.01 (m, 8H, 4CH_2_), 2.30 (d, *J* = 5.4 Hz, 6H, CH_3_ + CH_3_), 3.50 (dt, *J* = 11.6, 3.6 Hz, 1H, CH-N), 4.78 (dd, *J* = 8.8, 3.1 Hz, 1H, CH-SO_2_), 7.03–7.81 (m, 6H, Ph-H), 8.27 (d, *J* = 9.1 Hz, 1H, CO-NH), 9.37 (s, 1H, SO_2_-NH); ^13^C NMR (DMSO-*d*_*6*_) *δ*: 19.41, 19.59, 21.16, 21.36, 24.37, 31.13, 44.46, 63.39, 122.08, 123.89, 126.02, 126.96, 128.20, 130.59, 130.97, 131.14, 133.62, 134.18, 134.40, 136.01, 139.11, 170.00; IR (*ν*, cm^−1^): 3392, 3053, 1645; MS (z/e): 489[M + H]^+^, 294, 133, 105, 79; Anal. Calcd for C_22_H_24_ClF_3_N_2_O_3_S: C, 54.04; H, 4.95; N, 5.73; found: C, 53.89; H, 5.08; N, 5.54.

### *N*-(2-trifluoromethyl-4-chlorophenyl)-2-(3,5-dimethylbenzoylamino) cyclohexylsulfonamide (IV-7)

(R^2^ = 3,5-(CH_3_)_2_C_6_H_3_) White solid; yield, 93%; mp 193–195 °C; ^1^H NMR (DMSO*-d*_*6*_) *δ*: 1.33–2.31 (m, 8H, 4CH_2_), 2.28 (s, 6H, CH_3_ + CH_3_), 3.41 (td, *J* = 11.0, 2.8 Hz, 1H, CH-N), 4.23 (ddd, *J* = 19.2, 10.5, 4.0 Hz, 1H, CH-SO_2_), 7.12–7.79 (m, 6H, 6H, Ph-H), 8.35 (d, *J* = 8.5 Hz, 1H, CO-NH), 9.33 (s, 1H, SO_2_-NH); ^13^C NMR (DMSO-*d*_*6*_) *δ*: 21.21, 24.34, 24.45, 27.04, 32.87, 48.47, 63.01, 65.13, 122.04, 123.85, 125.37, 125.82, 126.99, 131.07, 131.16, 132.65, 133.58, 134.28, 135.17, 137.46, 137.52, 166.38; IR (*ν*, cm^−1^): 3324, 2977, 1626; MS (z/e): 489[M + H]^+^, 294, 230, 133, 105; Anal. Calcd for C_22_H_24_ClF_3_N_2_O_3_S: C, 54.04; H, 4.95; N, 5.73; found: C, 54.27; H, 4.77; N, 5.93.

### *N*-(2-trifluoromethyl-4-chlorophenyl)-2-(2-fluorobenzoylamino) cyclohexylsulfonamide (IV-8)

(R^2^ = 2-FC_6_H_4_) White solid; yield, 96%; mp 167–169 °C; ^1^H NMR (DMSO*-d*_*6*_) *δ*: 1.38–2.11 (m, 8H, 4CH_2_), 3.54 (m, 1H, CH-N), 4.76 (s, 1H, CH-SO_2_), 7.26–7.83 (m, 7H, Ph-H), 8.29 (dd, *J* = 7.2, 65.3 Hz, 1H, CO-NH), 9.41 (s, 1H, SO_2_-NH); ^13^C NMR (DMSO-*d*_*6*_) *δ*: 19.65, 21.76, 24.12, 30.55, 45.14, 62.95, 116.41, 122.04, 123.86, 124.63, 127.02, 130.71, 130.98, 131.28, 132.74, 133.63, 133.97, 158.90, 160.55, 164.18; IR (*ν*, cm^−1^): 3308, 3077, 1635; MS (z/e): 479[M + H]^+^, 284, 220, 123, 95; Anal. Calcd for C_20_H_19_ClF_4_N_2_O_3_S: C, 50.16; H, 4.00; N, 5.85; found: C, 50.38; H, 4.14; N, 5.77.

### *N*-(2-trifluoromethyl-4-chlorophenyl)-2-(3-fluorobenzoylamino) cyclohexylsulfonamide (IV-9)

(R^2^ = 3-FC_6_H_4_) White solid; yield, 94%; mp 192–194 °C; ^1^H NMR (DMSO*-d*_*6*_) *δ*: 1.31–2.20 (m, 8H, 4CH_2_), 3.52 (d, *J* = 11.8 Hz, 1H, CH-N), 4.76 (s, 1 H, CH-SO_2_), 7.38–7.80 (m, 7H, Ph-H), 8.21 (d, *J* = 8.5 Hz, 1H, CO-NH), 9.41 (s, 1H, SO_2_-NH); ^13^C NMR (DMSO-*d*_*6*_) *δ*: 19.65, 21.65, 24.13, 30.61, 45.33, 62.96, 115.08, 118.36, 122.04, 123.85, 124.39, 127.09, 130.54, 130.60, 131.19, 131.36, 133.62, 133.96, 137.66, 166.04; IR (*ν*, cm^−1^): 3378, 3050, 1630; MS (z/e): 479[M + H]^+^, 284, 220, 123, 95; Anal. Calcd for C_20_H_19_ClF_4_N_2_O_3_S: C, 50.16; H, 4.00; N, 5.85; found: C, 50.31; H, 3.83; N, 6.02.

### *N*-(2-trifluoromethyl-4-chlorophenyl)-2-(2-chlorobenzoylamino) cyclohexylsulfonamide (IV-10)

(R^2^ = 2-ClC_6_H_4_) White solid; yield, 97%; mp 199–201 °C; ^1^H NMR (DMSO*-d*_*6*_) *δ*: 1.33–2.20 (m, 8H, 4CH_2_), 3.52 (m, 1H, CH-N), 4.78 (dd, *J* = 3.0, 8.9 Hz, 1H, CH-SO_2_), 7.46–7.81 (m, 7H, Ph-H), 8.58 (d, *J* = 9.2 Hz, 1H, CO-NH), 9.37 (s, 1H, SO_2_-NH); ^13^C NMR (DMSO-*d*_*6*_) *δ*: 19.43, 21.41, 24.31, 30.94, 44.55, 63.14, 122.06, 123.88, 127.02, 127.20, 129.59, 129.74, 130.53, 130.92, 130.99, 131.18, 133.64, 134.07, 137.17, 166.65; IR (*ν*, cm^−1^): 3386, 3083, 1655; MS (z/e): 495[M + H]^+^, 300, 236, 139, 111; Anal. Calcd for C_20_H_19_Cl_2_F_3_N_2_O_3_S: C, 48.49; H, 3.87; N, 5.66; found: C, 48.62; H, 4.01; N, 5.39.

### *N*-(2-trifluoromethyl-4-chlorophenyl)-2-(3-chlorobenzoylamino) cyclohexylsulfonamide (IV-11)

(R^2^ = 3-ClC_6_H_4_) White solid; yield, 95%; mp 184–186 °C; ^1^H NMR (DMSO*-d*_*6*_) *δ*: 1.33–2.07 (m, 8H, 4CH_2_), 3.51–3.53 (m, 1H, CH-N), 4.76 (s, 1H, CH-SO_2_), 7.46–7.81 (m, 7H, Ph-H), 8.58 (d, *J* = 8.6 Hz, 1H, CO-NH), 9.37 (s, 1H, SO_2_-NH); ^13^C NMR (DMSO-*d*_*6*_) *δ*: 19.66, 21.65, 24.13, 30.62, 45.32, 62.96, 122.04, 123.86, 126.98, 127.08, 128.02, 130.41, 131.12, 131.25, 131.33, 133.15, 133.62, 133.97, 137.34, 166.04; IR (*ν*, cm^−1^): 3353, 3069, 1638; MS (z/e): 495[M + H]^+^, 300, 236, 139, 111; Anal. Calcd for C_20_H_19_Cl_2_F_3_N_2_O_3_S: C, 48.49; H, 3.87; N, 5.66; found: C, 48.70; H, 3.66; N, 5.87.

### *N*-(2-trifluoromethyl-4-chlorophenyl)-2-(4-chlorobenzoylamino) cyclohexylsulfonamide (IV-12)

(R^2^ = 4-ClC_6_H_4_) White solid; yield, 67%; mp 188–190 °C; ^1^H NMR (DMSO*-d*_*6*_) *δ*: 1.29–2.17 (m, 8H, 4CH_2_), 3.51 (dt, *J* = 11.9, 3.5 Hz, 1H, CH-N), 4.74 (dd, *J* = 8.1, 3.6 Hz, 1H, CH-SO_2_), 7.50–7.86 (m, 7H, Ph-H), 8.19 (d, *J* = 8.7 Hz, 1H, CO-NH), 9.40 (s, 1H, SO_2_-NH); ^13^C NMR (DMSO-*d*_*6*_) *δ*: 19.67, 21.66, 24.13, 30.62, 45.32, 62.99, 122.04, 123.86, 126.61, 126.81, 127.04, 128.45, 130.12, 131.09, 131.31, 133.62, 133.99, 134.06, 136.25, 166.41; IR (*ν*, cm^−1^): 3386, 3081, 1634; MS (z/e): 495[M + H]^+^, 300, 236, 139, 111; Anal. Calcd for C_20_H_19_Cl_2_F_3_N_2_O_3_S: C, 48.49; H, 3.87; N, 5.66; found: C, 48.33; H, 4.10; N, 5.81.

### *N*-(2-trifluoromethyl-4-chlorophenyl)-2-(2,6-dichlorobenzoylamino) cyclohexylsulfonamide (IV-13)

(R^2^ = 2,6-Cl_2_C_6_H_3_) White solid; yield, 90%; mp 216–218 °C; ^1^H NMR (DMSO*-d*_*6*_) *δ*: 1.34–2.19 (m, 8H, 4CH_2_), 3.35–3.38 (m, 1H, CH-N), 4.45–4.49 (m, 1H, CH-SO_2_), 7.41–7.81 (m, 6H, Ph-H), 8.86 (d, *J* = 7.8 Hz, 1H, CO-NH), 9.46 (s, 1H, SO_2_-NH); ^13^C NMR (DMSO-*d*_*6*_) *δ*: 21.81, 22.31, 24.27, 29.38, 46.63, 62.44, 121.98, 123.79, 127.17, 127.73, 128.18, 128.40, 131.33, 131.69, 131.80, 131.85, 133.63, 134.03, 136.63, 163.20; IR (*ν*, cm^−1^): 3309, 3110, 1645; HRMS-ESI, m/z calcd for C_20_H_19_Cl_3_F_3_N_2_O_3_S [M + H]^+^529.0134; found, 529.0128.

### *N*-(2-trifluoromethyl-4-chlorophenyl)-2-(3,5-dichlorobenzoylamino) cyclohexylsulfonamide (IV-14)

(R^2^ = 3,5-Cl_2_C_6_H_3_) White solid; yield, 98%; mp 236–238 °C; ^1^H NMR (DMSO*-d*_*6*_) *δ*: 1.33–2.38 (m, 8H, 4CH_2_), 3.39 (td, *J* = 11.2, 3.0 Hz, 1H, CH-N), 4.21 (ddd, *J* = 19.3, 10.7, 4.0 Hz, 1H, CH-SO_2_), 7.56–7.80 (m, 6H, Ph-H), 8.70 (d, *J* = 8.5 Hz, 1H, CO-NH), 9.39 (s, 1H, SO_2_-NH); ^13^C NMR (DMSO-*d*_*6*_) *δ*: 24.30, 24.38, 27.03, 32.83, 48.76, 64.92, 120.19, 122.01, 123.83, 126.45, 126.95, 127.03, 127.15, 130.84, 131.50, 133.60, 134.13, 134.51, 138.39, 163.14; IR (*ν*, cm^−1^): 3268, 3086, 1644; MS (z/e): 529[M + H]^+^, 334, 270, 173, 145; Anal. Calcd for C_20_H_18_Cl_3_F_3_N_2_O_3_S: C, 45.34; H, 3.42; N, 5.29; found: C, 45.27; H, 3.67; N, 5.04.

### *N*-(2-trifluoromethyl-4-chlorophenyl)-2-(2-trifluoromethylbenzoylamino) cyclohexylsulfonamide (IV-15)

(R^2^ = 2-CF_3_C_6_H_4_) Colorless crystal; yield, 90%; mp 88–89 °C; ^1^H NMR (DMSO*-d*_*6*_) *δ*: 1.34–2.03 (m, 8H, 4CH_2_), 3.49–3.51 (m, 1H, CH-N), 4.80 (s, 1H, CH-SO_2_), 7.56–7.81 (m, 7H, Ph-H), 8.67 (d, *J* = 5.5 Hz, 1H, CO-NH), 9.33 (s, 1H, SO_2_-NH); ^13^C NMR (DMSO-*d*_*6*_) *δ*: 19.25, 21.37, 24.33, 30.78, 44.42, 63.10, 122.06, 123.29, 123.87, 125.11, 126.31, 127.06, 129.18, 129.86, 131.21, 131.33, 132.52, 133.63, 134.03, 136.78, 167.22; IR (*ν*, cm^−1^): 3338, 3195, 1657; MS (z/e): 529[M + H]^+^, 334, 270, 173, 145; Anal. Calcd for C_21_H_19_ClF_6_N_2_O_3_S: C, 47.69; H, 3.62; N, 5.30; found: C, 47.89; H, 3.52; N, 5.21.

### *N*-(2-trifluoromethyl-4-chlorophenyl)-2-(3-trifluoromethylbenzoylamino) cyclohexylsulfonamide (IV-16)

(R^2^ = 3-CF_3_C_6_H_4_) White solid; yield, 89%; mp 178–179 °C; ^1^H NMR (DMSO*-d*_*6*_) *δ*: 1.42–2.19 (m, 8H, 4CH_2_), 3.52 (d, *J* = 11.3 Hz, 1H, CH-N), 4.77 (s, 1H, CH-SO_2_), 7.60–8.11 (m, 7H, Ph-H), 8.41 (d, *J* = 8.7 Hz, 1H, CO-NH), 9.41 (s, 1H, SO_2_-NH); ^13^C NMR (DMSO-*d*_*6*_) *δ*: 19.75, 21.74, 24.06, 30.57, 45.42, 62.88, 122.04, 123.51, 123.85, 124.86, 125.31, 127.06, 128.03, 129.05, 129.26, 129.69, 131.13, 132.32, 133.63, 136.29, 166.09; IR (*ν*, cm^−1^): 3381, 3092, 1636; MS (z/e): 529[M + H]^+^, 334, 270, 173, 145; Anal. Calcd for C_21_H_19_ClF_6_N_2_O_3_S: C, 47.69; H, 3.62; N, 5.30; found: C, 47.55; H, 3.76; N, 5.13.

### *N*-(2-trifluoromethyl-4-chlorophenyl)-2-(2-methoxy-5-chlorobenzoylamino) cyclohexylsulfonamide (IV-17)

(R^2^ = 2-CH_3_O-5-ClC_6_H_3_) White solid; yield, 97%; mp 140–141 °C; ^1^H NMR (DMSO*-d*_*6*_) *δ*: 1.44–2.15 (m, 8H, 4CH_2_), 3.53–3.56 (m, 1H, CH-N), 3.93 (s, 3H, OCH_3_), 4.63 (dd, *J* = 7.0, 3.4 Hz, 1H, CH-SO_2_), 7.22–7.80 (m, 6H, Ph-H), 8.58 (d, *J* = 7.4 Hz, 1H, CO-NH), 9.54 (s, 1H, SO_2_-NH); ^13^C NMR (DMSO-*d*_*6*_) *δ*: 20.01, 22.44, 23.96, 29.84, 45.62, 57.02, 62.71, 114.86, 122.01, 123.83, 124.09, 124.98, 127.08, 130.23, 131.29, 131.53, 132.40, 133.64, 133.79, 156.49, 163.29; IR (*ν*, cm^−1^): 3380, 3125, 1653; MS (z/e): 525[M + H]^+^, 266, 169, 126, 111; Anal. Calcd for C_21_H_21_Cl_2_F_3_N_2_O_4_S: C, 48.01; H, 4.03; N, 5.33; found: C, 48.26; H, 3.90; N, 5.62.

### *N*-(2-trifluoromethyl-4-chlorophenyl)-2-(acetylamino) cyclohexylsulfonamide (IV-18)

(R^2^ = Me) Colorless crystal; yield, 99%; mp 145–146 °C; ^1^H NMR (DMSO*-d*_*6*_) *δ*: 1.34–2.03 (m, 8H, 4CH_2_), 1.87 (s, 3H, CH_3_), 3.39 (dt, *J* = 12.1, 3.2 Hz, 1H, CH-N), 4.55 (dd, *J* = 8.8, 2.9 Hz, 1H, CH-SO_2_), 7.60–7.78 (m, 3H, Ph-H), 7.95 (d, *J* = 9.2 Hz, 1H, CO-NH), 9.26 (s, 1H, SO_2_-NH); ^13^C NMR (DMSO-*d*_*6*_) *δ*: 19.49, 21.40, 23.08, 24.24, 30.87, 44.07, 63.19, 122.06, 123.87, 126.91, 129.99, 130.72, 133.59, 134.22, 170.44; IR (*ν*, cm^−1^): 3390, 3037, 1657; MS (z/e): 398[M]^+^, 194, 159, 140; Anal. Calcd for C_15_H_18_ClF_3_N_2_O_3_S: C, 45.17; H, 4.55; N, 7.02; found: C, 44.95; H, 4.21; N, 7.26.

### *N*-(2-trifluoromethyl-4-chlorophenyl)-2-(propionylamino) cyclohexylsulfonamide (IV-19)

(R^2^ = Et) Colorless crystal; yield, 98%; mp 165–167 °C; ^1^H NMR (DMSO*-d*_*6*_) *δ*: 0.98 (t, *J* = 7.6 Hz, 3H, CH_3_), 1.29–2.00 (m, 8H, 4CH_2_), 2.15 (q, *J* = 7.6 Hz, 2H, CH_2_), 3.39 (dt, *J* = 12.0, 3.3 Hz, 1H, CH-N), 4.56 (dd, *J* = 8.8, 3.1 Hz, 1H, CH-SO_2_), 7.59–7.79 (m, 3H, Ph-H), 7.84 (d, *J* = 9.2 Hz, 1H, CO-NH), 9.25 (s, 1H, SO_2_-NH); ^13^C NMR (DMSO-*d*_*6*_) *δ*: 10.19, 19.52, 21.45, 24.24, 28.76, 30.87, 43.94, 63.27, 122.05, 123.87, 126.94, 130.24, 130.80, 133.56, 134.19, 174.04; IR (*ν*, cm^−1^): 3375, 3095, 1643; MS (z/e): 412[M]^+^, 218, 154, 69, 57; Anal. Calcd for C_16_H_20_ClF_3_N_2_O_3_S: C, 46.55; H, 4.88; N, 6.79; found: C, 46.27; H, 5.01; N, 6.58.

### *N*-(2-trifluoromethyl-4-chlorophenyl)-2-(n-butyrylamino) cyclohexylsulfonamide (IV-20)

(R^2^ = *n*-propyl) Colorless crystal; yield, 93%; mp 119–121 °C; ^1^H NMR (DMSO*-d*_*6*_) *δ*: 0.86 (t, *J* = 4.8 Hz, 3H, CH_3_), 1.32–2.14 (m, 12H, 6CH_2_), 3.35–3.40 (m, 1H, CH-N), 4.56 (d, *J* = 4.0 Hz, 1H, CH-SO_2_), 7.59–7.80 (m, 3H, Ph-H), 7.87 (d, *J* = 6.4 Hz, 1H, CO-NH), 9.29 (s, 1H, SO_2_-NH); ^13^C NMR (DMSO-*d*_*6*_) *δ*: 13.98, 19.10, 19.49, 21.46, 24.26, 30.98, 37.56, 43.96, 63.26, 122.06, 123.87, 126.98, 130.35, 130.85, 133.59, 134.19, 173.11; IR (*ν*, cm^−1^): 3392, 3101, 1658; HRMS-ESI, m/z calcd for C_17_H_23_ClF_3_N_2_O_3_S [M + H]^+^427.1070; found, 427.1076.

### *N*-(2-trifluoromethyl-4-chlorophenyl)-2-(n-valerylamino) cyclohexylsulfonamide (IV-21)

(R^2^ = *n*-butyl) White crystal; yield, 92%; mp 175–177 °C; ^1^H NMR (DMSO*-d*_*6*_) *δ*: 0.84 (t, *J* = 6.9 Hz, 3H, CH_3_), 1.23–2.24 (m, 14H, 7CH_2_), 3.20 (td, *J* = 10.1, 3.4 Hz, 1H, CH-N), 4.06 (ddd, *J* = 18.7, 9.3, 4.0 Hz, 1H, CH-SO_2_), 7.57–7.80 (m, 3H, Ph-H), 7.93 (d, *J* = 8.5 Hz, 1H, CO-NH), 9.31 (s, 1H, SO_2_-NH); ^13^C NMR (DMSO-*d*_*6*_) *δ*: 14.26, 22.34, 23.81, 25.31, 26.31, 28.64, 31.41, 35.89, 47.42, 65.02, 122.02, 123.83, 126.92, 130.97, 131.09, 133.55, 134.25, 172.43; IR (*ν*, cm^−1^): 3360, 1647; MS (z/e): 440[M]^+^, 246, 195, 57; Anal. Calcd for C_18_H_24_ClF_3_N_2_O_3_S: C, 49.03; H, 5.49; N, 6.35; found: C, 49.25; H, 5.30; N, 6.28.

### *N*-(2-trifluoromethyl-4-chlorophenyl)-2-(n-hexanoylamino) cyclohexylsulfonamide (IV-22)

(R^2^ = *n*-pentyl) White crystal; yield, 87%; mp 119–121 °C; ^1^H NMR (DMSO*-d*_*6*_) *δ*: 0.86 (t, *J* = 7.3 Hz, 3H, CH_3_), 1.21–2.19 (m, 16H, 8CH_2_), 3.40 (d, *J* = 11.9 Hz, 1H, CH-N), 4.57 (d, *J* = 5.4 Hz, 1H, CH-SO_2_), 7.60–7.86 (m, 3H, Ph-H), 7.79 (s, 1H, CO-NH), 9.26 (s, 1H, SO_2_-NH); ^13^C NMR (DMSO-*d*_*6*_) *δ*: 14.28, 22.34, 23.91, 25.31, 26.31, 28.64, 31.41, 32.30, 35.88, 47.41, 64.99, 109.90, 122.02, 123.84, 126.99, 131.01, 133.57, 134.24, 172.41; IR (*ν*, cm^−1^): 3376, 3029, 1646; HRMS-ESI, m/z calcd for C_19_H_27_ClF_3_N_2_O_3_S [M + H]^+^455.1383; found, 455.1389.

### *N*-(2-trifluoromethyl-4-chlorophenyl)-2-(n-heptanoylamino) cyclohexylsulfonamide (IV-23)

(R^2^ = *n*-hexyl) White crystal; yield, 95%; mp 105–106 °C; ^1^H NMR (DMSO*-d*_*6*_) *δ*: 0.84 (t, *J* = 7.4 Hz, 3H, CH_3_), 1.20–2.24 (m, 18H, 9CH_2_), 3.20 (td, *J* = 10.1, 3.4 Hz, 1H, CH-N), 4.06 (ddd, *J* = 18.7, 9.4, 4.0 Hz, 1H, CH-SO_2_), 7.57–7.80 (m, 3H, Ph-H), 7.93 (d, *J* = 8.5 Hz, 1H, CO-NH), 9.32 (s, 1H, SO_2_-NH); ^13^C NMR (DMSO-*d*_*6*_) *δ*: 14.11, 22.14, 23.82, 23.93, 26.34, 27.51, 32.33, 35.61, 40.44, 47.42, 65.03, 122.03, 123.84, 126.98, 130.99, 131.09, 133.57, 134.25, 172.41; IR (*ν*, cm^−1^): 3325, 3110, 1643; MS (z/e): 468[M]^+^, 274, 195, 113; Anal. Calcd for C_20_H_28_ClF_3_N_2_O_3_S: C, 51.22; H, 6.02; N, 5.97; found: C, 51.42; H, 5.90; N, 6.15.

### *N*-(2-trifluoromethyl-4-chlorophenyl)-2-(2-chloroacetylamino) cyclohexylsulfonamide (IV-24)

(R^2^ = ClCH_2_) Gray solid; yield, 94%; mp 121–124 °C; ^1^H NMR (DMSO*-d*_*6*_) *δ*: 1.28–2.03 (m, 8H, 4CH_2_), 3.42 (dt, *J* = 12.1, 3.4 Hz, 1H, CH-N), 4.10 (dd, *J* = 53.0, 12.9 Hz, 2H, CH_2_), 4.52 (dd, *J* = 8.3, 3.3 Hz, 1H, CH-SO_2_), 7.59–7.80 (m, 3H, Ph-H), 8.19 (d, *J* = 8.8 Hz, 1H, CO-NH), 9.30 (s, 1H, SO_2_-NH); ^13^C NMR (DMSO-*d*_*6*_) *δ*: 19.63, 21.70, 24.05, 30.49, 43.00, 44.89, 62.73, 122.02, 123.84, 127.04, 131.06, 131.33, 133.64, 133.92, 166.10; IR (*ν*, cm^−1^): 3383, 3180, 1678; HRMS-ESI, m/z calcd for C_15_H_18_Cl_2_F_3_N_2_O_3_S [M + H]^+^433.0367; found, 433.0371.

### *N*-(2-trifluoromethyl-4-chlorophenyl)-2-(2,2-dichloroacetylamino) cyclohexylsulfonamide (IV-25)

(R^2^ = Cl_2_CH) Colorless crystal; yield, 82%; mp 179–180 °C; ^1^H NMR (DMSO*-d*_*6*_) *δ*: 1.44–1.95 (m, 8H, 4 CH_2_), 3.44 (dt, *J* = 3.3, 12.2 Hz, 1H, CH-N), 4.49 (dd, *J* = 8.2, 3.5 Hz, 1H, CH-SO_2_),6.54 (s, 1H, CH-Cl_2_), 7.50–7.81 (m, 3H, Ph-H), 8.47 (d, *J* = 8.6 Hz, 1H, CO-NH), 9.41 (s, 1H, SO_2_-NH); ^13^C NMR (DMSO-*d*_*6*_) δ: 14.47, 19.61, 23.98, 30.25, 45.35, 62.37, 66.82, 122.01, 123.83, 127.12, 131.58, 133.65, 133.80, 162.80, 163.24; IR (*ν*, cm^−1^): 3367, 3273, 1672; MS (z/e): 468(M^+^), 274, 210, 130, 81, 64; Anal. Calcd for C_15_H_16_ClF_3_N_2_O_3_S: C, 38.52; H, 3.45; N, 5.99; found: C, 38.66; H, 3.59; N, 5.73.

### *N*-(2-trifluoromethyl-4-chlorophenyl)-2-(2,2,2-chloroacetylamino) cyclohexylsulfonamide (IV-26)

(R^2^ = Cl_3_C) Colorless crystal; yield, 88%; mp 151–154 °C; ^1^H NMR (DMSO*-d*_*6*_) *δ*: 1.28–2.37 (m, 8H, 4CH_2_), 3.51–3.58 (m, 1H, CH-N), 3.95–4.04 (m, 1H, CH-SO_2_), 7.55–7.81 (m, 3H, Ph-H), 8.85 (d, *J* = 8.4 Hz, 1H, CO-NH), 9.46 (s, 1H, SO_2_-NH); ^13^C NMR (DMSO-*d*_*6*_) *δ*: 24.27, 27.11, 28.68, 31.88, 50.33, 63.74, 93.26, 121.99, 123.81, 127.05, 131.72, 132.05, 133.64, 134.09, 160.27; IR (*ν*, cm^−1^): 3421, 3311, 1708; MS (z/e): 306, 242, 161; Anal. Calcd for C_15_H_15_Cl_4_F_3_N_2_O_3_S: C, 35.88; H, 3.01; N, 5.58; found: C, 36.01; H, 3.22; N, 5.47.

### *N*-(2-trifluoromethyl-4-chlorophenyl)-2-(2-bromoacetylamino) cyclohexylsulfonamide (IV-27)

(R^2^ = BrCH_2_) Colorless crystal; yield, 90%; mp 131–132 °C; ^1^H NMR (DMSO*-d*_*6*_) *δ*: 1.33–2.08 (m, 8H, 4CH_2_), 3.41 (m, 1H, CH-N), 3.88–4.16 (m, 2H, CH_2_-Br), 4.52 (s, 1H, CH-SO_2_), 7.59–7.81 (m, 3H, Ph-H), 8.33 (dd, *J* = 9.0, 61.8 Hz, 1H, CO-NH), 9.32 (d, *J* = 18.6 Hz, 1H, SO_2_-NH); ^13^C NMR (DMSO-*d*_*6*_) *δ*: 19.63, 21.70, 24.06, 30.50, 43.00, 44.89, 62.74, 122.02, 123.84, 127.06, 131.05, 131.32, 133.62, 133.92, 166.11; IR (*ν*, cm^−1^): 3383, 3090, 1678; MS (z/e): 477[M + H]^+^, 80; Anal. Calcd for C_15_H_17_BrClF_3_N_2_O_3_S: C, 37.71; H, 3.59; N, 5.86; found: C, 37.94; H, 3.47; N, 5.62.

### *N*-(2-trifluoromethyl-4-chlorophenyl)-2-(2-methoxyacetylamino) cyclohexylsulfonamide (IV-28)

(R^2^ = CH_3_OCH_2_) White solid; yield, 79%; mp 125–127 °C; ^1^H NMR (DMSO*-d*_*6*_) *δ*: 1.38–2.10 (m, 8 H, 4CH_2_), 3.31 (s, 3H, OCH_3_), 3.47 (dt, *J* = 11.5, 3.3 Hz, 1H, CH-N), 3.79–3.86 (m, 2H, OCH_2_), 4.51 (dd, *J* = 7.4, 3.5 Hz, 1H, CH-SO_2_), 7.55 (d, *J* = 8.0 Hz, 1H, CO-NH), 7.60–7.81 (m, 3H, Ph-H), 9.42 (s, 1H, SO_2_-NH); ^13^C NMR (DMSO-*d*_*6*_) *δ*: 19.80, 21.98, 23.87, 30.17, 44.51, 58.98, 62.62, 71.52, 122.02, 123.83, 127.10, 127.15, 131.05, 131.36, 133.66, 169.47; IR (*ν*, cm^−1^): 3394, 3099, 1647; MS (z/e): 428[M]^+^, 234, 195, 170; Anal. Calcd for C_16_H_20_ClF_3_N_2_O_4_S: C, 44.81; H, 4.70; N, 6.53; found: C, 45.03; H, 4.52; N, 6.77.

### *N*-(2-trifluoromethyl-4-chlorophenyl)-2-(2-ethoxyacetylamino) cyclohexylsulfonamide (IV-29)

(R^2^ = C_2_H_5_OCH_2_) Colorless crystal; yield, 32%; mp 125–127 °C; ^1^H NMR (DMSO*-d*_*6*_) *δ*: 1.14 (t, *J* = 7.0 Hz, 3H, CH_3_) 1.30–2.07 (m, 8H, 4CH_2_), 3.46–3.51 (m, 3H, OCH_2_-CO, CH-N), 3.86 (q, *J* = 15.3 Hz, 2H, OCH_2_), 4.47 (dd, *J* = 7.3, 3.6 Hz, 1H, CH-SO_2_), 7.53 (d, *J* = 7.8 Hz, 1H, CO-NH), 7.60–7.81 (m, 3H, Ph-H), 9.48 (s, 1H, SO_2_-NH); ^13^C NMR (DMSO-*d*_*6*_) *δ*: 15.32, 19.88, 22.11, 23.82, 29.99, 44.66, 62.51, 66.62, 69.67, 109.90, 115.73, 122.01, 123.82, 127.07, 131.18, 133.66, 169.71; IR (*ν*, cm^−1^): 3412, 3070, 1681; HRMS-ESI, m/z calcd for C_17_H_23_ClF_3_N_2_O_4_S [M + H]^+^443.1019; found, 443.1024.

### *N*-(2-trifluoromethyl-4-chlorophenyl)-2-(2,2,2-trichloroacetylamino) cyclopentylsulfonamide (IV-30)

(n = 0, R^1^ = H) Colorless crystal; yield, 98%; mp 86–88 °C; ^1^H NMR (DMSO*-d*_*6*_) *δ*: 1.63–2.28 (m, 6H, 3CH_2_), 3.94 (q, *J* = 7.5 Hz, 1H, CH-N), 4.40 (p, *J* = 7.1 Hz, 1H, CH-SO_2_), 7.59–7.82 (m, 3H, Ph-H), 8.65 (d, *J* = 6.9 Hz, 1H, CO-NH), 9.72 (s, 1H, SO_2_-NH); ^13^C NMR (DMSO-*d*_*6*_) *δ*: 21.44, 26.54, 30.73, 53.99, 63.48, 92.81, 121.93, 123.75, 127.25, 131.84, 132.01, 133.56, 133.73, 161.25; IR (*ν*, cm^−1^): 3363, 3190, 1726; MS (z/e): 488(M)^+^, 294, 230, 164, 67; Anal. Calcd for C_14_H_13_Cl_4_F_3_N_2_O_3_S: C, 34.45; H, 2.68; N, 5.74; found: C, 34.56; H, 2.87; N, 5.49.

### *N*-(2-trifluoromethyl-4-chlorophenyl)-2-(2,2,2-trichloroacetylamino) cycloheptylsulfonamide (IV-31)

(n = 2, R^1^ = H) Colorless crystal; yield, 97%; mp 114–115 °C; ^1^H NMR (DMSO*-d*_*6*_) *δ*: 1.17–2.17 (m, 10H, 5CH_2_), 3.57–3.59 (m, 1H, CH-N), 4.57 (s, 1H, CH-SO_2_), 7.60–7.78 (m, 3H, Ph-H), 8.34 (d, *J* = 6.5 Hz, 1H, CO-NH), 9.58 (s, 1H, SO_2_-NH); ^13^H NMR (DMSO-*d*_*6*_) *δ*: 8.96, 22.60, 23.79, 25.95, 27.86, 31.06, 46.04, 50.26, 65.05, 93.06, 122.09, 123.90, 125.72, 127.12, 130.96, 133.59, 160.72; IR (*ν*, cm^−1^): 3381, 3242, 1710; MS (z/e): 530(M)^+^, 320, 256, 162, 95, 67; Anal. Calcd for C_16_H_17_Cl_4_F_3_N_2_O_3_S: C, 37.23; H, 3.32; N, 5.43; found: C, 37.55; H, 3.21; N, 5.62.

### *N*-(2-trifluoromethyl-4-chlorophenyl)-3-methyl-2-(2,2,2-trichloroacetylamino) cyclohexylsulfonamide (IV-32)

(n = 1, R^1^ = 3-Me) Colorless crystal; yield, 96%; mp 137~139 °C; ^1^H NMR (DMSO*-d*_*6*_) *δ*: 0.93–2.35 (m, 10H, 5CH_2_), 3.75 (dd, *J* = 9.4, 4.6 Hz, 1H, CH-N), 3.97 (td, *J* = 8.6, 4.2 Hz, 1H, CH-SO_2_), 7.54–7.81 (m, 3H, Ph-H), 8.55 (d, *J* = 8.2 Hz, 1H, CO-NH), 9.66 (s, 1H, SO_2_-NH); ^13^C NMR (DMSO-*d*_*6*_) *δ*: 18.24, 19.65, 24.93, 31.96, 50.48, 55.50, 60.87, 92.94, 121.97, 123.79, 127.19, 130.90, 131.59, 133.66, 133.73, 161.15; IR (*ν*, cm^−1^): 3398, 3250, 1707; MS (z/e): 516(M)^+^, 322, 258, 164, 95, 67; Anal. Calcd for C_16_H_17_Cl_4_F_3_N_2_O_3_S: C, 37.23; H, 3.32; N, 5.43; found: C, 37.42; H, 3.12; N, 5.60.

### *N*-(2-trifluoromethyl-4-chlorophenyl)-4-methyl-2-(2,2,2-trichloroacetylamino) cyclohexylsulfonamide (IV-33)

(n = 1, R^1^ = 4-Me) Colorless crystal; yield, 93%; mp 123–124 °C; ^1^H NMR (DMSO*-d*_*6*_) *δ*: 0.93 (d, *J* = 6.0 Hz, 3H, CH_3_), 1.40–2.40 (m, 7H, C_4_H_7_), 3.82 (s, 1H, CH-N), 4.10–4.20 (m, 1H, CH-SO_2_), 7.53–7.82 (m, 3H, Ph-H), 8.81 (d, *J* = 7.2 Hz, 1H, CO-NH), 9.85 (s, 1H, SO_2_-NH); ^13^C NMR (DMSO-*d*_*6*_) *δ*: 22.18, 25.42, 28.17, 31.15, 34.89, 51.80, 59.87, 92.66, 121.92, 123.74, 127.29, 131.20, 131.89, 133.53, 133.80, 160.81; IR (*ν*, cm^−1^): 3360, 3226, 1693; MS (z/e): 516(M)^+^, 259, 224, 202, 112, 81; Anal. Calcd for C_16_H_17_Cl_4_F_3_N_2_O_3_S: C, 37.23; H, 3.32; N, 5.43; found: C,36.99; H, 3.21; N, 5.60.

### *N*-(2-trifluoromethyl-4-chlorophenyl)-5-methyl-2-(2,2,2-trichloroacetylamino) cyclohexylsulfonamide (IV-34)

(n = 1, R^1^ = 5-Me) White crystal; yield, 98%; mp 144–145 °C; ^1^H NMR (DMSO*-d*_*6*_) *δ*: 0.95–2.33 (m, 10H, 5CH_2_), 3.64 (td, *J* = 11.6, 2.9 Hz, 1H, CH-N), 3.90–4.03 (m, 1H, CH-SO_2_), 7.55–7.81 (m, 3H, Ph-H), 8.83 (d, *J* = 8.4 Hz, 1H, CO-NH), 9.45 (s, 1H, SO_2_-NH); ^13^C NMR (DMSO-*d*_*6*_) *δ*: 22.11, 31.03, 31.71, 32.72, 35.13, 50.22, 63.45, 93.26, 122.00, 123.81, 127.07, 131.67, 132.05, 133.61, 134.11, 160.32; IR (*ν*, cm^−1^): 3431, 3336, 1687; MS (z/e): 516(M)^+^, 322, 258, 95, 67, 55; Anal. Calcd for C_16_H_17_Cl_4_F_3_N_2_O_3_S: C, 37.23; H, 3.32; N, 5.43; found: C, 37.08; H, 3.53; N, 5.27.

### *N*-(2-trifluoromethyl-4-chlorophenyl)-5-ethyl-2-(2,2,2-trichloroacetylamino) cyclohexylsulfonamide (IV-35)

(n = 1, R^1^ = 5-Et) White crystal; yield, 93%; mp 122–125 °C; ^1^H NMR (DMSO*-d*_*6*_) *δ*: 0.84(t, *J* = 7.4 Hz, 3H, CH_3_), 1.15–2.35 (m, 9H, C_5_H_9_), 3.83 (d, *J* = 4.4 Hz, 1H, CH-N), 4.17 (dd, *J* = 10.4, 6.7 Hz, 1H, CH-SO_2_), 7.55–7.84 (m, 3H, Ph-H), 8.67 (d, *J* = 6.6 Hz, 1H, CO-NH), 9.81 (s, 1H, SO_2_-NH); ^13^C NMR (DMSO-*d*_*6*_) *δ*: 11.75, 26.04, 27.69, 28.54, 30.29, 32.20, 51.19, 60.10, 92.73, 121.94, 123.76, 125.58, 127.26, 131.13, 131.84, 133.76, 160.91; IR (*ν*, cm^−1^): 3394, 3261, 1705; MS (z/e): 530(M)^+^, 320, 256, 162, 95, 67; Anal. Calcd for C_17_H_19_Cl_4_F_3_N_2_O_3_S: C, 38.51; H, 3.61; N, 5.28; found: C, 38.69; H, 3.50; N, 5.04.

### *N*-(2-trifluoromethyl-4-chlorophenyl)-5-tertiarybutyl-2-(2,2,2-trichloroacetylamino) cyclohexylsulfonamide (IV-36)

(n = 1, R^1^ = 5-*t*-Bu) Colorless crystal; yield, 90%; mp 124–126 °C; ^1^H NMR (DMSO*-d*_*6*_) *δ*: 0.87–2.21 (m, 16H, C_8_H_16_), 3.57 (dt, *J* = 12.9, 3.3 Hz, 1H, CH-N), 4.38 (dd, *J* = 5.9, 3.1 Hz, 1H, CH-SO_2_), 7.61–7.83 (m, 3H, Ph-H), 7.97 (d, *J* = 6.0 Hz, 1H, CO-NH), 9.85 (s, 1H, SO_2_-NH); ^13^C NMR (DMSO-*d*_*6*_) *δ*: 20.55, 22.95, 27.55, 32.69, 46.21, 47.02, 62.47, 92.92, 121.95, 123.77, 127.12, 132.13, 132.36, 133.42, 133.72, 160.95; IR (*ν*, cm^−1^): 3400, 3284, 1708; MS (z/e): 530(M)^+^, 109, 67; Anal. Calcd for C_19_H_23_Cl_4_F_3_N_2_O_3_S: C, 40.88; H, 4.15; N, 5.02; found: C, 41.02; H, 3.98; N, 5.21.

### Bioassays

#### *In vitro* fungicidal activity

*In vitro* effects of compounds against *B. cinerea* were evaluated by mycelium growth rate method^30^,^31^,^32^. The tested compounds were dissolved in DMSO (dimethyl sulfoxide) and mixed with sterile molten potato dextrose agar (PDA) to a final concentration of 50 mg/L. EC_50_ values were estimated using logit analysis. The concentration gradients were 50, 12.5, 3.13, 0.78 mg/L on PDA and a commercial fungicide procymidone was used as the positive control. EXCEL 2010 was used to analyze bioassay data. The variance analysis was carried out by using SPSS 20.0 software for the inhibition rate, EC_50_ and control efficiency.

The relative inhibition rate of the synthetic compounds compared to blank control was calculated *via* the following equation (1):


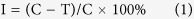


In which, I stands for the rate of inhibition (%), C is the diameter of mycelia in the blank control test (in mm), and T is the diameter of mycelia in the presence of tested compounds (in mm).

#### *In vivo* antifungal activity

*In vivo* effects were checked on leaves of cucumber (*Cucumis sarivus* L.) by mycelium inoculation method with pot cultural test in greenhouse^37^,^38^,^39^,^40^. The cucumber seedlings at 2–3 leaf stages were used to assay the fungicidal activity against *B. cinerea*. The compounds were confected to 2.5% EC (emulsifiable concentrate) formulation. The formulation was diluted to 500 mg/L with water and sprayed on the surface of the cucumber leaves. After air drying, the surface of the leaves was inoculated with 6 mm plugs of *B. cinerea*, which was maintained on potato dextrose agar (PDA). This procedure was repeated three times, and nine replicates were performed per treatment. The chesulfamide (**L**, Fig. 3) was used as the positive control.

The fungicidal activity was assessed when the untreated cucumber plant (blank control) fully developed symptoms. The area of inoculated leaves covered by disease symptoms was evaluated and compared to that of untreated ones to determine the average disease index. The relative control efficacy of compounds compared to the blank assay was calculated *via* the following equation (2):


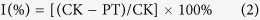


where I is relative control efficacy, CK is the average disease index during the blank assay and PT is the average disease index after treatment during testing.

## Additional Information

**How to cite this article**: Liu, C.-H. *et al*. Synthesis, Fungicidal Activity, and Structure Activity Relationship of *β*-Acylaminocycloalkylsulfonamides against *Botrytis cinerea. Sci. Rep.* **7**, 42096; doi: 10.1038/srep42096 (2017).

**Publisher's note:** Springer Nature remains neutral with regard to jurisdictional claims in published maps and institutional affiliations.

## Supplementary Material

Supplementary Materials

## Figures and Tables

**Figure 1:**
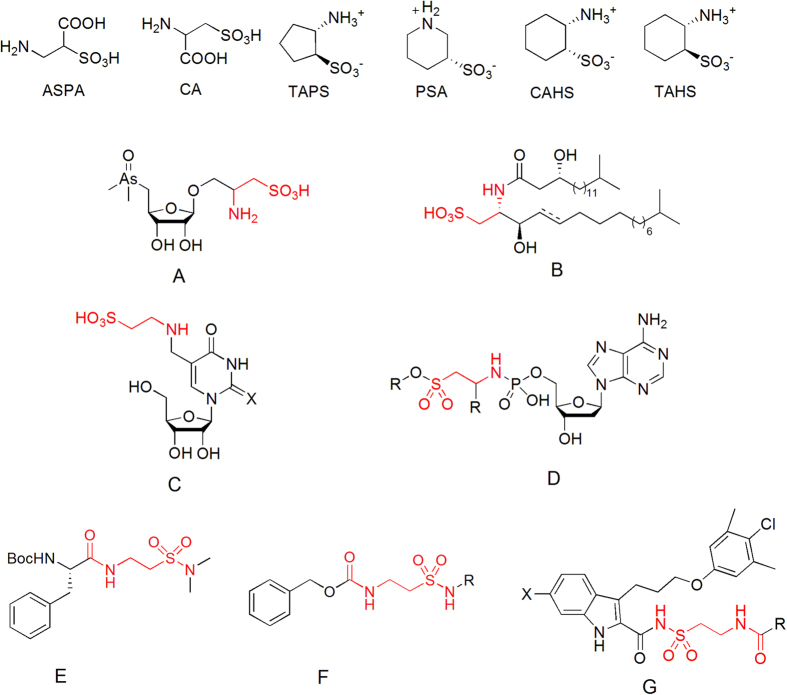
Taurine derivatives.

**Figure 2:**
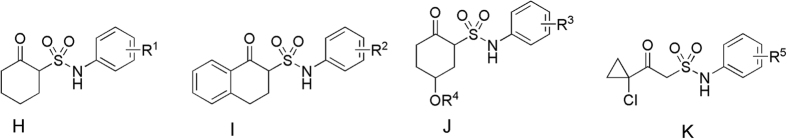
2-Oxycycloalkylsulfonamides derivatives.

**Table 1:** EC_50_ values of title compounds **IV** against six *B. cinerea* strains.

Compound Number	R^2^	EC_50_ (mg/L) (95% confidence limits of EC50)					
		As-12	Cy-07	Dd-04	Dl-17	Fs-06	Hld-16
IV-23 ab	CH_3_(CH_2_)_5_―	13.87 (9.25–20.81)	18.13 (9.92–33.14)	9.14 (6.34–13.18)	15.19 (11.63–19.85)	13.63 (10.12–18.36)	19.40 (13.67–27.53)
IV-24 ab	ClCH_2_―	16.14 (11.94–21.81)	12.34 (8.44–18.05)	8.89 (5.94–13.30)	13.69 (10.06–18.62)	25.81 (14.13–47.16)	25.44 (19.80–32.68)
IV-26 a	Cl_3_C―	4.79 (3.37–6.81)	1.60 (0.65–3.91)	1.97 (1.18–3.28)	0.37 (0.07–2.08)	7.56 (4.57–12.51)	5.88 (3.36–10.30)
IV-29 ab	C_2_H_5_OCH_2_―	13.27 (10.57–16.67)	10.37 (6.78–15.88)	5.29 (3.51–7.99)	11.81 (8.50–16.39)	8.94 (7.20–11.09)	31.90 (19.81–51.36)
procymidone bc	—	16.93 (10.28–27.88)	21.36 (14.18–32.18)	8.17 (5.80–11.49)	2.49 (1.33–4.66)	75.84 (36.17–159.00)	12.91 (9.15–18.20)

The letters a–c denoted the difference significance analysis results of the same compound against six different strains. Means followed by the same letter within the same column are not significantly different (p > 0.05, Fisher1s LSD multiple comparison test).

**Table 2:** EC_50_ values of title compounds **IV-26** and **IV-30**~**IV-36** against five *B. cinerea* strains.

Compound number	*n*	R^1^	EC_50_ (mg/L) (95% confidence limits of EC_50_)				
			As-11	Cy-09	Dl-11	Fs-11	Hld-15
IV-26 a	2	H	0.41 (0.08–2.01)	1.13 (0.46–2.75)	0.15 (0.02–1.26)	3.64 (1.72–7.72)	1.87 (1.06–3.31)
IV-30 a	1	H	0.66 (0.17–2.48)	2.28 (1.54–3.38)	0.77 (0.32–1.87)	11.68 (9.08–15.03)	0.85 (0.39–1.86)
IV-31 a	3	H	4.59 (2.87–7.33)	1.36 (0.58–3.15)	0.96 (0.47–1.99)	9.49 (2.22–40.56)	0.82 (0.20–3.26)
IV-32 a	2	3-CH_3_	14.76 (2.92–74.55)	10.71 (0.71–160.53)	0.01 (0.00–25.75)	7.93 (2.60–24.18)	0.96 (0.13–7.13)
IV-33 a	2	4-CH_3_	0.18 (0.01–2.95)	2.23 (0.37–13.43)	0.15 (0.02–1.32)	15.56 (5.34–45.32)	0.15 (0.01–2.67)
IV-34 a	2	5-CH_3_	0.56 (0.06–4.85)	6.19 (2.94–13.04)	2.22 (1.37–3.59)	16.75 (7.52–37.27)	1.19 (0.47–2.98)
IV-35 ab	2	5-C_2_H_5_	51.4 (2.52–1049.18)	19.87 (1.42–277.31)	0.22 (0.01–4.01)	16.42 (5.30–50.84)	31.99 (3.84–266.66)
IV-36 c	2	5-C(CH_3_)_3_	>100	44.12 (18.73–103.92)	9.49 (6.30–14.28)	>100	30.76 (18.10–52.25)
procymidone bc	—	—	0.22 (0.07–0.65)	20.00 (14.52–27.55)	4.40 (3.43–5.65)	>100	>100

The letters a–d denoted the difference significance analysis results of the same compound against five different strains. Means followed by the same letter within the same column are not significantly different (p > 0.05, Fisher1s LSD multiple comparison test).

**Table 3:** Control efficiency of compounds against *B. cinerea* on leaves of cucumber.

Compd.	Inhibition rate (%) ± SEM
**IV-25**	24.54 ± 11.43 b
**IV-26**	37.58 ± 32.58 ab
**IV-30**	35.78 ± 16.95 ab
**IV-31**	64.30 ± 15.57 a
**IV-32**	39.37 ± 22.08 ab
**IV-33**	36.68 ± 34.42 ab
chesulfamide	32.11 ± 23.32 ab

The letters a–b denoted the results of difference significance analysis. Means followed by the same letter within the same column are not significantly different (p > 0.05, Fisher1s LSD multiple comparison test).
