# Supplementary material for: Synthesis, Fungicidal Activity, and Structure Activity Relationship of β-Acylaminocycloalkylsulfonamides against Botrytis cinerea
Source: Sci Rep. 2017 Feb 8;7:42096. doi: 10.1038/srep42096 (PMC5296765; doi:10.1038/srep42096)
Supplement: Supplementary Materials [file srep42096-s1.doc]

**Synthesis, Fungicidal Activity, and Structure Activity Relationship of** *β***-Acylaminocycloalkylsulfonamides against *Botrytis cinerea***

Chun-Hui Liu^1,#^, Xiao-Yuan Chen^1,#^, Pei-Wen Qin^1^, Zhi-Qiu Qi^1^, Ming-Shan Ji^1^, Xing-Yu Liu^2^, P. Vijaya Babu^2^, Xing-Hai Li^1,*^ and Zi-Ning Cui^2,3,*^

^1^ Department of Pesticide Science, Plant Protection College, Shenyang Agricultural University, Shenyang 110866, Liaoning, China

^2^ State Key Laboratory for Conservation and Utilization of Subtropical Agro-bioresources, Integrative Microbiology Research Centre, Guangdong Province Key Laboratory of Microbial Signals and Disease Control, South China Agricultural University, Guangzhou 510642, China

^3^ Key Laboratory of Green Pesticide and Agricultural Bioengineering, Ministry of Education, Guizhou University, Guiyang 550025, China

* Correspondence: xinghai30@163.com (X.L.); ziningcui@scau.edu.cn (Z.C.); Tel.: +86-24-8834-2018 (X.L.); +86-20-8528-8229 (Z.C.); Fax: +86-24-8848-7148 (X.L.); +86-20-8528-8229 (Z.C.)

^#^Both authors contributed equally to this paper.

Figure S1-1~S49-1: ^1^H NMR spectra of compounds **I-4~ I-8**, **II-1~** **II-8** and **IV-1~ IV-36**

Figure S1-2~S49-2: ^1^C NMR spectra of compounds **I-4~ I-8**, **II-1~** **II-8** and **IV-1~ IV-36**

Table S1. Crystal and experimental data of compounds **IV-3** and **IV-31**

Table S2. Bond lengths and angels of compound **IV-3**

Table S3. Bond lengths and angels of compound **IV-31**


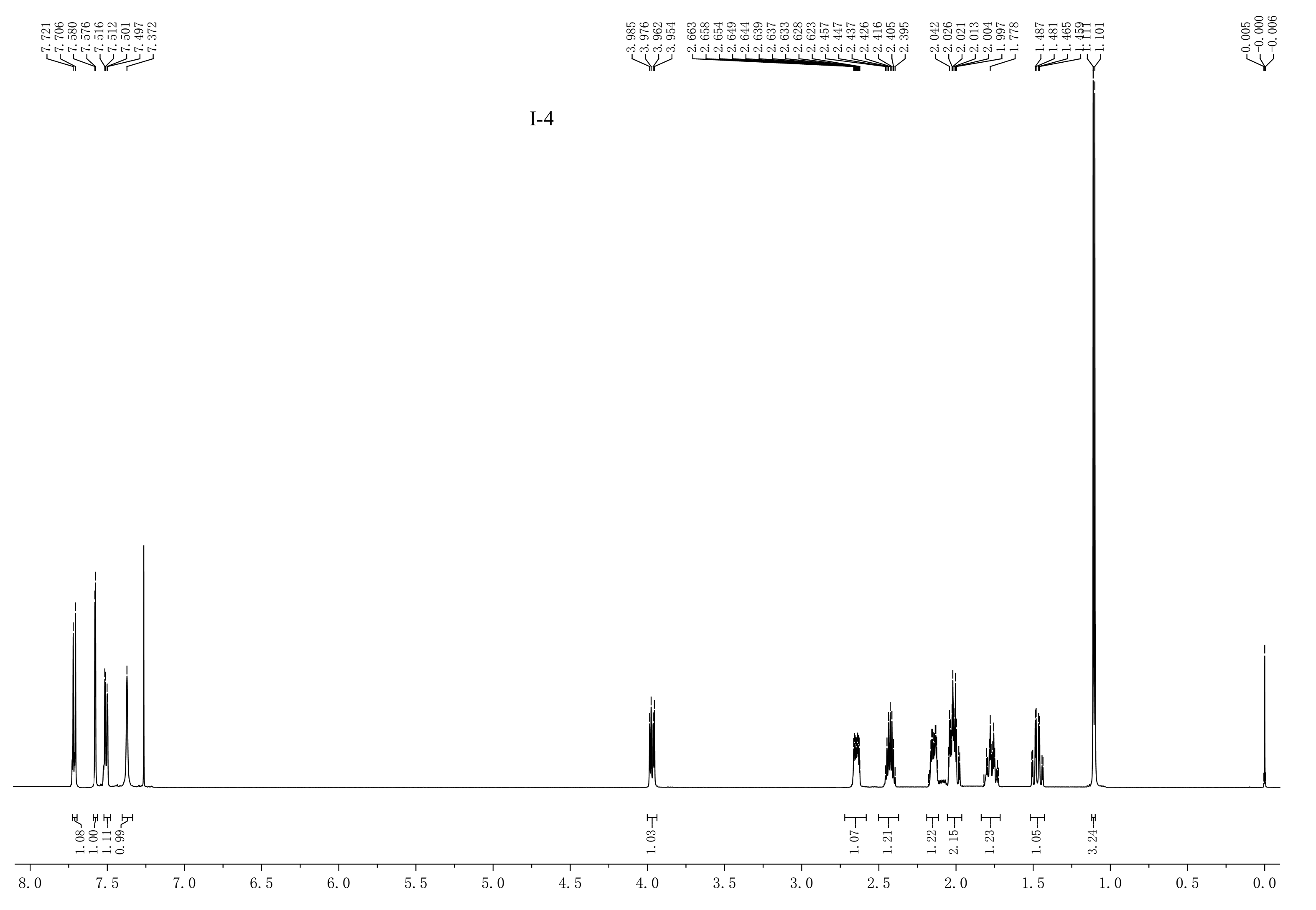


Figure S1-1 ^1^H NMR spectrum of compound **I-4**


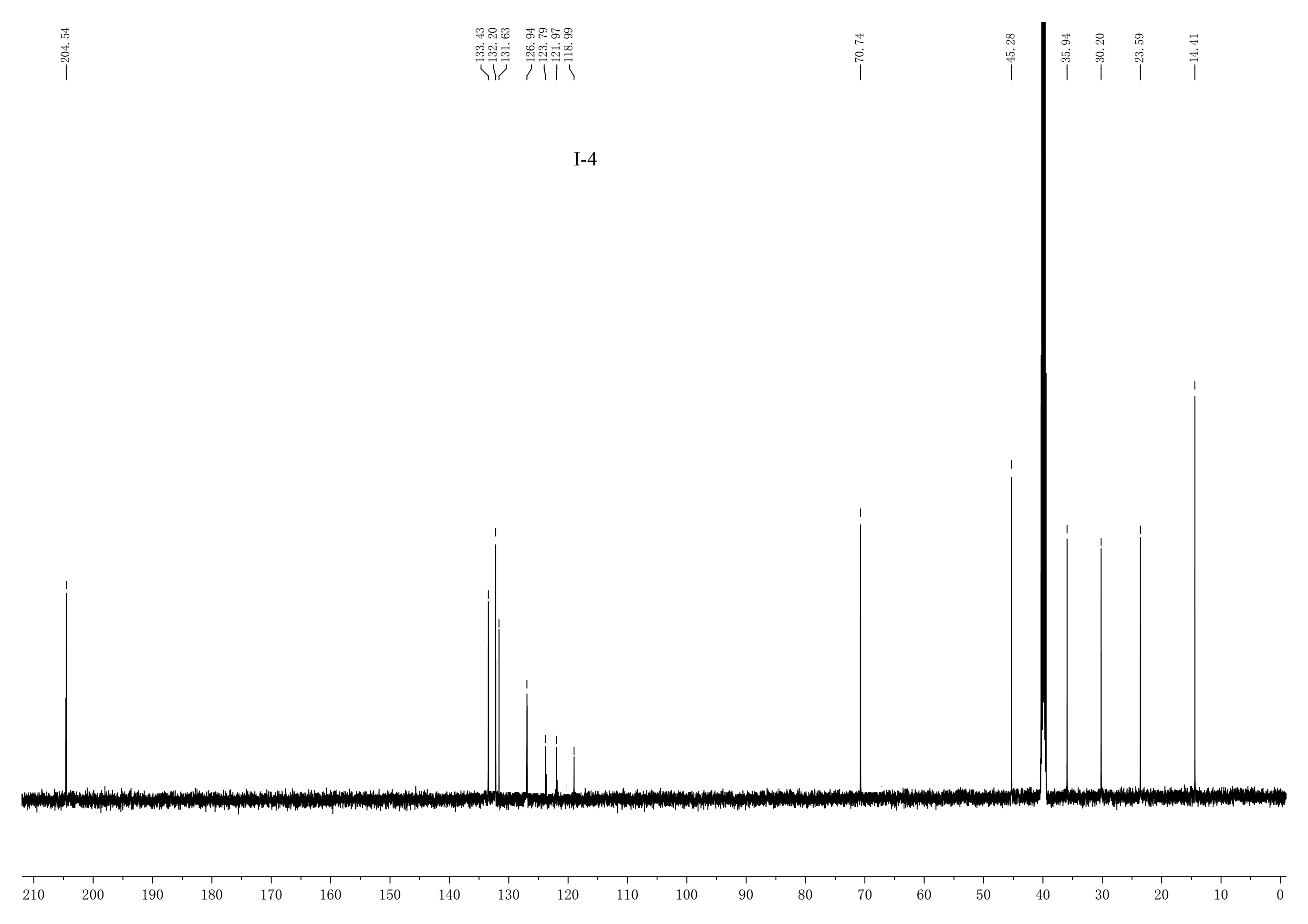


Figure S1-2 ^13^C NMR spectrum of compound **I-4**


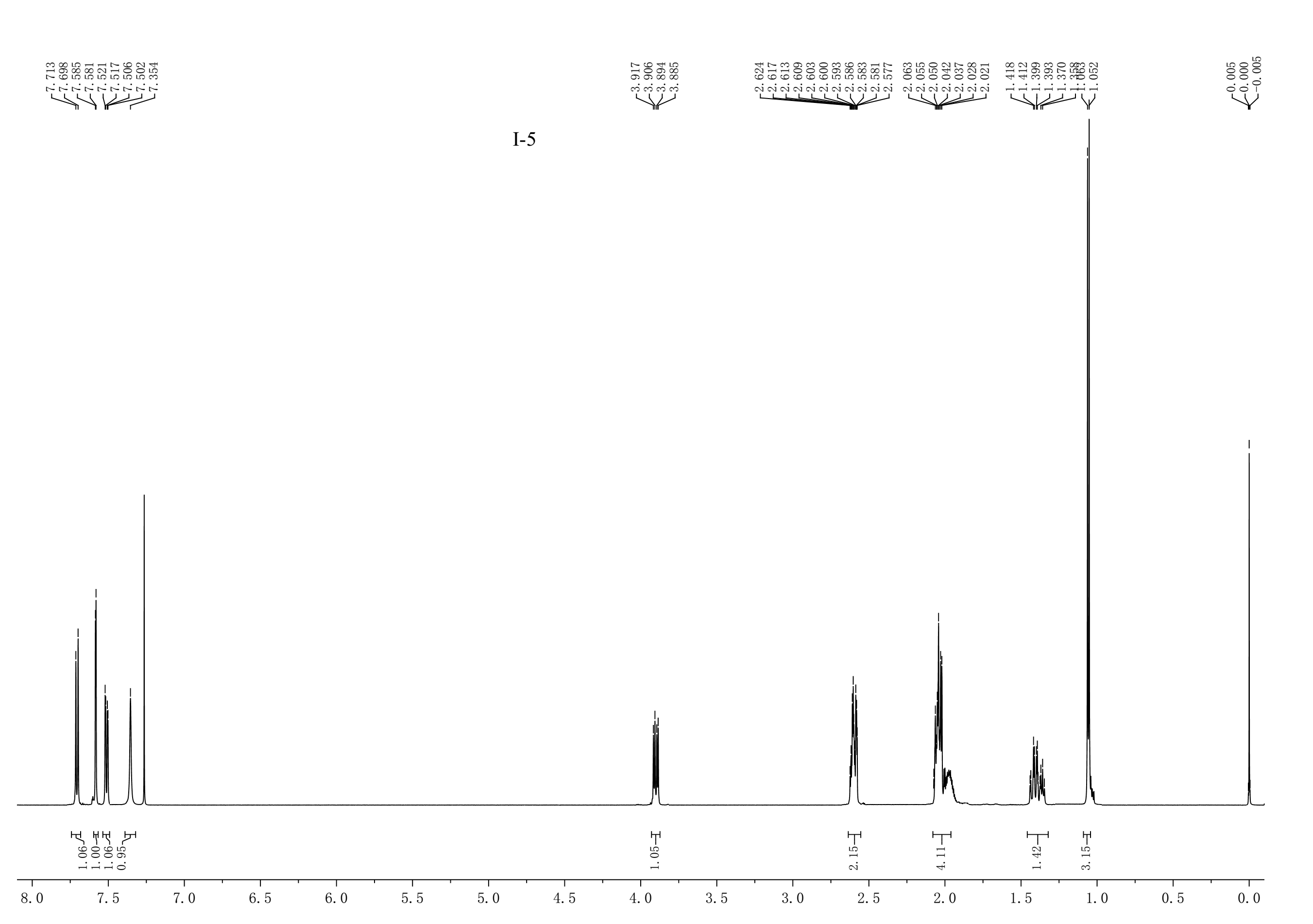


Figure S2-1 ^1^H NMR spectrum of compound **I-5**


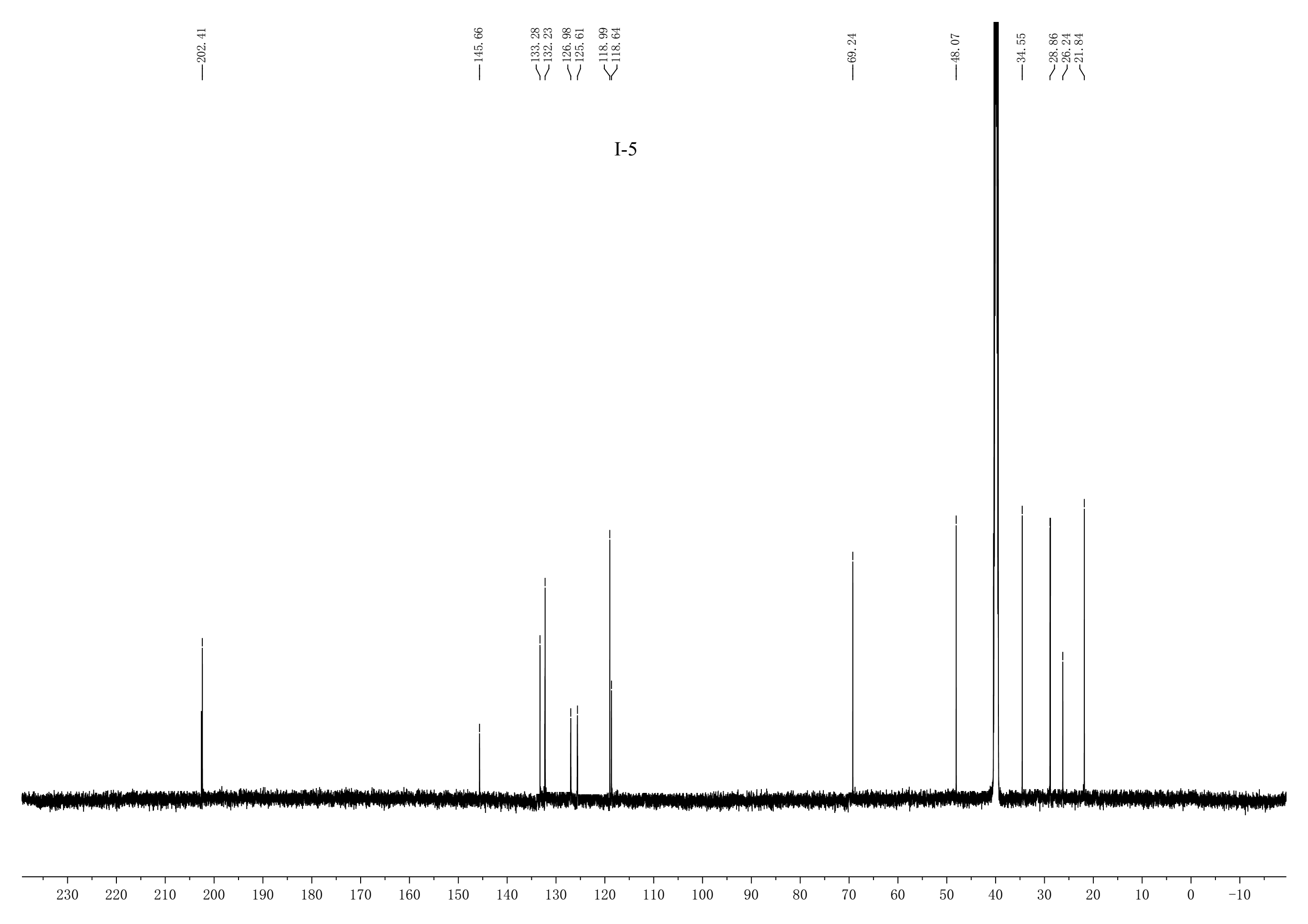


Figure S2-2 ^13^C NMR spectrum of compound **I-5**


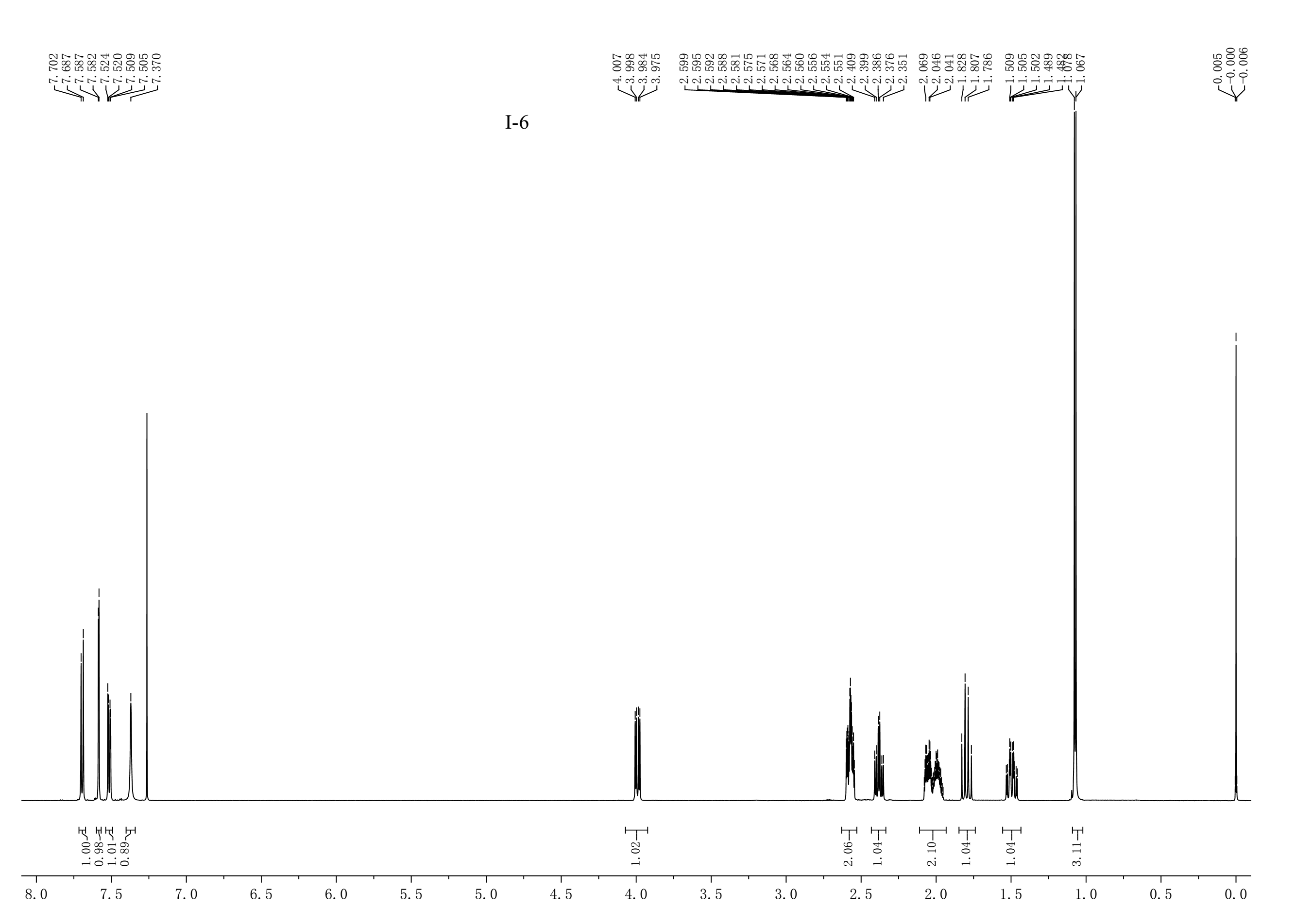


Figure S3-1 ^1^H NMR spectrum of compound **I-6**


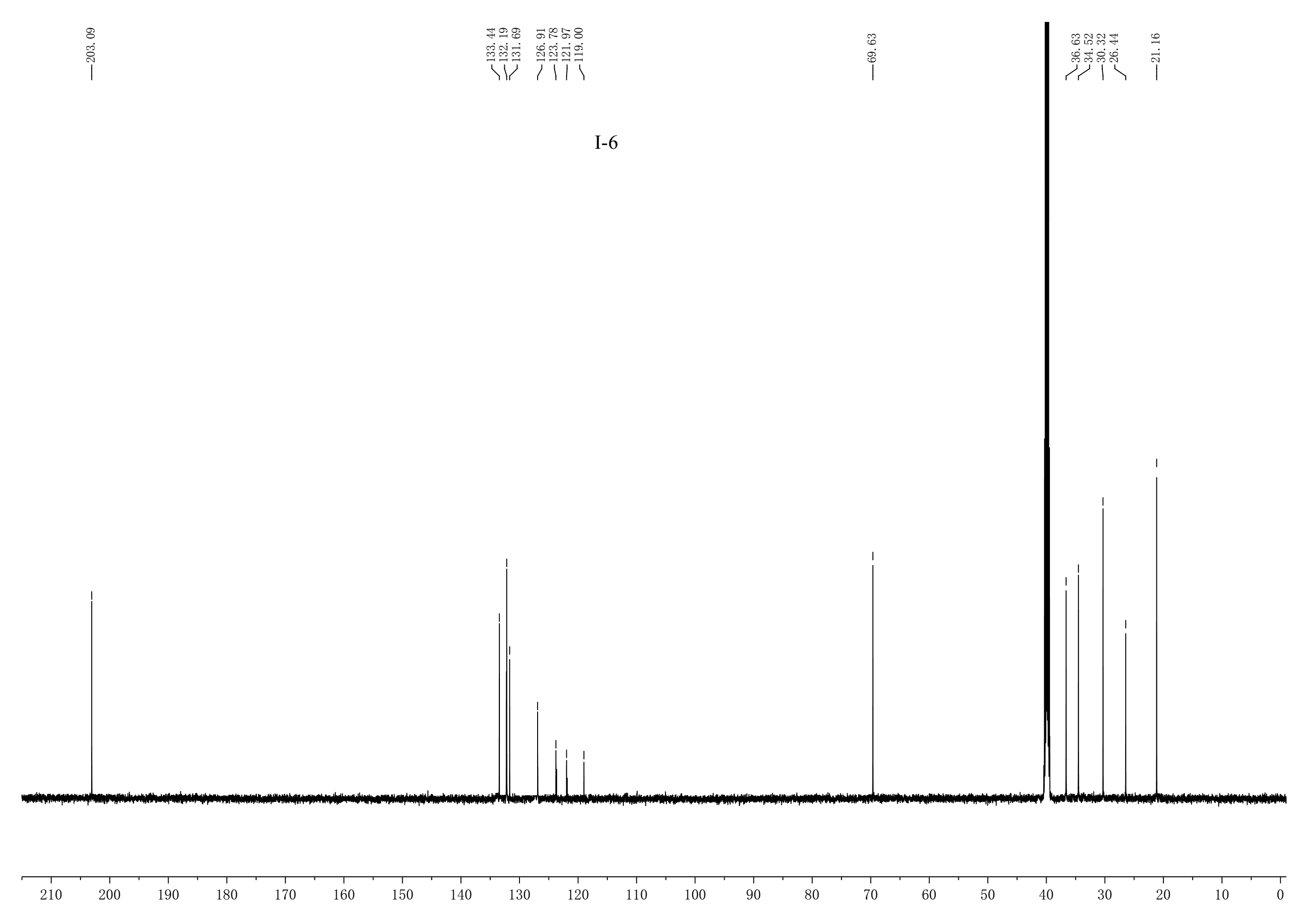


Figure S3-2 ^13^C NMR spectrum of compound **I-6**


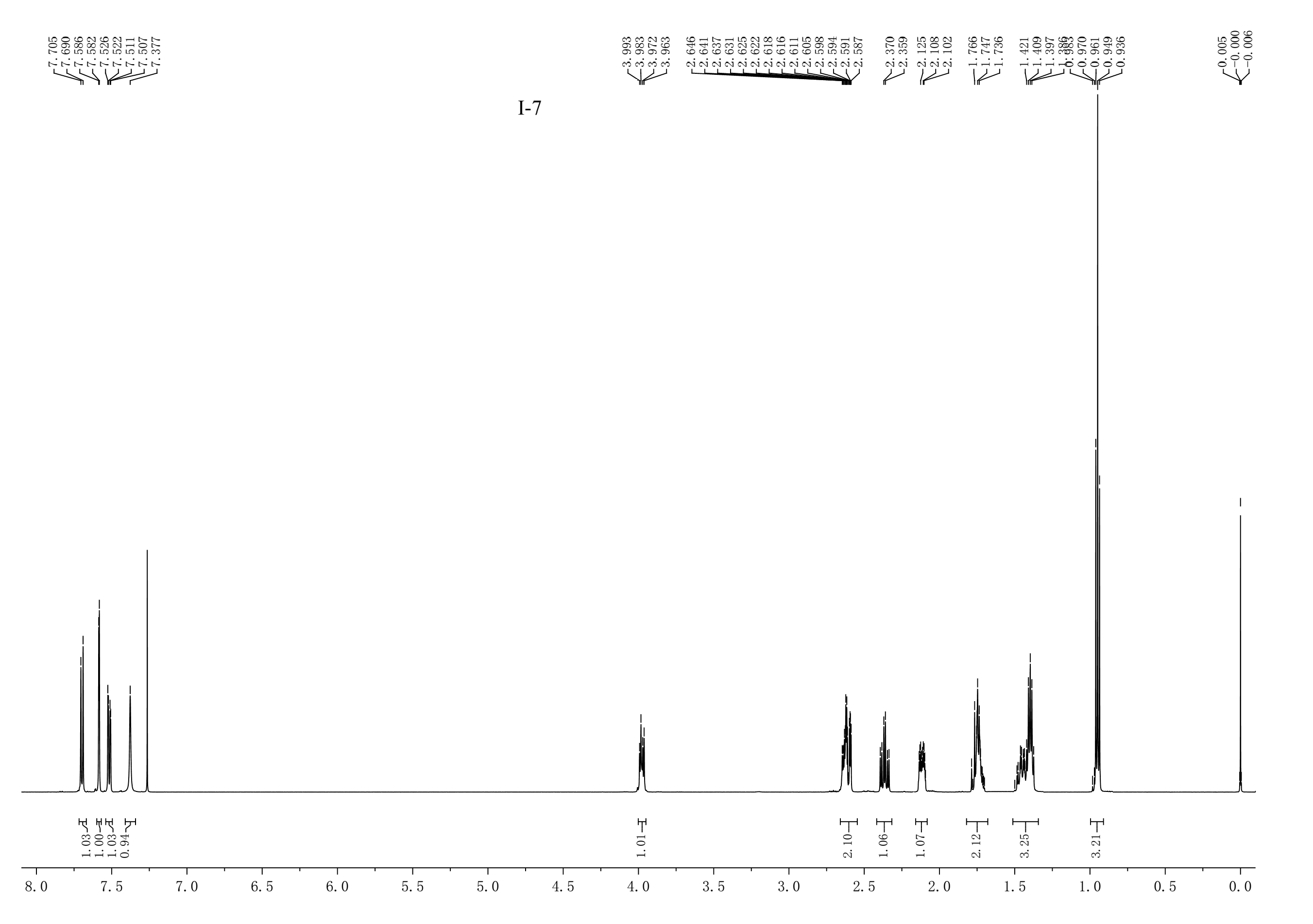


Figure S4-1 ^1^H NMR spectrum of compound **I-7**


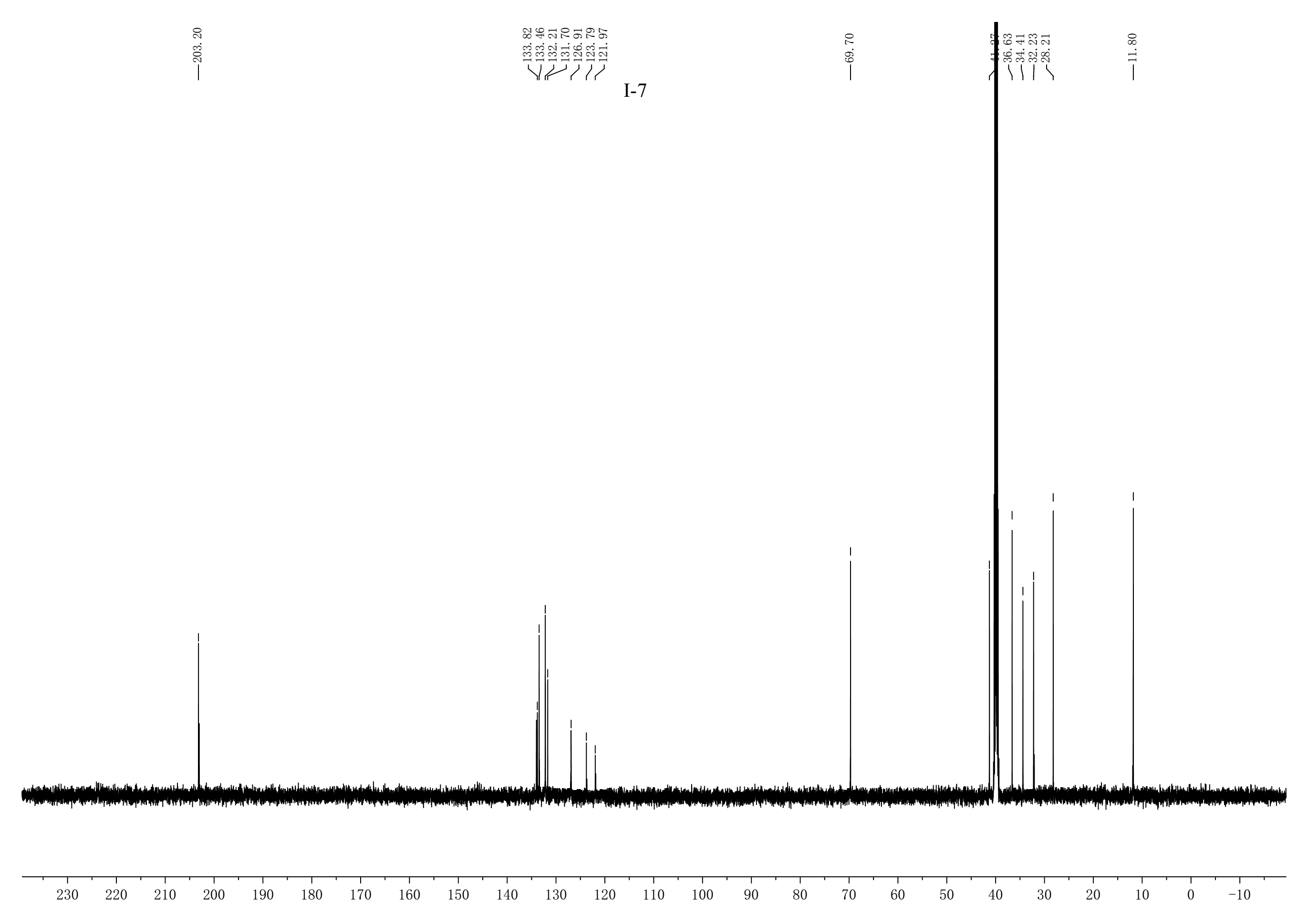


Figure S4-2 ^13^C NMR spectrum of compound **I-7**


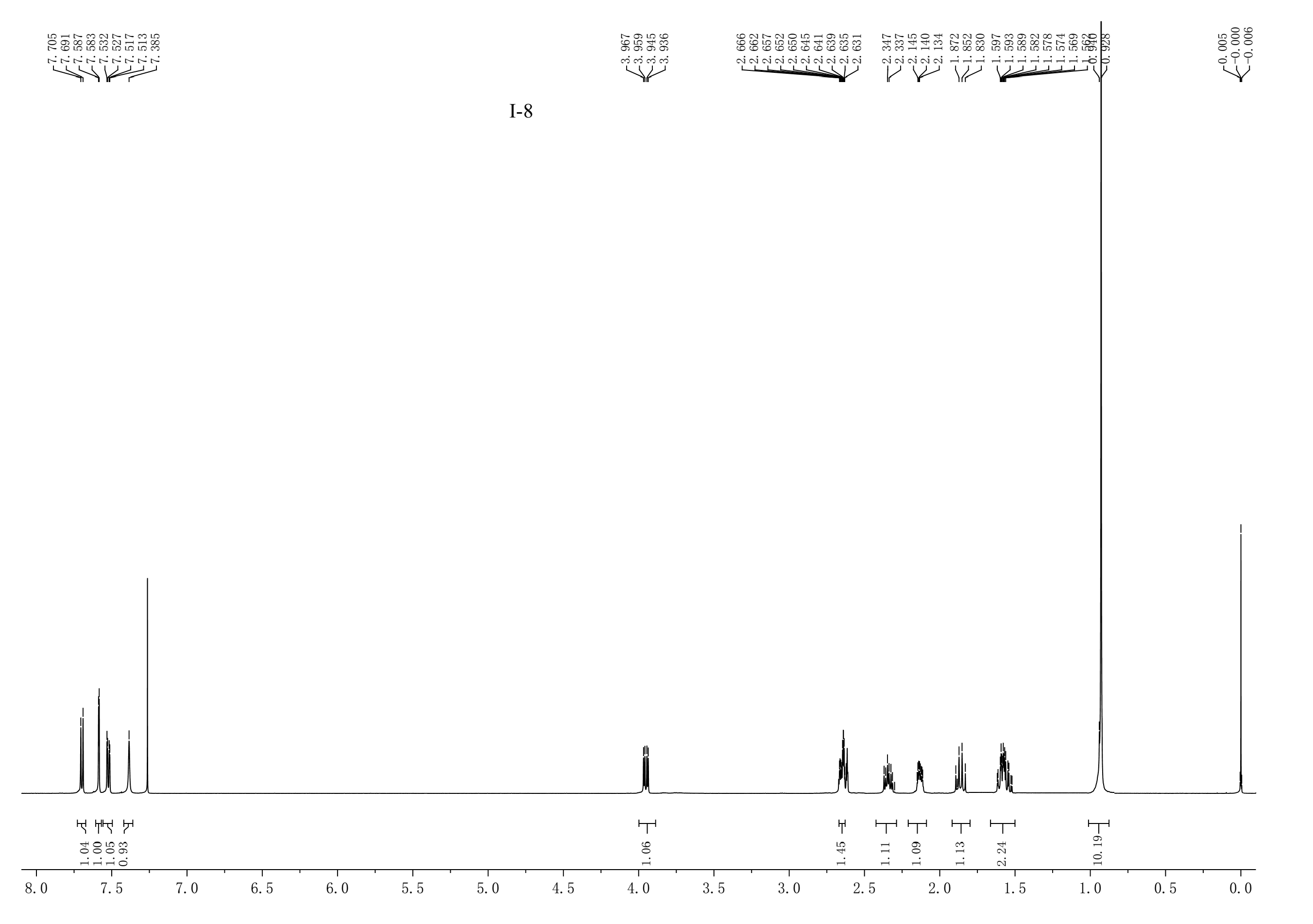


Figure S5-1 ^1^H NMR spectrum of compound **I-8**


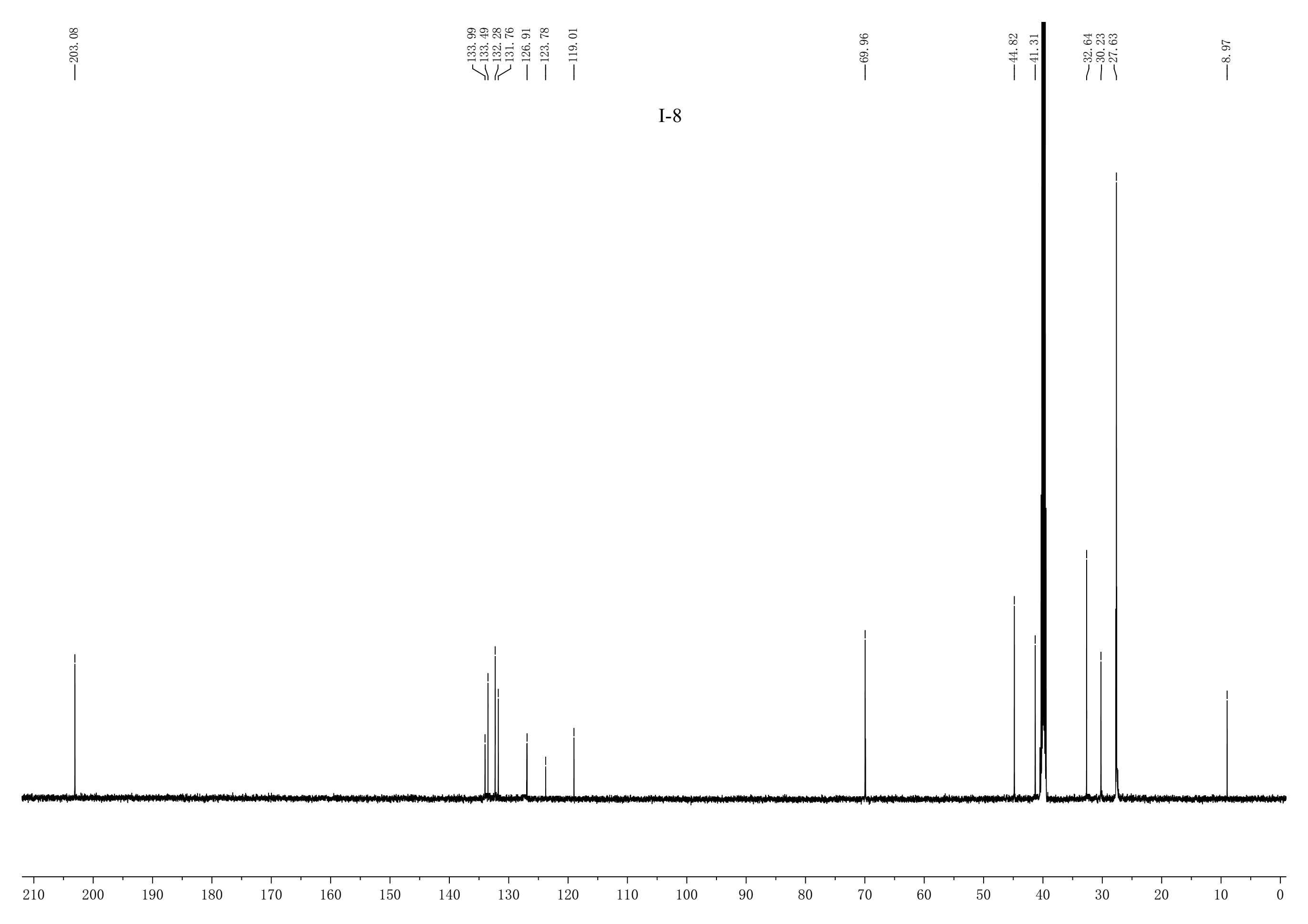


Figure S5-2 ^13^C NMR spectrum of compound **I-8**


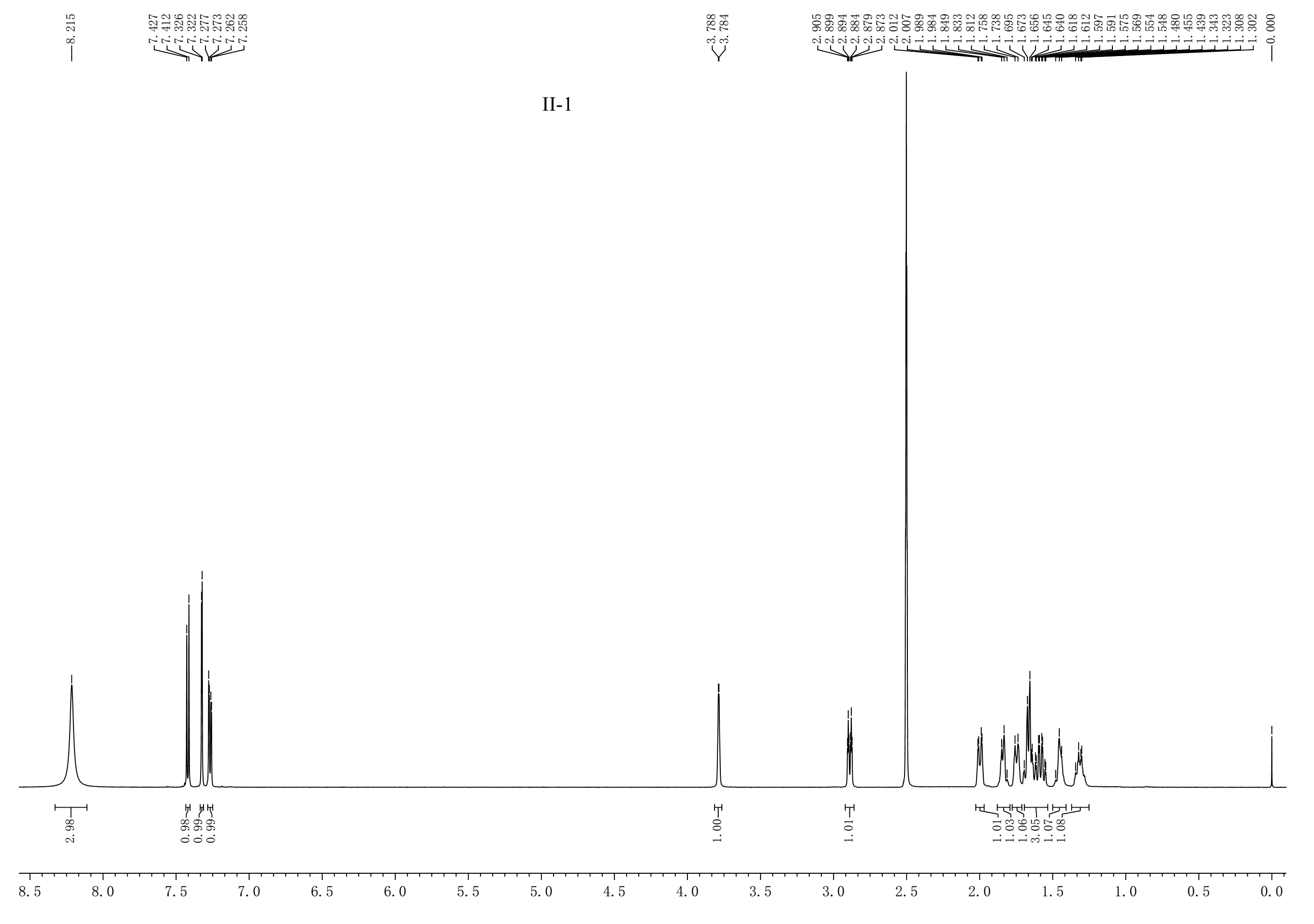


Figure S6-1 ^1^H NMR spectrum of compound **II-1**


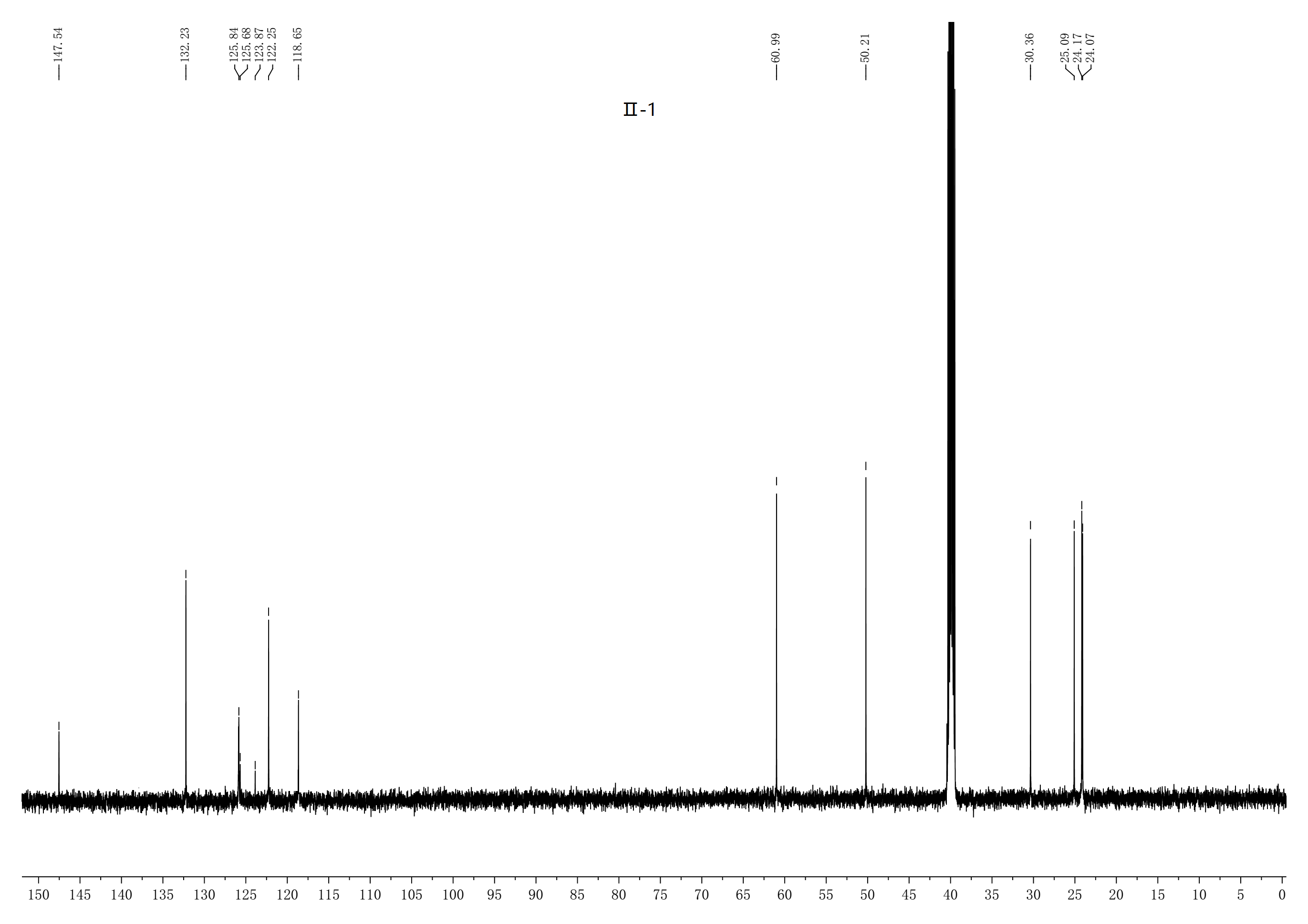


Figure S6-2 ^13^C NMR spectrum of compound **II-1**


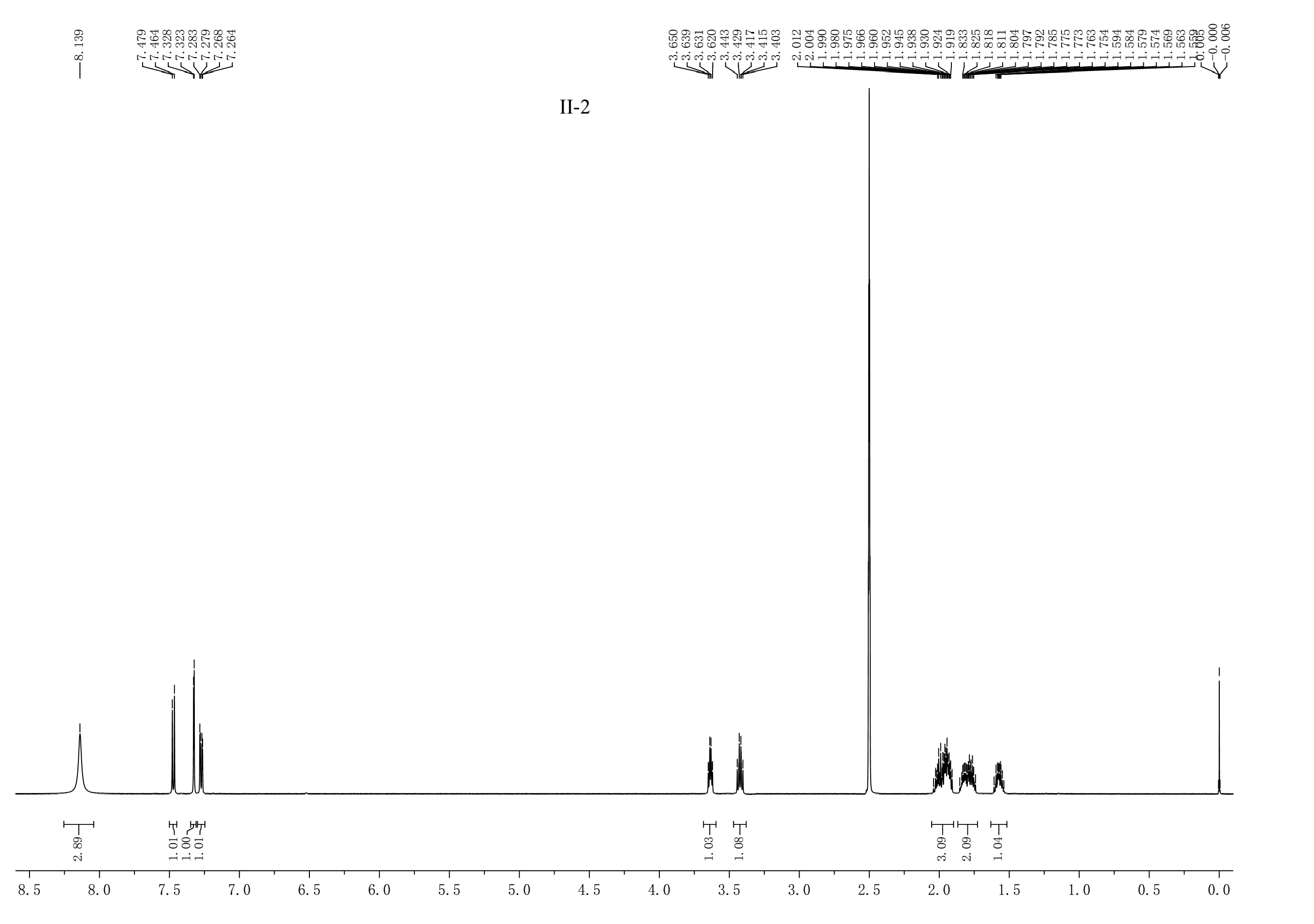


Figure S7-1 ^1^H NMR spectrum of compound **II-2**


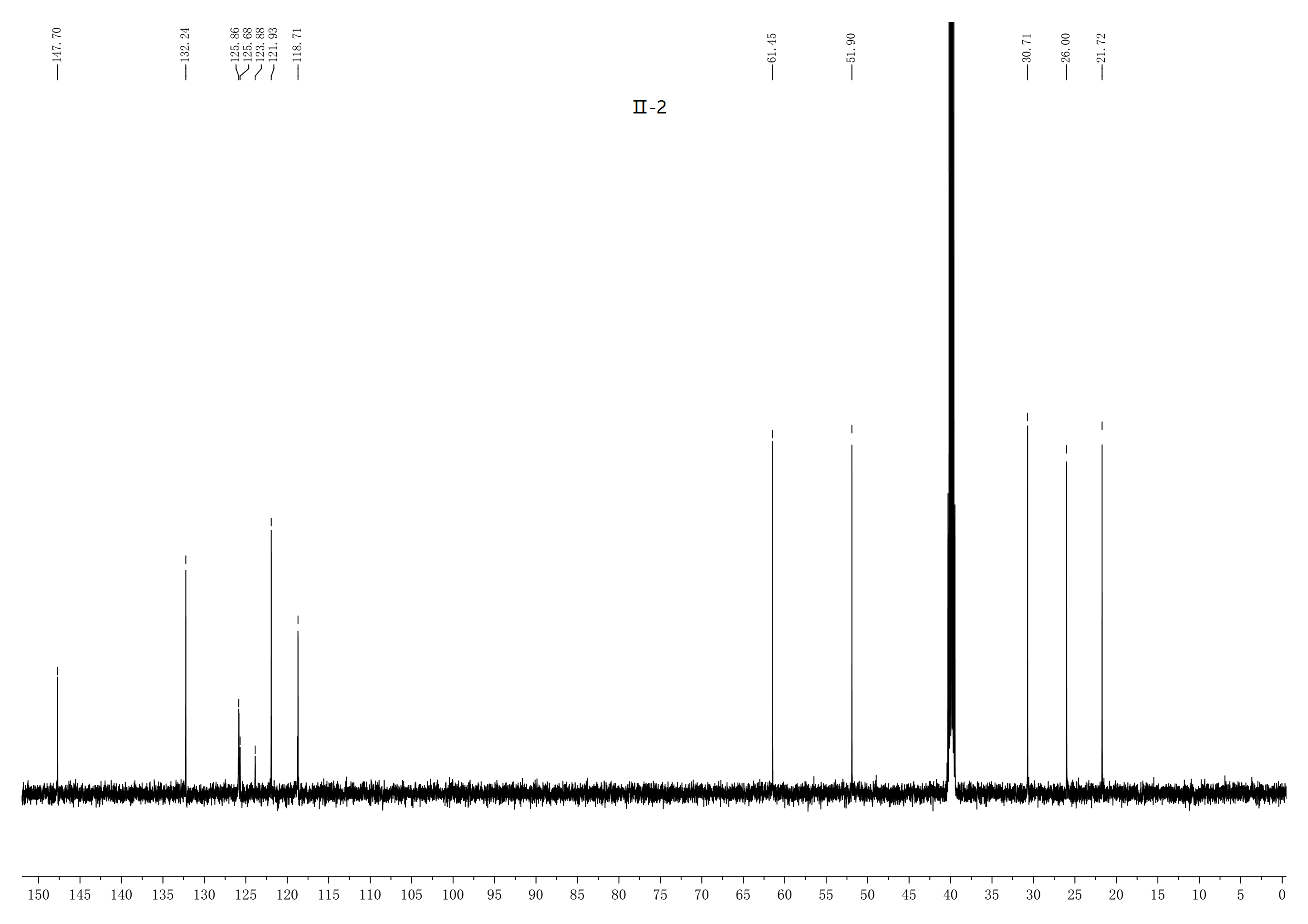


Figure S7-2 ^13^C NMR spectrum of compound **II-2**


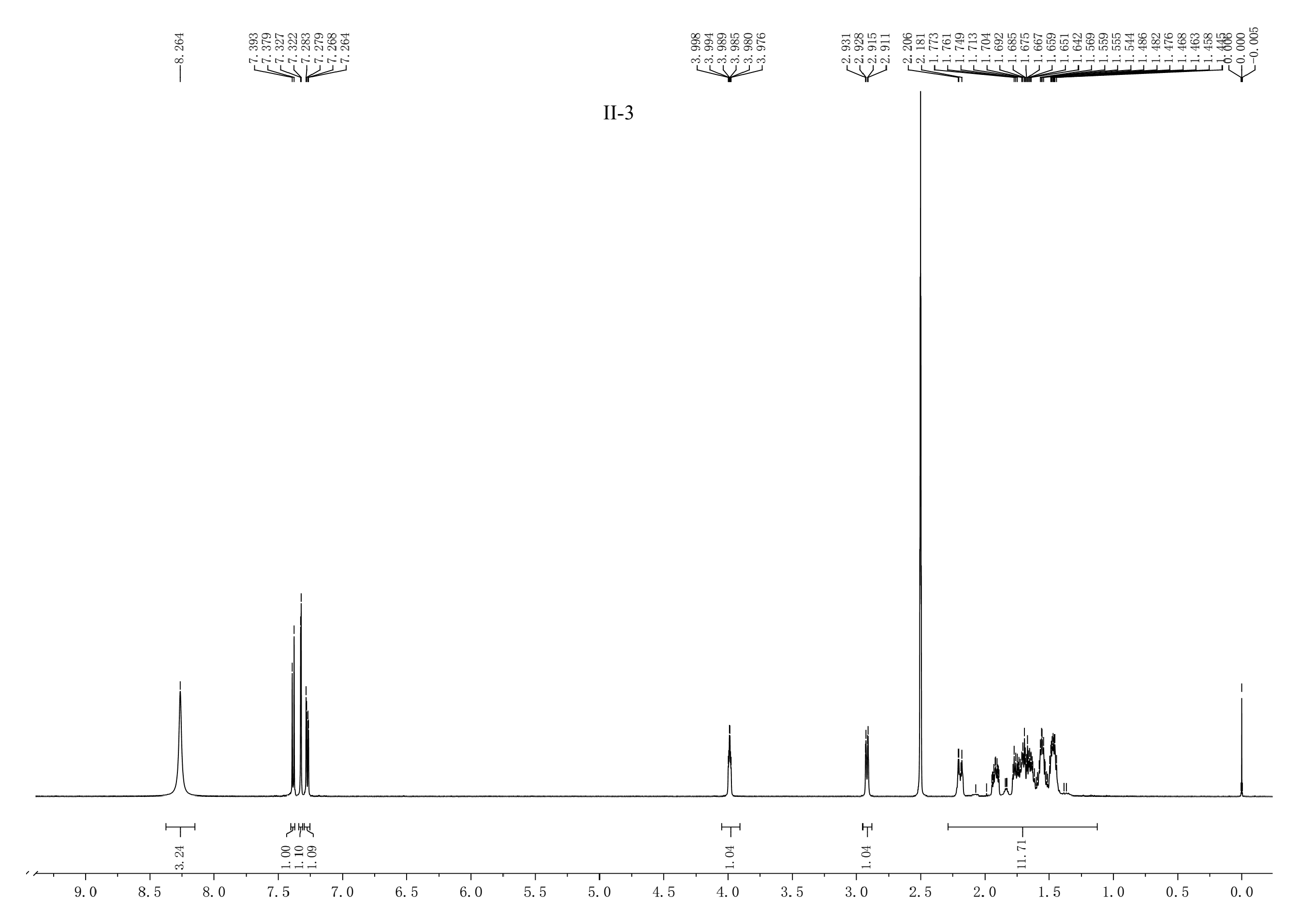


Figure S8-1 ^1^H NMR spectrum of compound **II-3**


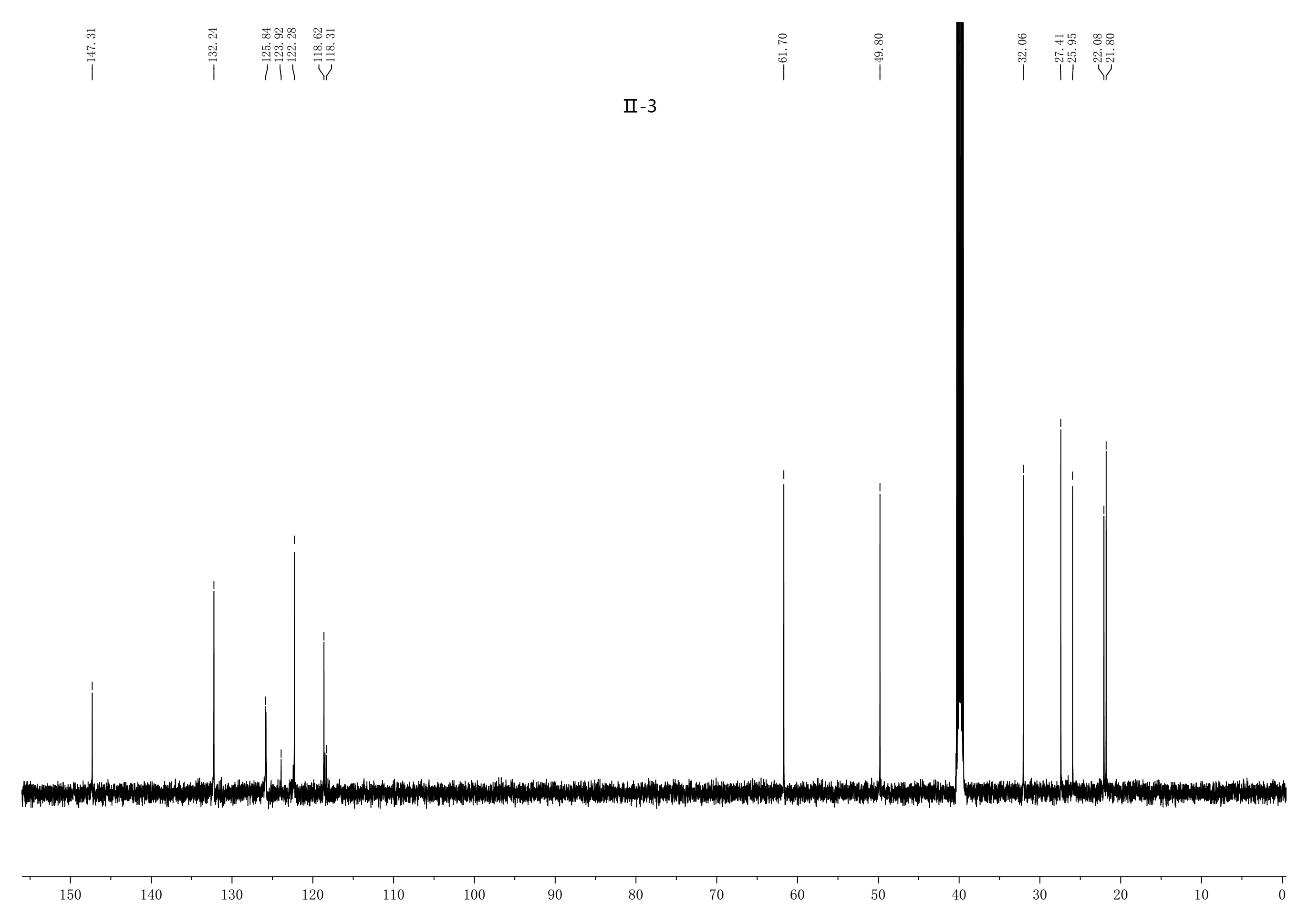


Figure S8-2 ^13^C NMR spectrum of compound **II-3**


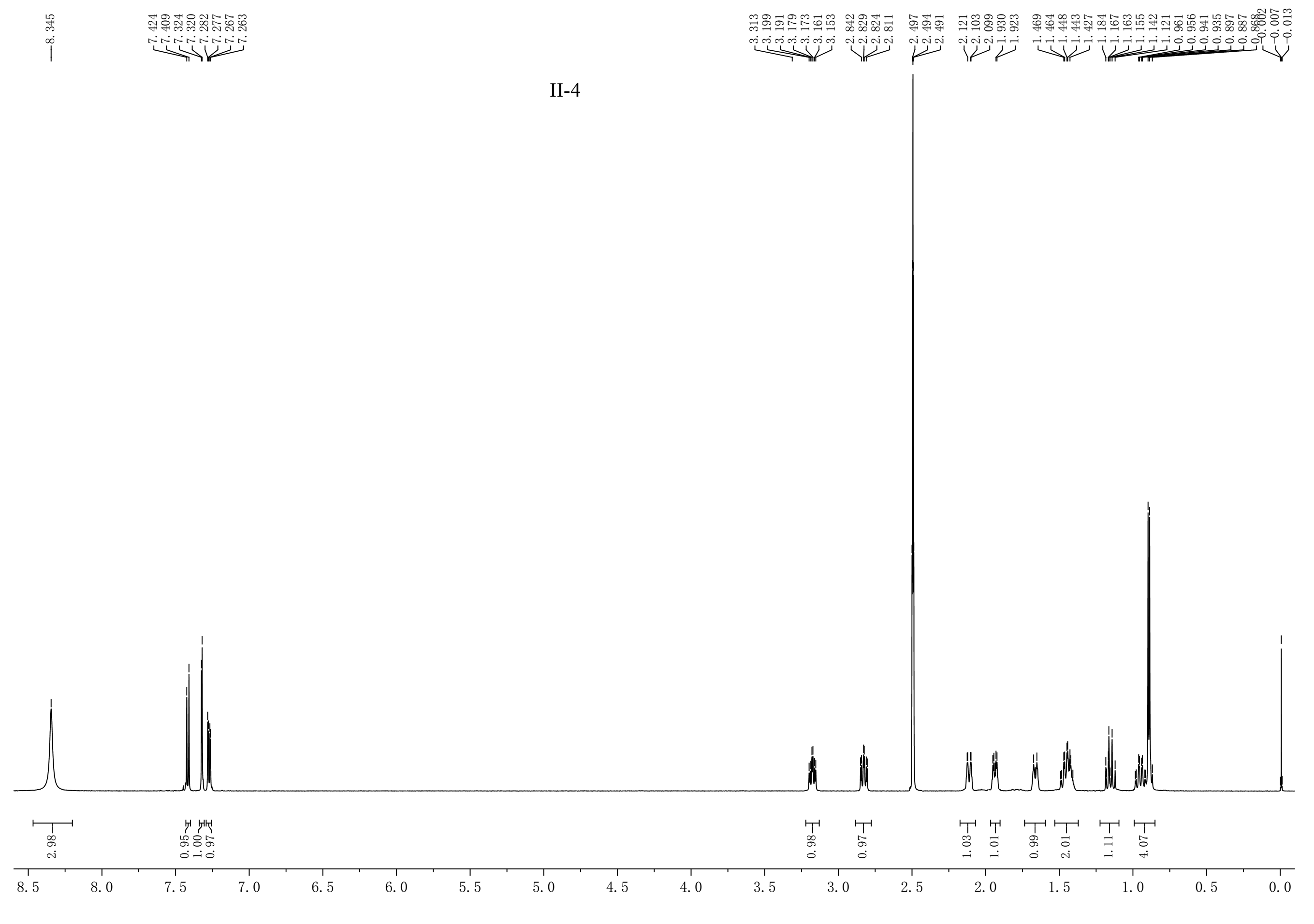


Figure S9-1 ^1^H NMR spectrum of compound **II-4**


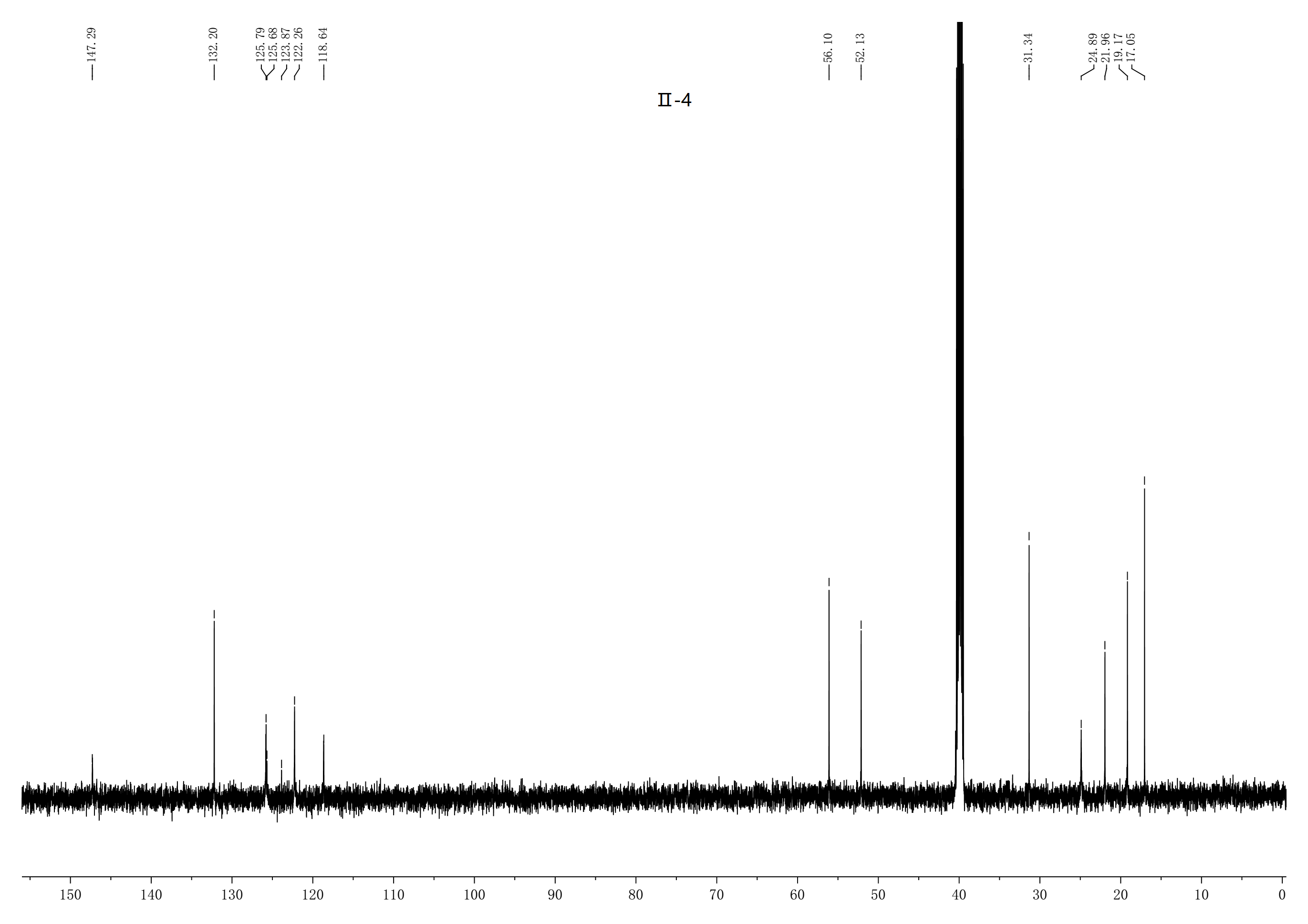


Figure S9-2 ^13^C NMR spectrum of compound **II-4**


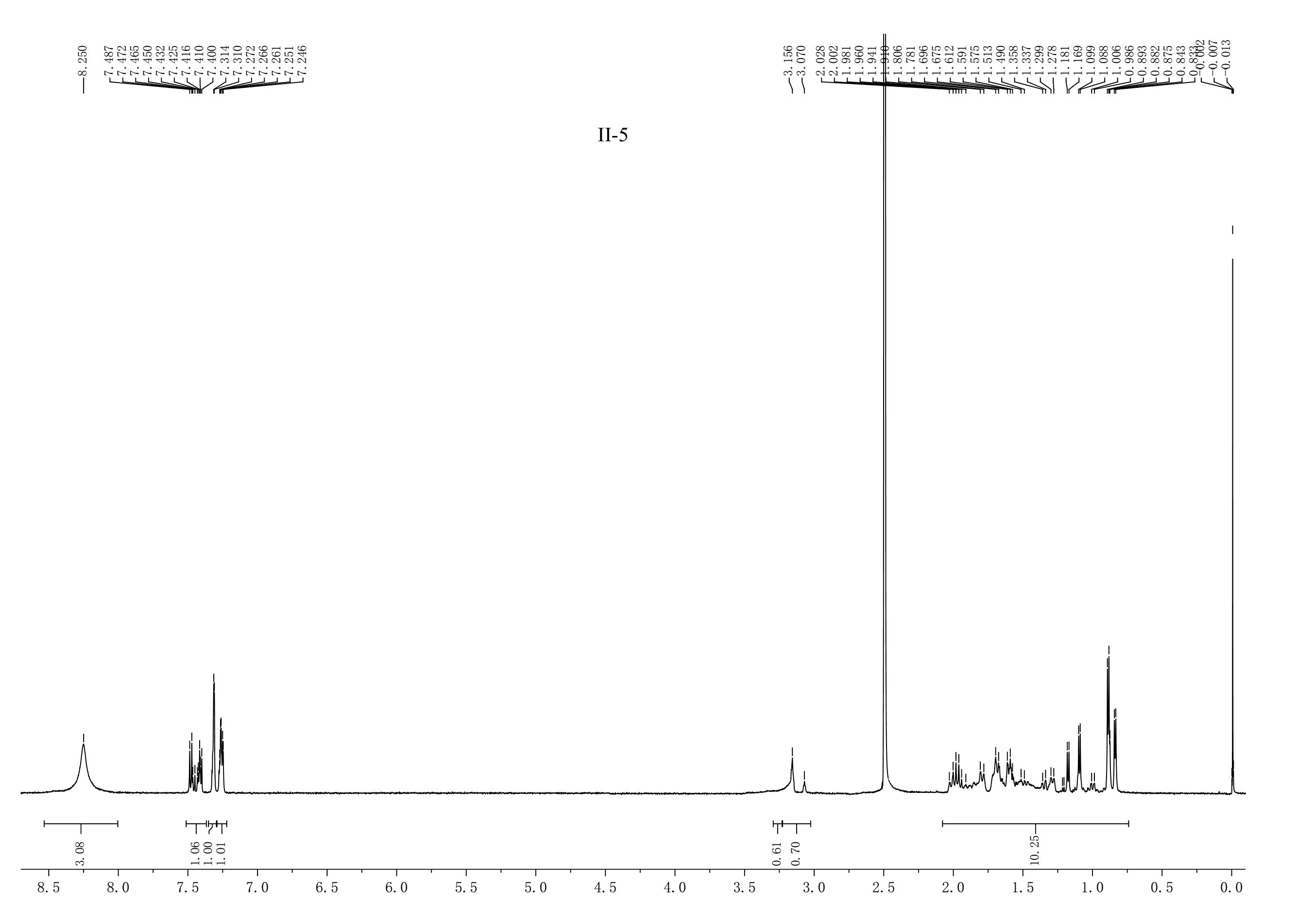


Figure S10-1 ^1^H NMR spectrum of compound **II-5**


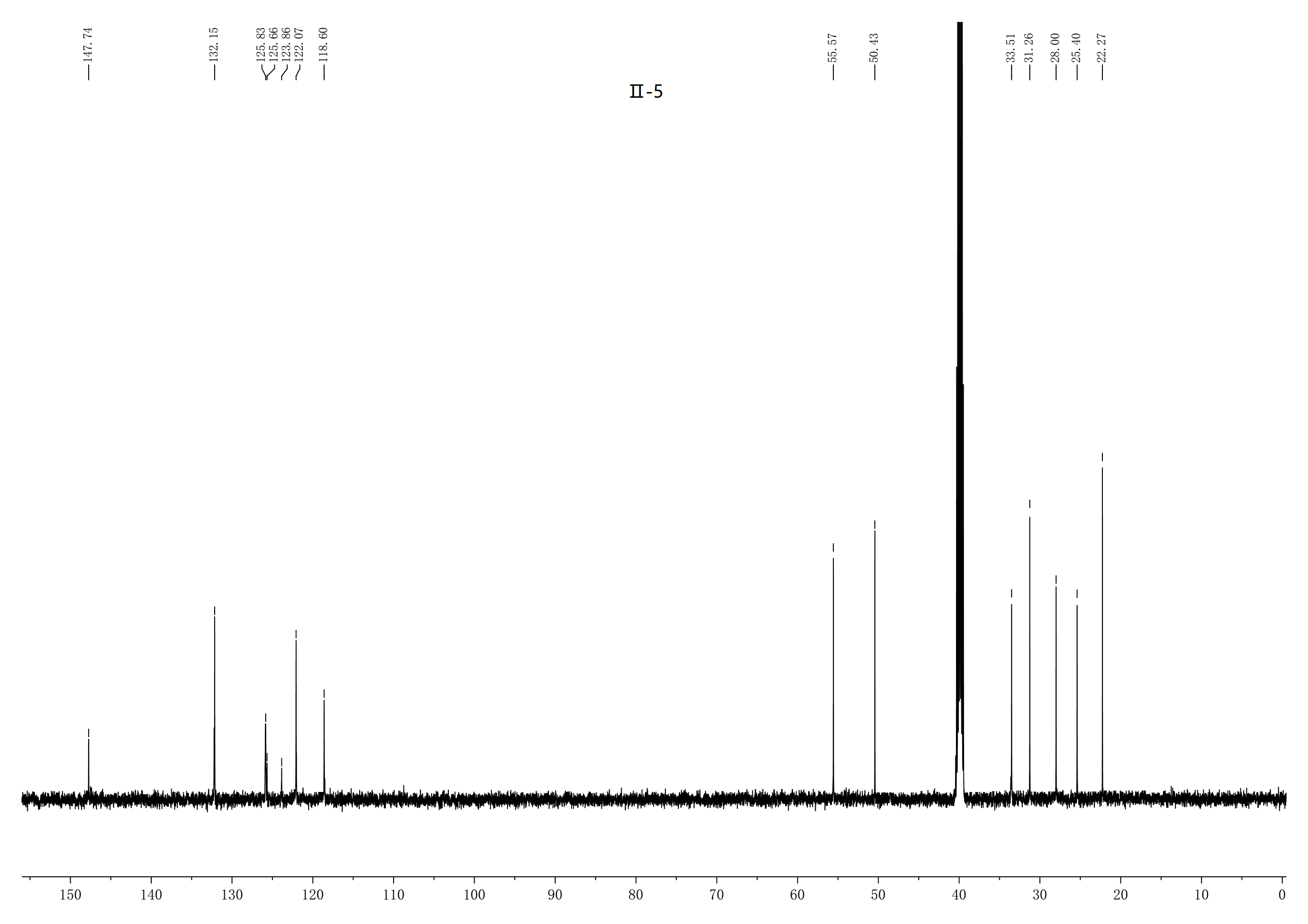


Figure S10-2 ^13^C NMR spectrum of compound **II-5**


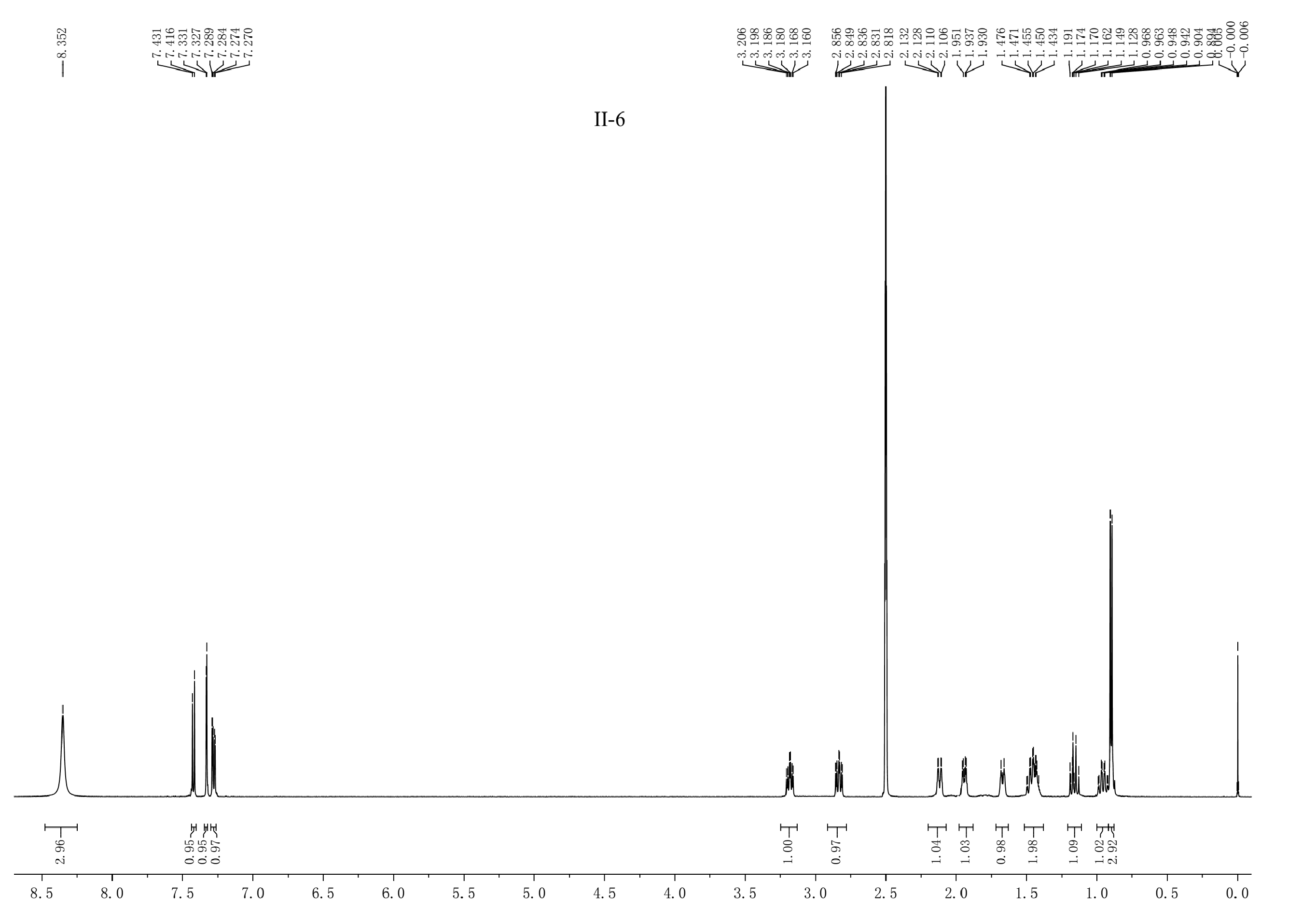


Figure S11-1 ^1^H NMR spectrum of compound **II-6**


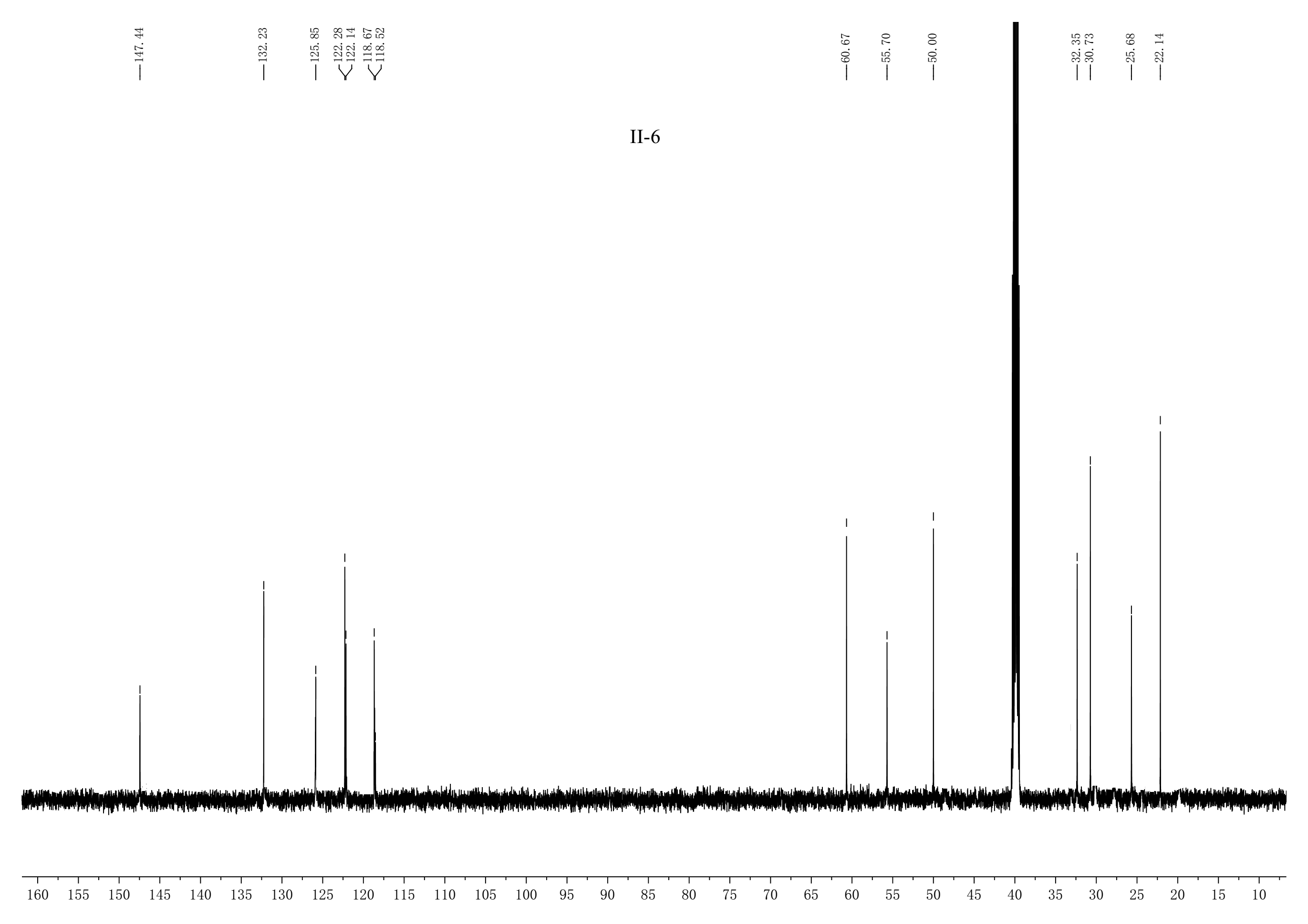


Figure S11-2 ^13^C NMR spectrum of compound **II-6**


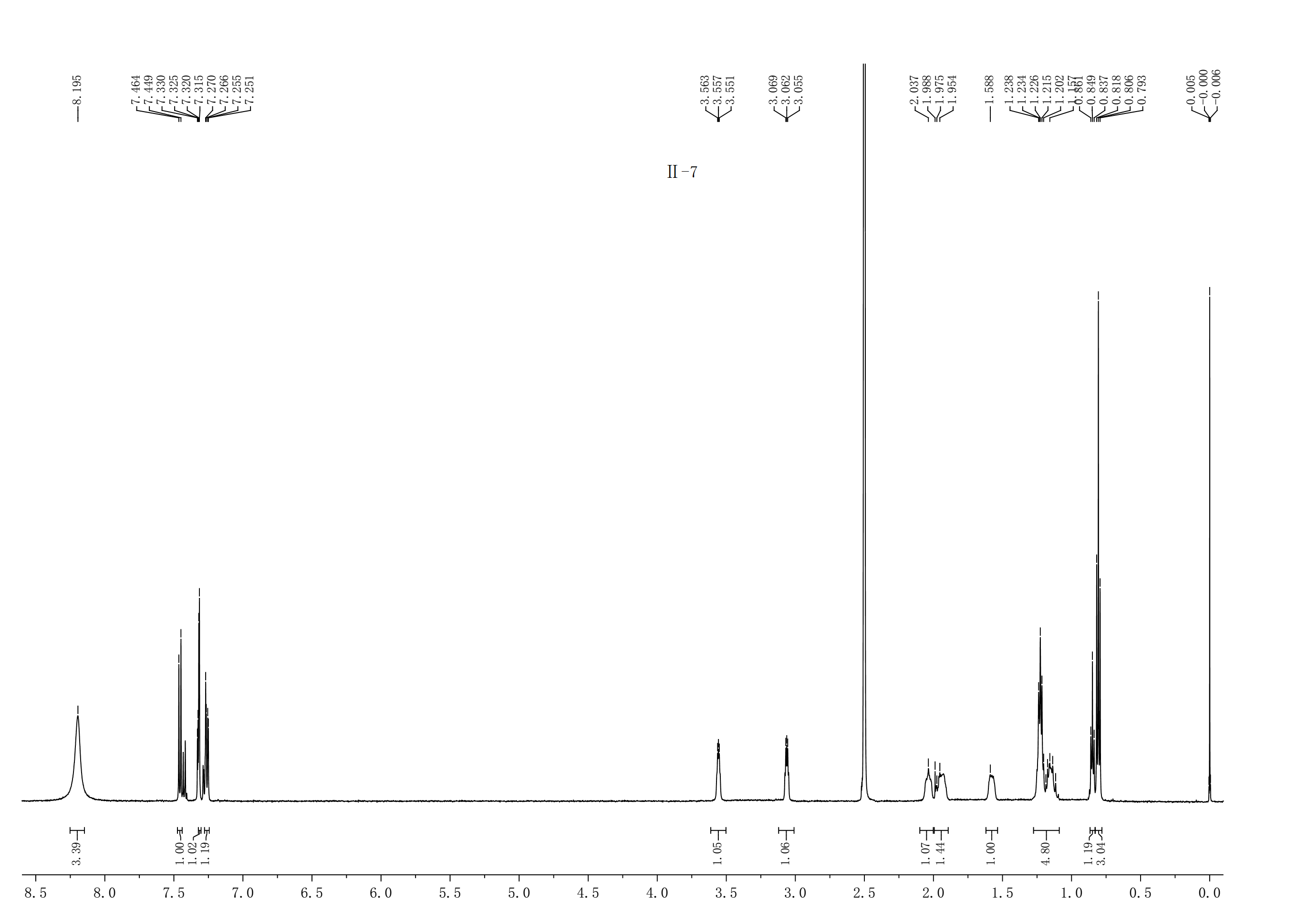


Figure S12-1 ^1^H NMR spectrum of compound **II-7**


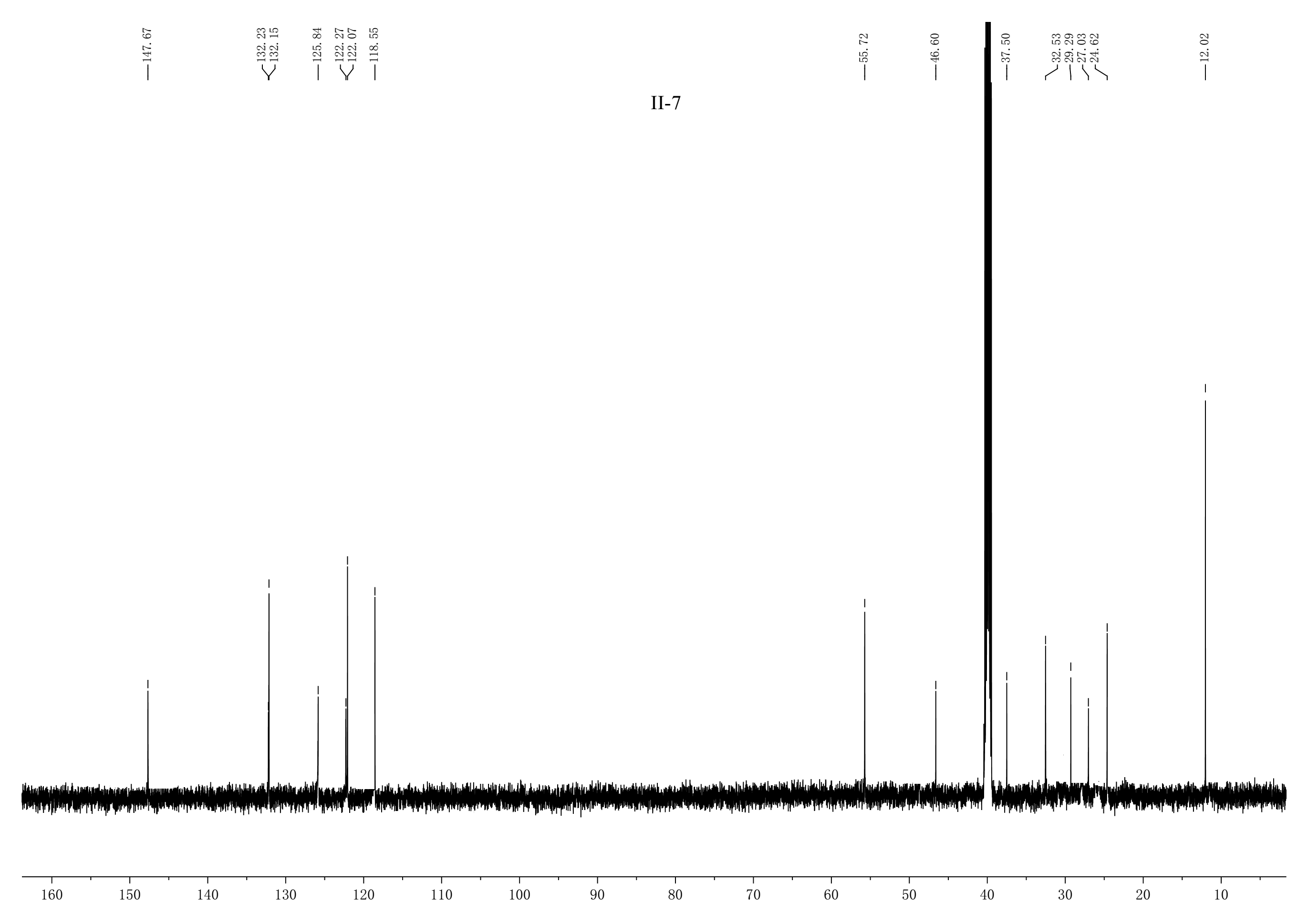


Figure S12-2 ^13^C NMR spectrum of compound **II-7**


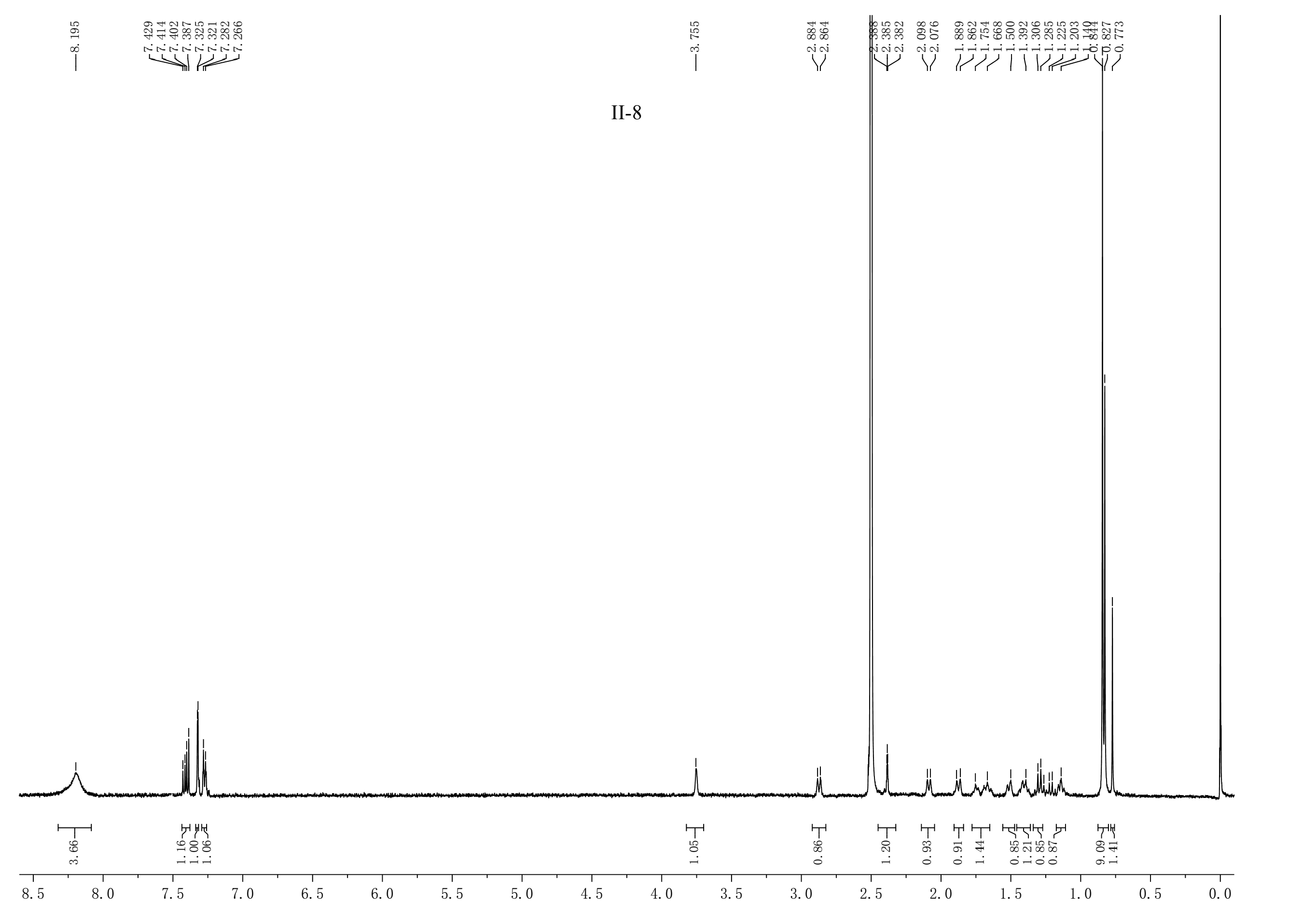


Figure S13-1 ^1^H NMR spectrum of compound **II-8**


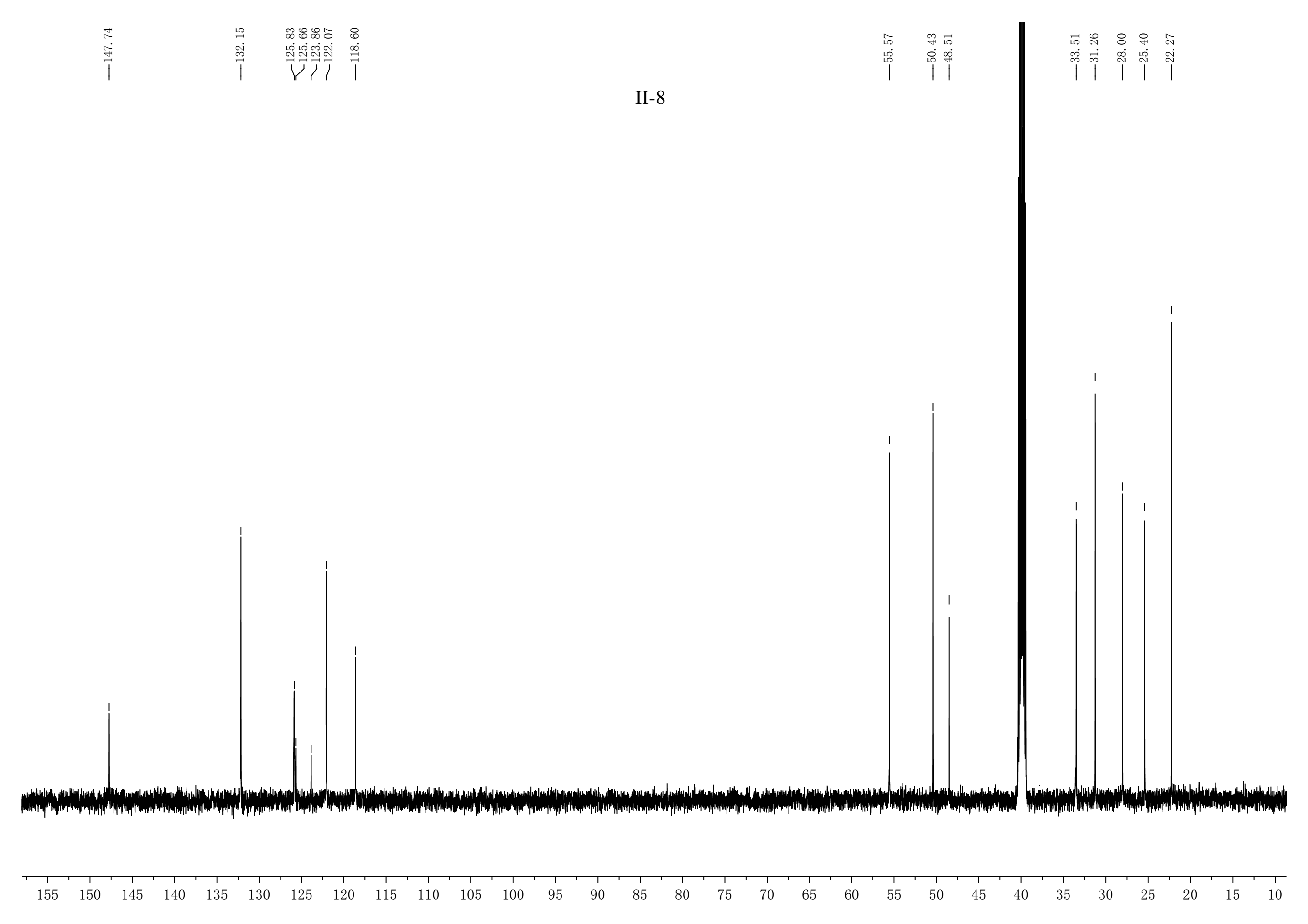


Figure S13-2 ^13^C NMR spectrum of compound **II-8**


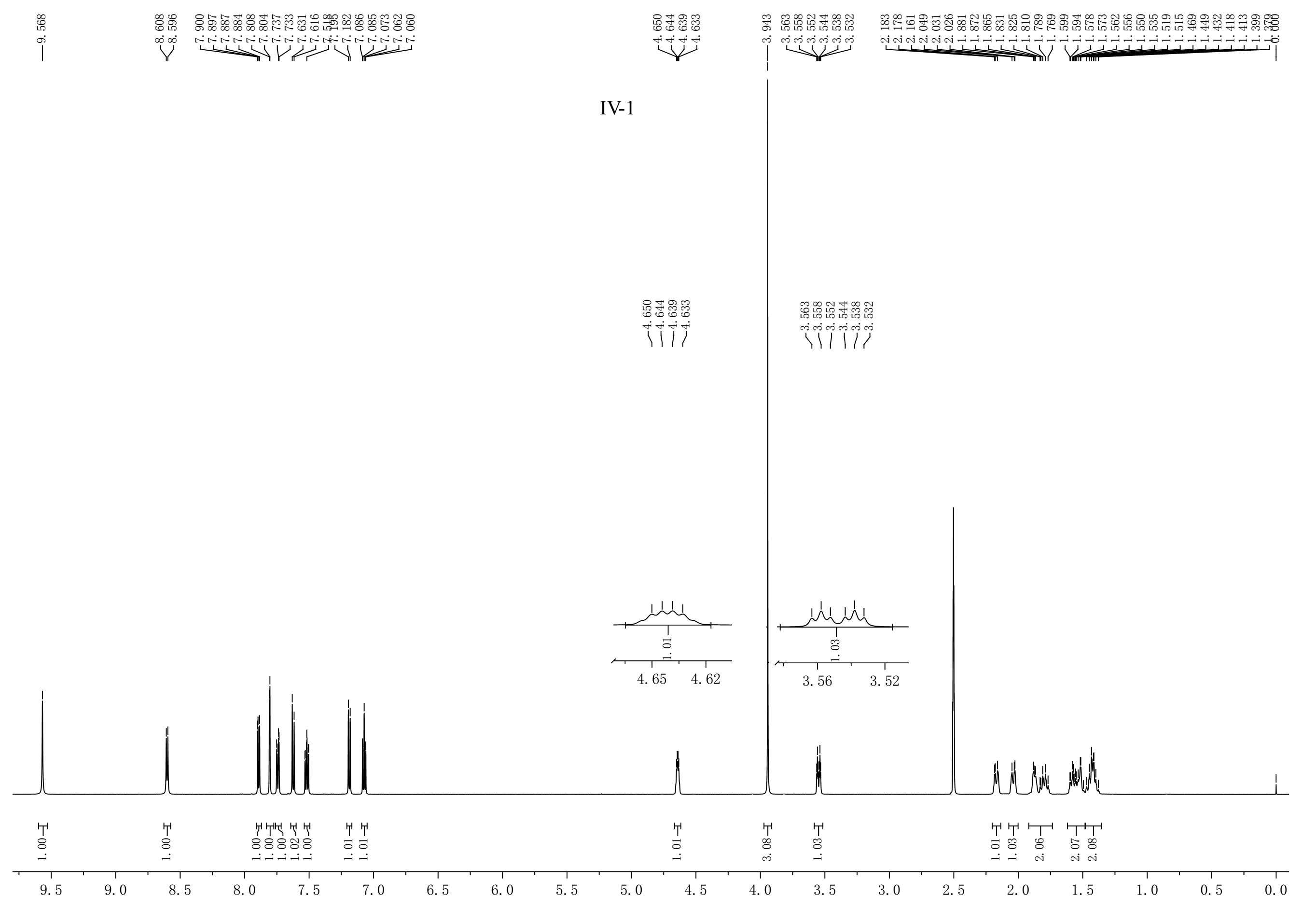


Figure S14-1 ^1^H NMR spectrum of compound **IV-1**


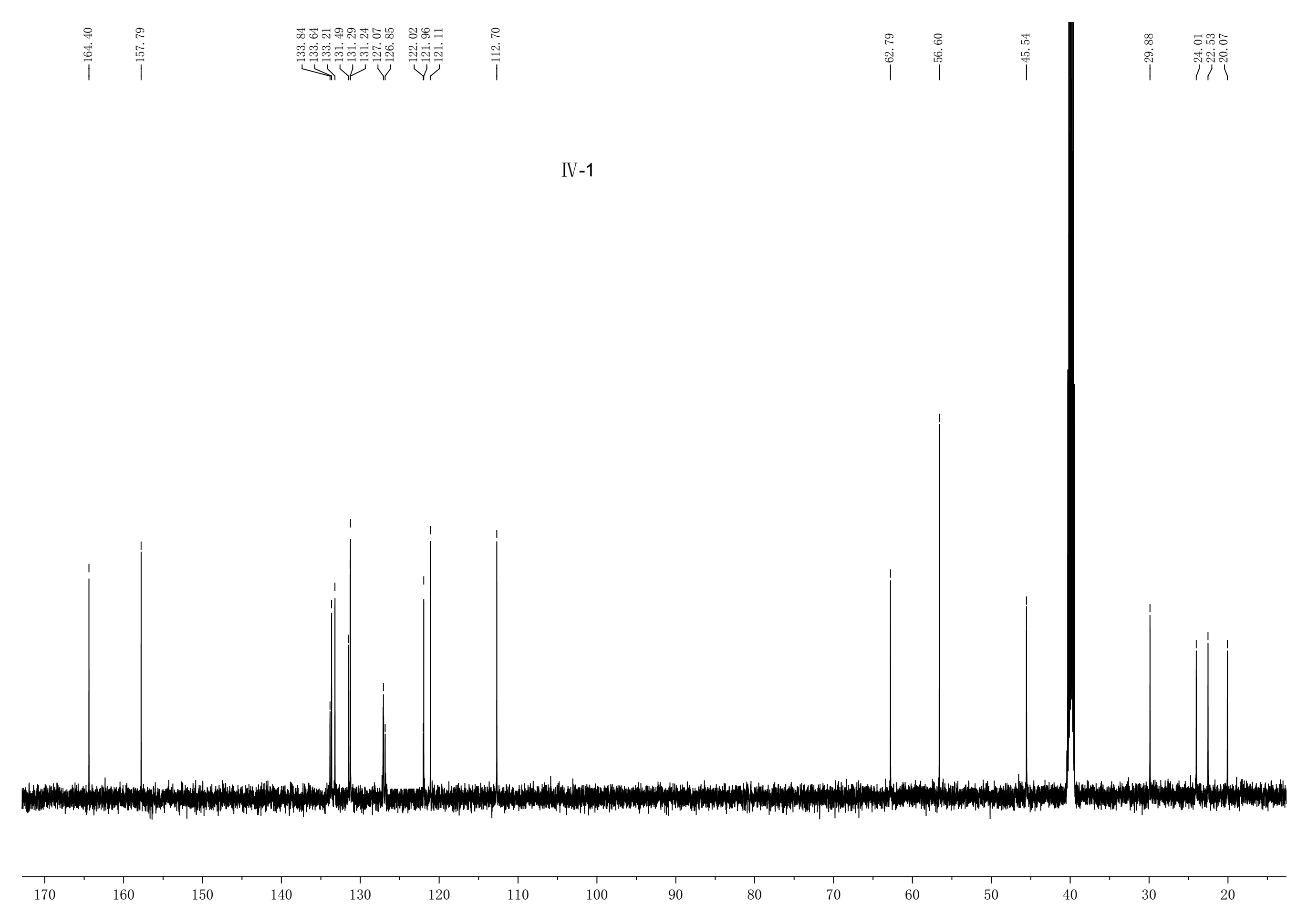


Figure S14-2 ^13^C NMR spectrum of compound **IV-1**


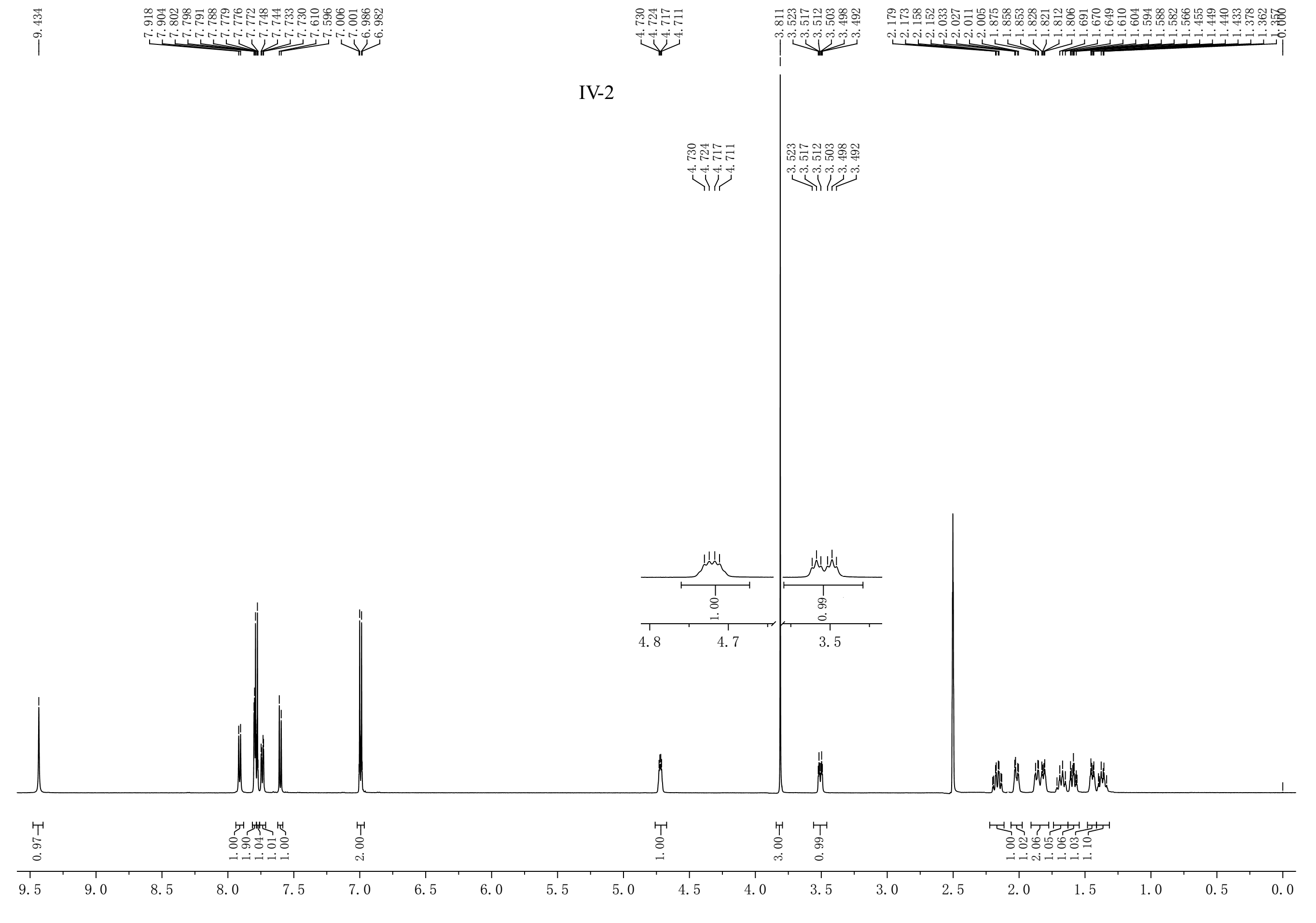


Figure S15-1 ^1^H NMR spectrum of compound **IV-2**


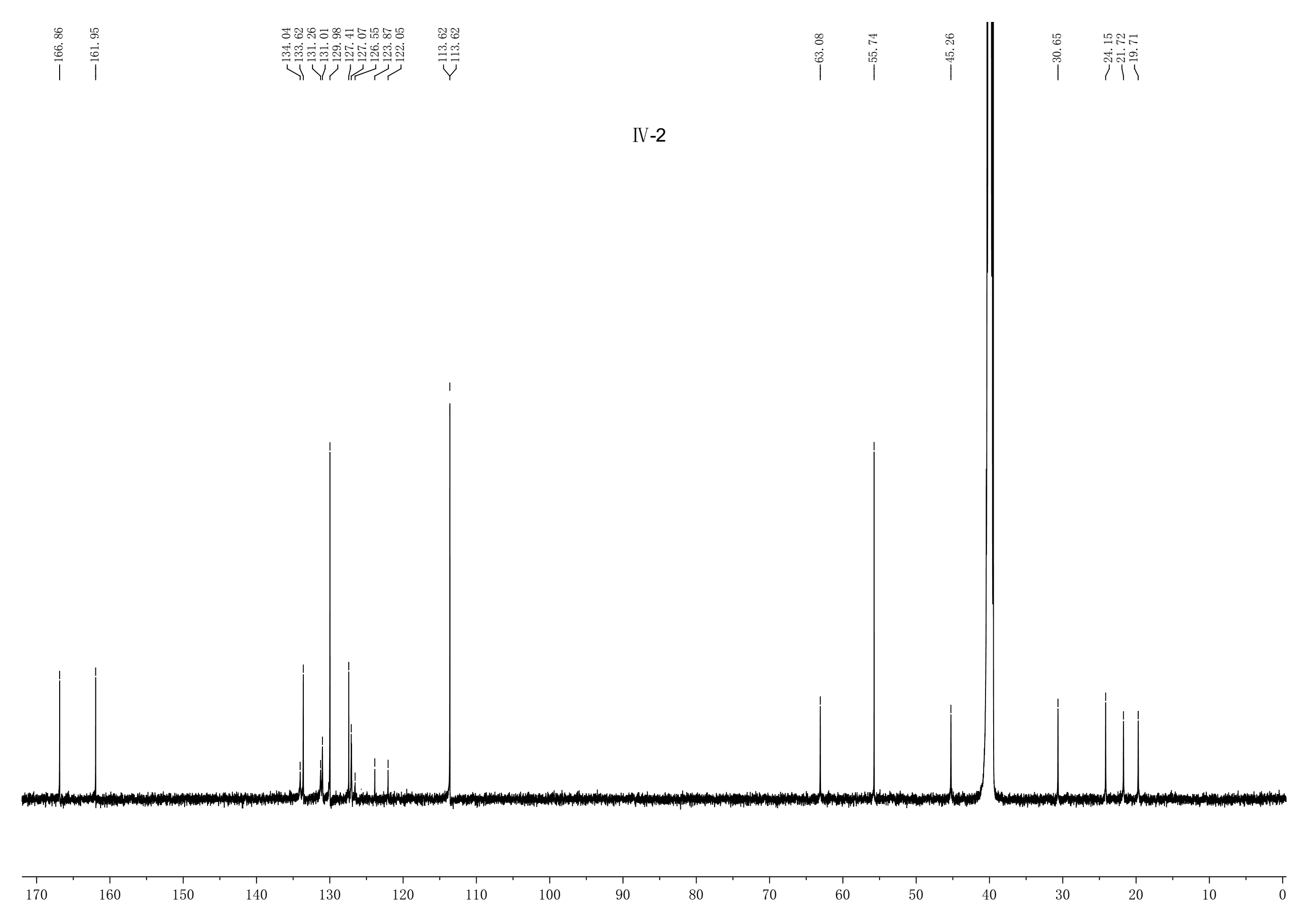


Figure S15-2 ^13^C NMR spectrum of compound **IV-2**


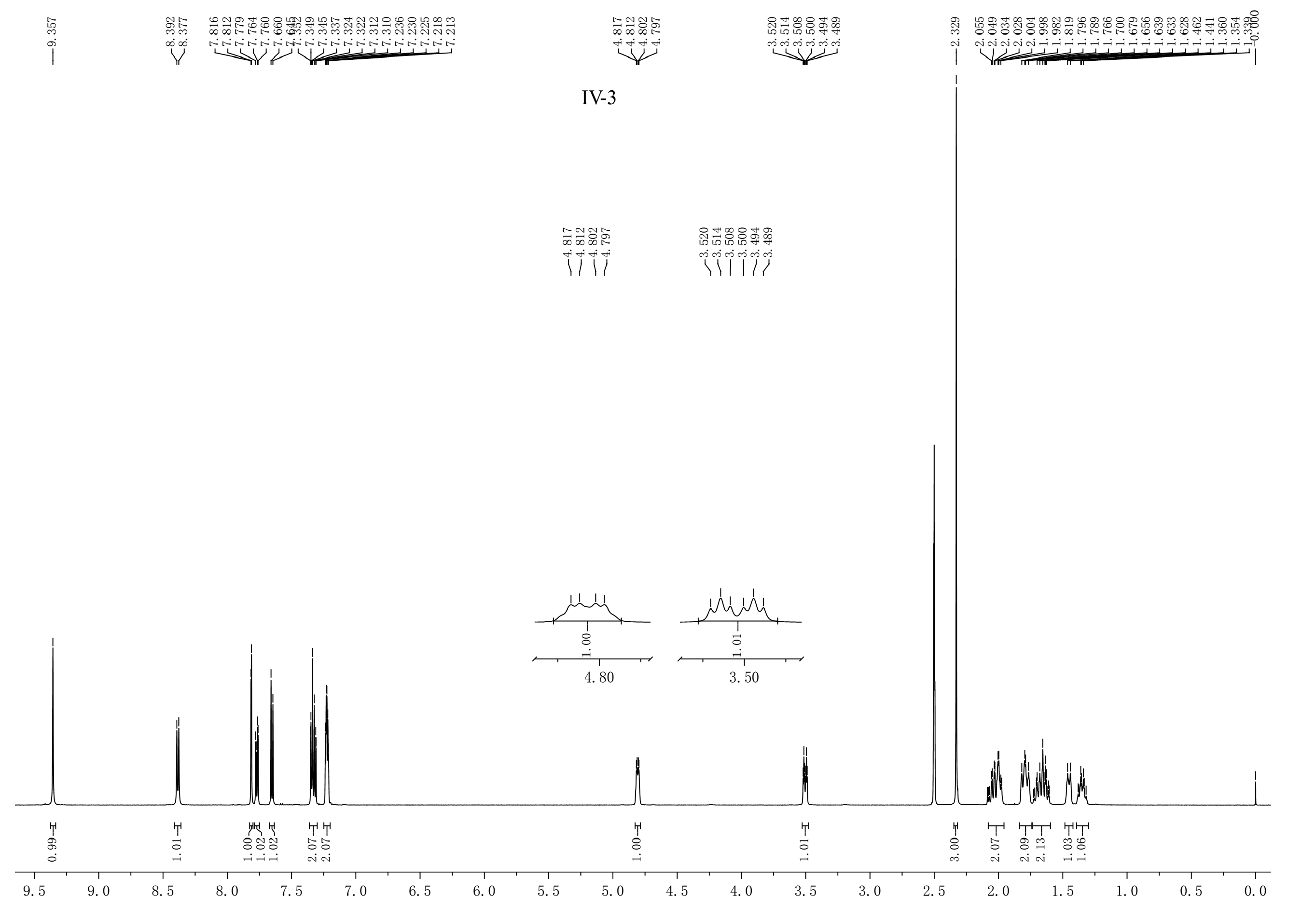


Figure S16-1 ^1^H NMR spectrum of compound **IV-3**


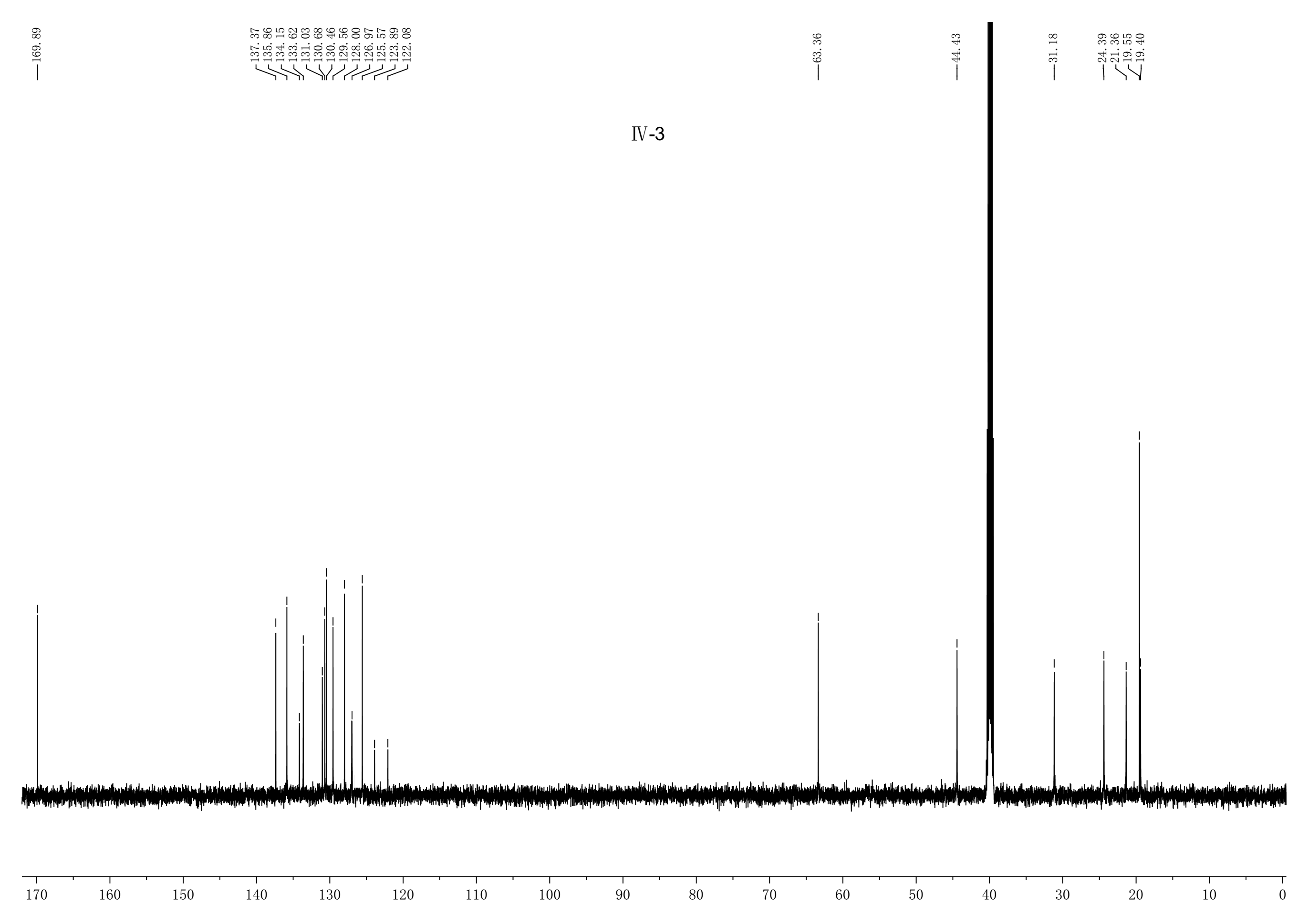


Figure S16-2 ^13^C NMR spectrum of compound **IV-3**


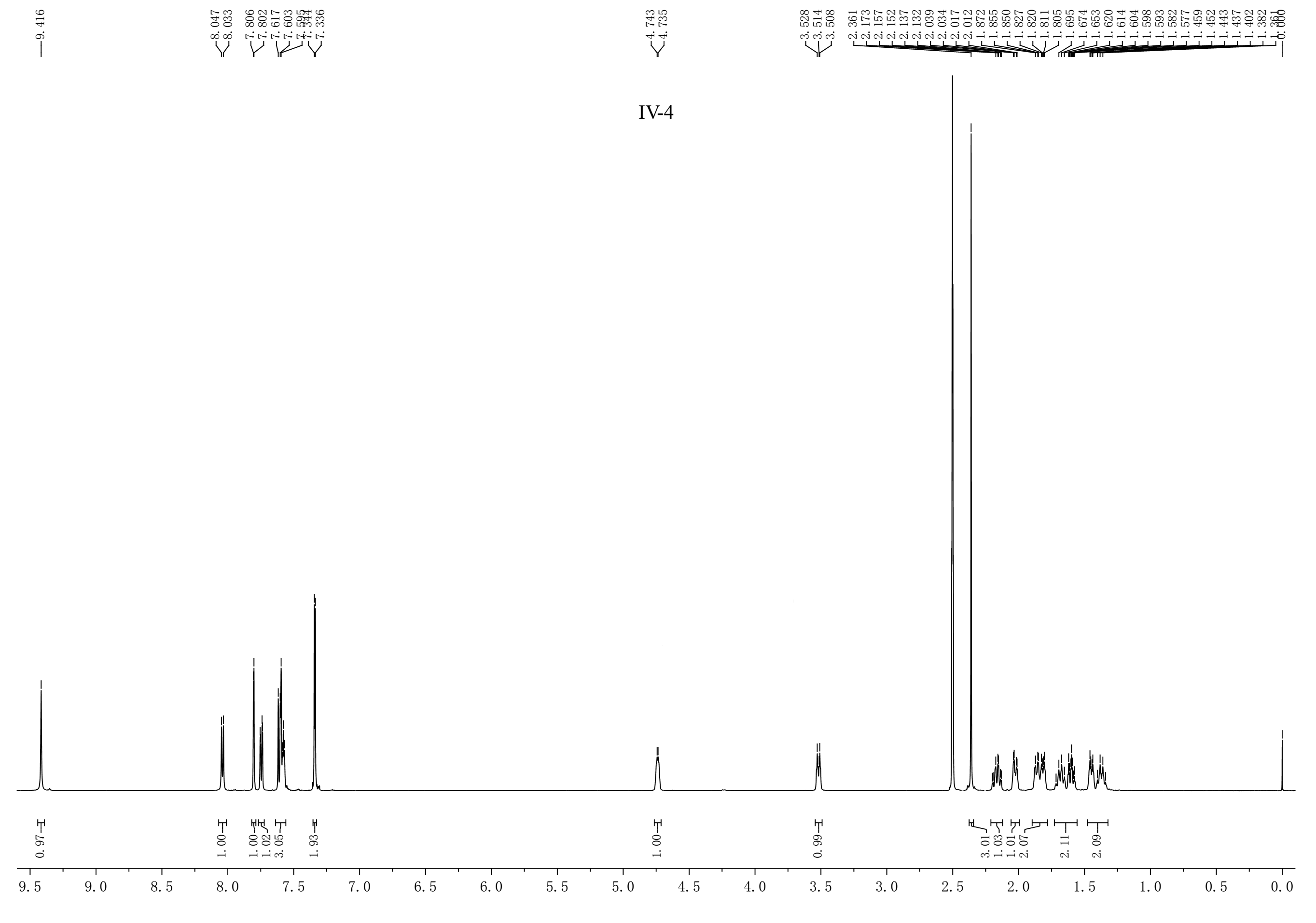


Figure S17-1 ^1^H NMR spectrum of compound **IV-4**


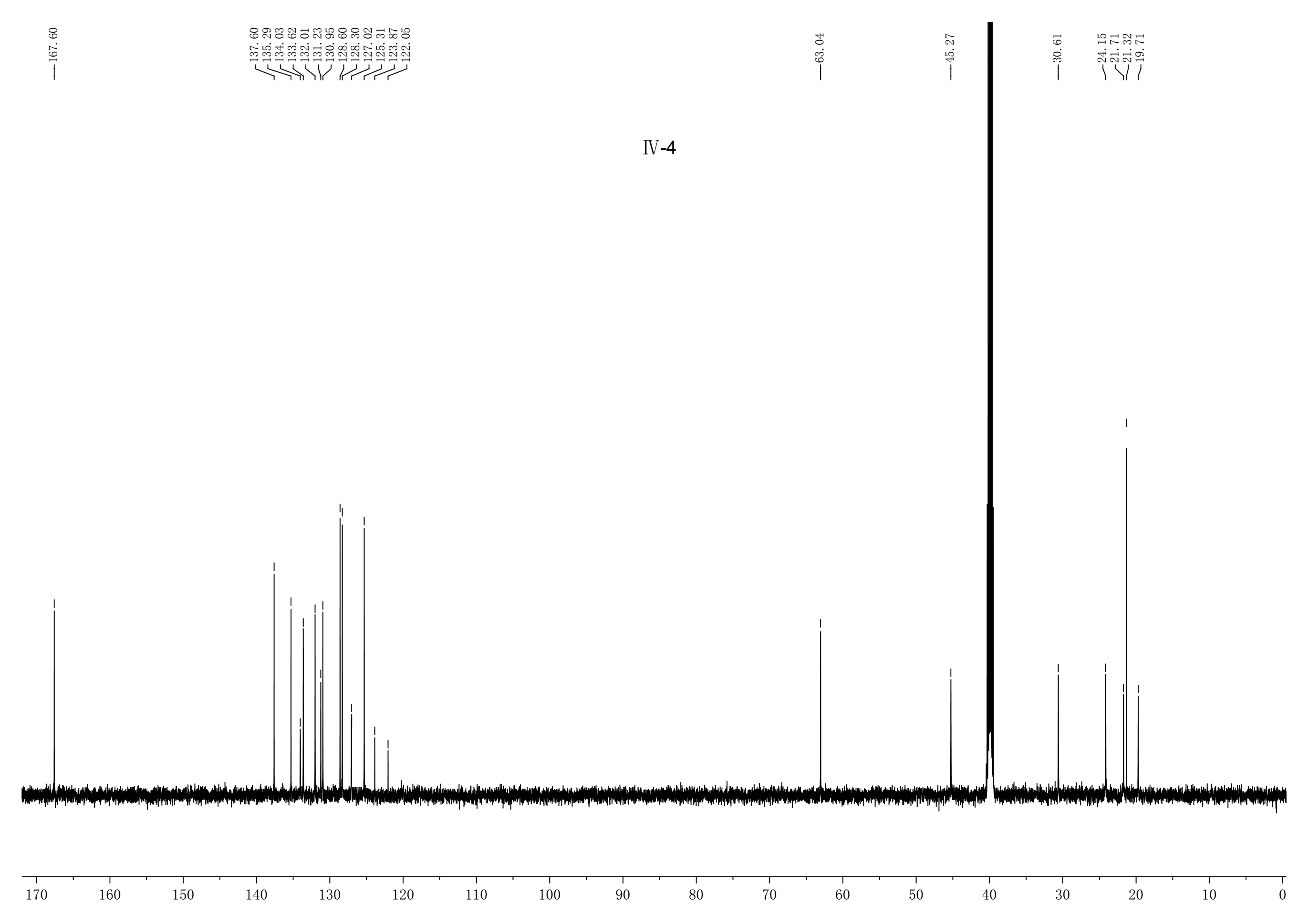


Figure S17-2 ^13^C NMR spectrum of compound **IV-4**


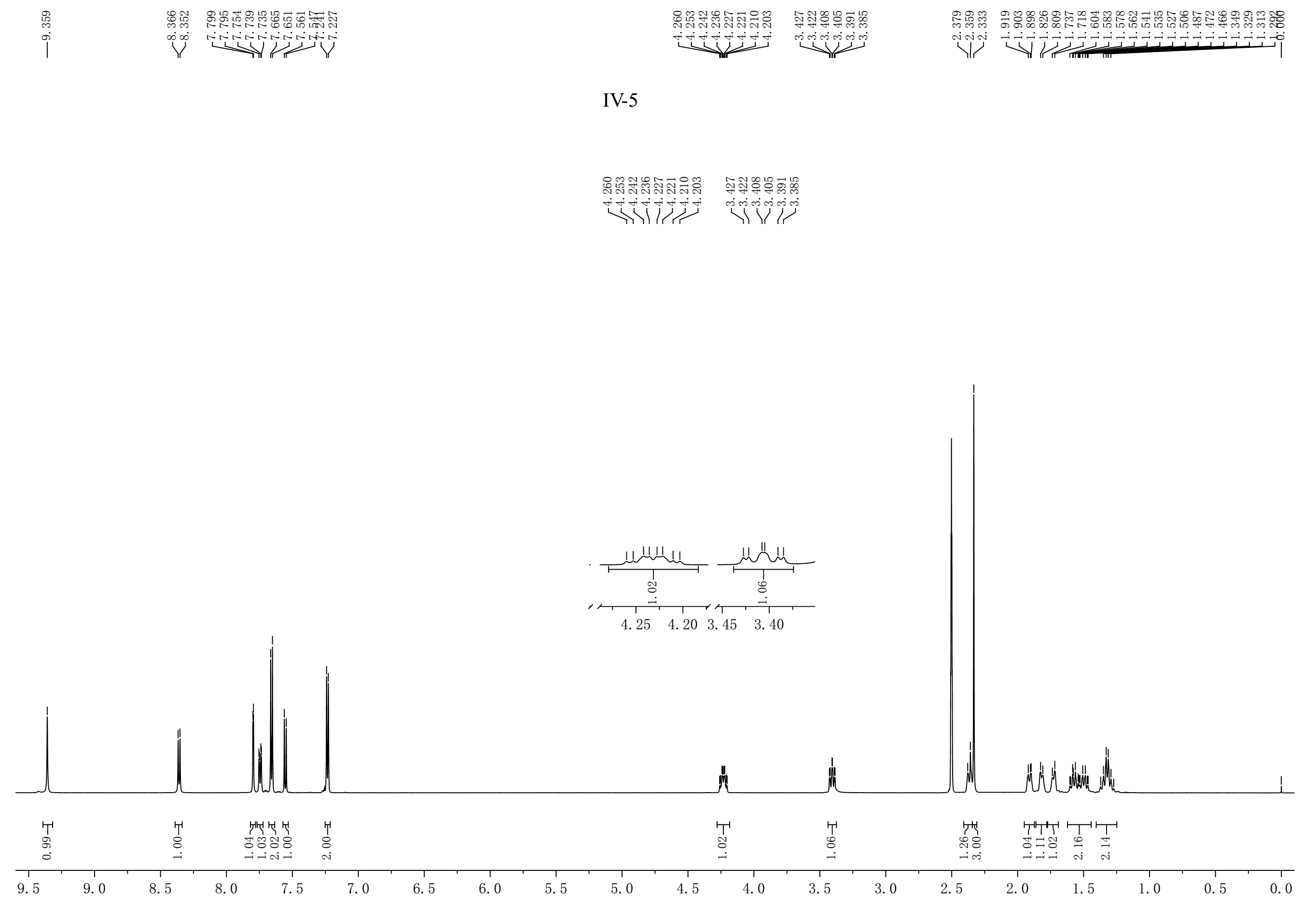


Figure S18-1 ^1^H NMR spectrum of compound **IV-5**

**
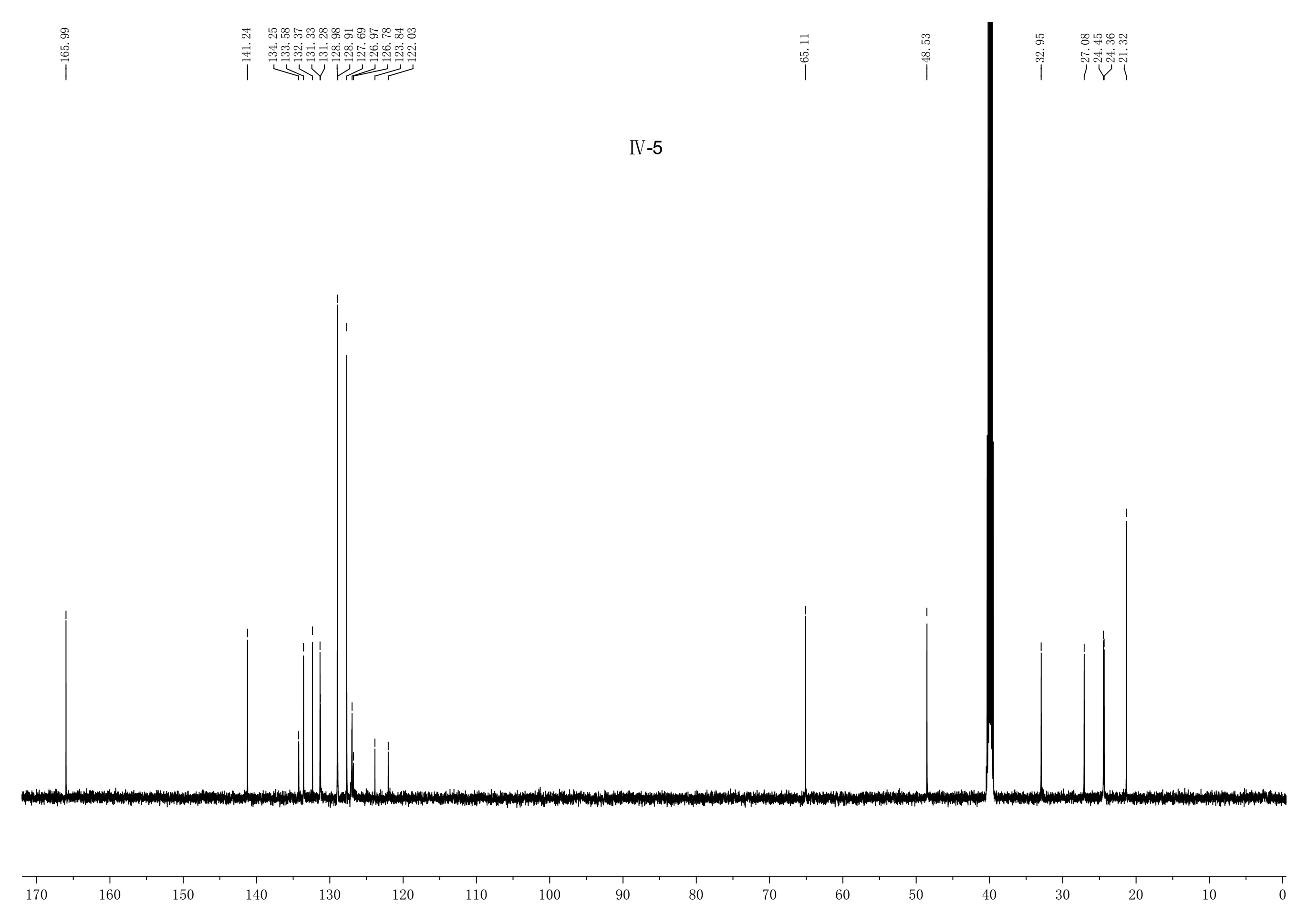
**

Figure S18-2 ^13^C NMR spectrum of compound **IV-5**


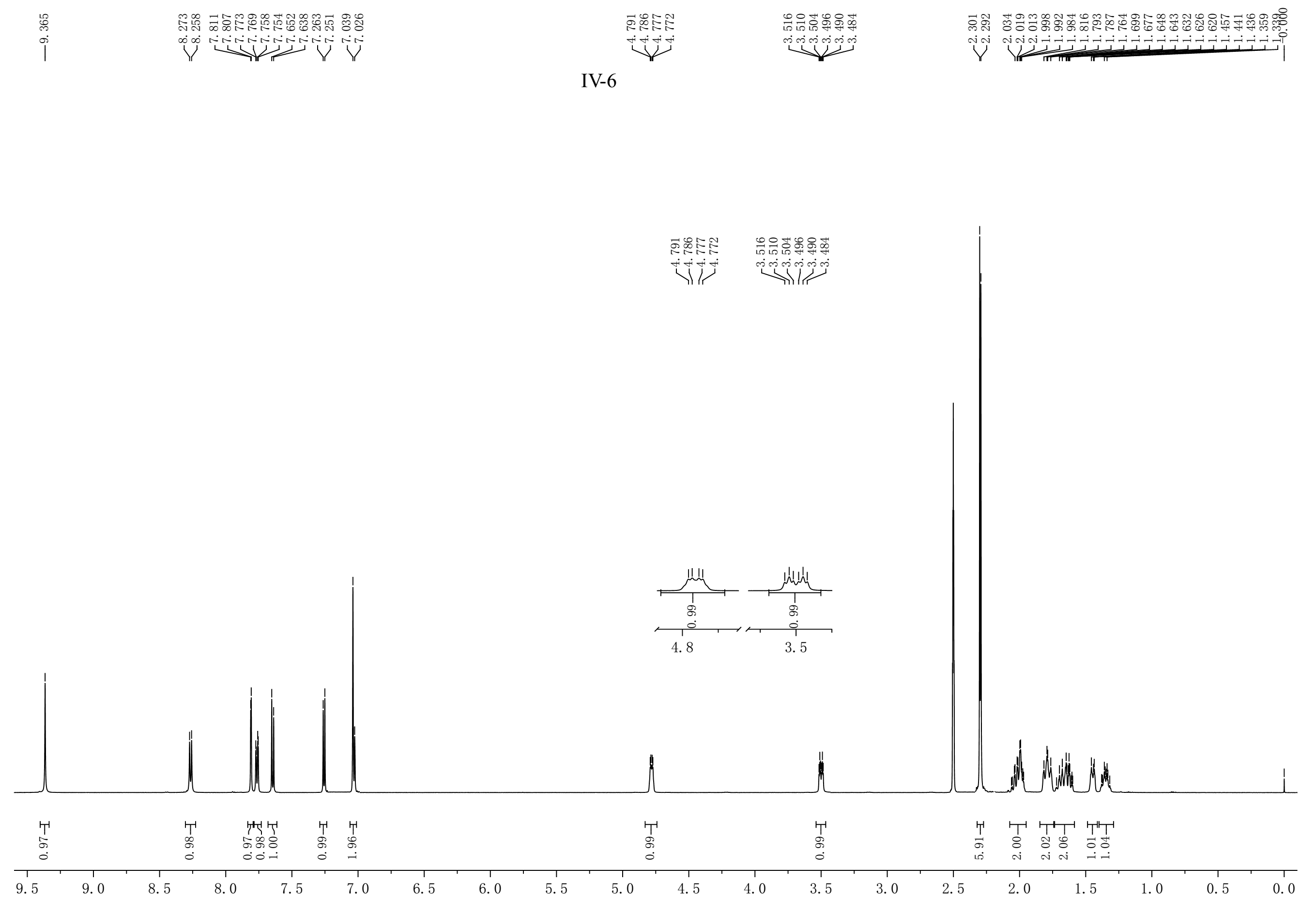


Figure S19-1 ^1^H NMR spectrum of compound **IV-6**


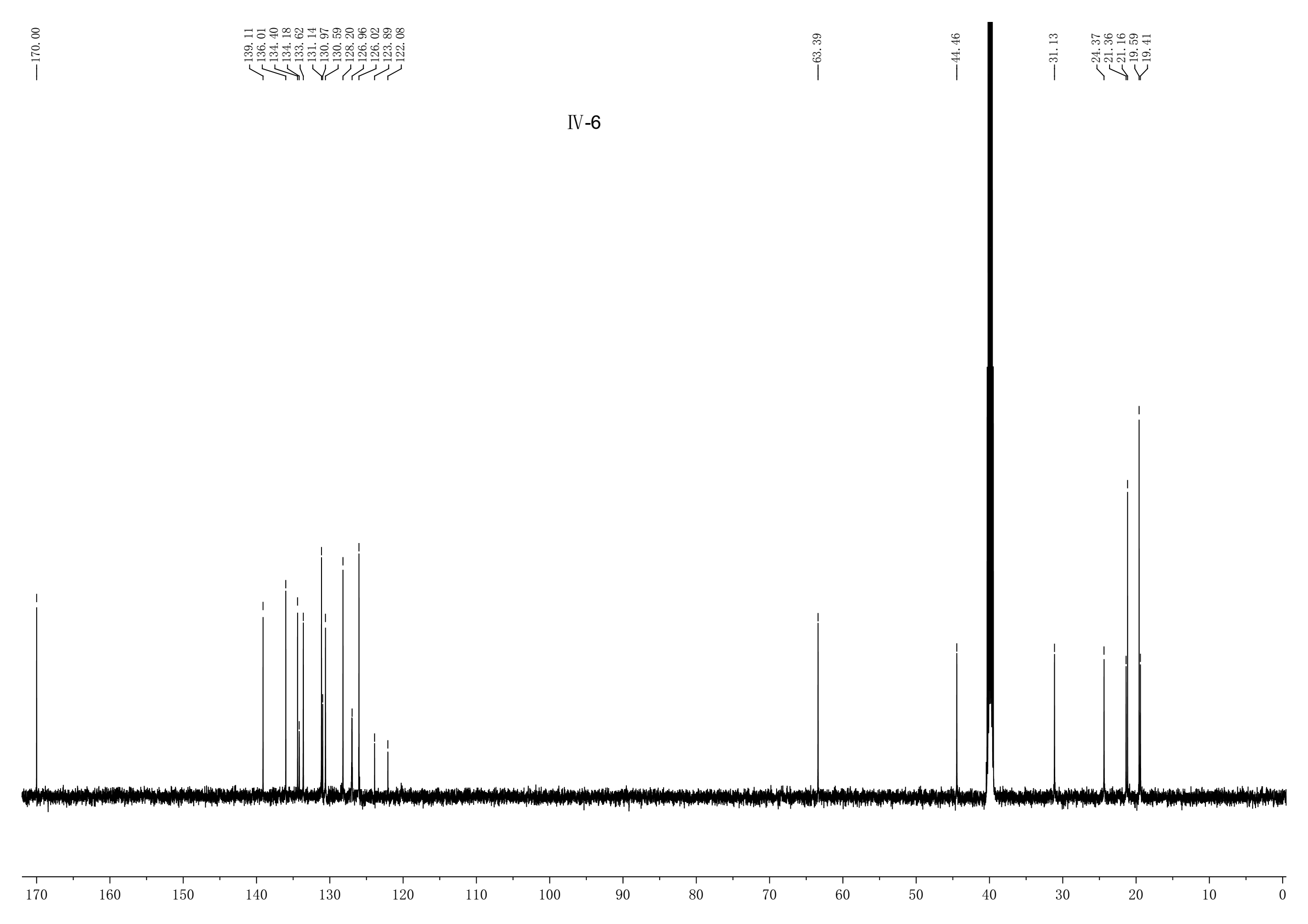


Figure S19-2 ^13^C NMR spectrum of compound **IV-6**


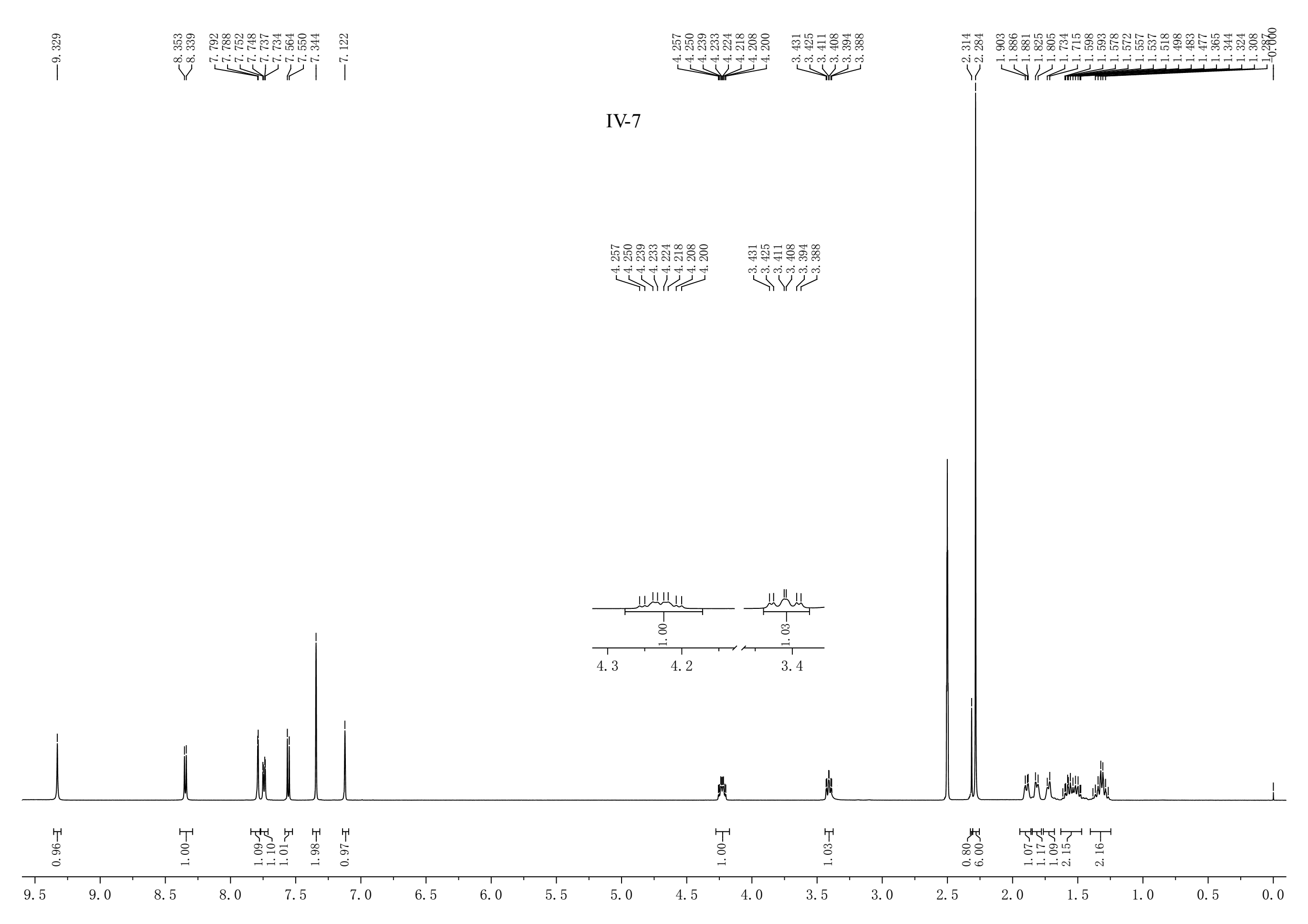


Figure S20-1 ^1^H NMR spectrum of compound **IV-7**


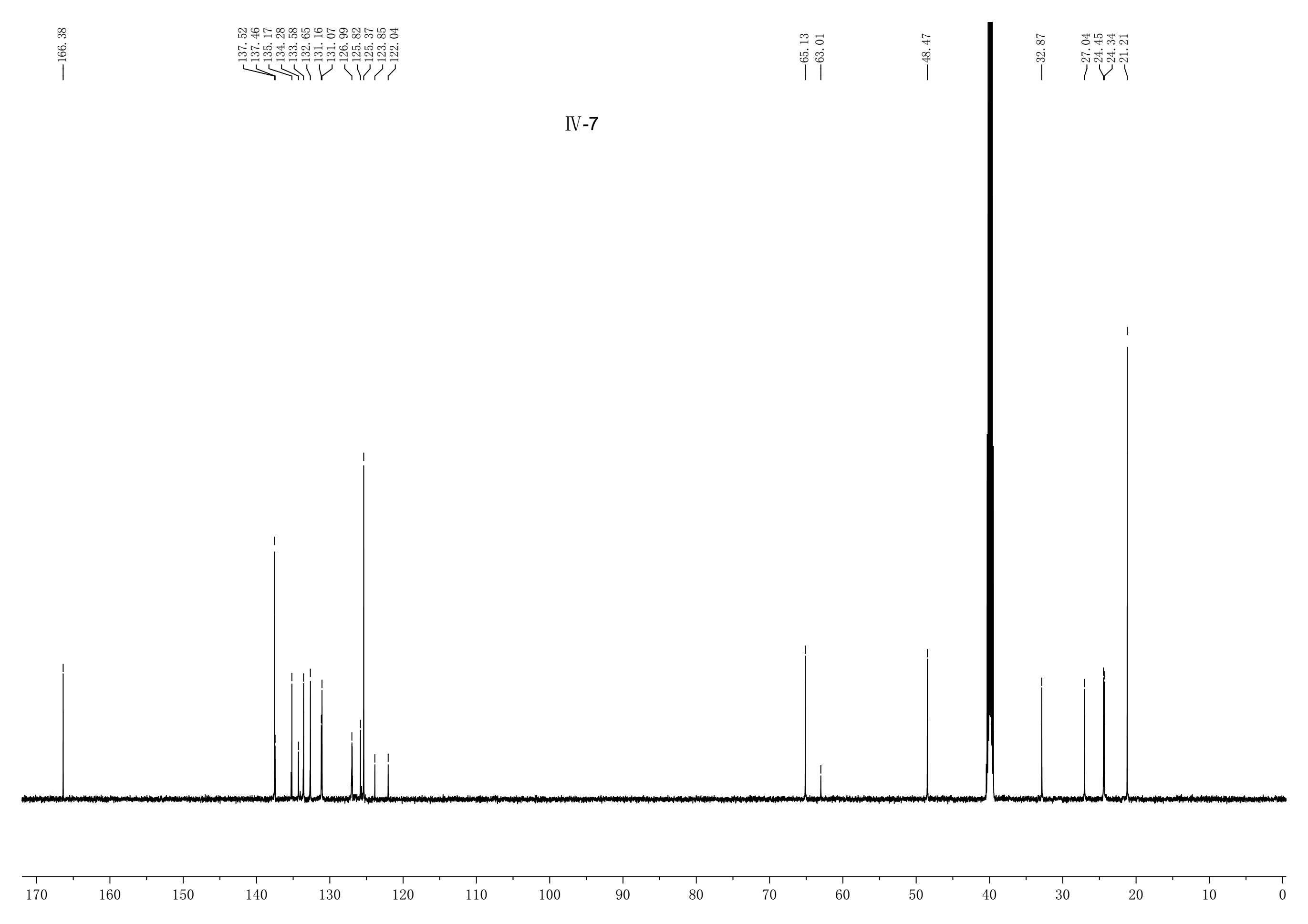


Figure S20-2 ^13^C NMR spectrum of compound **IV-7**


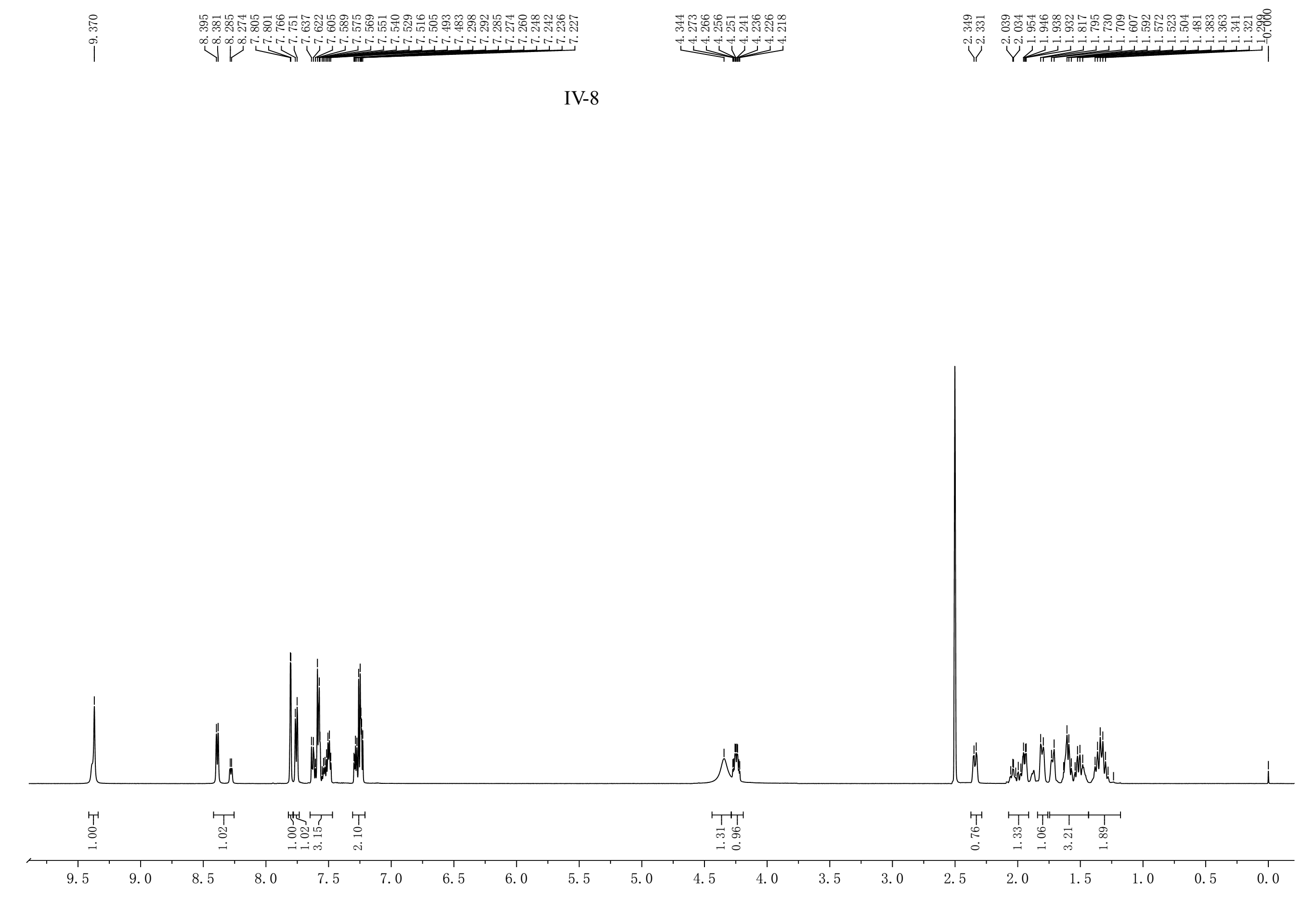


Figure S21-1 ^1^H NMR spectrum of compound **IV-8**


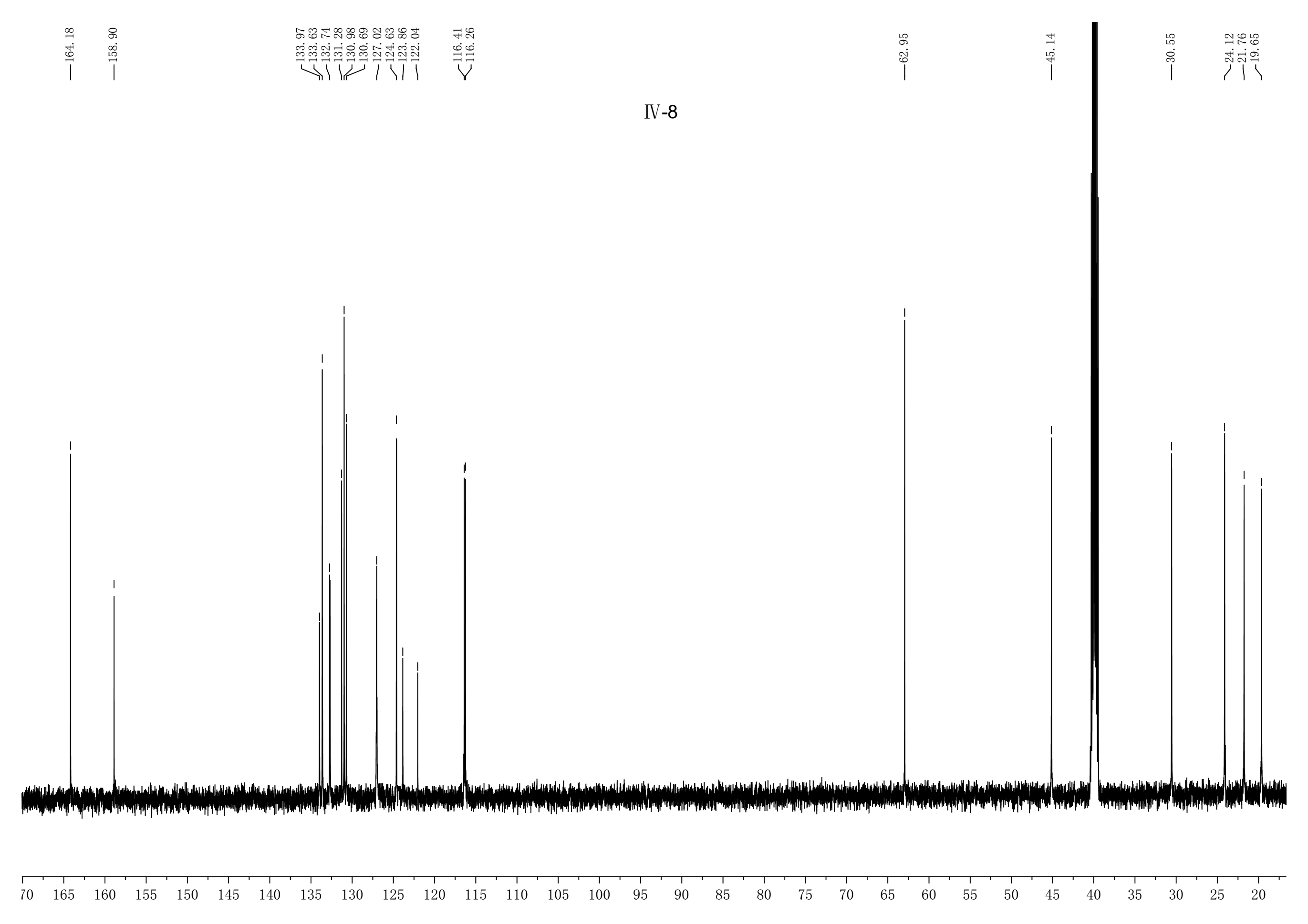


Figure S21-2 ^13^C NMR spectrum of compound **IV-8**


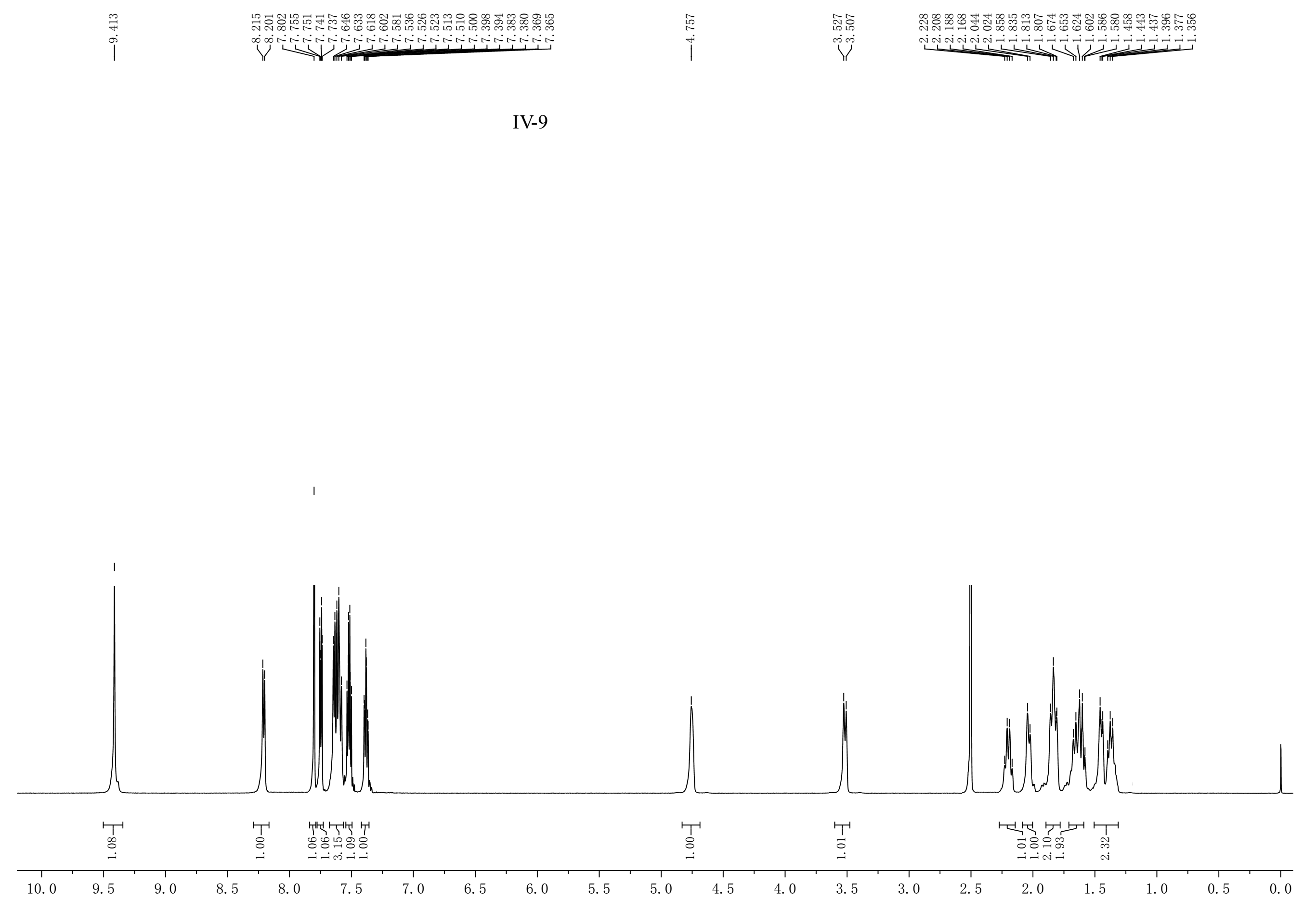


Figure S22-1 ^1^H NMR spectrum of compound **IV-9**


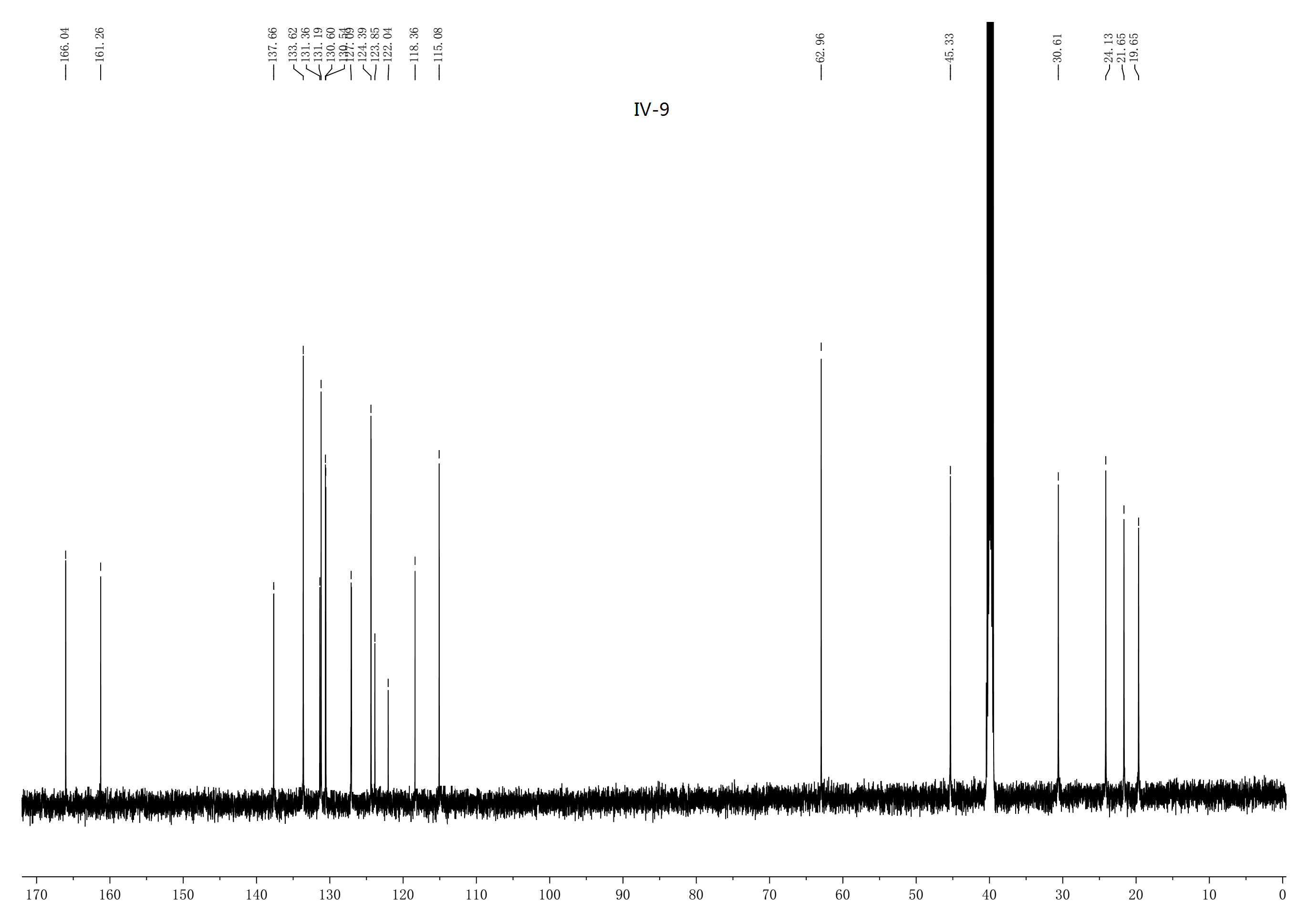


Figure S22-2 ^13^C NMR spectrum of compound **IV-9**


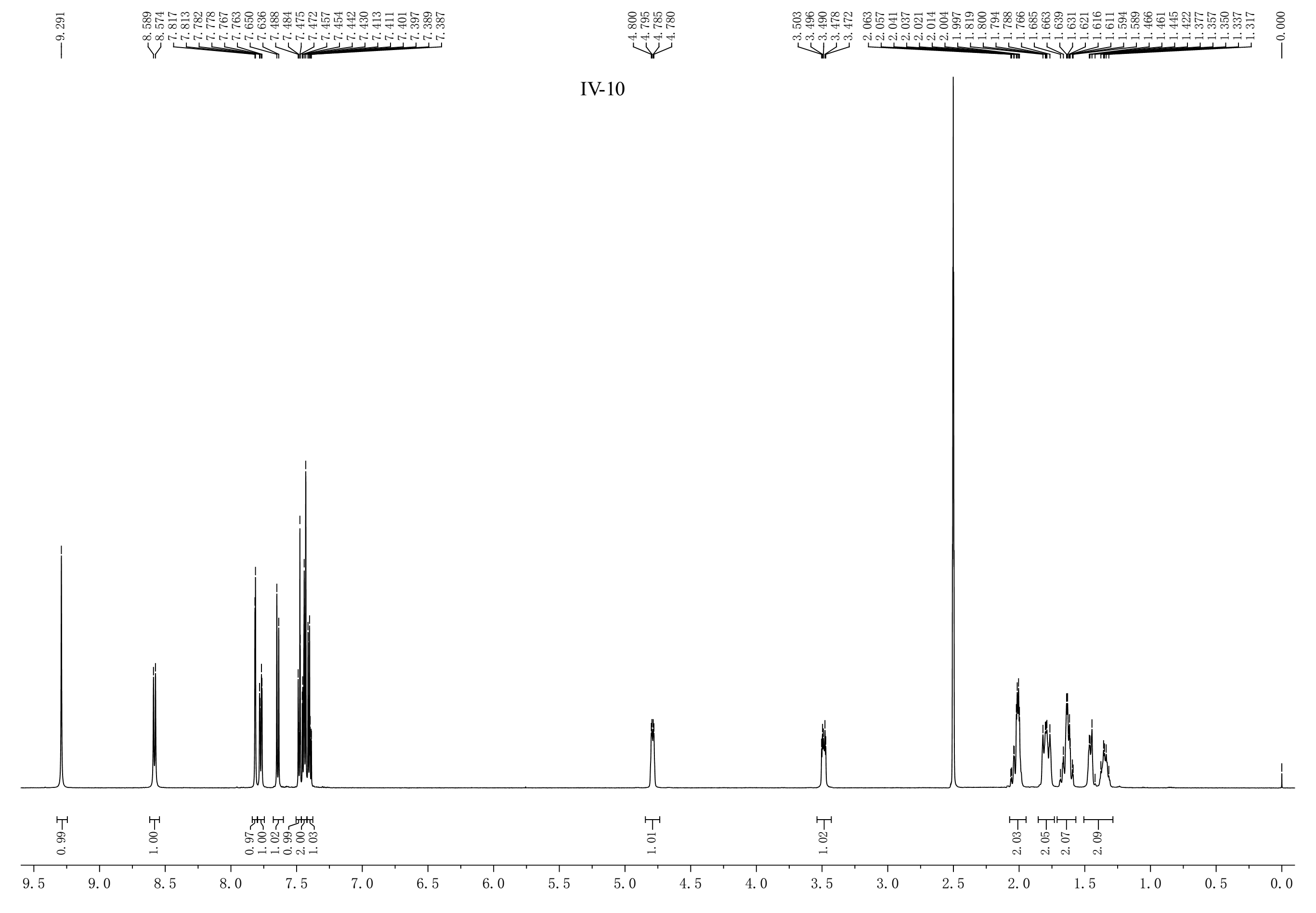


Figure S23-1 ^1^H NMR spectrum of compound **IV-10**


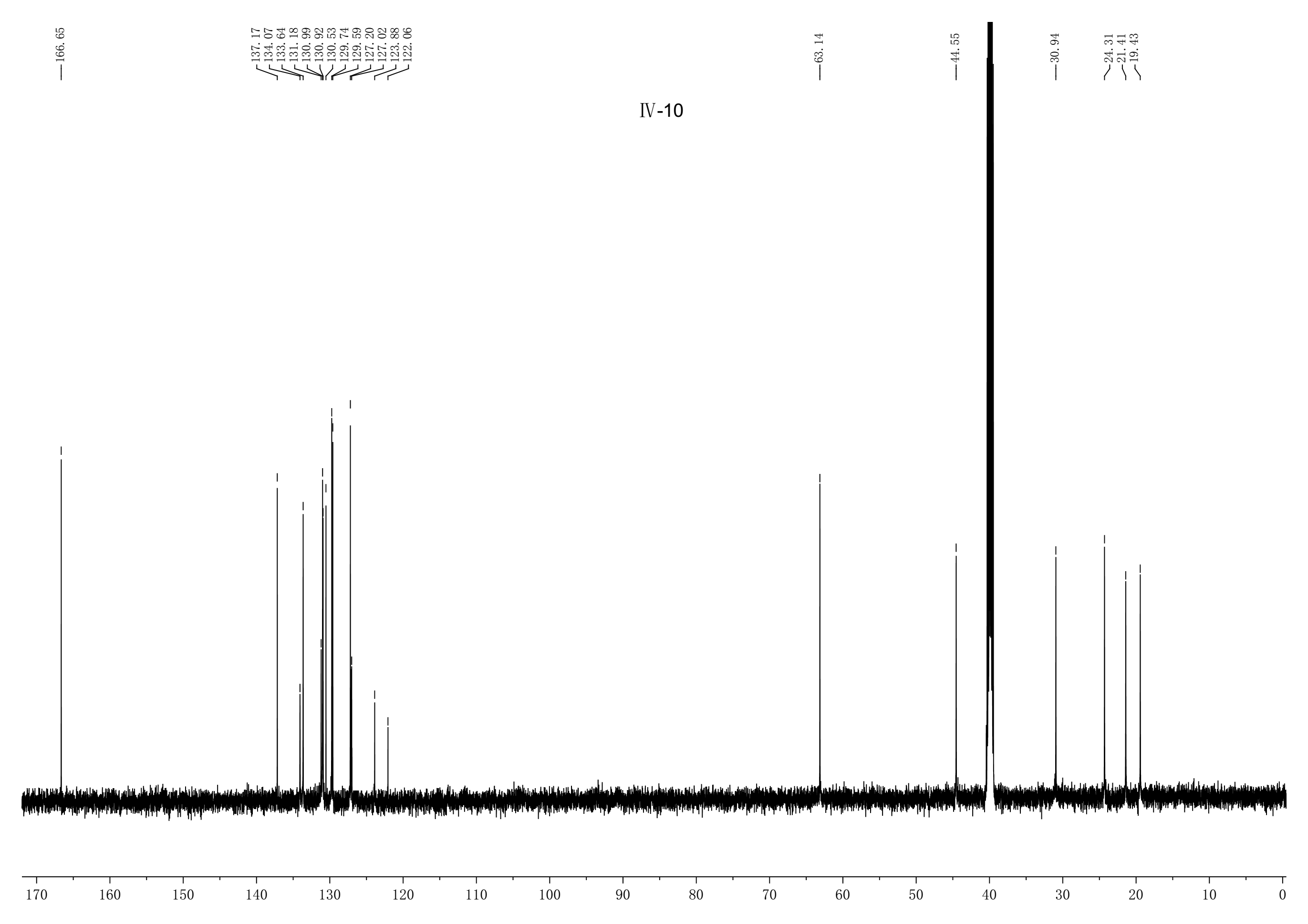


Figure S23-2 ^13^C NMR spectrum of compound **IV-10**


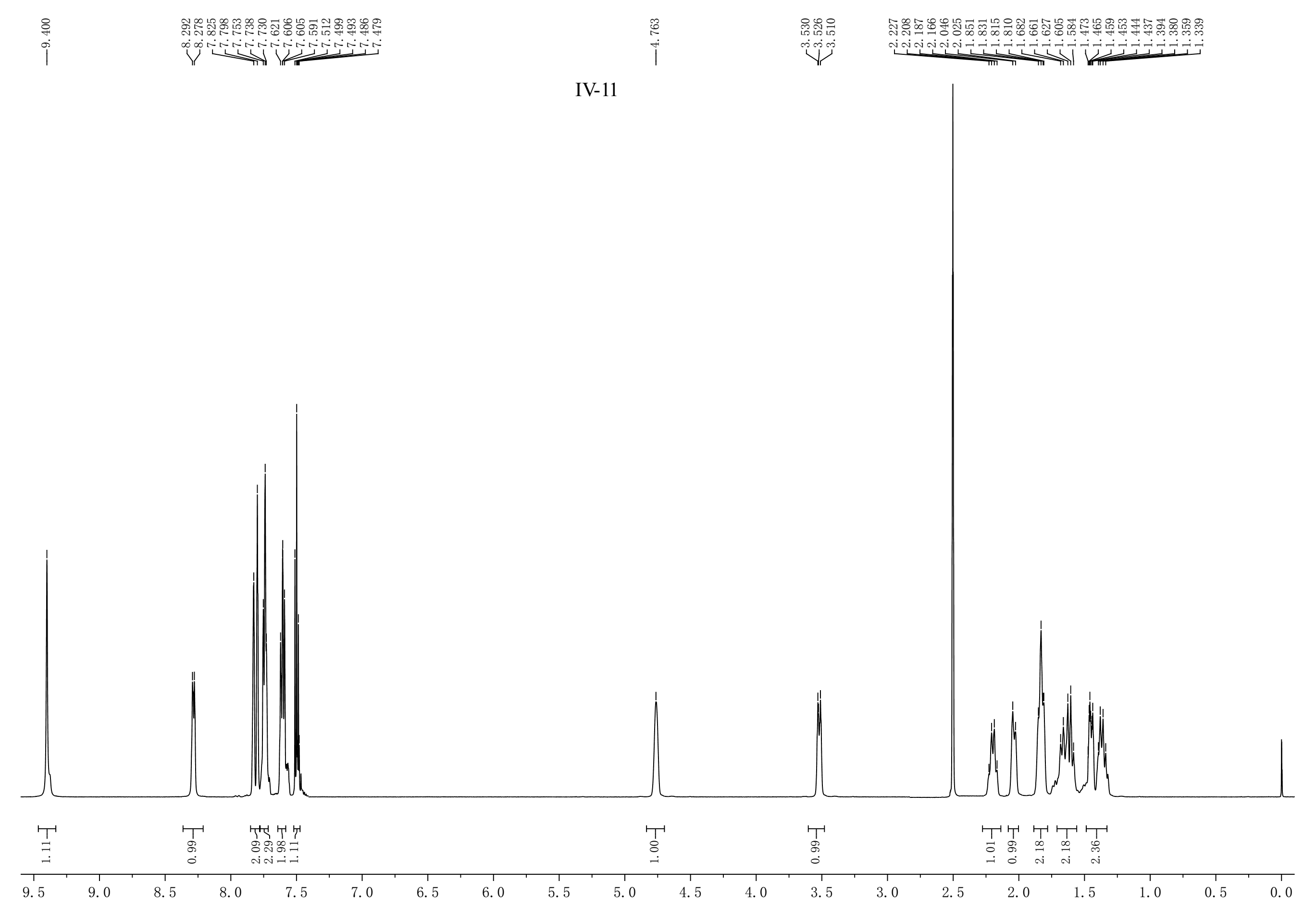


Figure S24-1 ^1^H NMR spectrum of compound **IV-11**


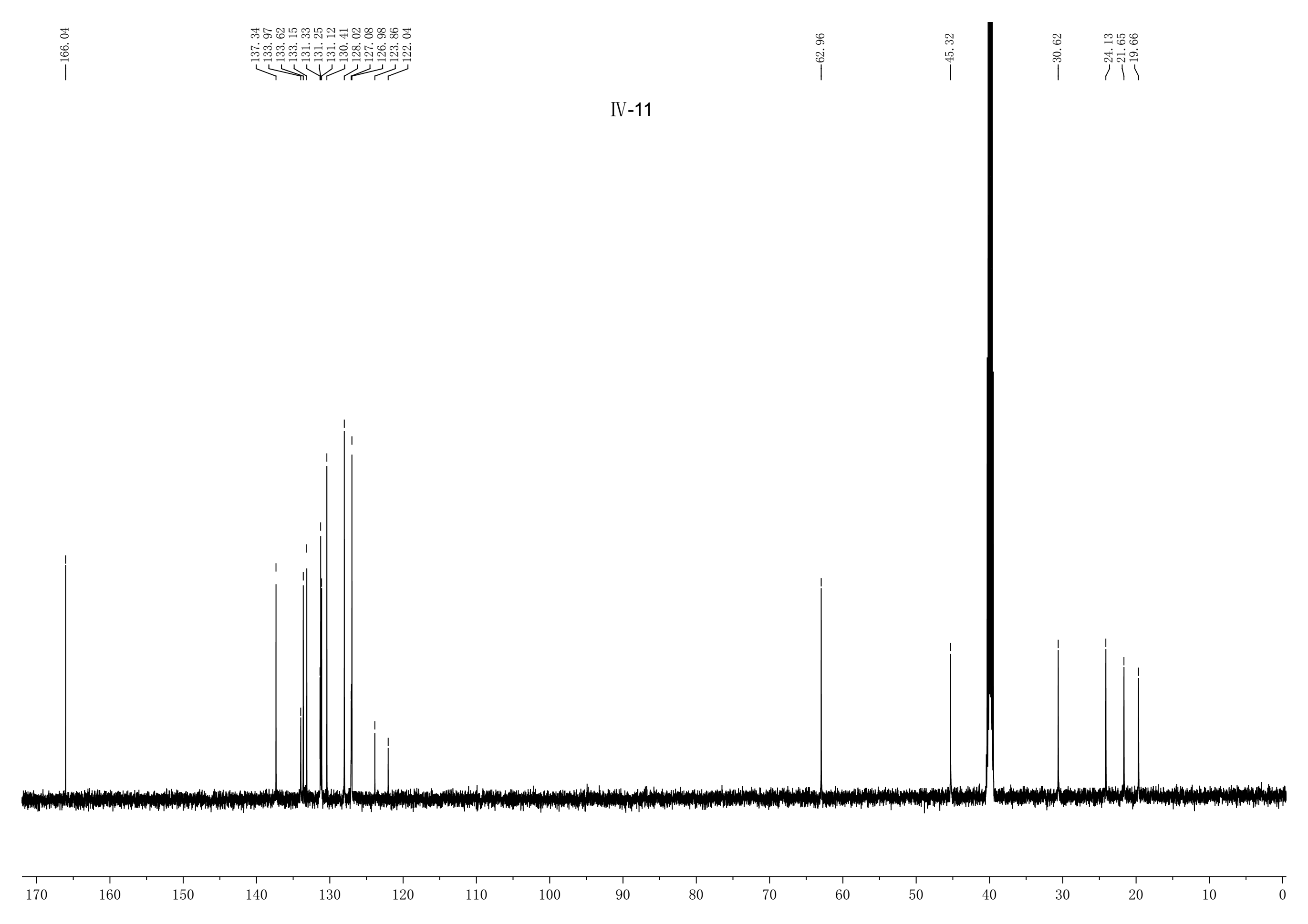


Figure S24-2 ^13^C NMR spectrum of compound **IV-11**


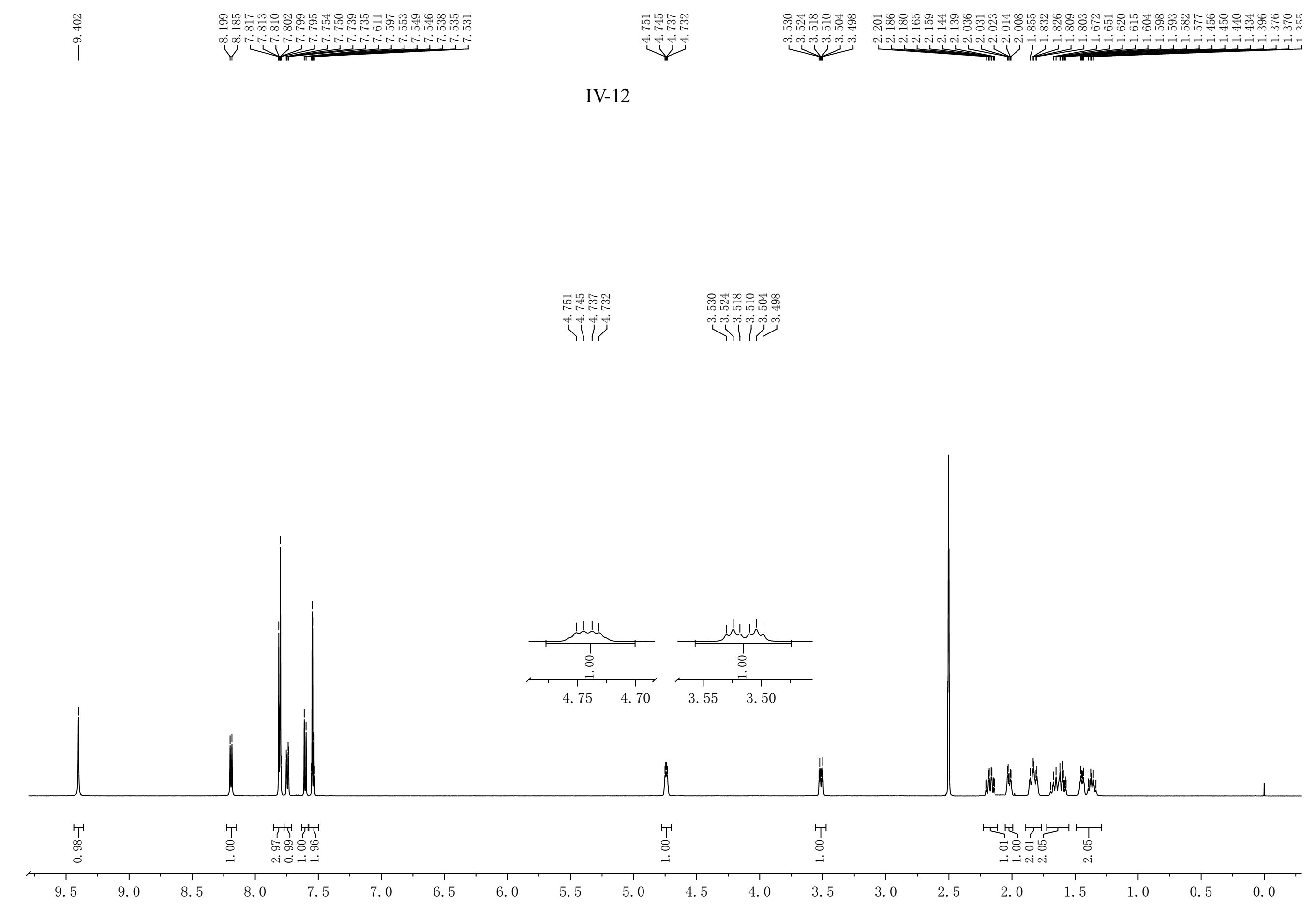


Figure S25-1 ^1^H NMR spectrum of compound **IV-12**


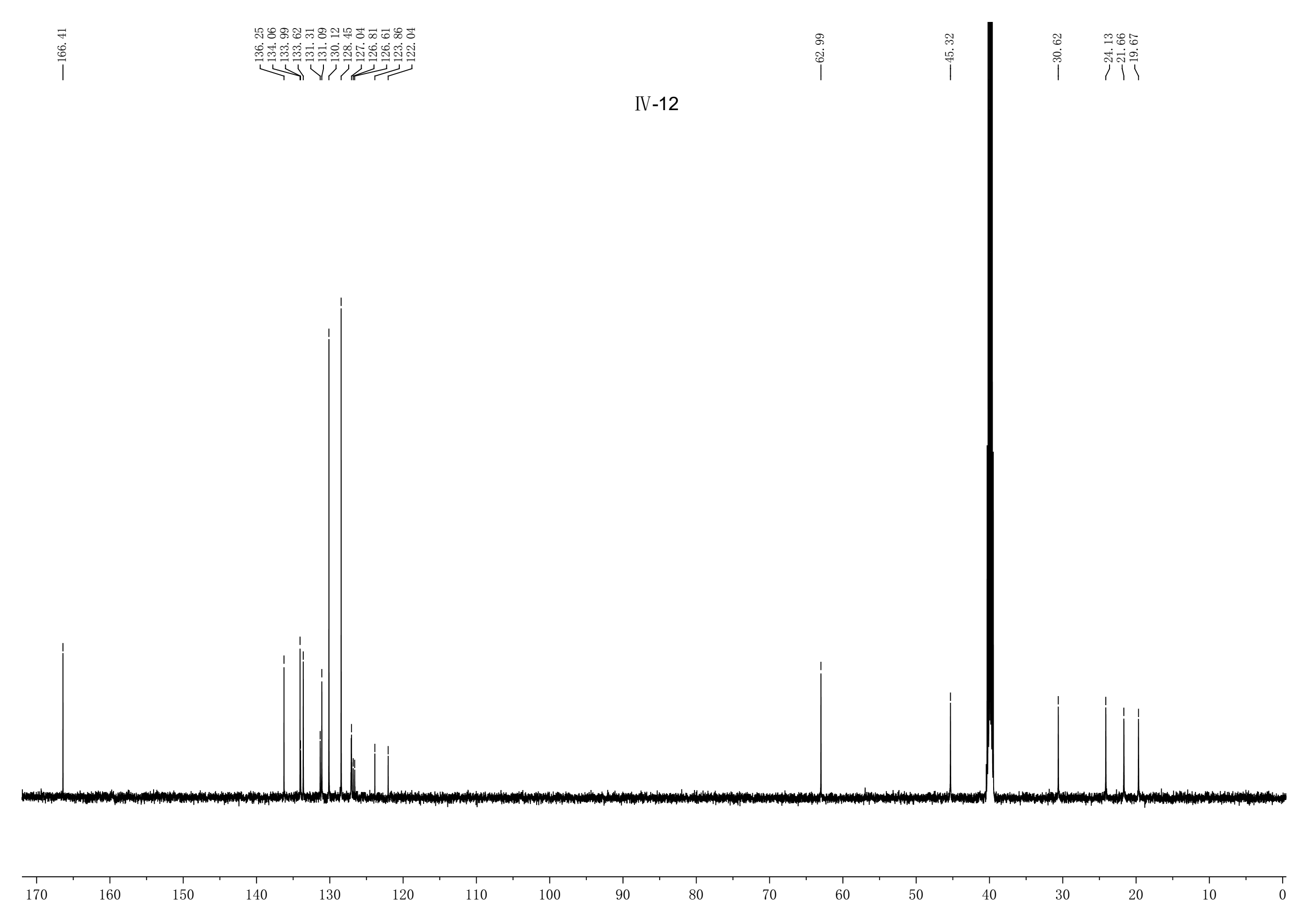


Figure S25-2 ^13^C NMR spectrum of compound **IV-12**


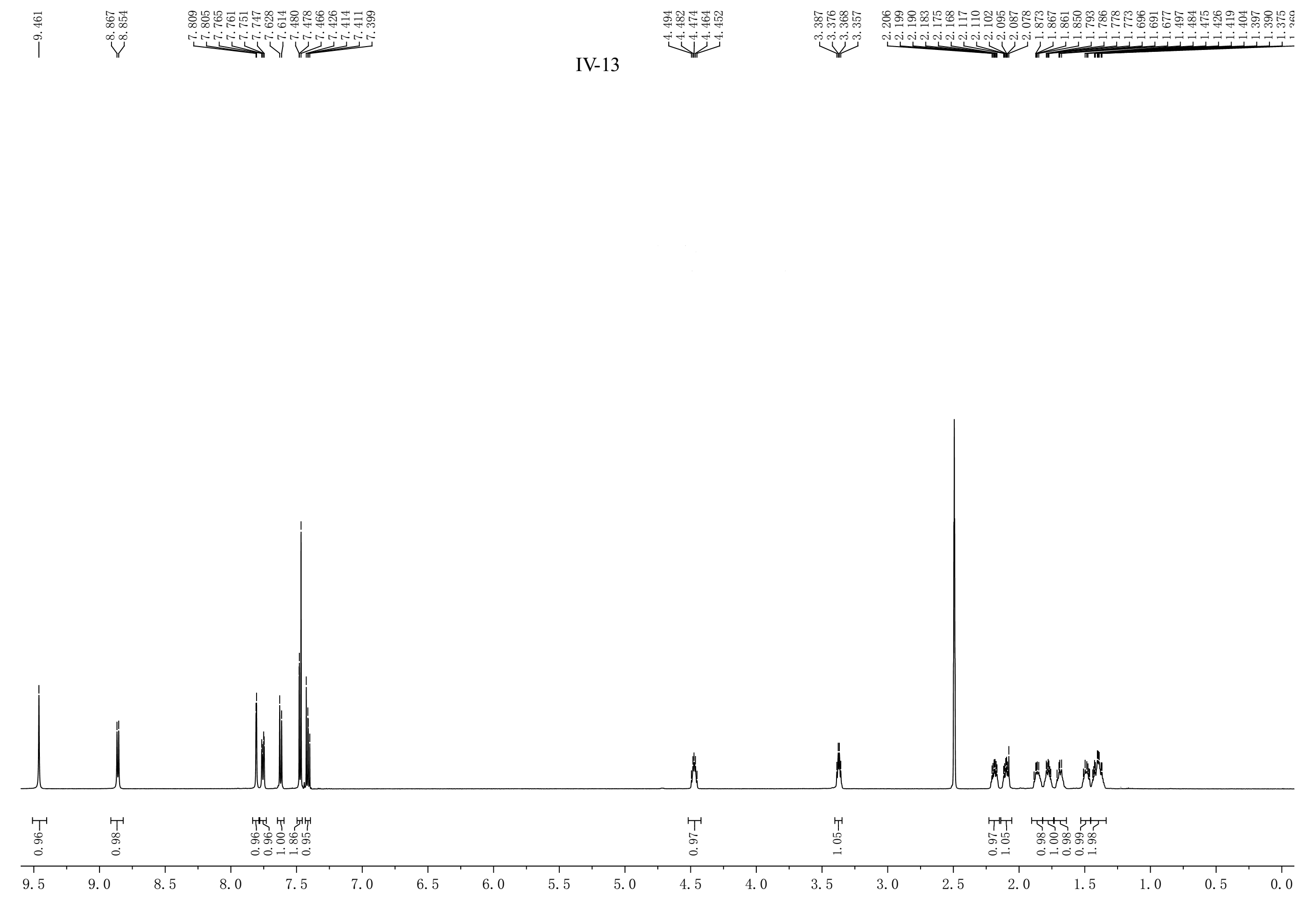


Figure S26-1 ^1^H NMR spectrum of compound IV-13


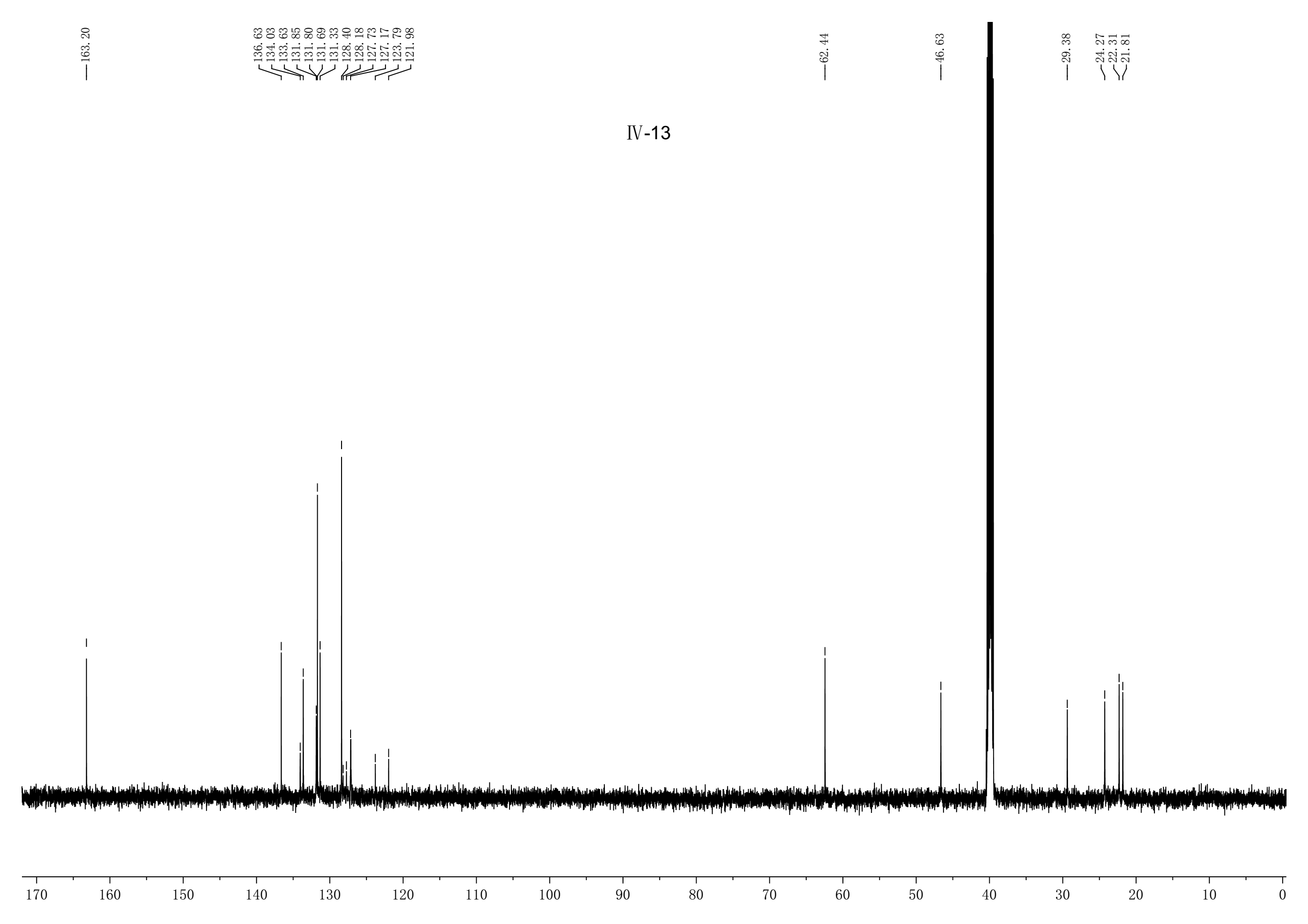


Figure S26-2 ^13^C NMR spectrum of compound **IV-13**


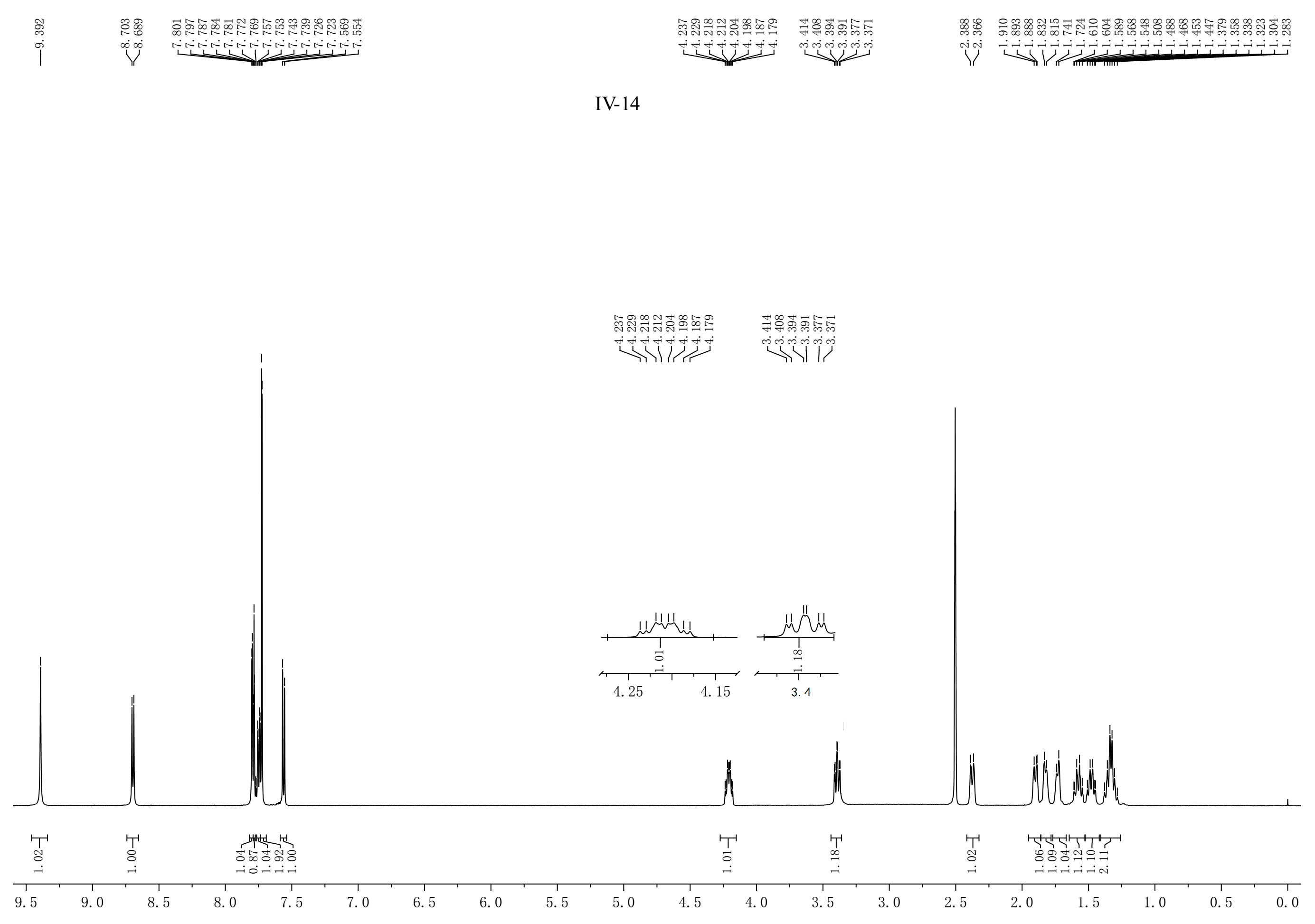


Figure S27-1 ^1^H NMR spectrum of compound **IV-14**


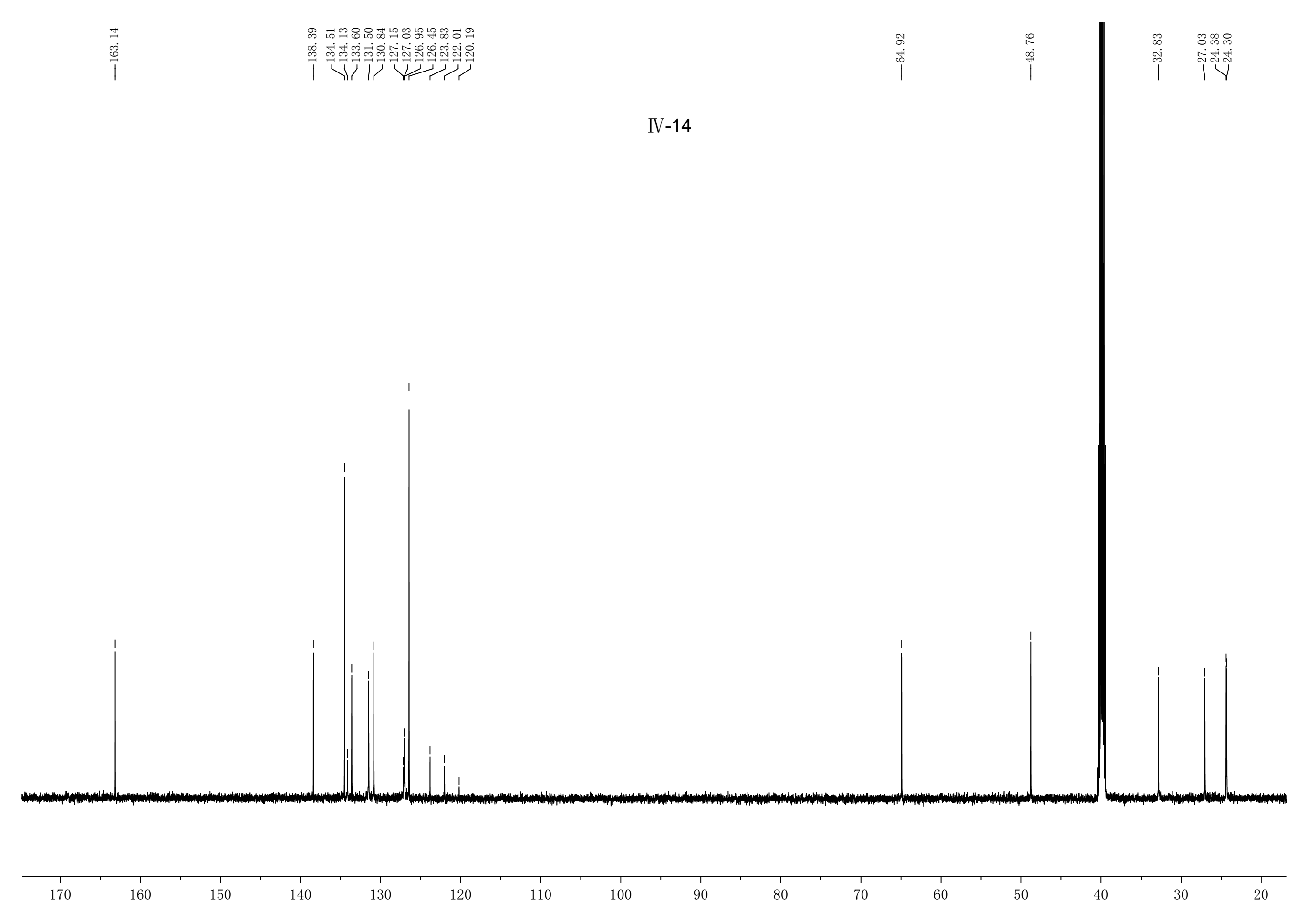


Figure S27-2 ^13^C NMR spectrum of compound **IV-14**


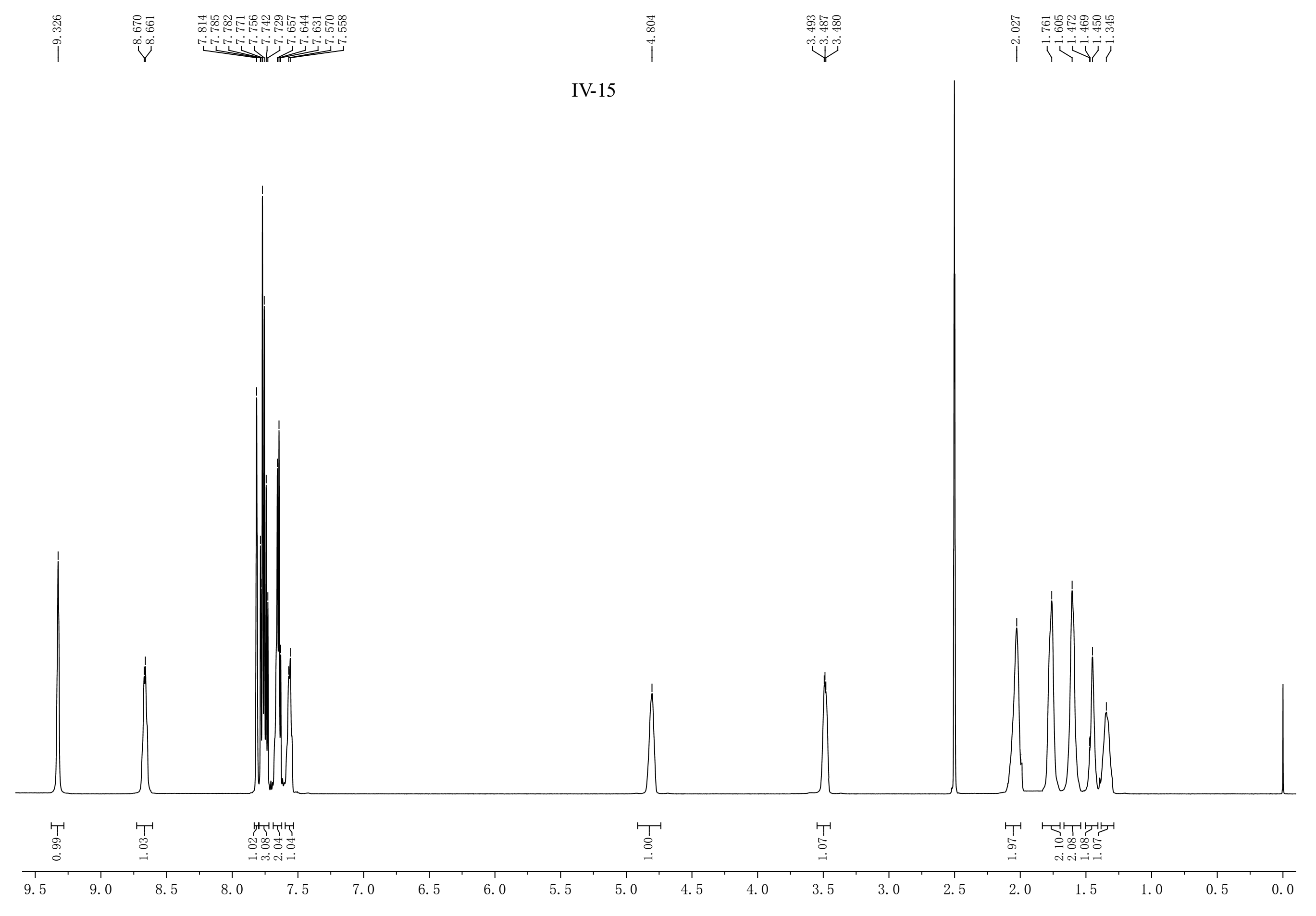


Figure S28-1 ^1^H NMR spectrum of compound **IV-15**


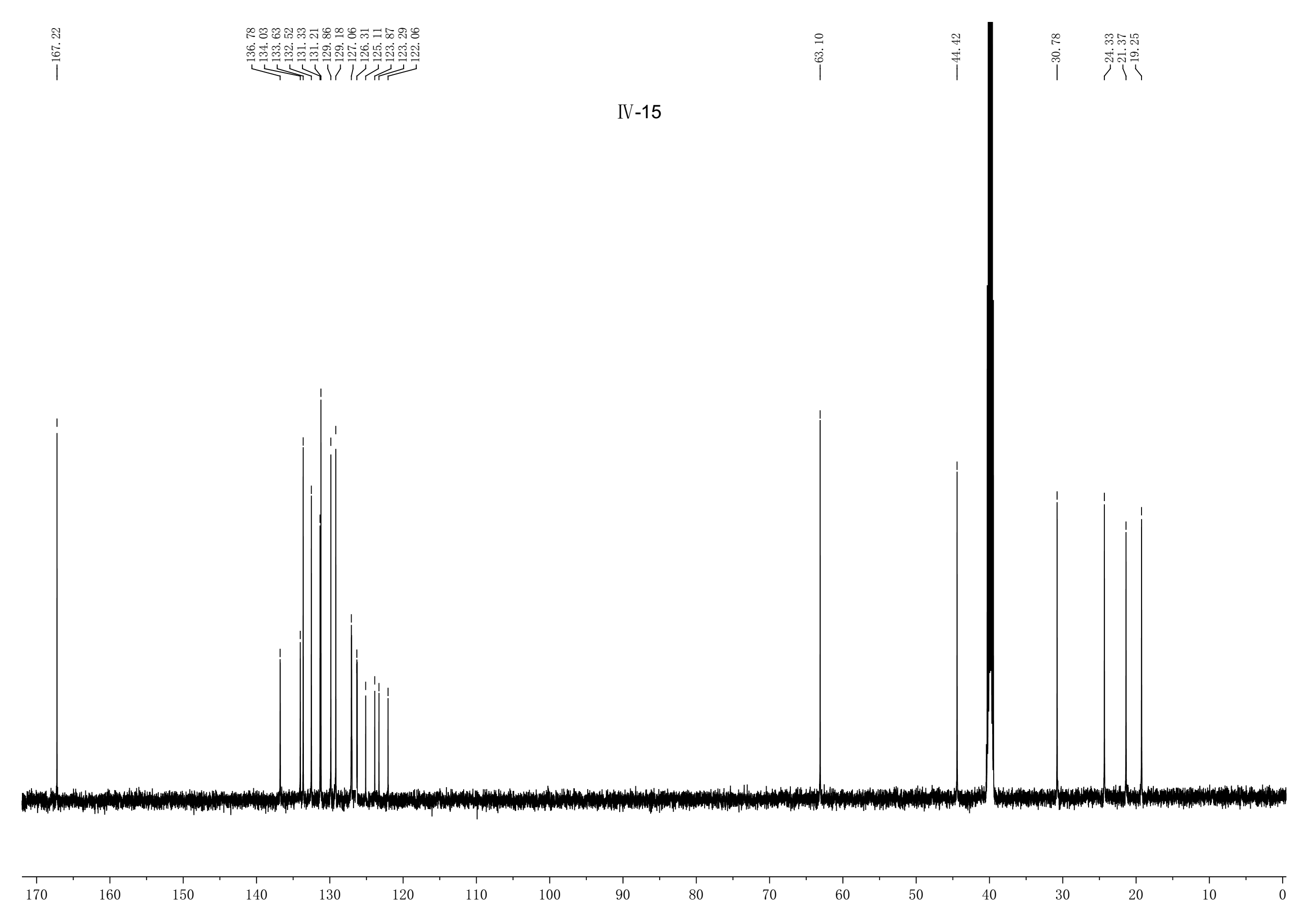


Figure S28-2 ^13^C NMR spectrum of compound **IV-15**


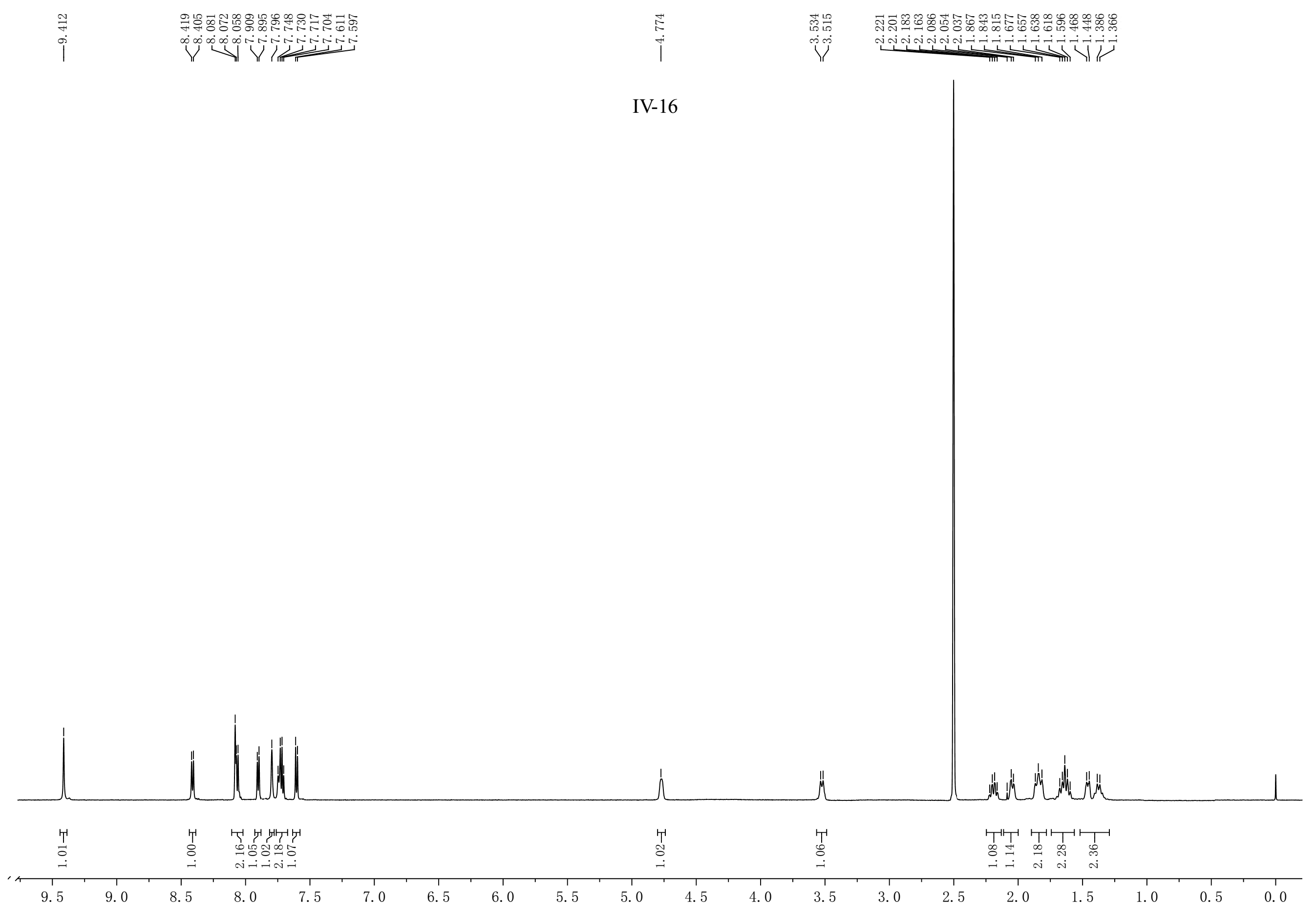


Figure S29-1 ^1^H NMR spectrum of compound **IV-16**


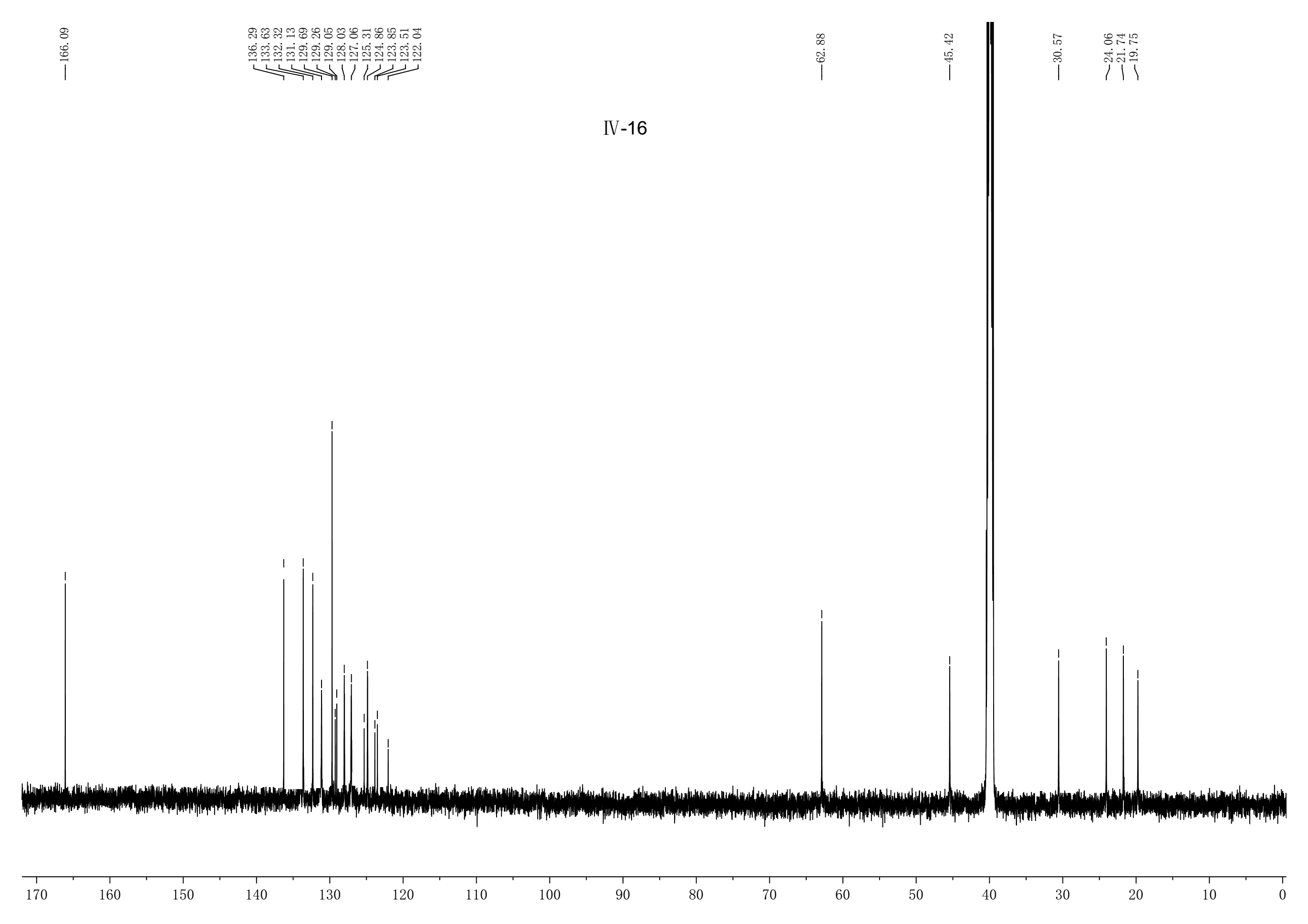


Figure S29-2 ^13^C NMR spectrum of compound **IV-16**


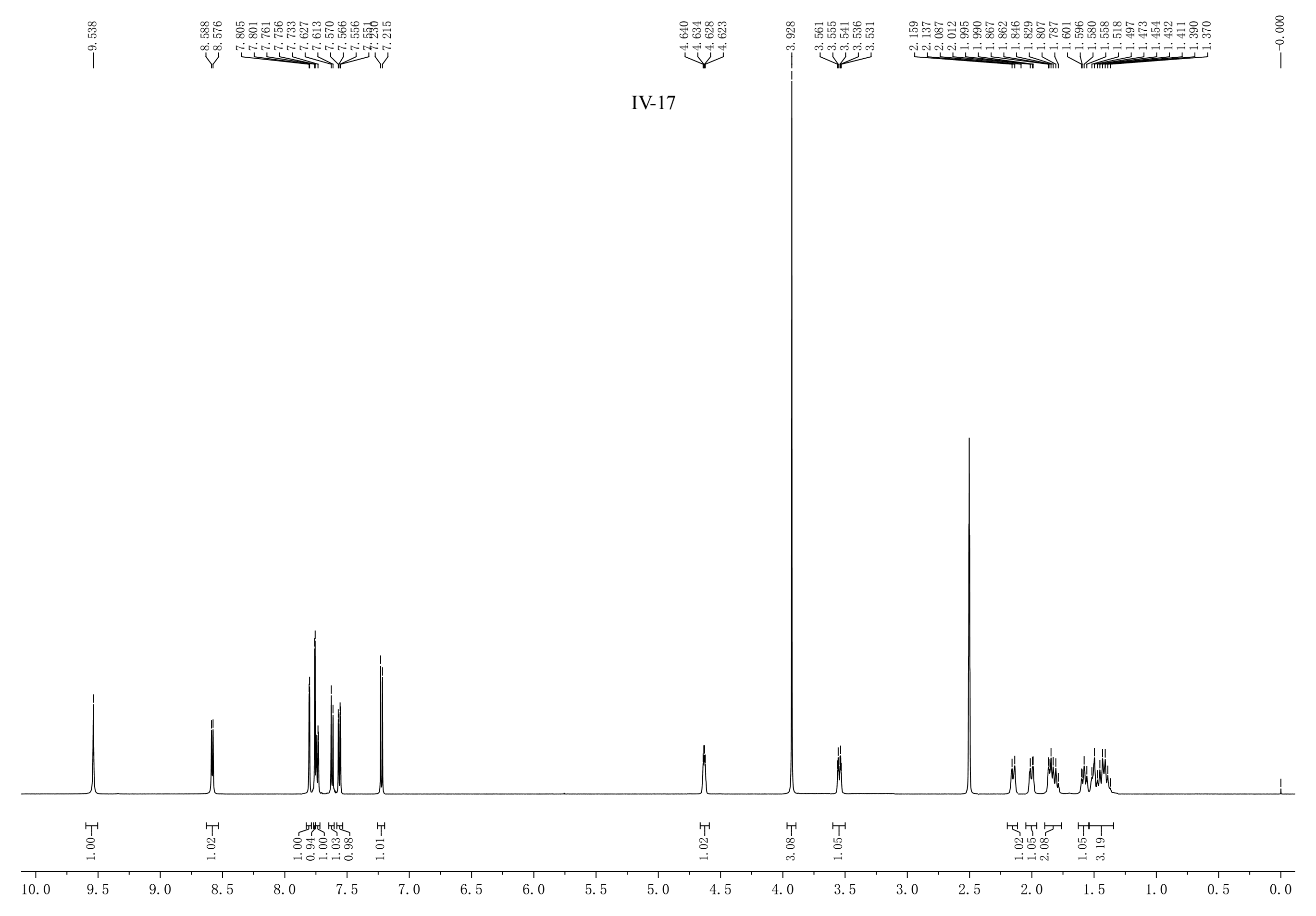


Figure S30-1 ^1^H NMR spectrum of compound **IV-17**


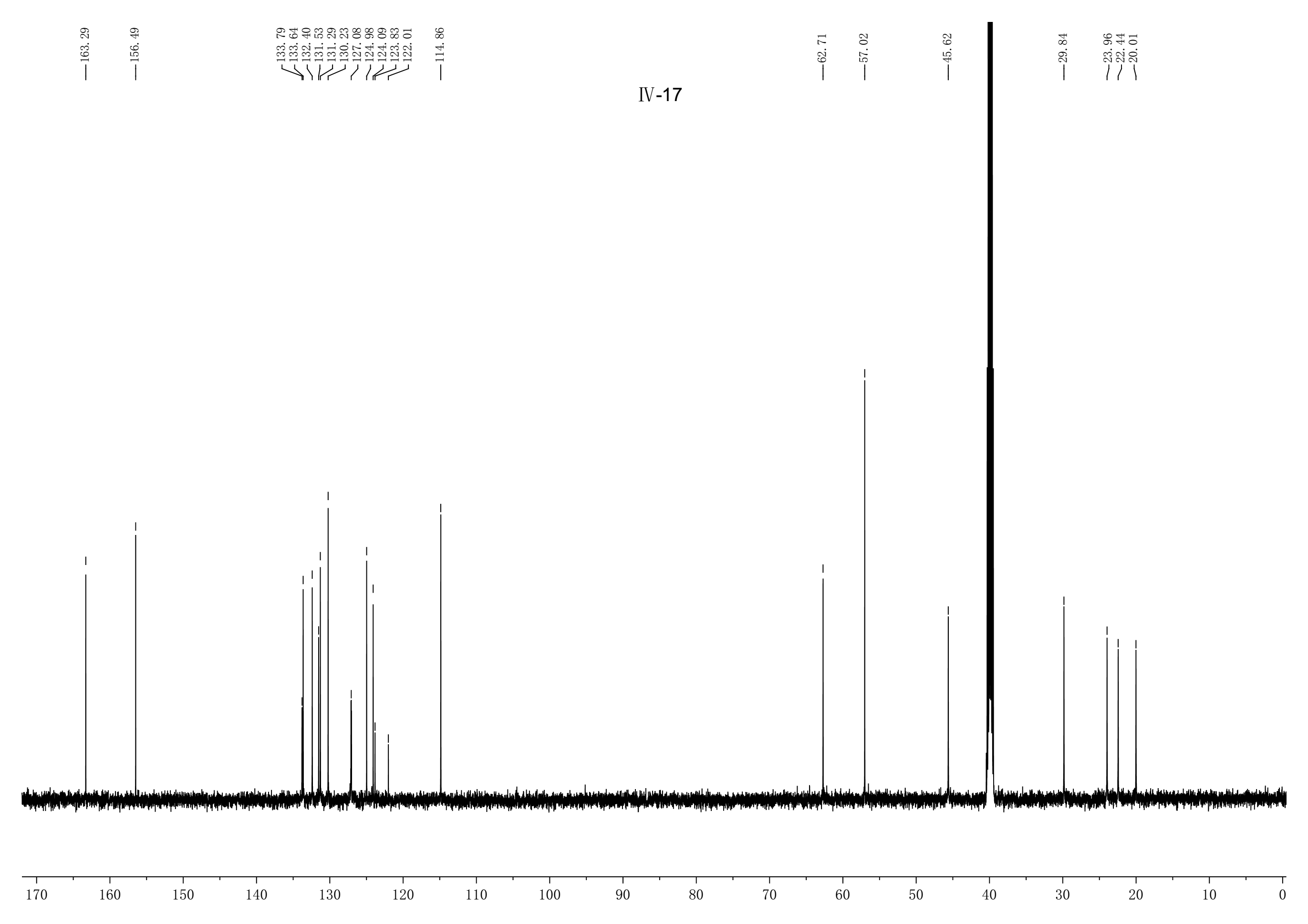


Figure S30-2 ^13^C NMR spectrum of compound **IV-17**


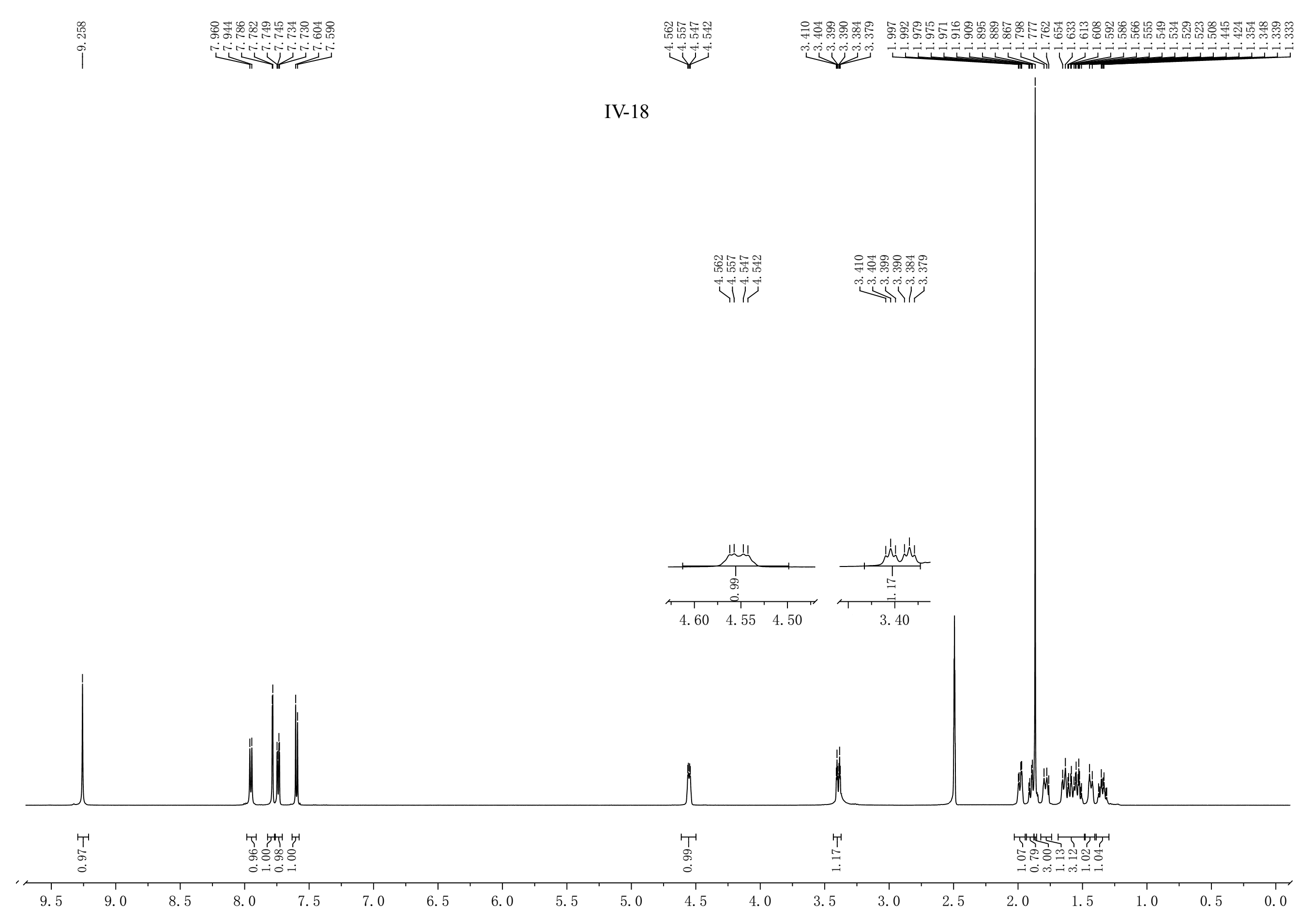


Figure S31-1 ^1^H NMR spectrum of compound **IV-18**


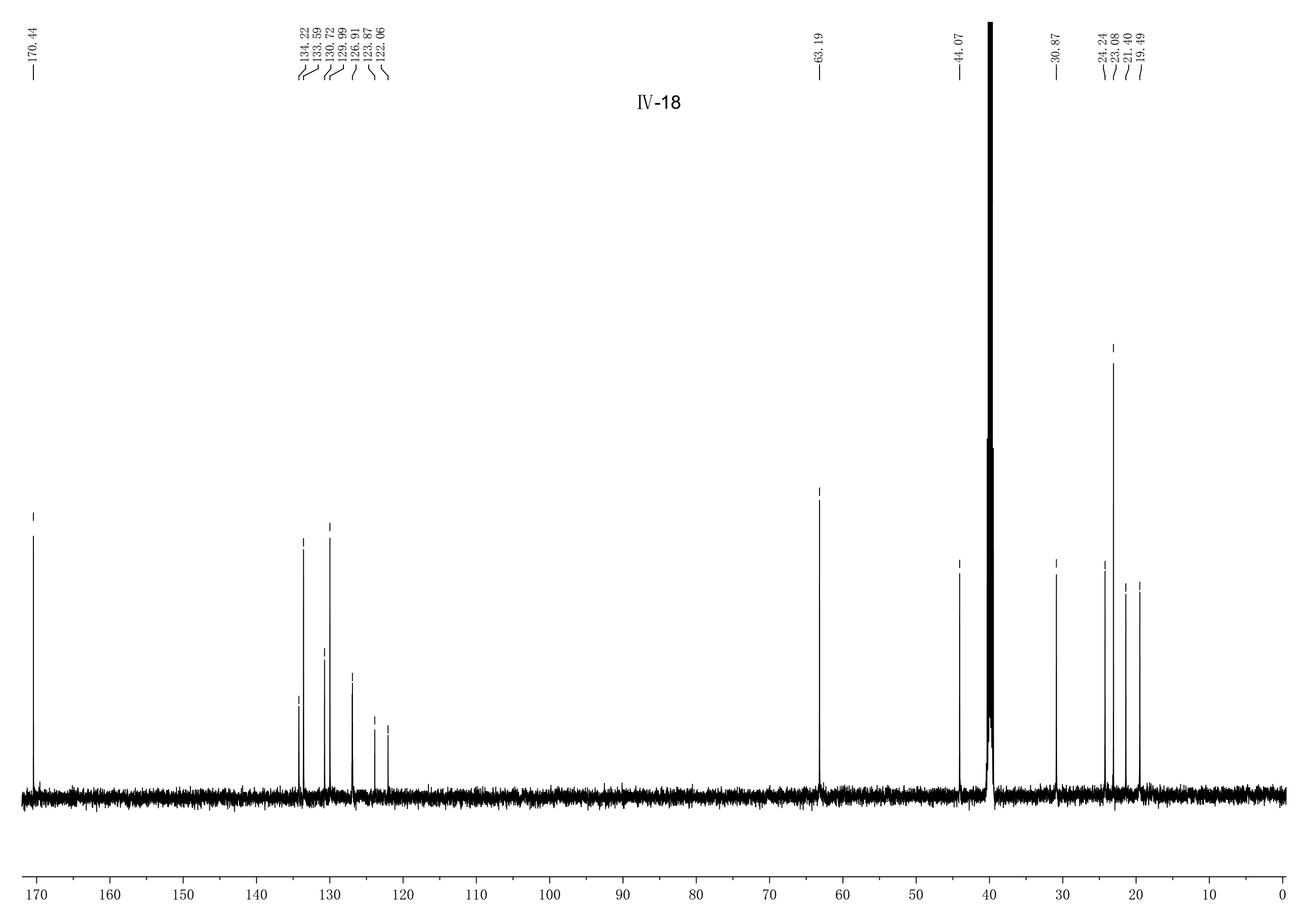


Figure S31-2 ^13^C NMR spectrum of compound **IV-18**


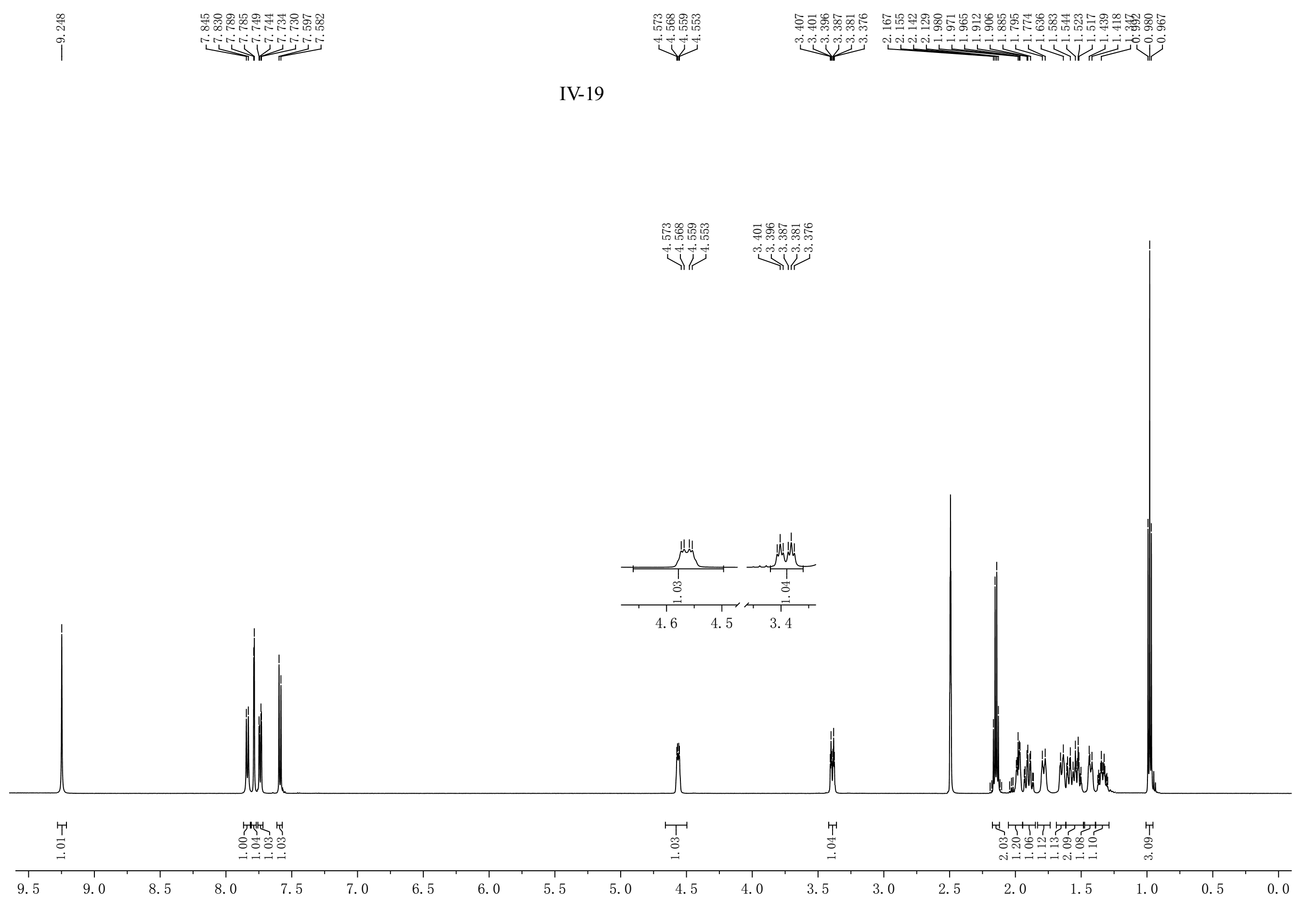


Figure S32-1 ^1^H NMR spectrum of compound **IV-19**


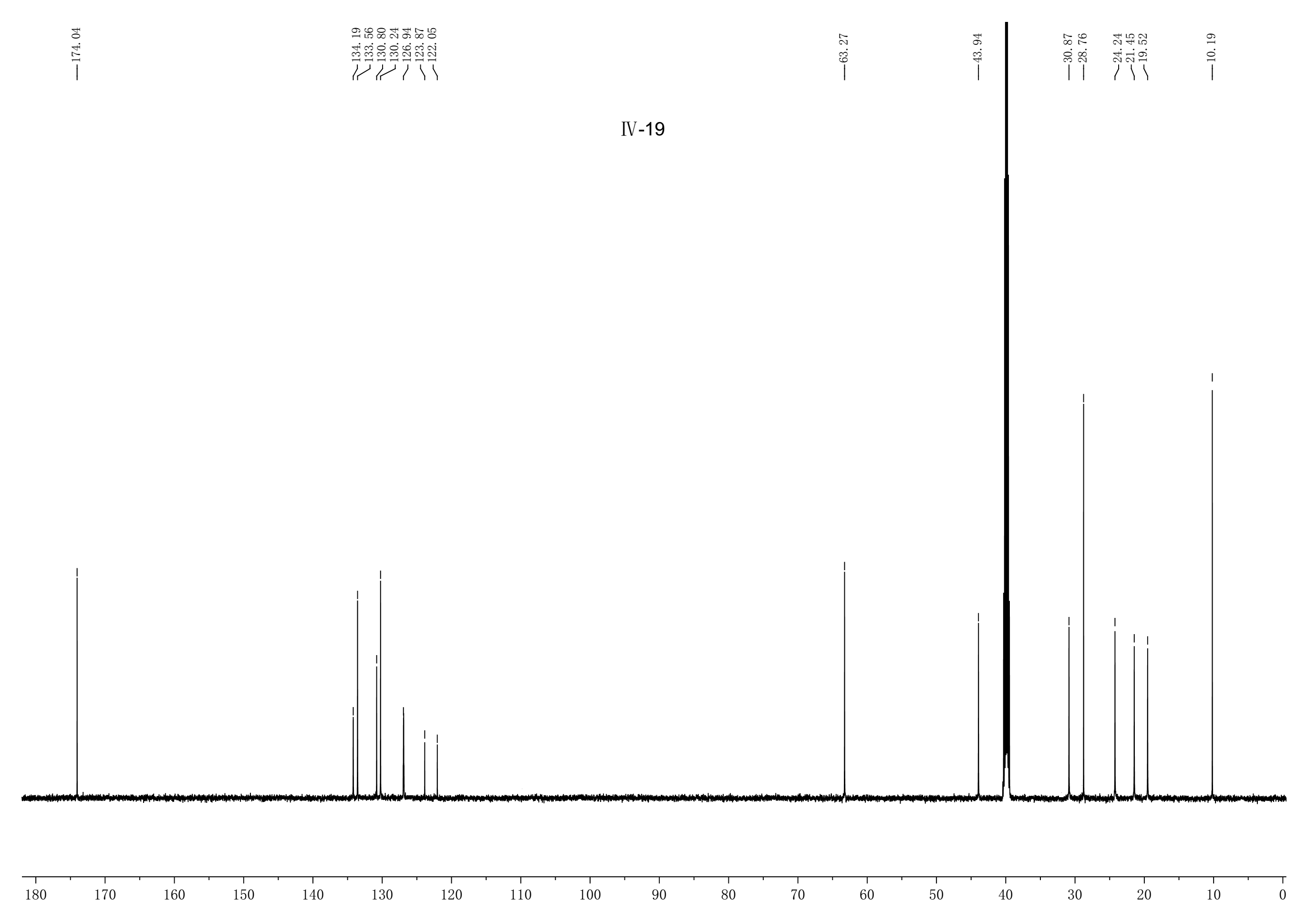


Figure S32-2 ^13^C NMR spectrum of compound **IV-19**


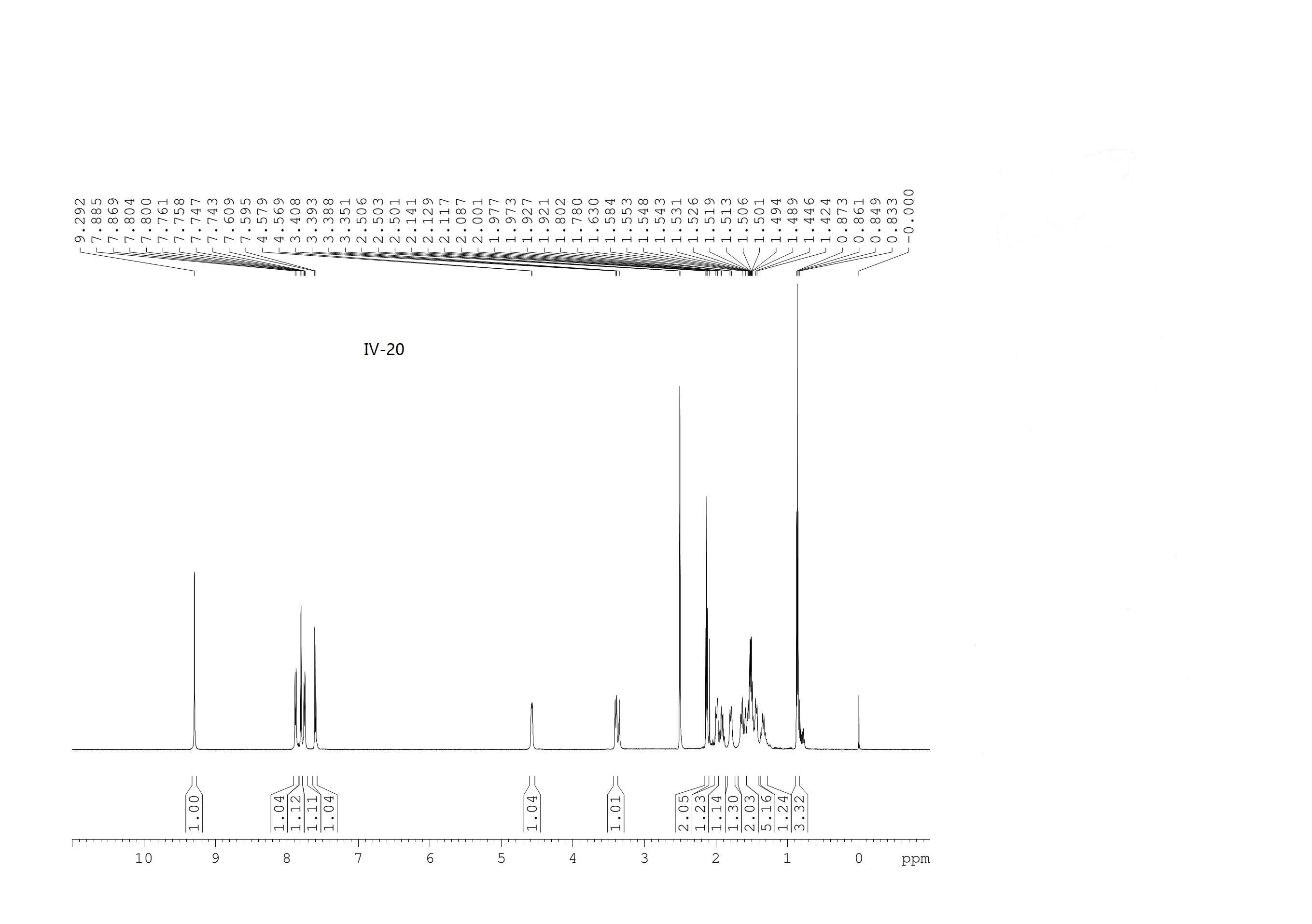


Figure S33-1 ^1^H NMR spectrum of compound **IV-20**


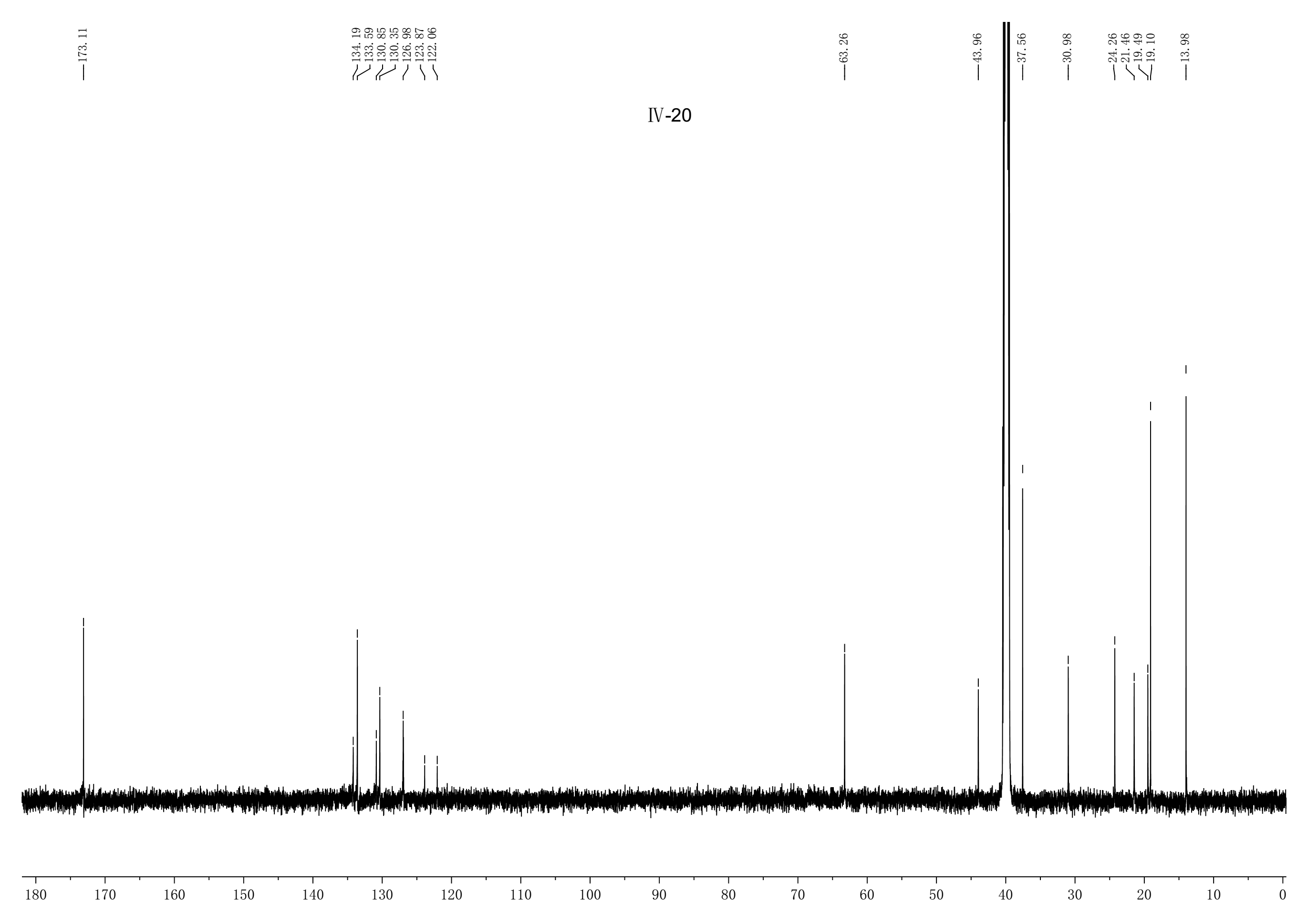


Figure S33-2 ^13^C NMR spectrum of compound **IV-20**


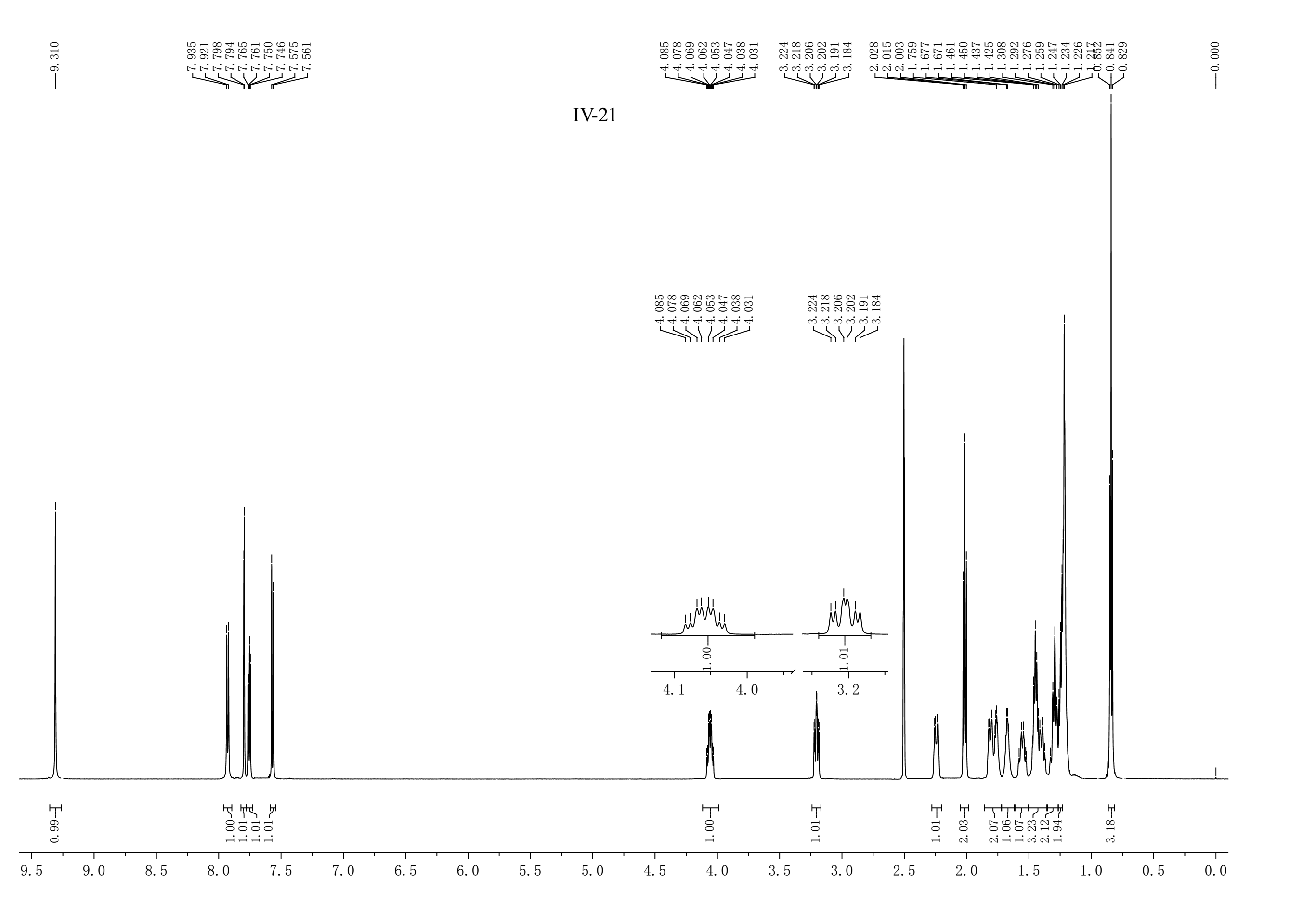


Figure S34-1 ^1^H NMR spectrum of compound **IV-21**


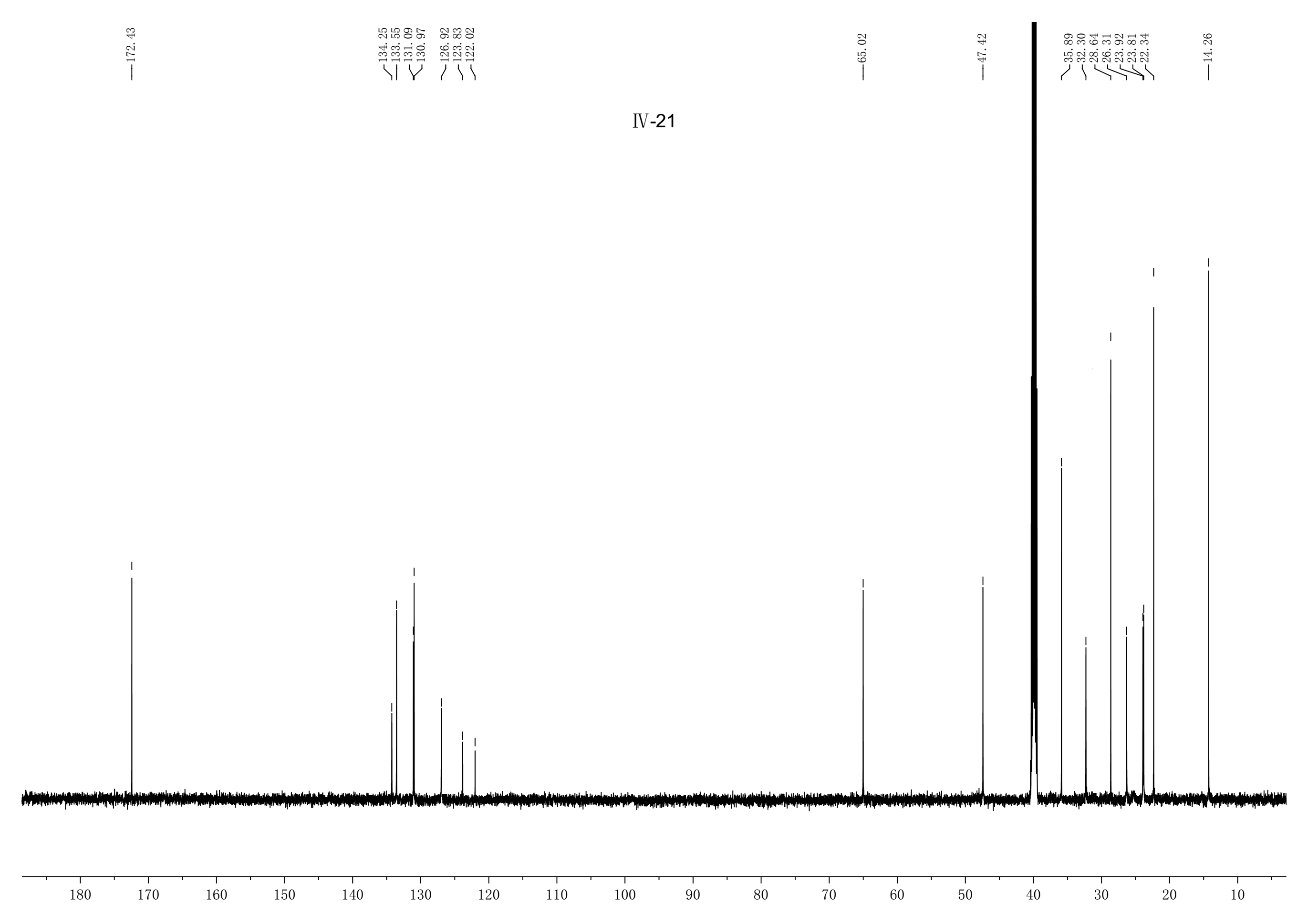


Figure S34-2 ^13^C NMR spectrum of compound **IV-21**


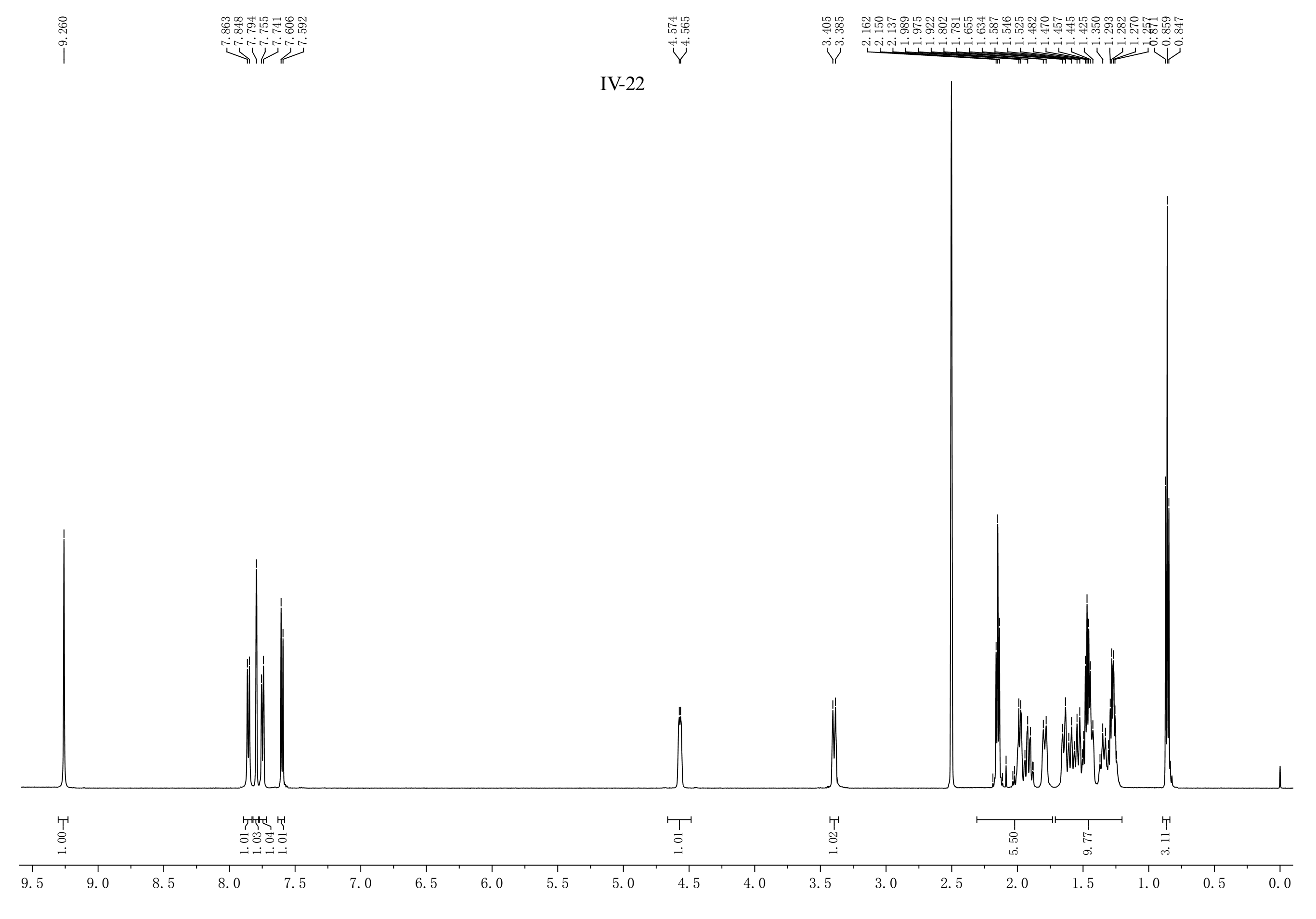


Figure S35-1 ^1^H NMR spectrum of compound **IV-22**


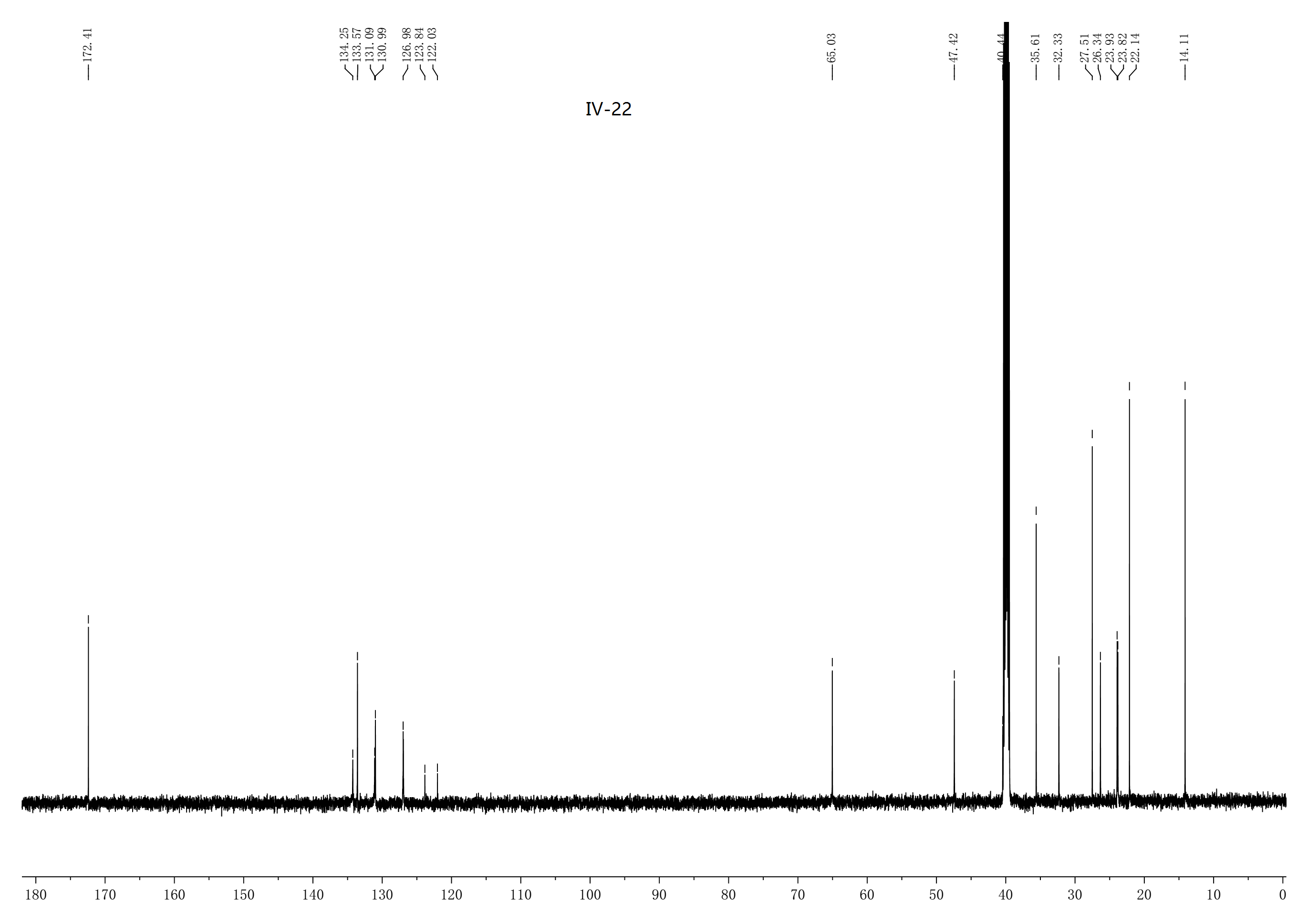


Figure S35-2 ^13^C NMR spectrum of compound **IV-22**


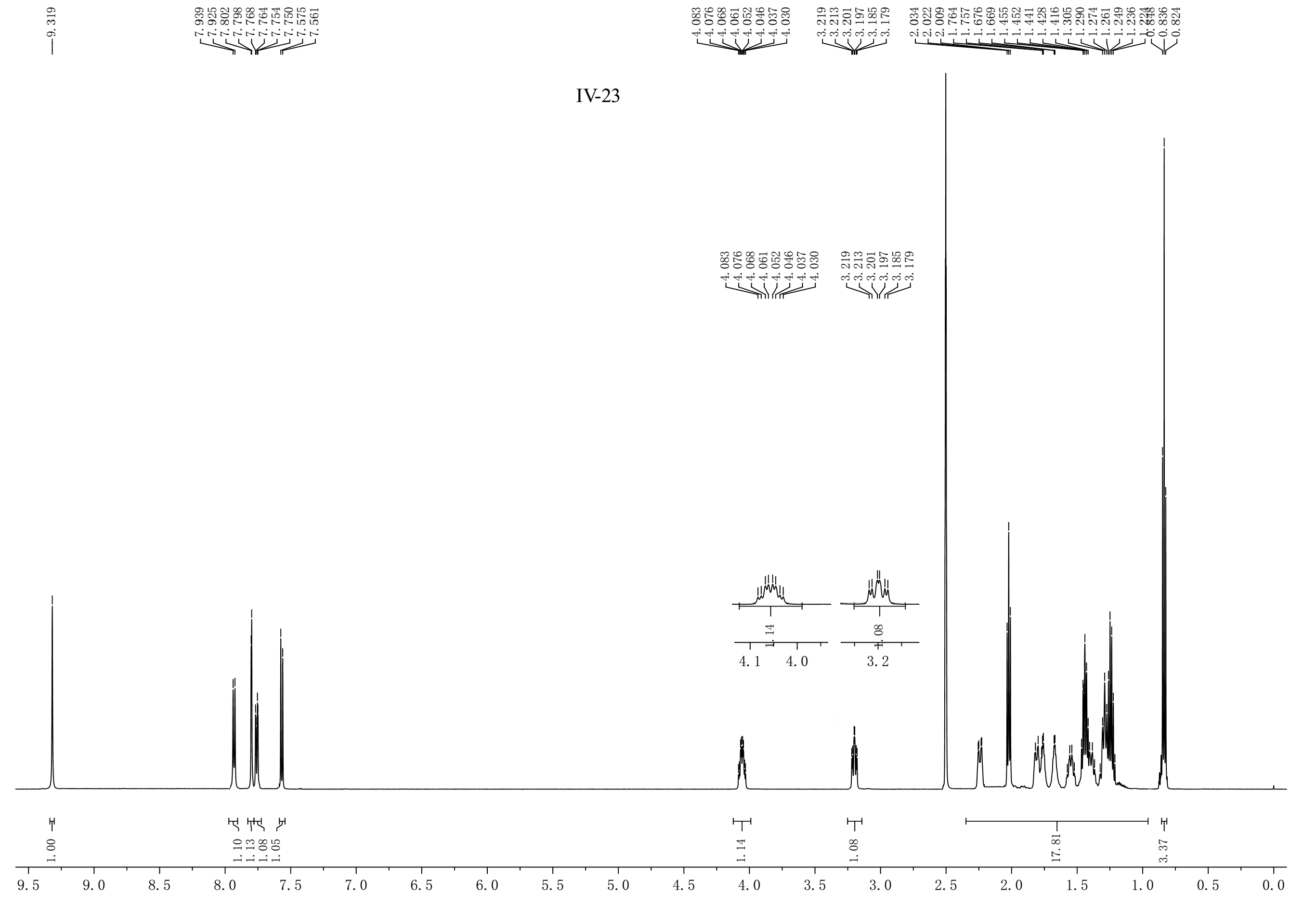


Figure S36-1 ^1^H NMR spectrum of compound **IV-23**

**
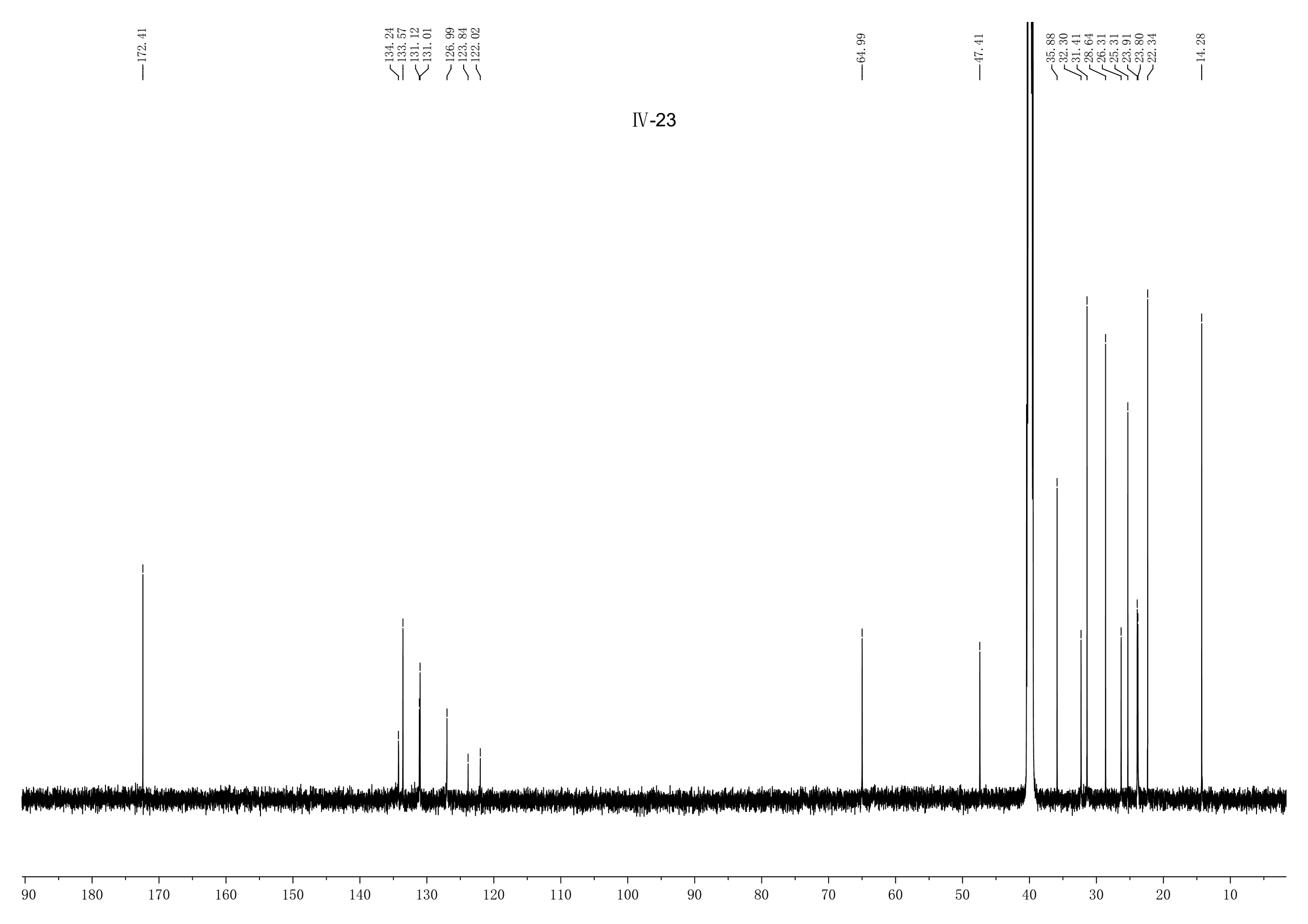
**

Figure S36-2 ^13^C NMR spectrum of compound **IV-23**


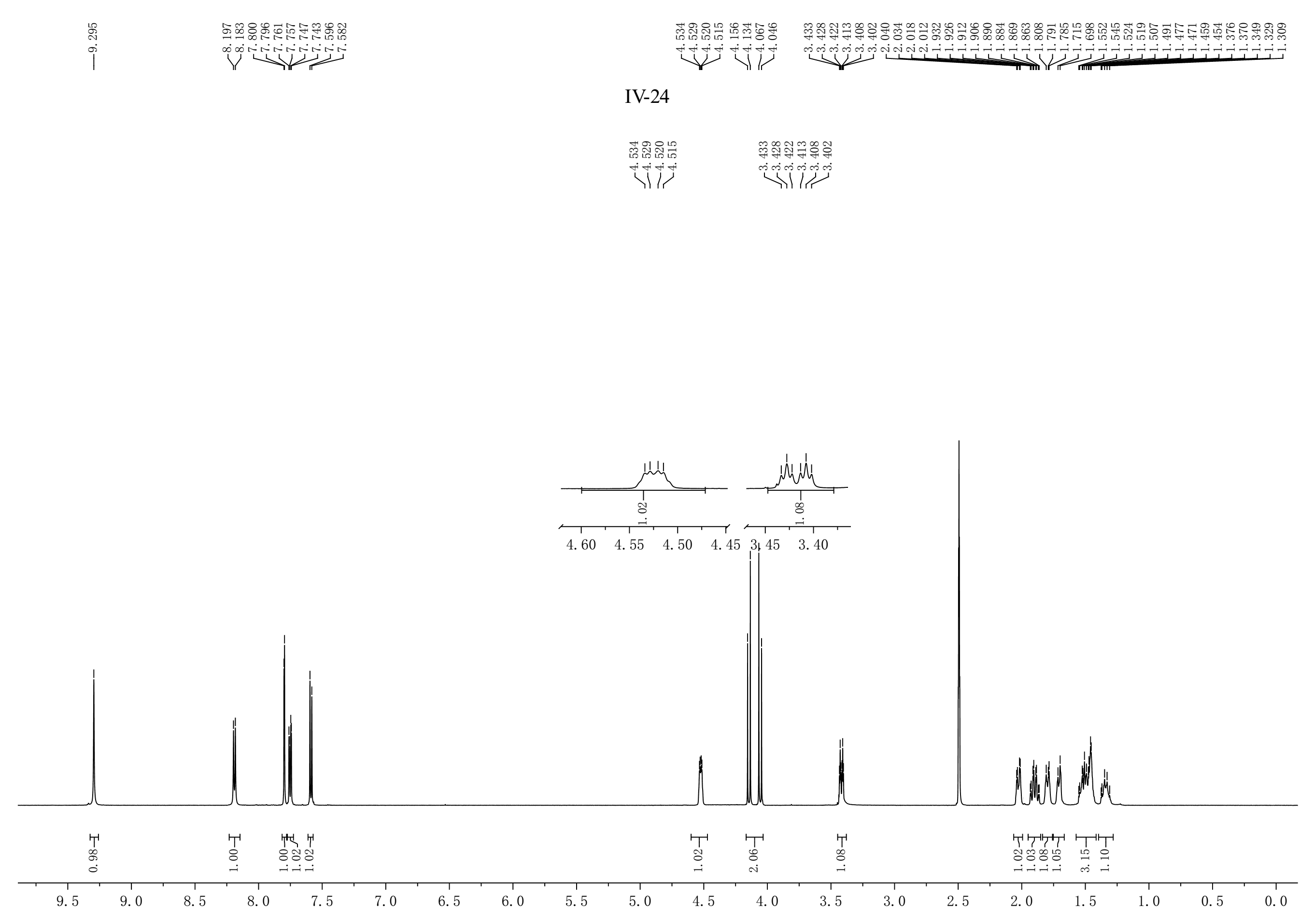


Figure S37-1 ^1^H NMR spectrum of compound **IV-24**


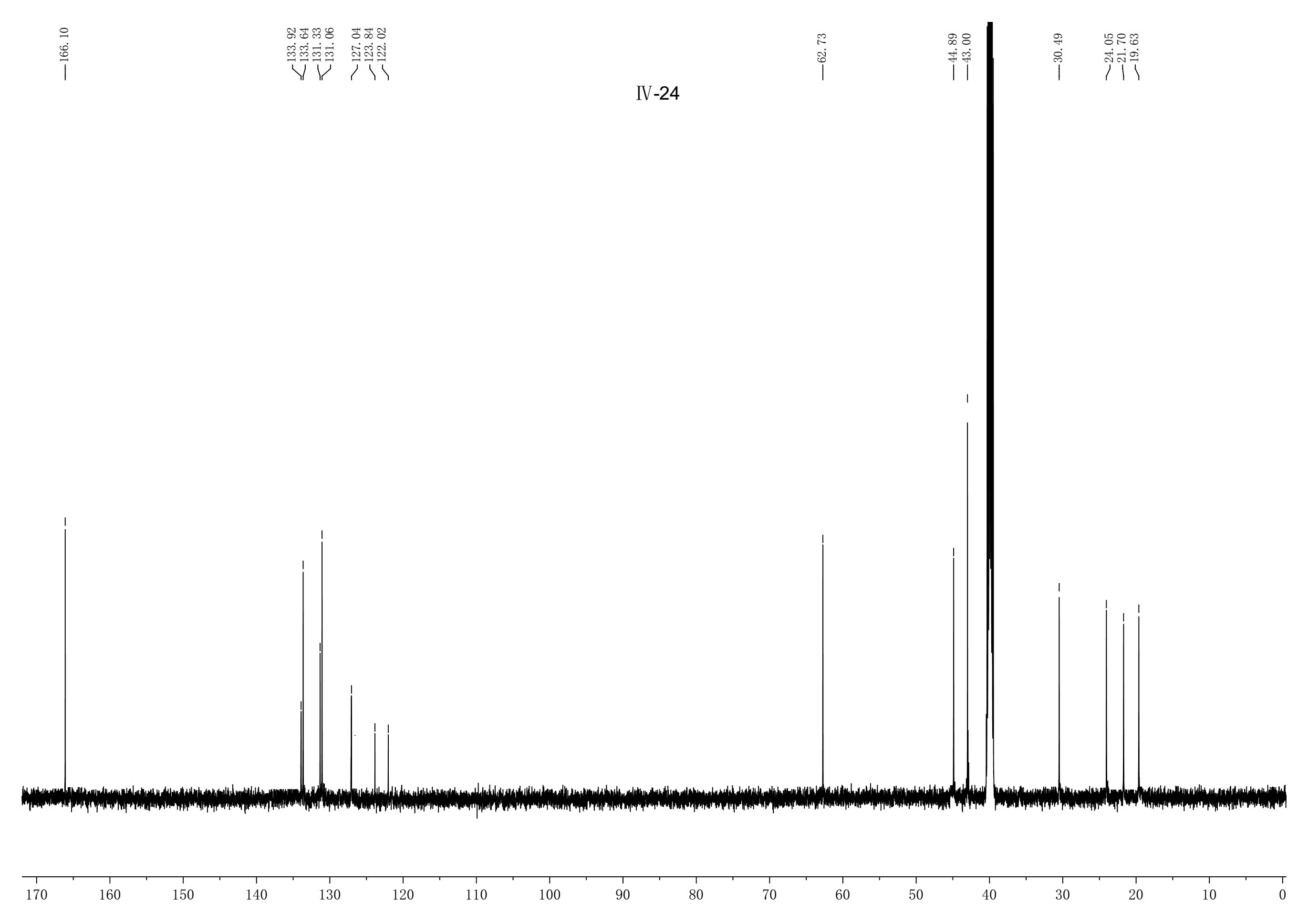


Figure S37-2 ^13^C NMR spectrum of compound **IV-24**


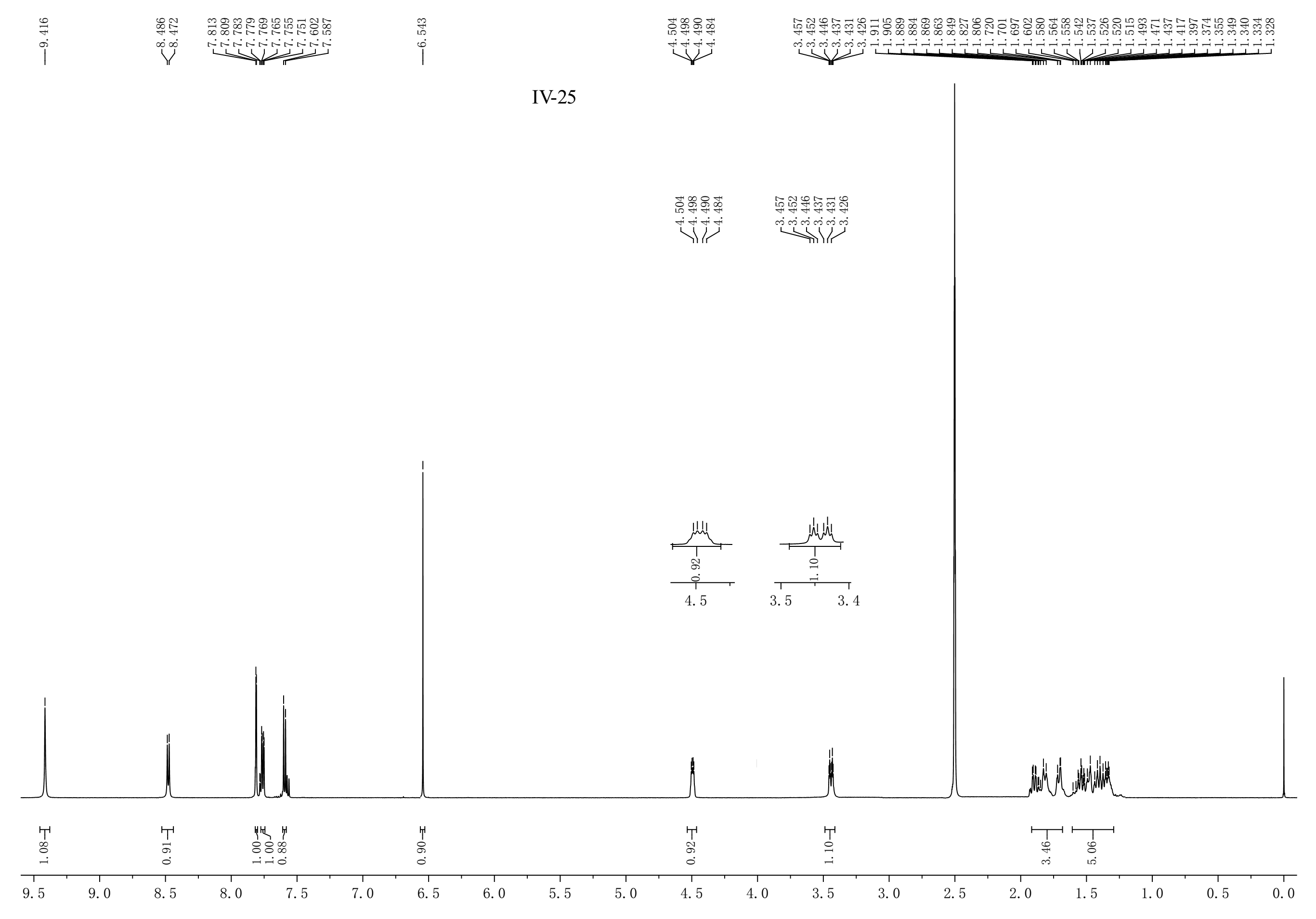


Figure S38-1 ^1^H NMR spectrum of compound **IV-25**


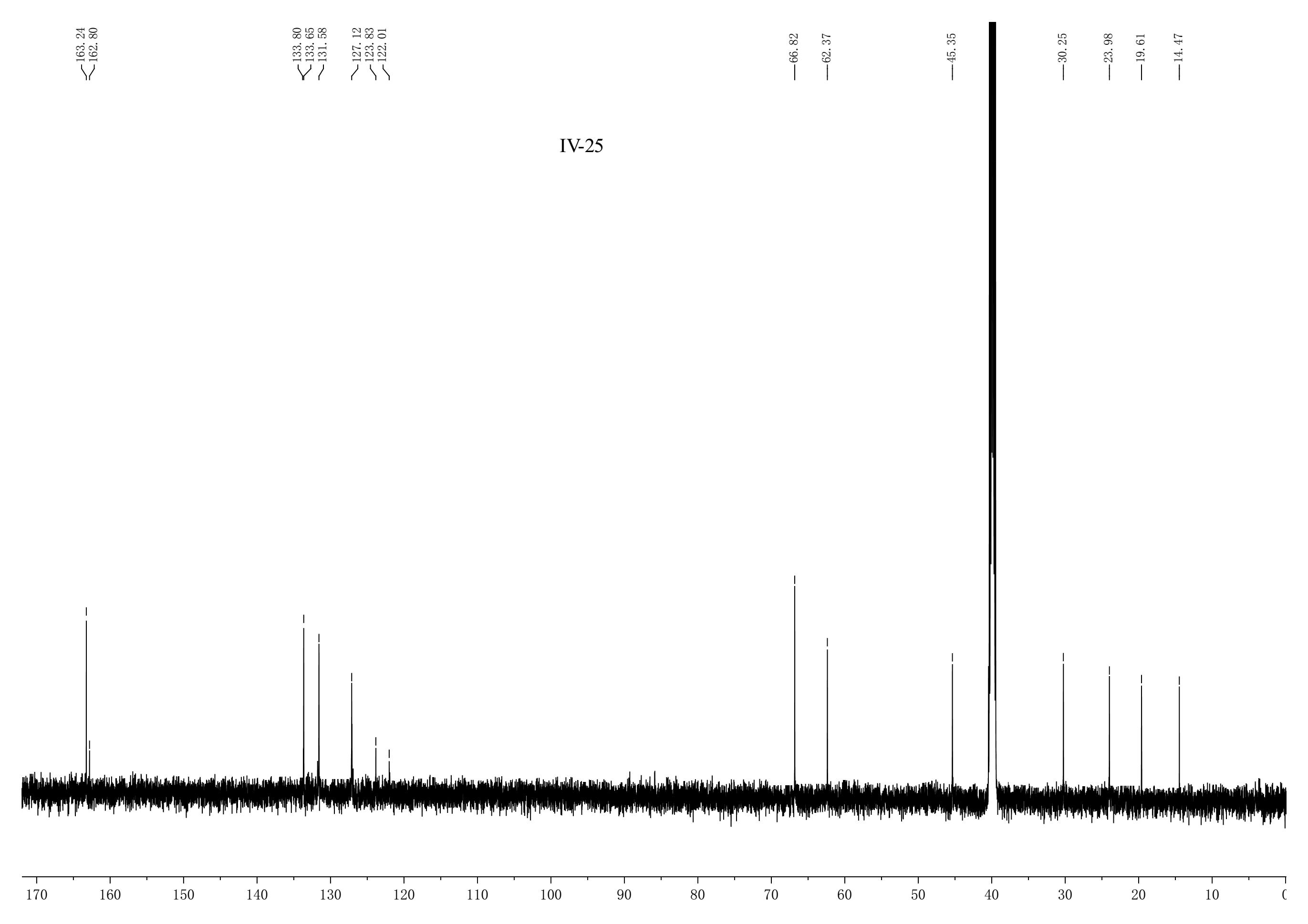


Figure S38-2 ^13^C NMR spectrum of compound **IV-25**


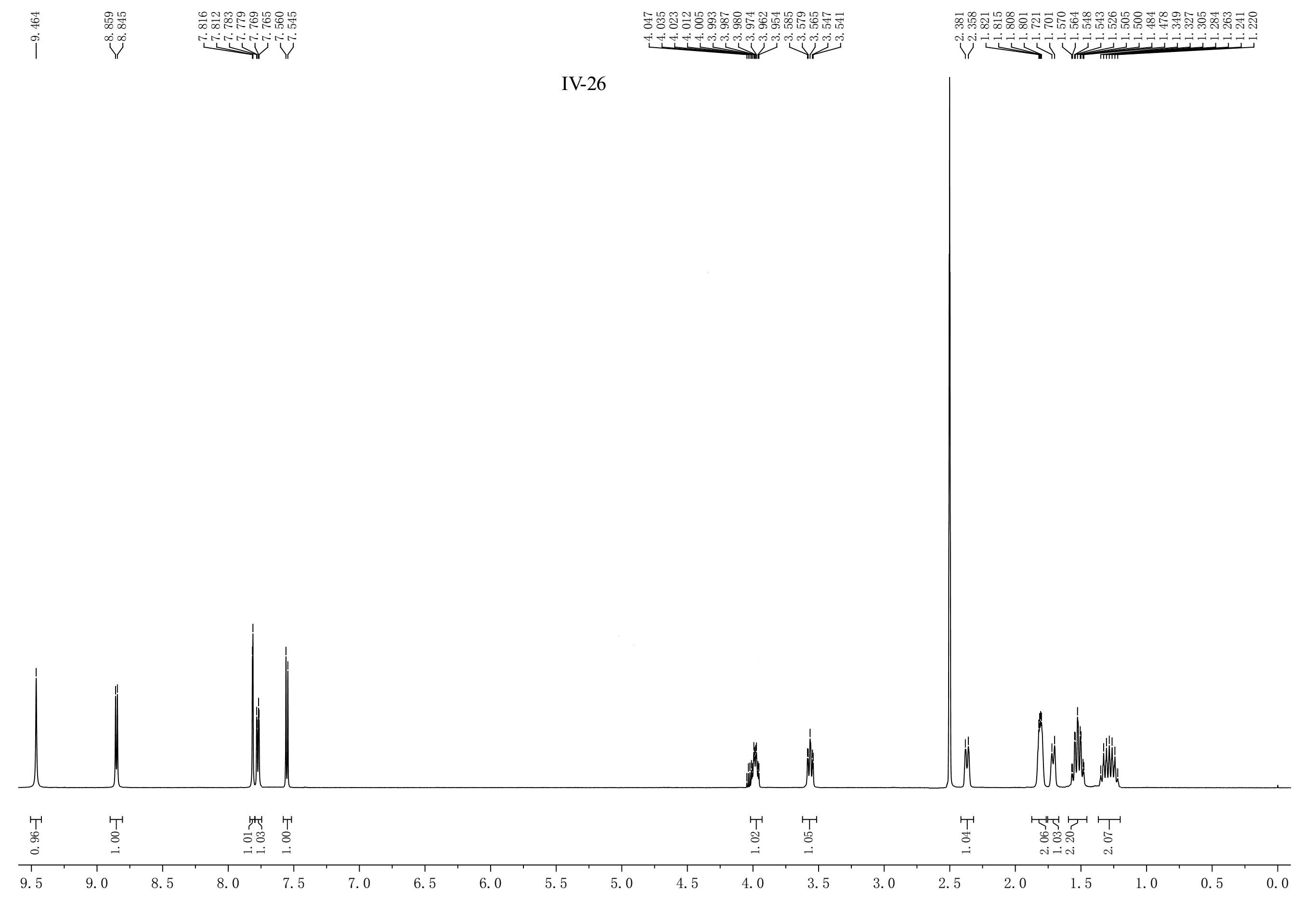


Figure S39-1 ^1^H NMR spectrum of compound **IV-26**


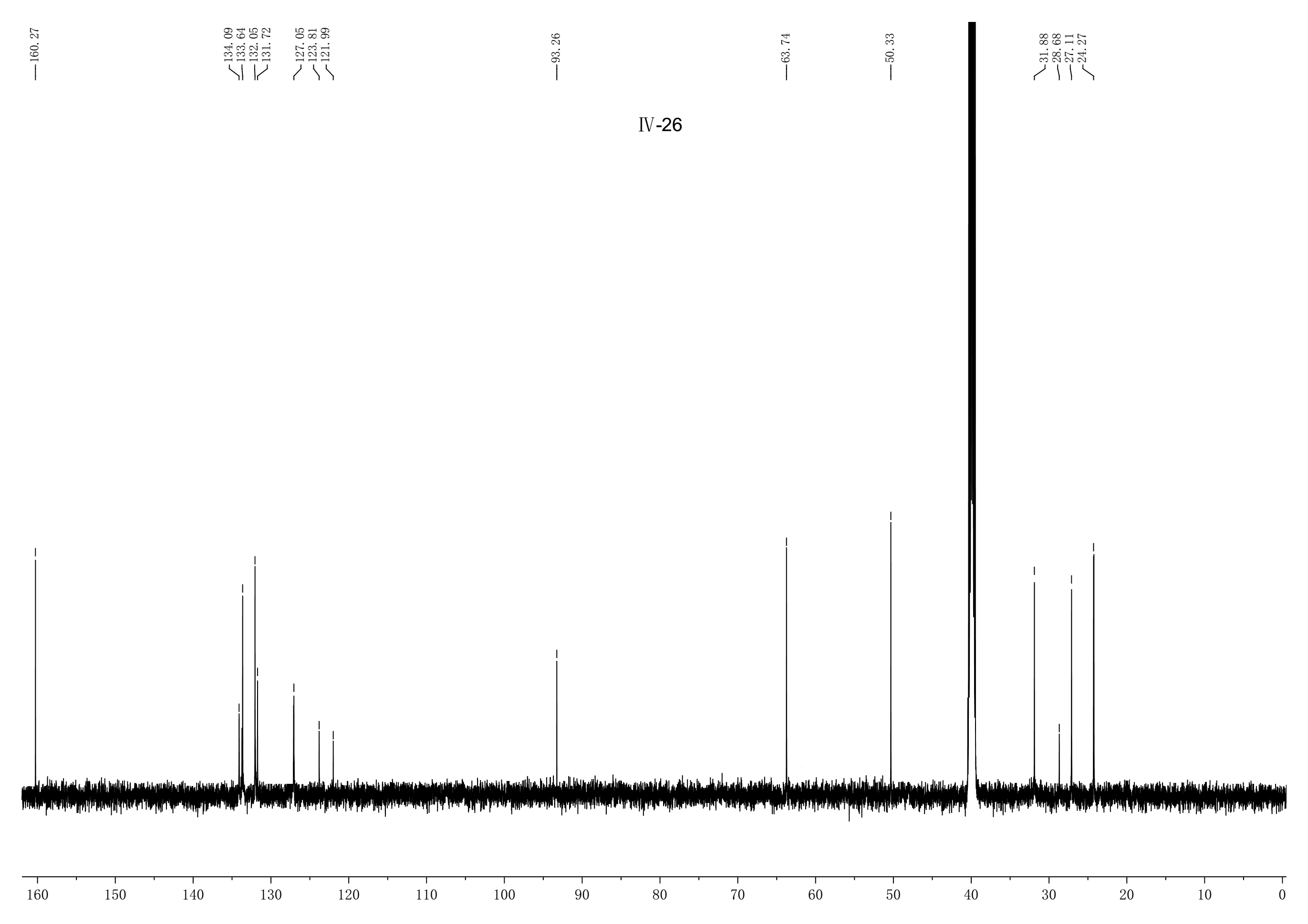


Figure S39-2 ^13^C NMR spectrum of compound **IV-26**


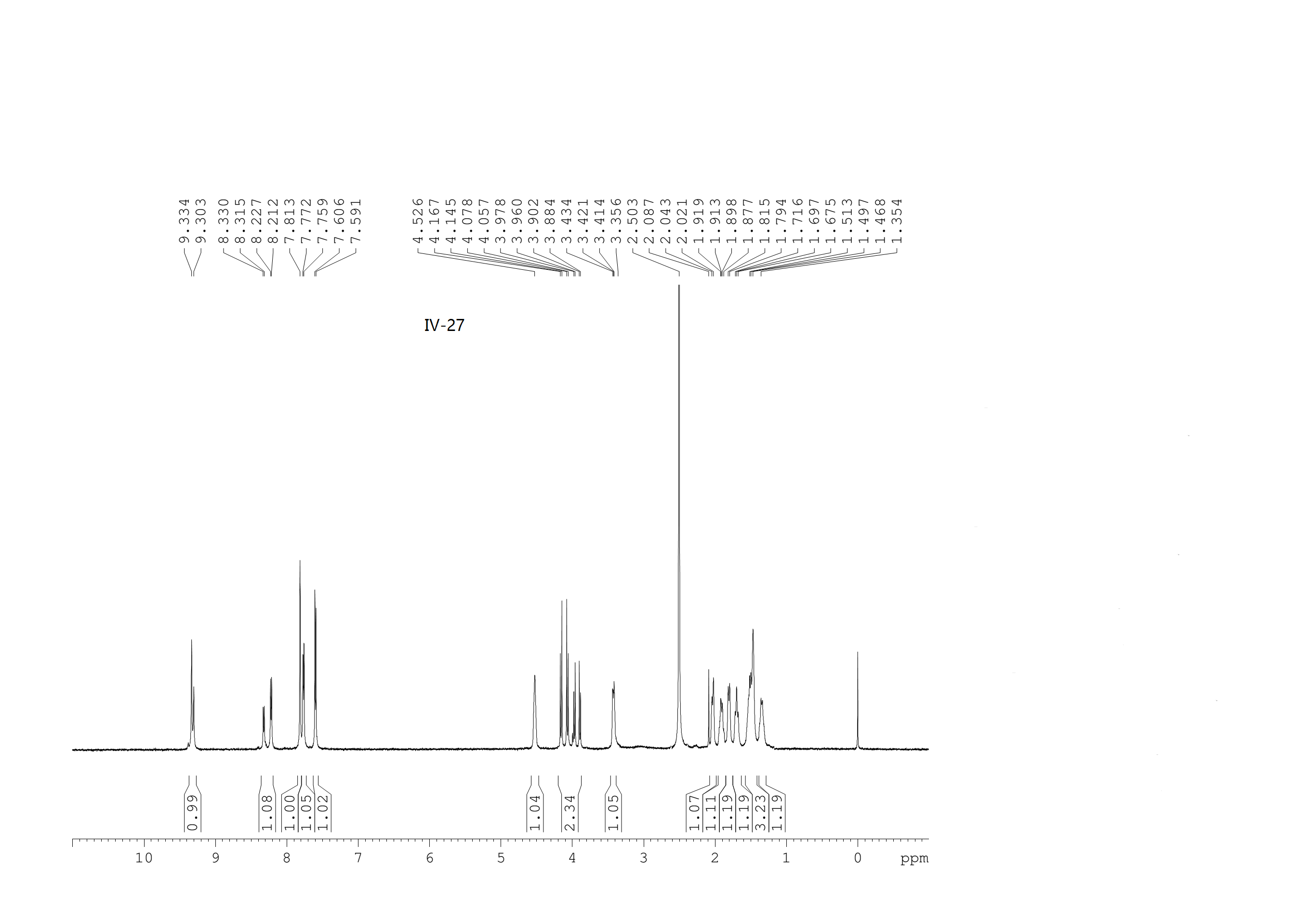


Figure S40-1 ^1^H NMR spectrum of compound **IV-27**


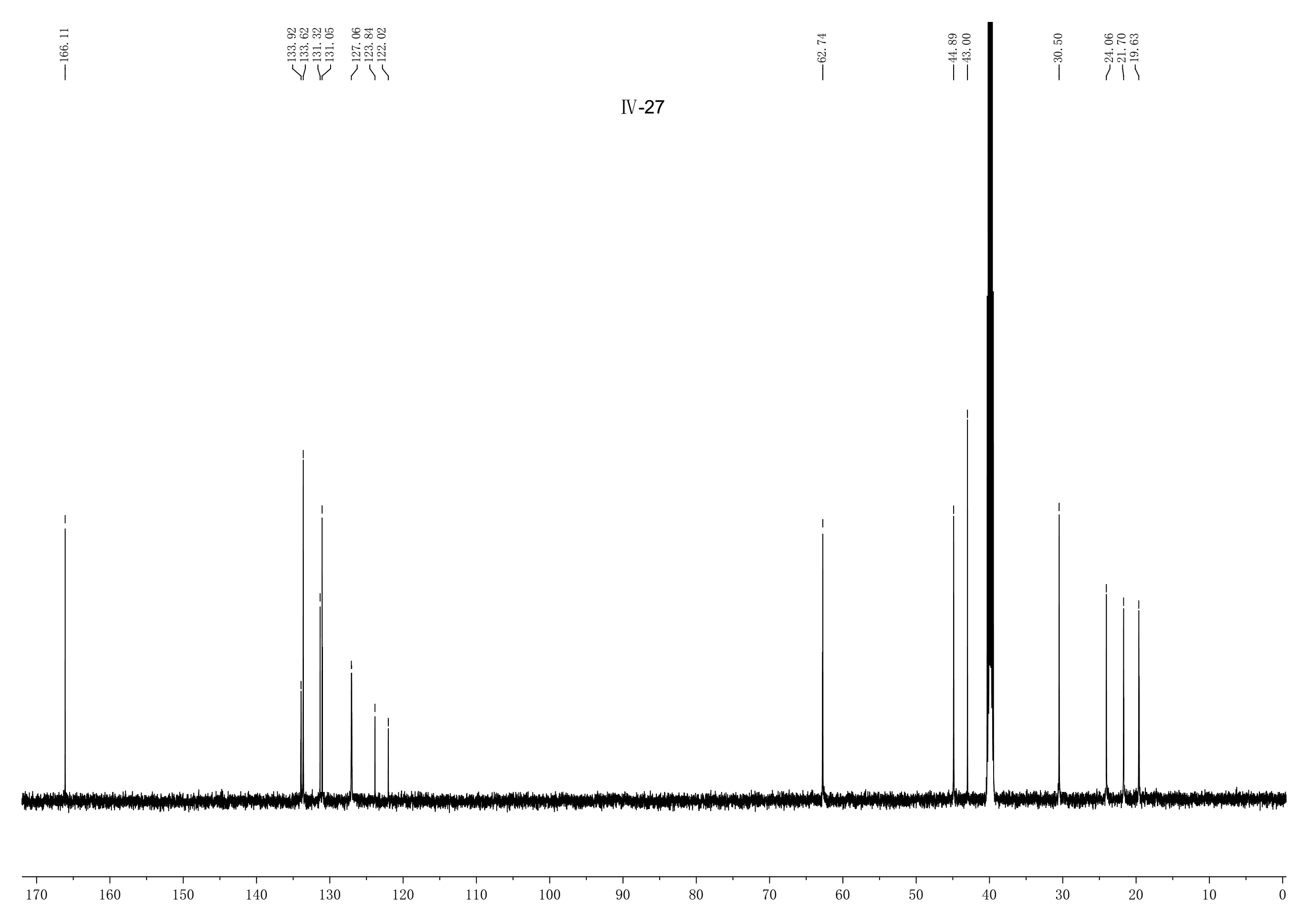


Figure S40-2 ^13^C NMR spectrum of compound **IV-27**


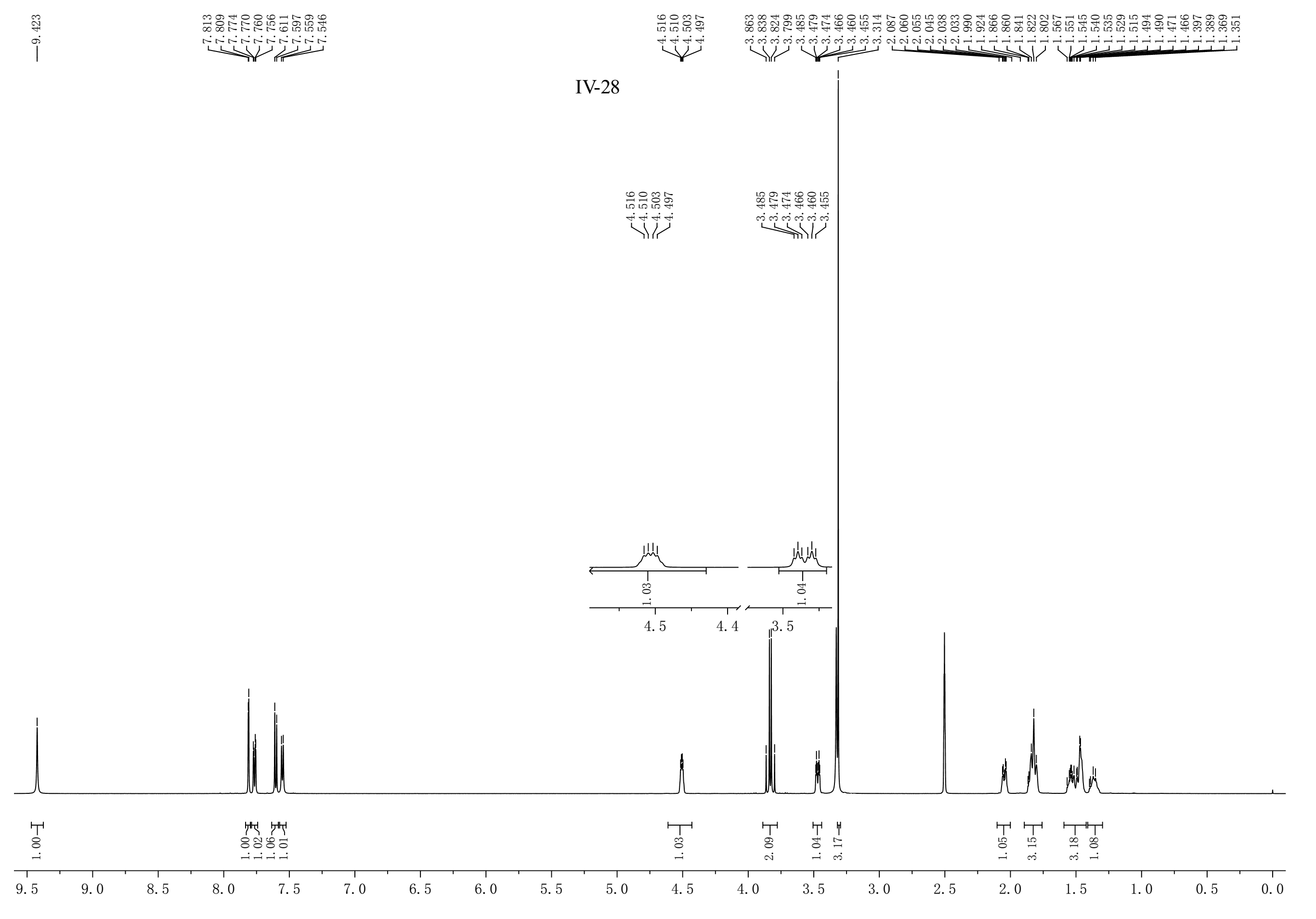


Figure S41-1 ^1^H NMR spectrum of compound **IV-28**


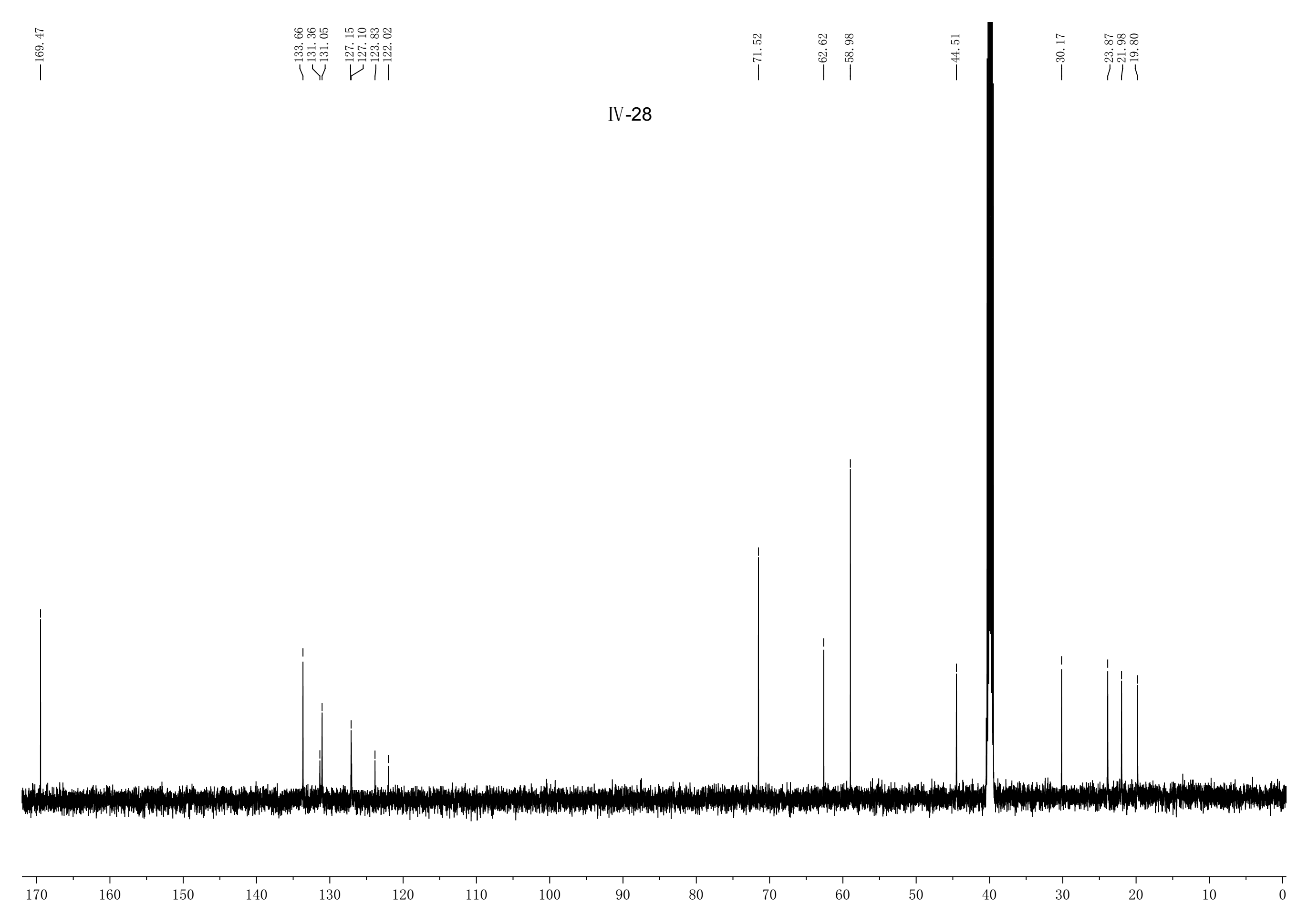


Figure S41-2 ^13^C NMR spectrum of compound **IV-28**


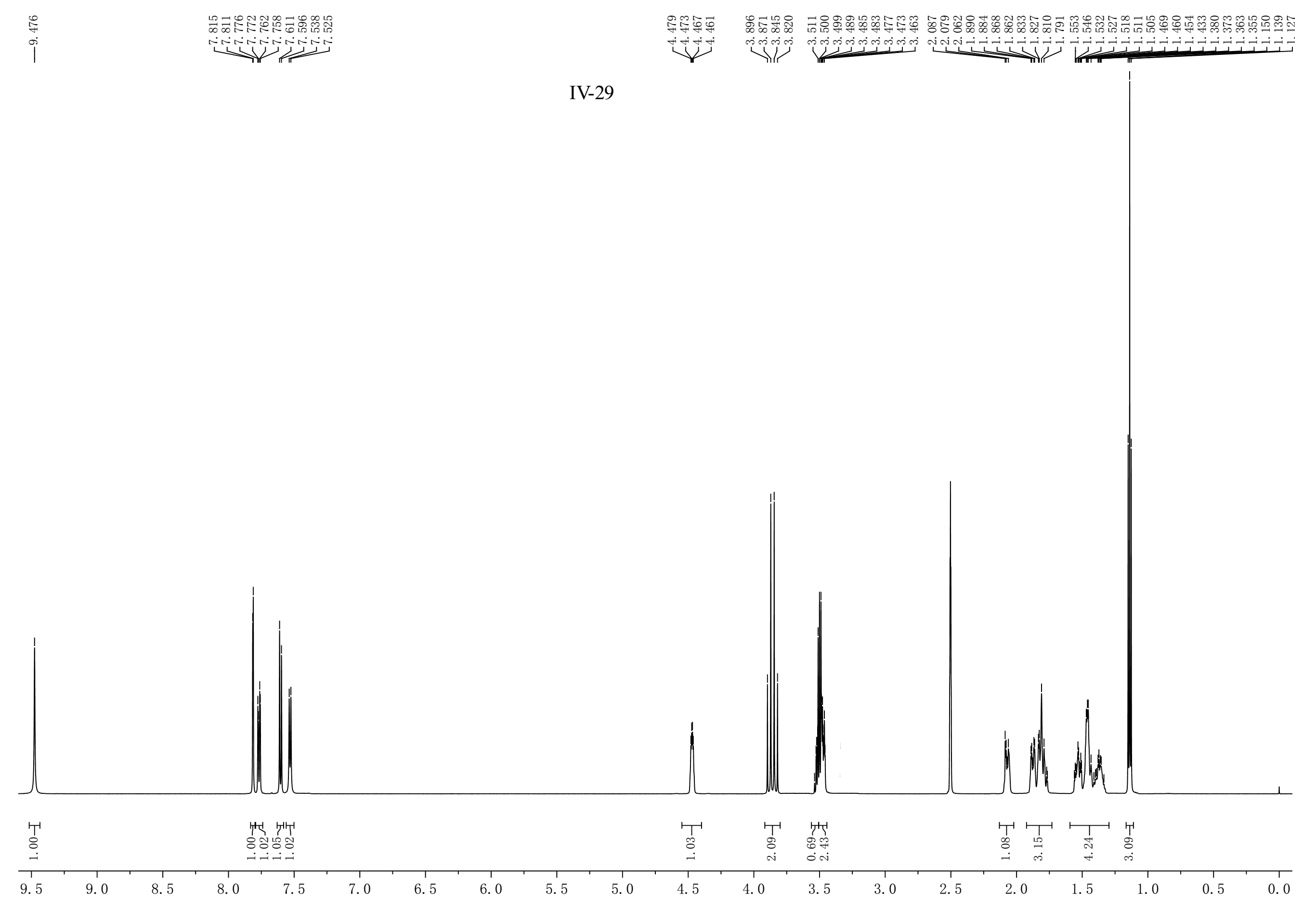


Figure S42-1 ^1^H NMR spectrum of compound **IV-29**


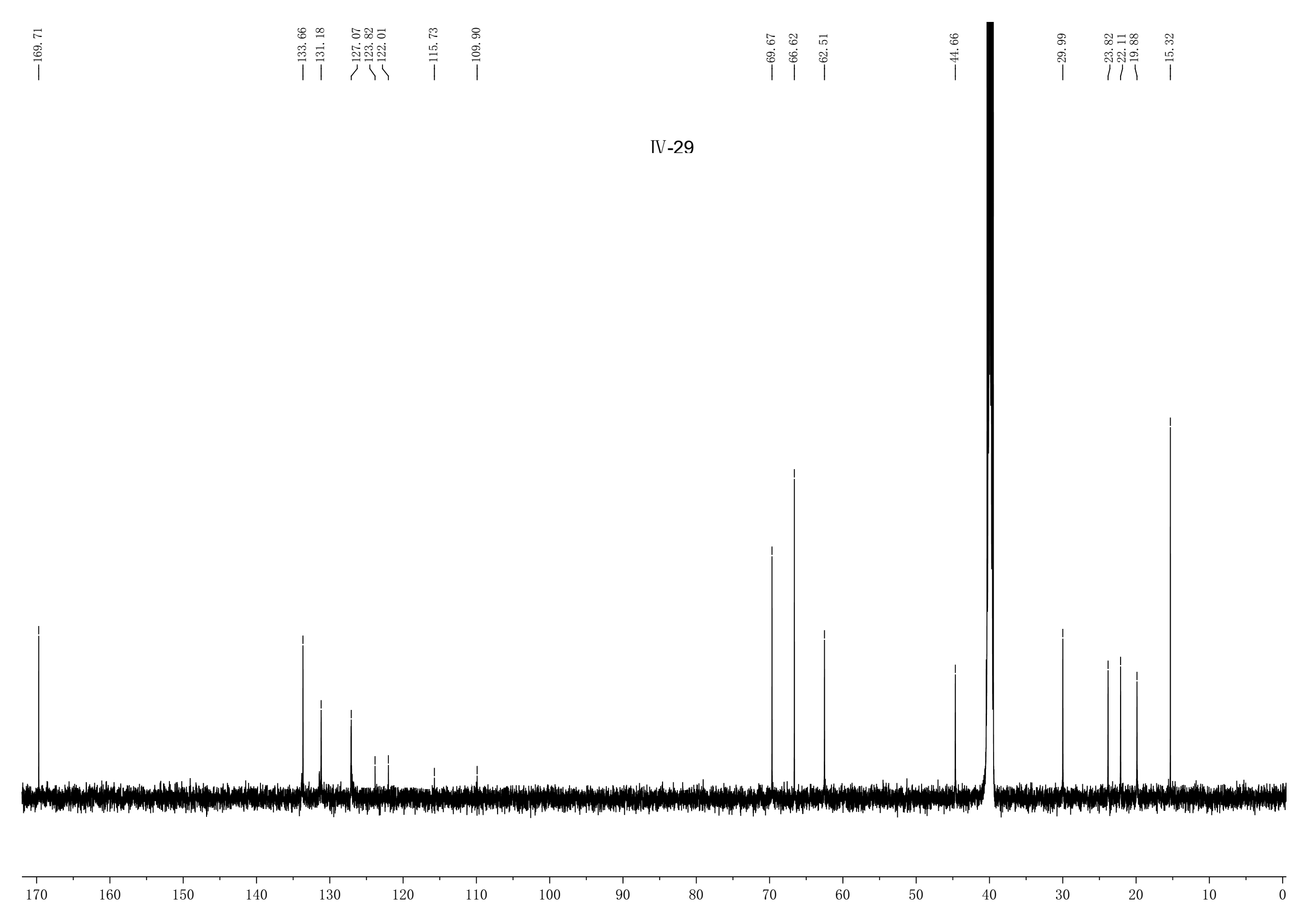


Figure S42-2 ^13^C NMR spectrum of compound **IV-29**


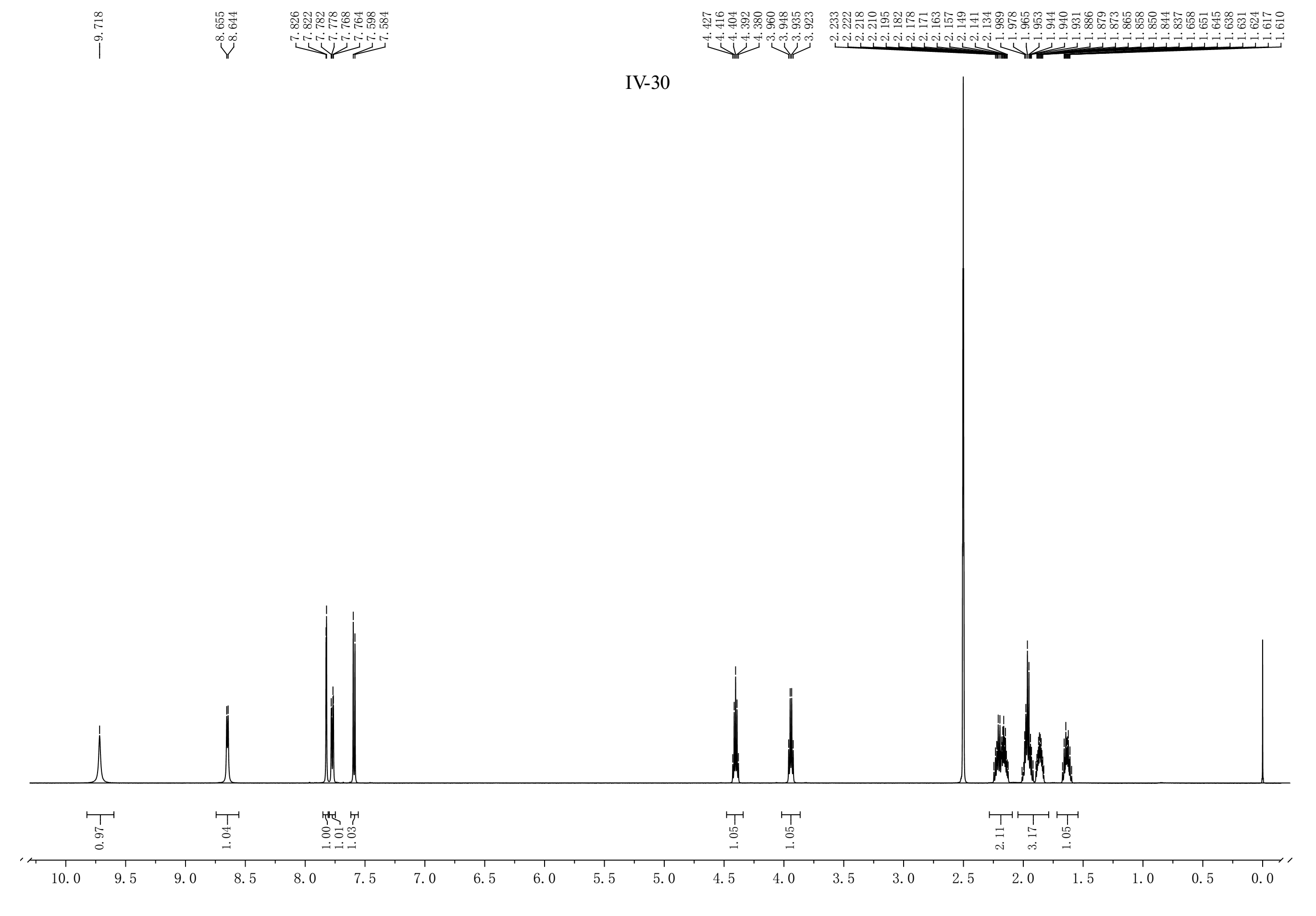


Figure S43-1 ^1^H NMR spectrum of compound **IV-30**


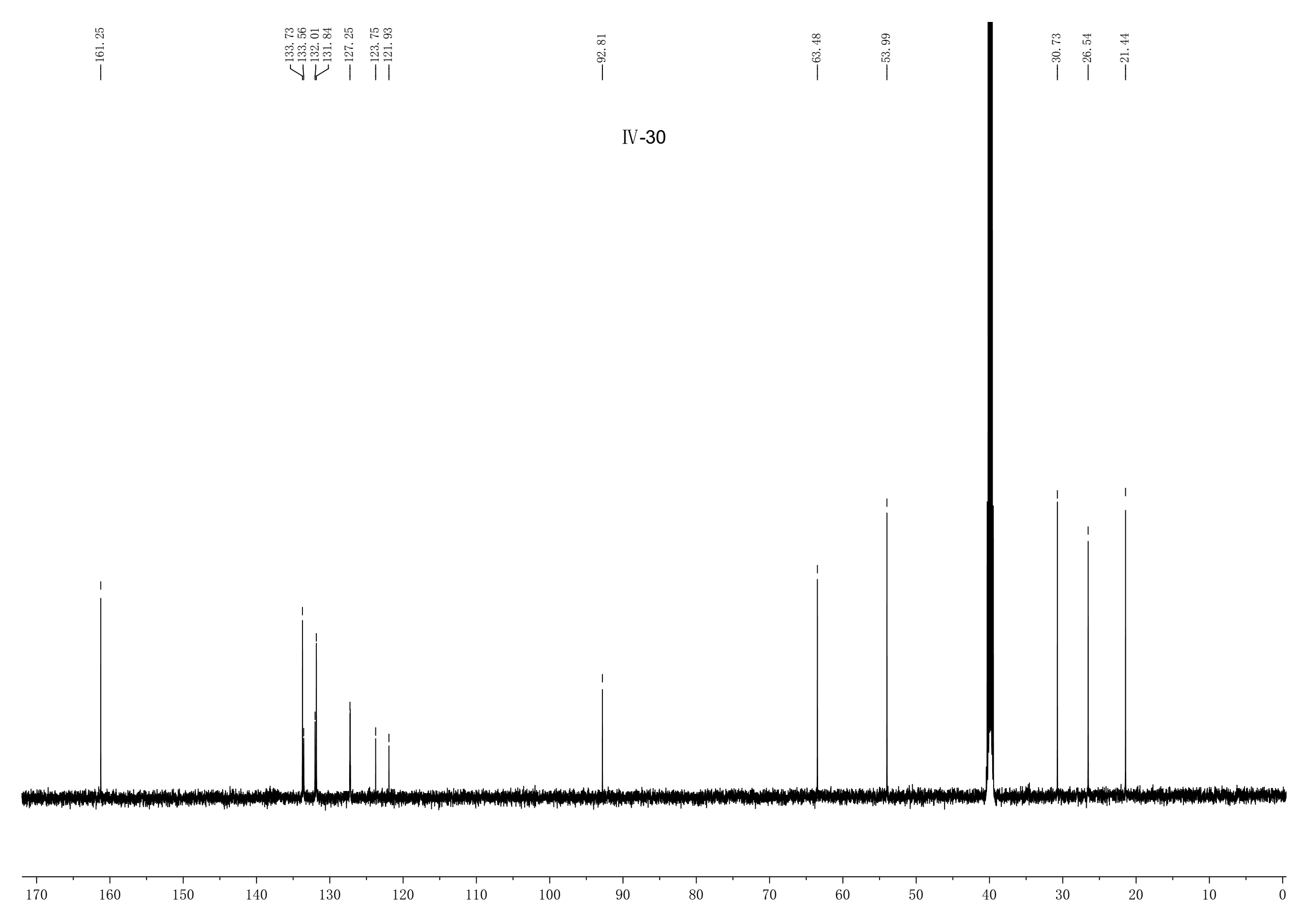


Figure S43-2 ^13^C NMR spectrum of compound **IV-30**


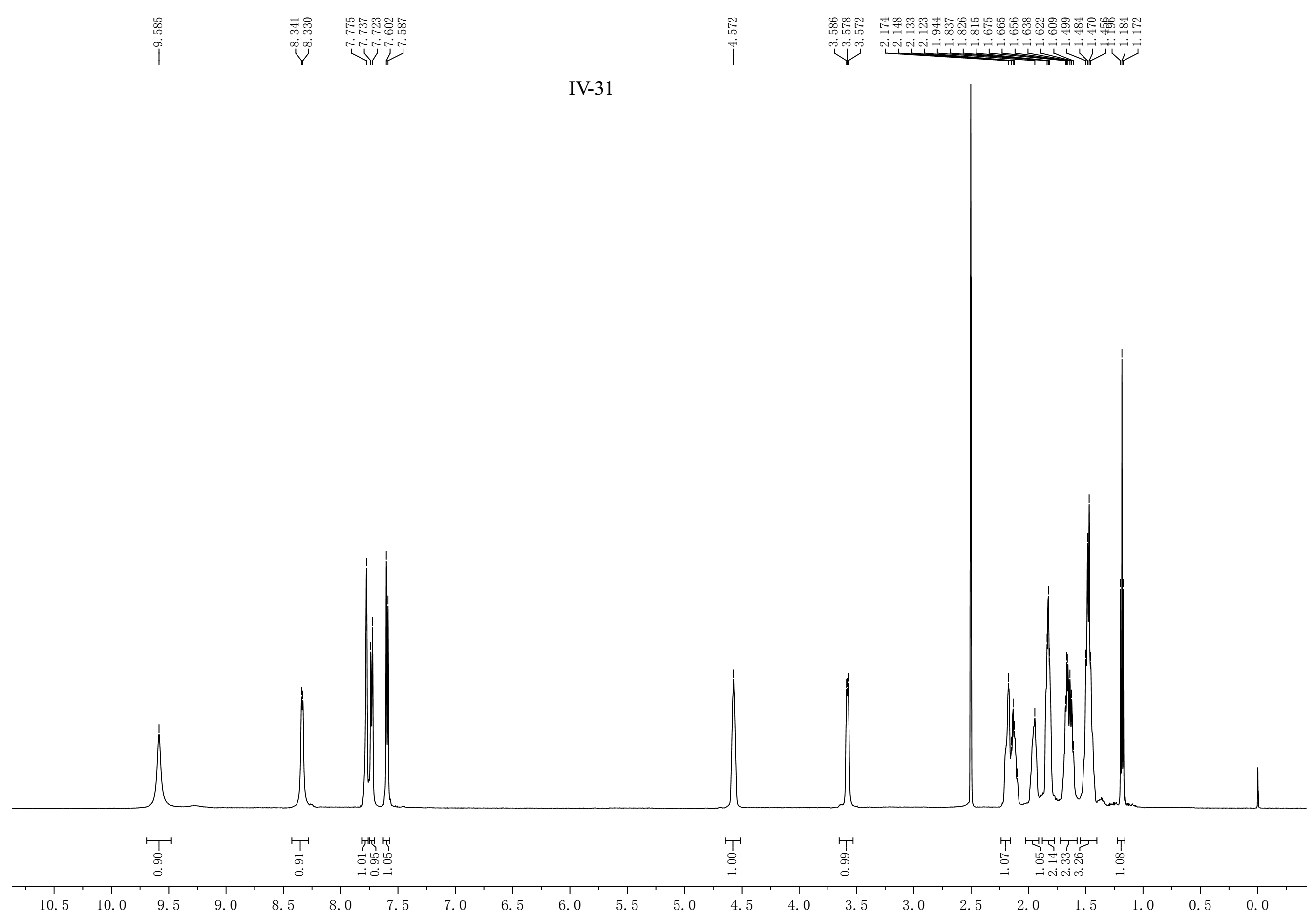


Figure S44-1 ^1^H NMR spectrum of compound **IV-31**


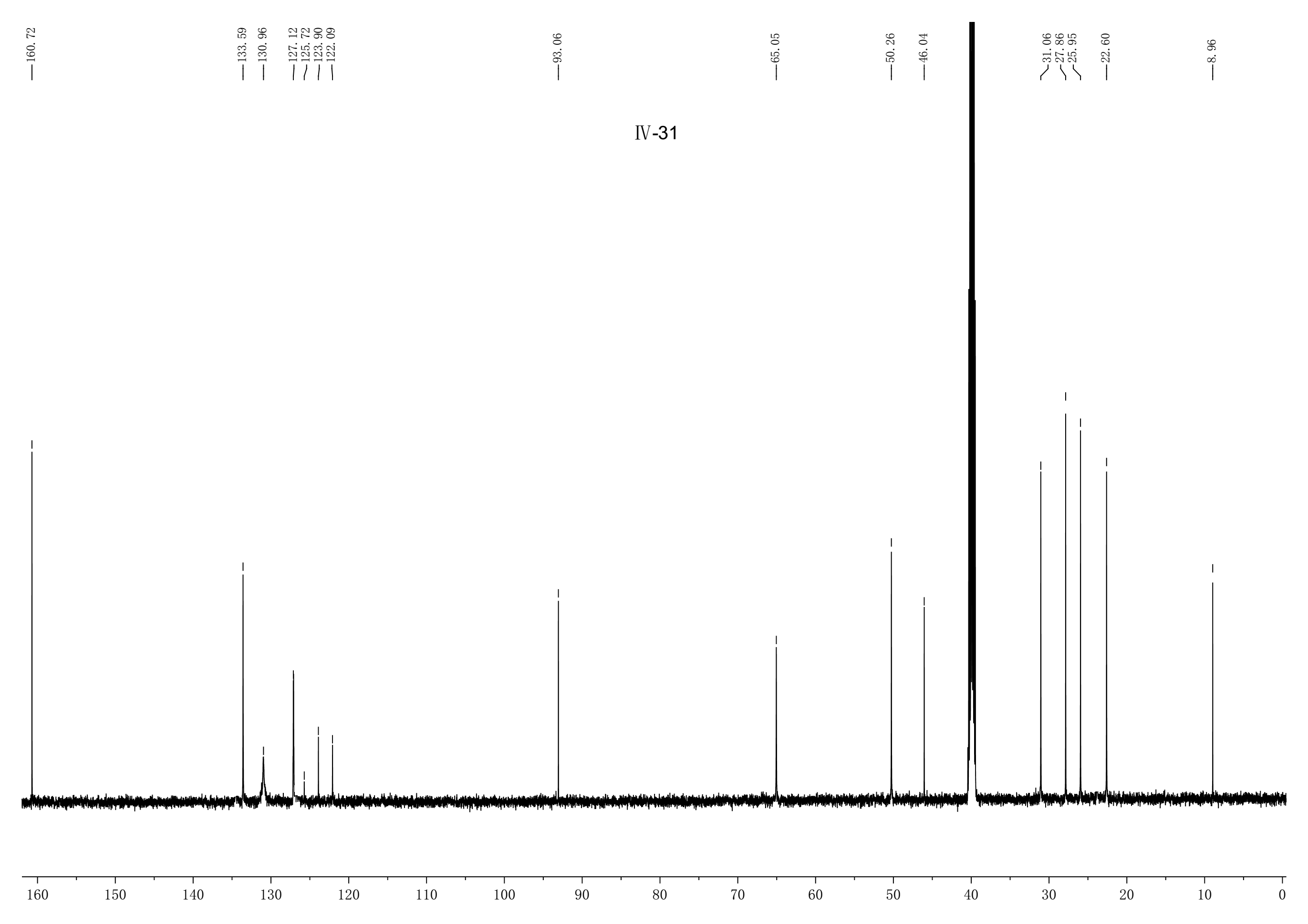


Figure S44-2 ^13^C NMR spectrum of compound **IV-31**


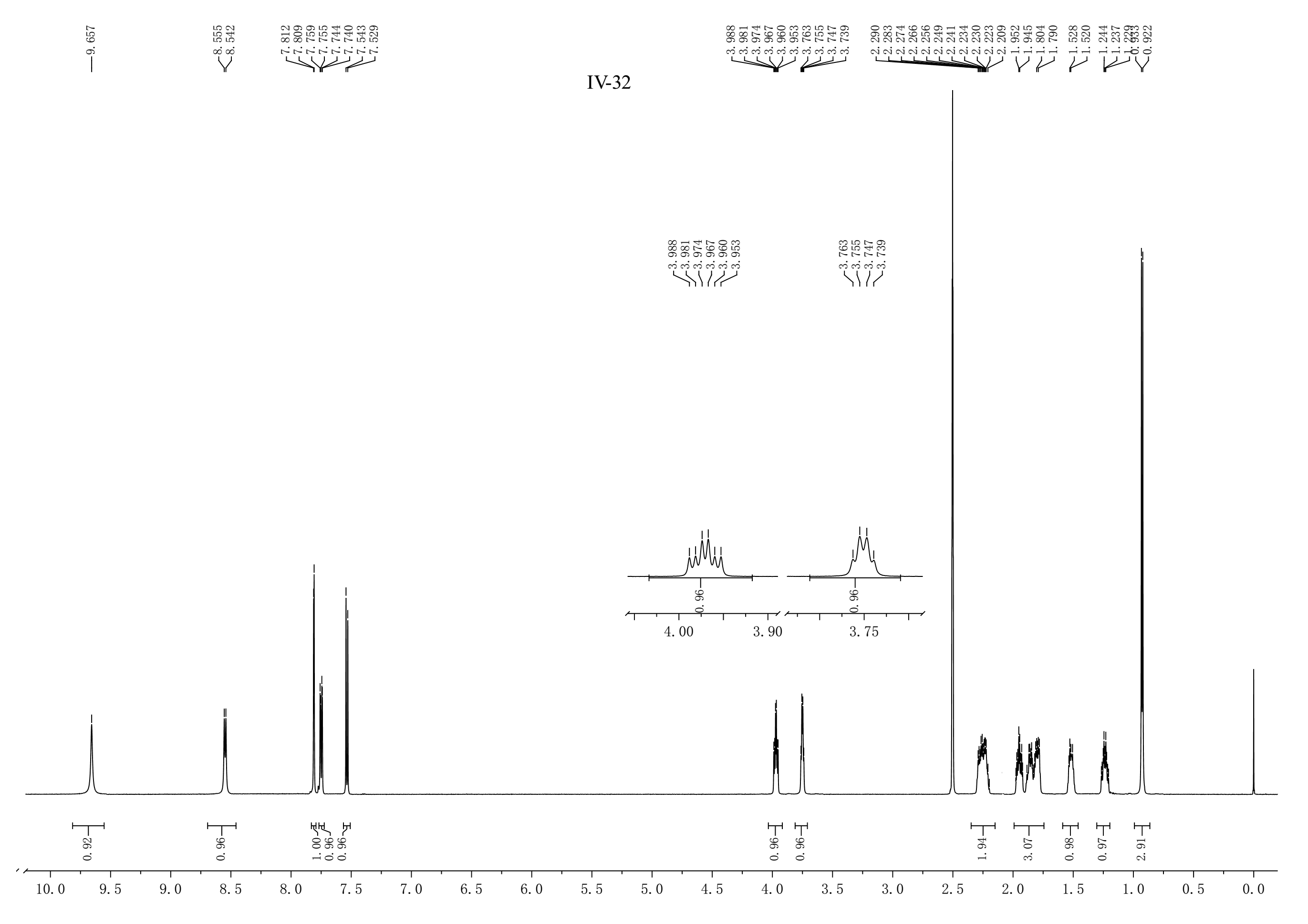


Figure S45-1 ^1^H NMR spectrum of compound **IV-32**


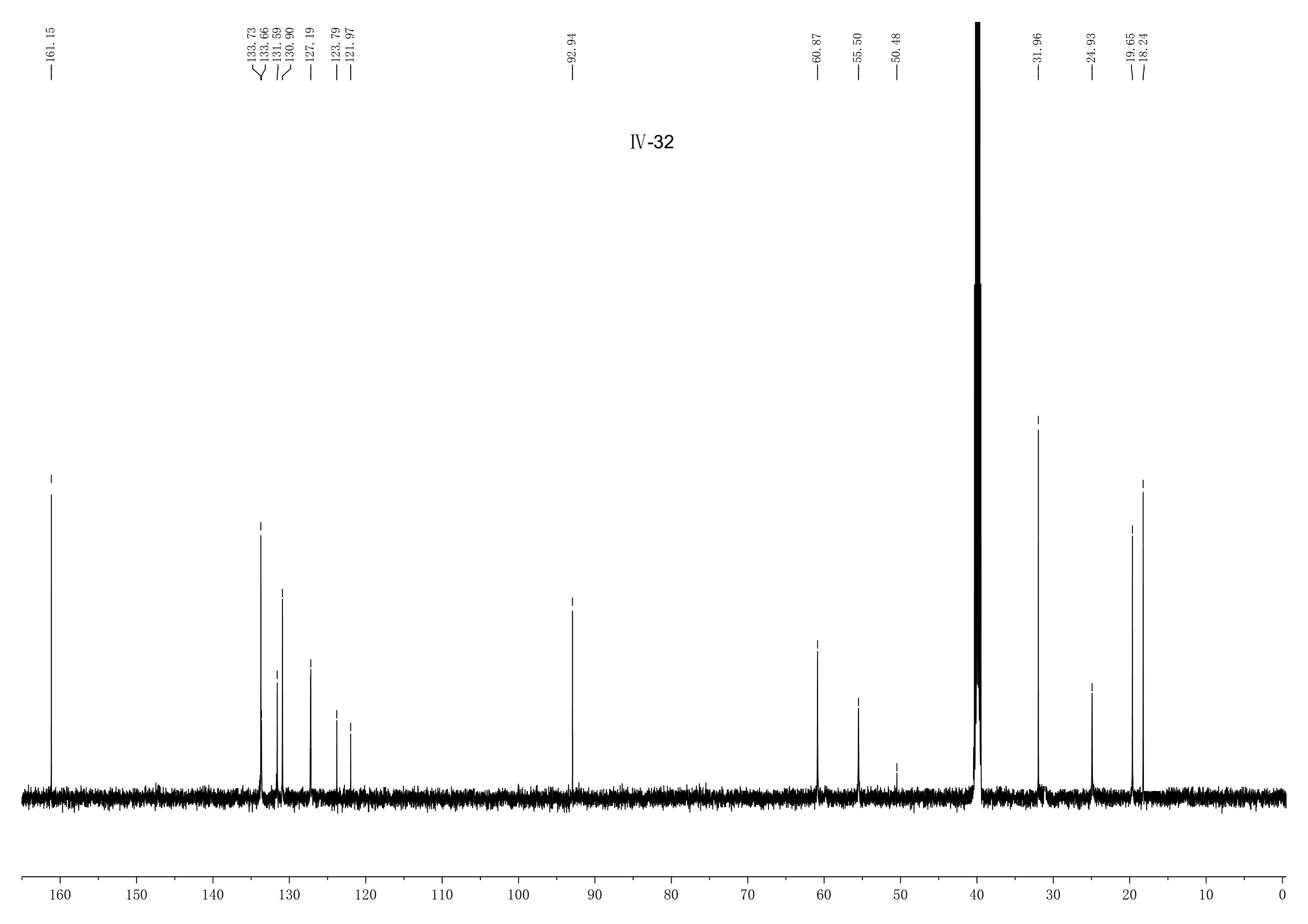


Figure S45-2 ^13^C NMR spectrum of compound **IV-32**


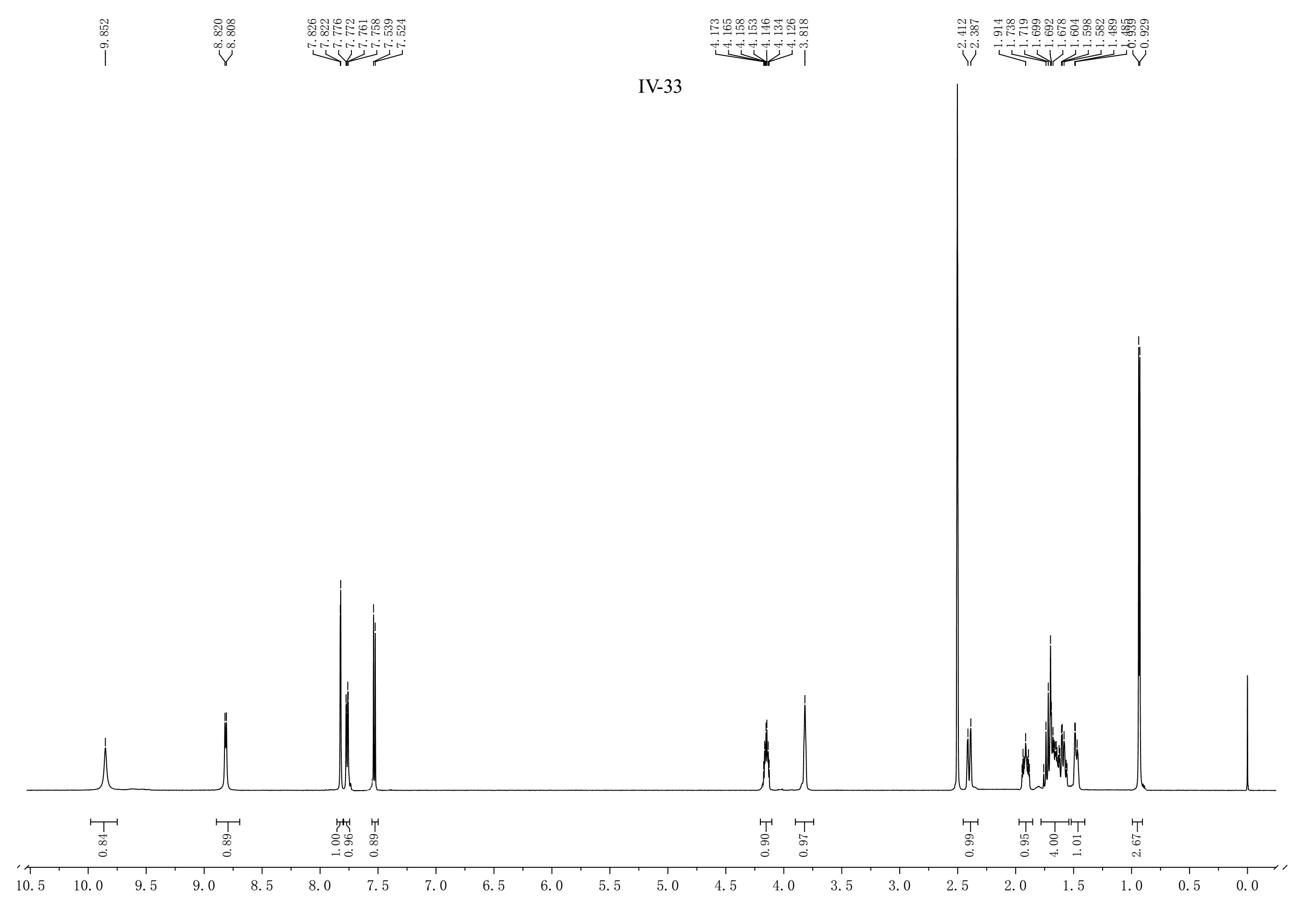


Figure S46-1 ^1^H NMR spectrum of compound **IV-33**


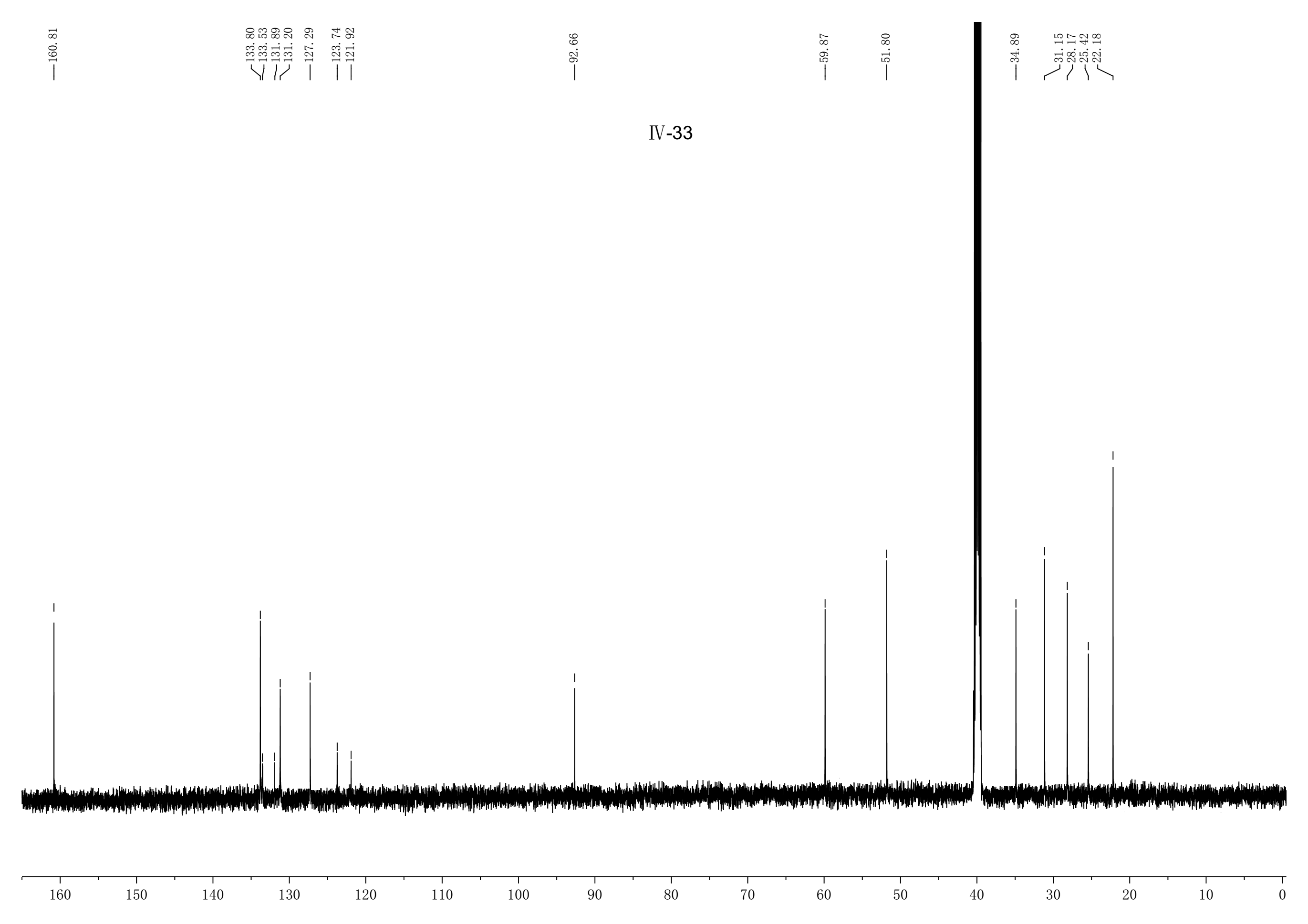


Figure S46-2 ^13^C NMR spectrum of compound **IV-33**


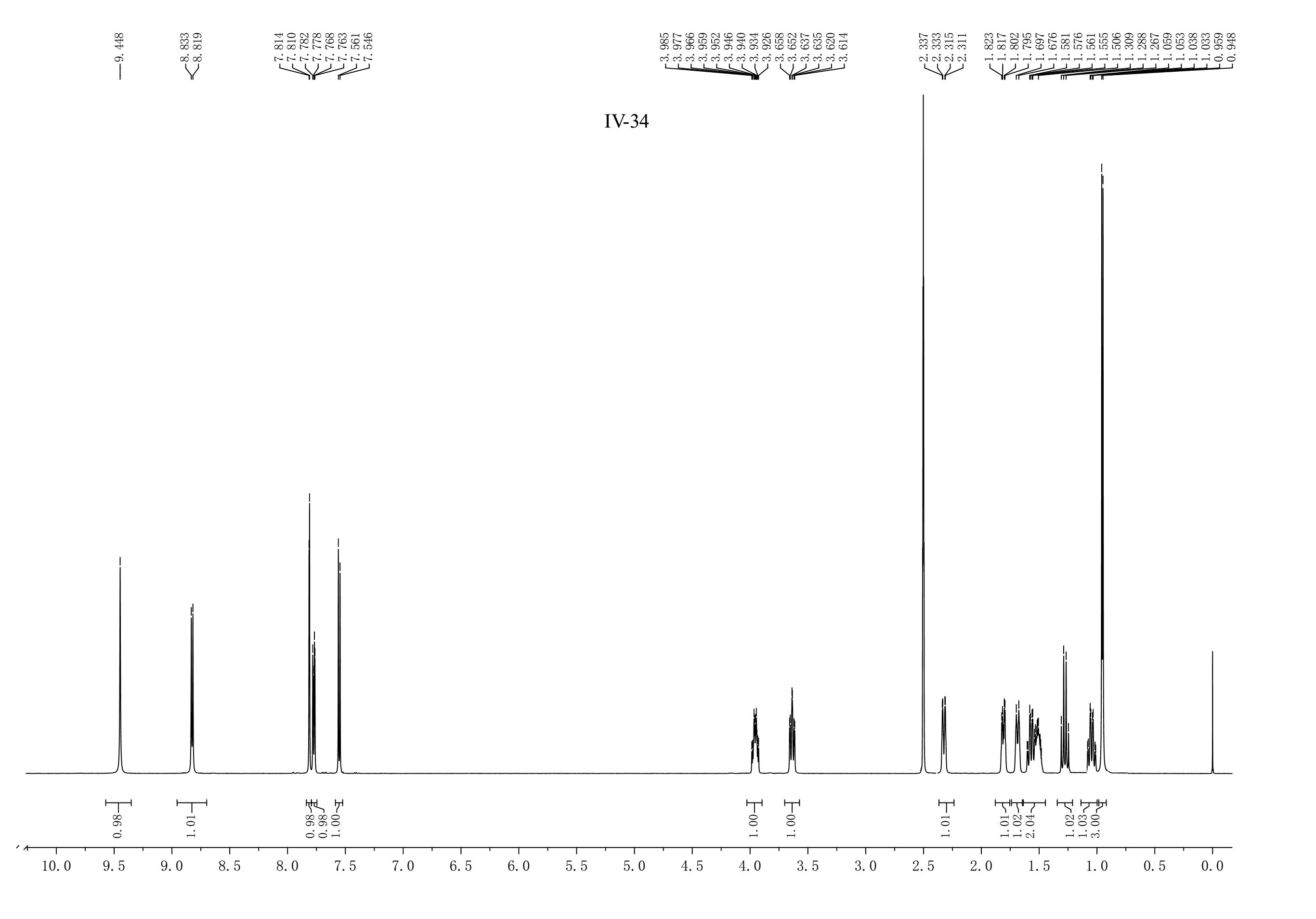


Figure S47-1 ^1^H NMR spectrum of compound **IV-34**


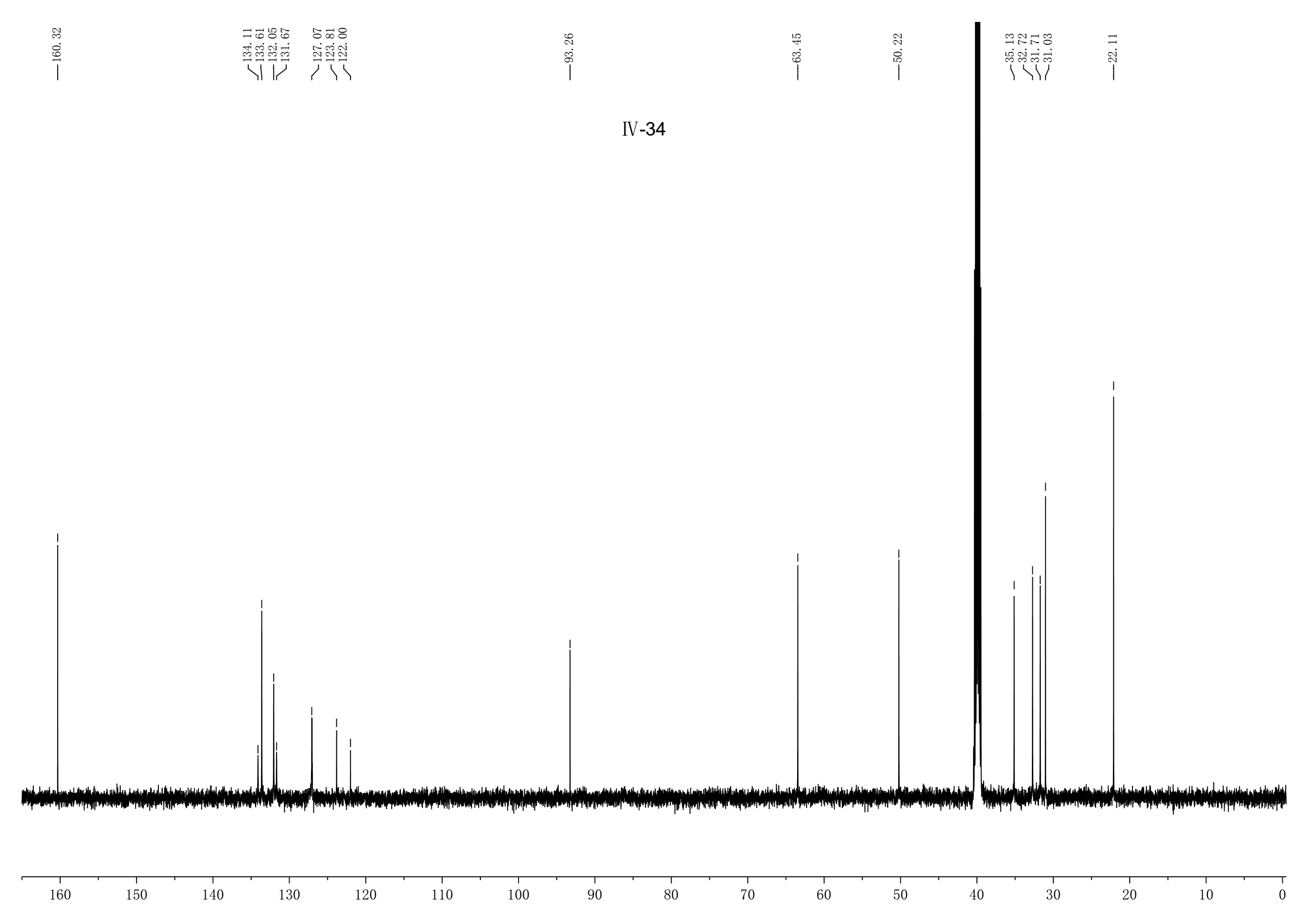


Figure S47-2 ^13^C NMR spectrum of compound **IV-34**


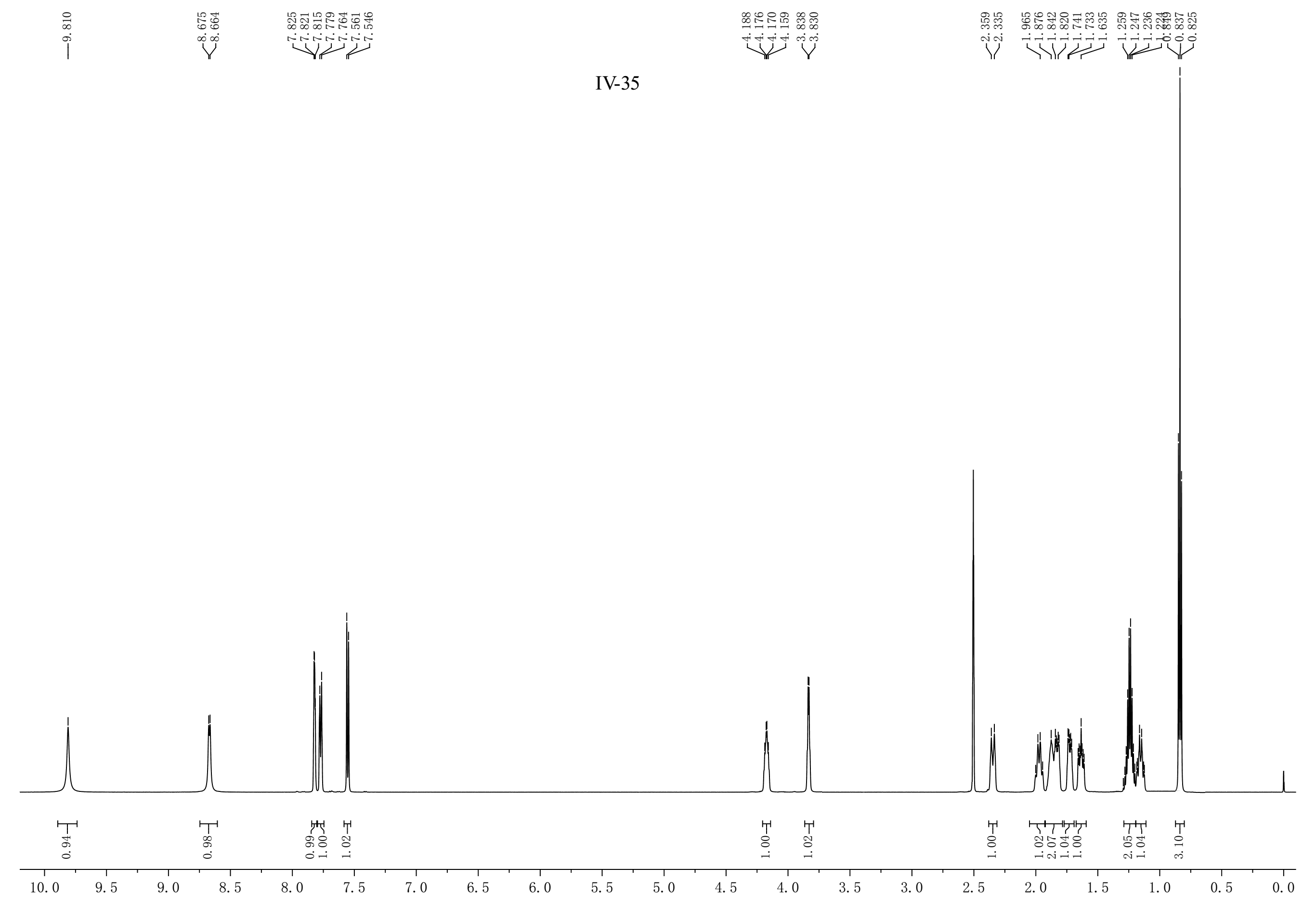


Figure S48-1 ^1^H NMR spectrum of compound **IV-35**


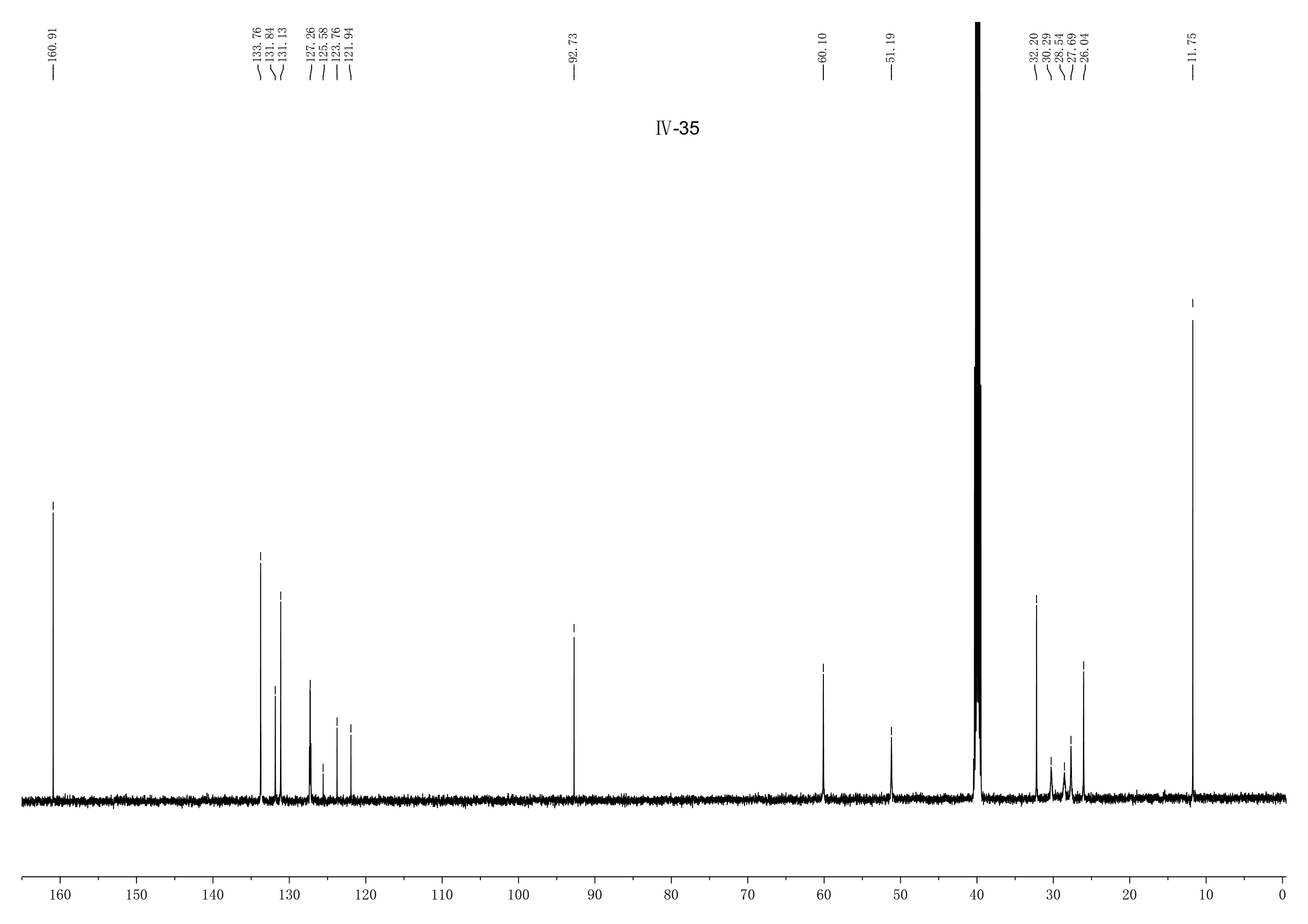


Figure S48-2 ^13^C NMR spectrum of compound **IV-35**


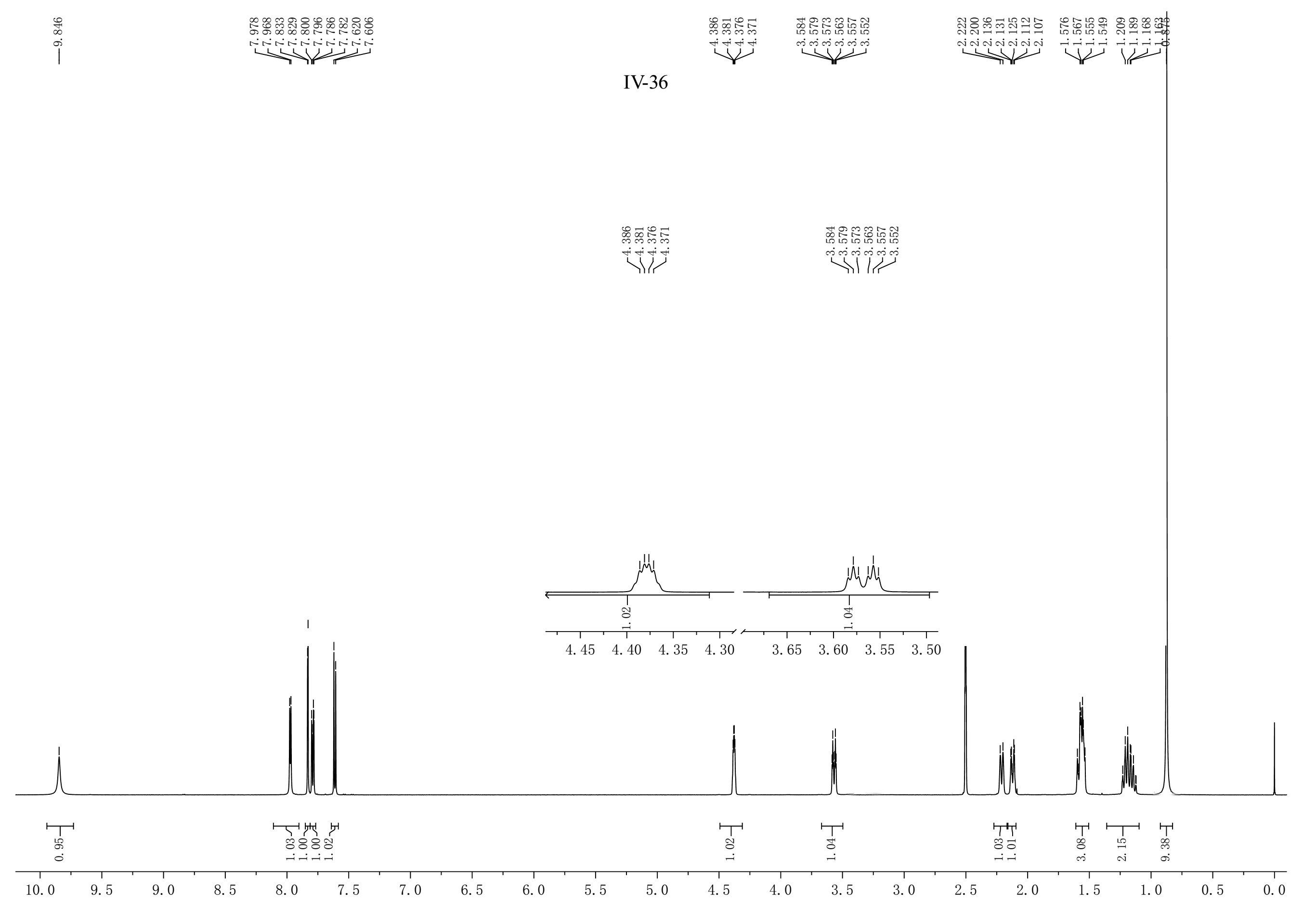


Figure S49-1 ^1^H NMR spectrum of compound **IV-36**


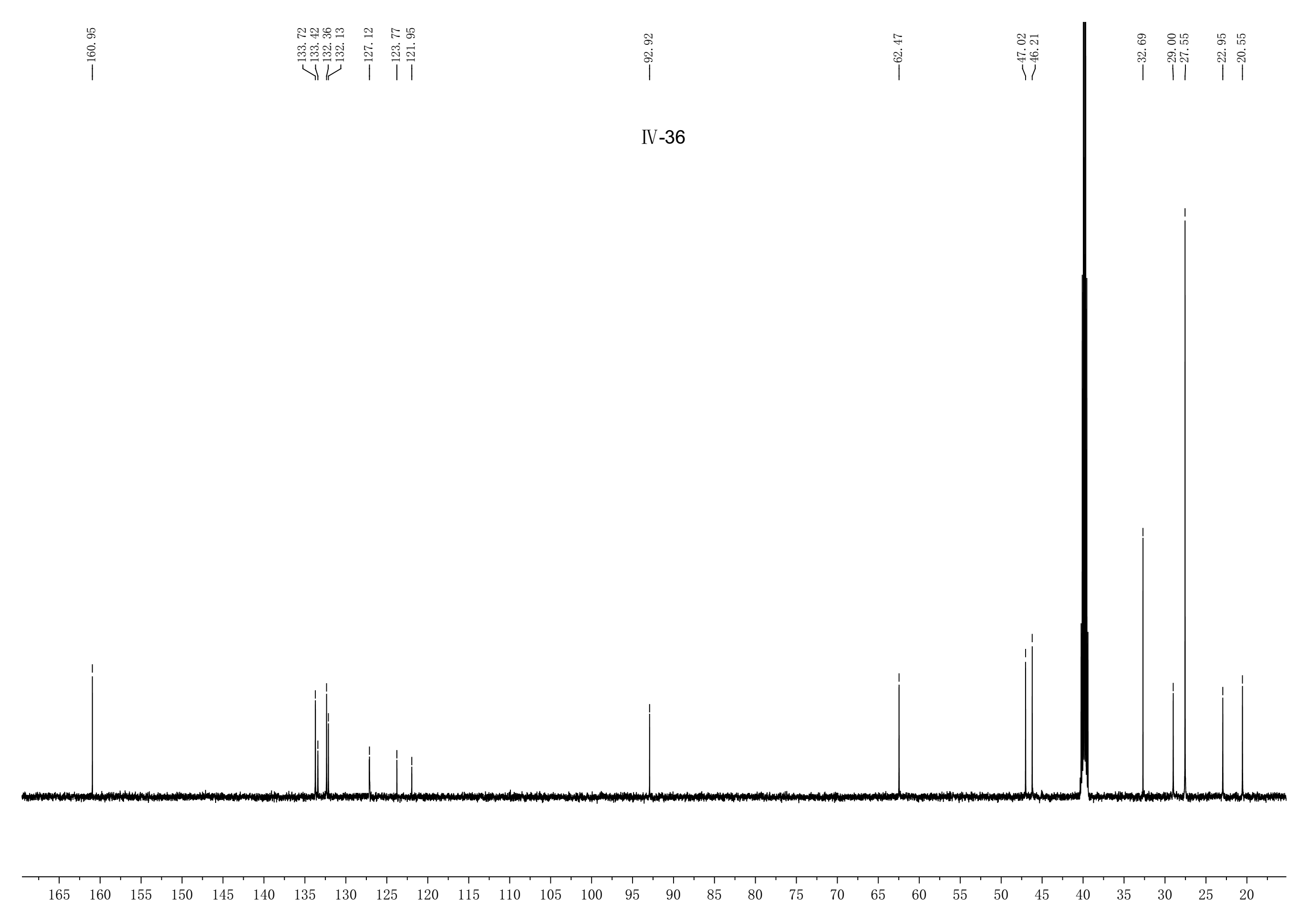


Figure S49-2 ^13^C NMR spectrum of compound **IV-36**

Table S1. Crystal and experimental data of compounds **IV-3** and **IV-31**

| **Compound** | **IV-3** | **IV-31** |
| --- | --- | --- |
| Empirical formula | C_21_H_22_ClF_3_N_2_O_3_S | C_16_H_17_Cl_4_F_3_N_2_O_3_S |
| Formula weight | 474.92 | 516.18 |
| *T* | 293(2) K | 293(2) K |
| Wavelength | 0.71073 Å | 0.71073 Å |
| Crystal system | Monoclinic | Monoclinic |
| Space group | P2(1)/n | P 1 21/n 1 (14) |
| Unit cell dimensions | a = 9.6923(9) Å,α = 90° | a = 11.2837(8)Å,α = 90° |
|  | b = 23.8041(16) Å, β = 109.123° | b = 11.3883(6)Å, β = 92.619° |
|  | c = 10.0511(8) Å, γ = 90° | c = 16.6115(11)Å, γ = 90° |
| Volume | 2191.0(3) Å^3^ | 2132.4(2)Å^3^ |
| *Z* | 4 | 4 |
| *D_x_* | 1.440 mg·m^-3^ | 1.602 mg·m^-3^ |
| Absorption coefficient | 0.321 mm^-1^ | 0.699 mm^-1^ |
| *F* (0 0 0) | 984 | 1040 |
| Crystal size | 0.03 × 0.02 × 0.01 mm | 0.02 × 0.01 × 0.01 mm |
| θ range for data collection | 3.35 to 26.50 | 2.86 to 25.50 |
| Completeness to θ = 26.50 | 99.80% | 99.80% |
| Limiting indices | -11 ≤ *h* ≤ 12, -17 ≤ *k* ≤ 29, -12 ≤ *l* ≤ 12 | -10 ≤ *h* ≤ 13, -13 ≤ *k* ≤ 13, -15 ≤ *l* ≤ 20 |
| Reflection collected/unique | 11038/4534 [R(int) = 0.0289] | 10651/3967 [R(int) = 0.0293] |
| Absorption correction | Empirical | Semi-empirical from equivalents |
| Max. and min. transmission | 0.9968 and 0.9904 | 1.00000 and 0.82794 |
| Data/restraints/parameters | 4534/0/281 | 3967/0/262 |
| Goodness-of-fit on *F*^2^ | 1.022 | 1.066 |
| Final *R* indices [*I* ＞ 2σ (*I*)] | R1 = 0.0503, wR2 = 0.1181 | R1 = 0.0529, wR2 = 0.1384 |
| (Δρ)max | 0.528 eÅ^-3^ | 0.557 eÅ^-3^ |
| (Δρ)min | -0.443 eÅ^-3^ | -0.420 eÅ^-3^ |
| Refinement method | Full-matrix least-squares on *F*^2^ | Full-matrix least-squares on *F*^2^ |
| CCDC No. | 1450549 | 1450548 |

Table S2. Bond lengths and angels of compound **IV-3**

| **No.** | **Lengths** | **(Å)** | **Angles** | **(°)** | **Torsion angles** | **(°)** |
| --- | --- | --- | --- | --- | --- | --- |
| 1 | Cl(1)-C(19) | 1.736(3) | O(2)-S(1)-O(3) | 119.71(12) | O2—S1—N2—C15 | -153.54(19) |
| 2 | S(1)-O(2) | 1.4268(18) | O(2)-S(1)-N(2) | 105.51(11) | O3—S1—N2—C15 | -23.4(2) |
| 3 | S(1)-O(3) | 1.4332(19) | O(3)-S(1)-N(2) | 109.58(11) | C14—S1—N2—C15 | 91.5(2) |
| 4 | S(1)-N(2) | 1.629(2) | O(2)-S(1)-C(14) | 108.71(11) | C9—N1—C8—O1 | -6.6(4) |
| 5 | S(1)-C(14) | 1.789(2) | O(3)-S(1)-C(14) | 106.99(12) | C9—N1—C8—C6 | 174.1(2) |
| 6 | F(1)-C(17) | 1.338(3) | N(2)-S(1)-C(14) | 105.51(11) | C7—C6—C8—O1 | -108.0(3) |
| 7 | F(2)-C(17) | 1.309(4) | C(8)-N(1)-C(9) | 121.9(2) | C4—C6—C8—O1 | 72.1(4) |
| 8 | F(3)-C(17) | 1.337(4) | C(15)-N(2)-S(1) | 123.34(16) | C7—C6—C8—N1 | 71.3(3) |
| 9 | O(1)-C(8) | 1.229(3) | O(1)-C(8)-N(1) | 122.3(3) | C4—C6—C8—N1 | -108.5(3) |
| 10 | N(1)-C(8) | 1.342(3) | O(1)-C(8)-C(6) | 122.9(2) | C8—N1—C9—C10 | -84.1(3) |
| 11 | N(1)-C(9) | 1.466(3) | N(1)-C(8)-C(6) | 114.8(2) | N1—C9—C10—C11 | -67.0(3) |
| 12 | N(2)-C(15) | 1.434(3) | N(1)-C(9)-C(10) | 111.3(2) | C12—C13—C14—S1 | -175.74(19) |
| 13 | C(1)-C(2) | 1.344(6) | N(1)-C(9)-C(14) | 111.4(2) | N1—C9—C14—C13 | 66.0(3) |
| 14 | C(3)-C(4) | 1.399(5) | C(13)-C(14)-S(1) | 112.15(17) | N1—C9—C14—S1 | -61.2(3) |
| 15 | C(4)-C(6) | 1.393(4) | C(9)-C(14)-S(1) | 111.91(18) | C10—C9—C14—S1 | 176.0(2) |
| 16 | C(4)-C(5) | 1.490(5) | C(21)-C(15)-N(2) | 119.8(2) | O2—S1—C14—C13 | -48.2(2) |
| 17 | C(6)-C(7) | 1.373(4) | C(16)-C(15)-N(2) | 121.3(2) | N2—S1—C14—C13 | 64.57(19) |
| 18 | C(6)-C(8) | 1.488(4) | F(2)-C(17)-F(3) | 106.4(3) | O3—S1—C14—C9 | -51.4(2) |
| 19 | C(9)-C(10) | 1.531(4) | F(2)-C(17)-F(1) | 106.9(3) | N2—S1—C14—C9 | -168.09(17) |
| 20 | C(9)-C(14) | 1.535(3) | F(3)-C(17)-F(1) | 105.0(3) | S1—N2—C15—C21 | 59.6(3) |
| 21 | C(10)-C(11) | 1.513(5) | F(2)-C(17)-C(16) | 114.4(3) | N2—C15—C16—C17 | 3.0(4) |
| 22 | C(11)-C(12) | 1.516(5) | F(3)-C(17)-C(16) | 111.8(2) | C15—C16—C17—F2 | -52.9(4) |
| 23 | C(12)-C(13) | 1.530(4) | F(1)-C(17)-C(16) | 111.7(2) | C18—C16—C17—F1 | 6.4(4) |
| 24 | C(13)-C(14) | 1.524(4) | C(20)-C(19)-Cl(1) | 119.9(2) | C16—C18—C19—Cl1 | -177.8(2) |
| 25 | C(15)-C(21) | 1.381(3) | C(18)-C(19)-Cl(1) | 118.9(2) | N2—C15—C21—C20 | 179.5(2) |

Table S3. Bond lengths and angels of compound **IV-31**

| **No.** | **Lengths** | **(Å)** | **Angles** | **(°)** | **Torsion angles** | **(°)** |
| --- | --- | --- | --- | --- | --- | --- |
| 1 | Cl1—C1 | 1.746(4) | O2—S1—O1 | 118.87(15) | O2—S1—N1—C16 | 172.2(2) |
| 2 | Cl2—C1 | 1.754(4) | O2—S1—N1 | 105.13(14) | O1—S1—N1—C16 | 44.3(3) |
| 3 | Cl3—C1 | 1.758(4) | O1—S1—N1 | 108.02(14) | C9—S1—N1—C16 | -70.7(3) |
| 4 | Cl4—C13 | 1.733(4) | O2—S1—C9 | 110.52(14) | C3—N2—C2—O3 | -5.4(5) |
| 5 | S1—O2 | 1.424(2) | O1—S1—C9 | 107.53(14) | C3—N2—C2—C1 | 171.7(3) |
| 6 | S1—O1 | 1.439(2) | N1—S1—C9 | 106.02(14) | Cl1—C1—C2—O3 | -15.2(4) |
| 7 | S1—N1 | 1.647(3) | C16—N1—S1 | 122.1(2) | Cl2—C1—C2—O3 | -135.9(3) |
| 8 | S1—C9 | 1.807(3) | C2—N2—C3 | 122.7(3) | Cl3—C1—C2—O3 | 104.7(3) |
| 9 | F1—C10 | 1.309(5) | C2—C1—Cl1 | 110.5(3) | Cl1—C1—C2—N2 | 167.5(3) |
| 10 | F2—C10 | 1.301(4) | Cl1—C1—Cl2 | 108.5(2) | Cl2—C1—C2—N2 | 46.8(4) |
| 11 | F3—C10 | 1.303(4) | Cl1—C1—Cl3 | 110.1(2) | Cl3—C1—C2—N2 | -72.6(3) |
| 12 | O3—C2 | 1.208(4) | Cl2—C1—Cl3 | 109.2(2) | C2—N2—C3—C4 | 109.3(4) |
| 13 | N1—C16 | 1.433(4) | O3—C2—N2 | 126.9(3) | N2—C3—C4—C5 | -157.7(3) |
| 14 | N2—C2 | 1.323(4) | O3—C2—C1 | 120.2(3) | C7—C8—C9—S1 | -113.5(3) |
| 15 | N2—C3 | 1.465(4) | N2—C2—C1 | 112.9(3) | N2—C3—C9—S1 | -70.4(3) |
| 16 | C1—C2 | 1.559(5) | N2—C3—C4 | 110.7(3) | C4—C3—C9—S1 | 55.7(3) |
| 17 | C3—C4 | 1.525(5) | N2—C3—C9 | 108.7(3) | O2—S1—C9—C8 | 40.1(3) |
| 18 | C3—C9 | 1.545(4) | C4—C3—C9 | 116.9(3) | O1—S1—C9—C8 | 171.4(2) |
| 19 | C4—C5 | 1.528(6) | C3—C4—C5 | 114.4(3) | N1—S1—C9—C8 | -73.3(2) |
| 20 | C5—C6 | 1.508(6) | C8—C9—S1 | 111.1(2) | O2—S1—C9—C3 | -91.8(2) |
| 21 | C6—C7 | 1.523(6) | C3—C9—S1 | 111.9(2) | O1—S1—C9—C3 | 39.4(2) |
| 22 | C7—C8 | 1.505(5) | F2—C10—F3 | 106.0(4) | N1—S1—C9—C3 | 154.8(2) |
| 23 | C8—C9 | 1.544(4) | F2—C10—F1 | 105.1(4) | F1—C10—C11—C12 | 108.1(4) |
| 24 | C10—C11 | 1.487(5) | F3—C10—F1 | 106.5(4) | F1—C10—C11—C16 | -72.4(5) |
| 25 | C11—C12 | 1.393(5) | F2—C10—C11 | 113.6(3) | C11—C12—C13—Cl4 | 176.8(3) |
| 26 | C11—C16 | 1.399(4) | F3—C10—C11 | 112.8(3) | C14—C15—C16—N1 | 177.4(3) |
| 27 | C12—C13 | 1.363(5) | F1—C10—C11 | 112.2(3) | C12—C11—C16—N1 | -179.5(3) |
| 28 | C13—C14 | 1.366(6) | C12—C11—C10 | 117.6(3) | C10—C11—C16—N1 | 1.1(5) |
| 29 | C14—C15 | 1.394(6) | C12—C13—Cl4 | 119.2(3) | S1—N1—C16—C15 | -58.5(4) |
| 30 | C15—C16 | 1.377(5) | C11—C16—N1 | 122.4(3) | S1—N1—C16—C11 | 123.9(3) |
